# Validated bioanalysis of oxylipins confirms specialized pro-resolving mediator formation in vitro and in vivo

**DOI:** 10.1016/j.jlr.2026.101075

**Published:** 2026-06-08

**Authors:** Robert K. Hofstetter, Markus Werner, Patrick Schädel, Mareike Wichmann-Costaganna, Katrin Fischer, Vera Bruggink, Katharina P.L. Meyer, Lukas Peltner, Kerstin Günther, Vivien Bachmann, Hannes Engelbrecht, Clemens Gutjahr, Bill Perkowski, Gregor Griebel, Nur Banu Bal, Nico Ueberschaar, Paul M. Jordan, Oliver Werz

**Affiliations:** 1 Department of Pharmaceutical/Medicinal Chemistry, Institute of Pharmacy, Friedrich Schiller University Jena, Jena, Germany; 2 Department of Pharmacology, Faculty of Pharmacy, Gazi University, Ankara, Turkey; 3 Mass Spectrometry Platform, Friedrich Schiller University Jena, Jena, Germany; 4 Jena Center for Soft Matter (JCSM), Friedrich Schiller University Jena, Jena, Germany

**Keywords:** inflammation, cell signaling, lipoxygenase, omega-3 fatty acids, arachidonic acid, oxidized lipids, lipidomics, mass spectrometry, liquid chromatography, validation

## Abstract

Specialized pro-resolving mediators (SPM) are di- or trihydroxylated polyunsaturated fatty acids with potent inflammation-resolving features. SPM typically occur at lower concentrations than classical pro-inflammatory eicosanoids, making their detection highly challenging. Thus, reporting of biological SPM levels has been discrepant and not always transparent, sparking uncertainty concerning the formation capacity for di- and especially for trihydroxylated SPM in vitro and in vivo. Here, we create common ground by providing a systematic and comprehensive assessment of SPM formation across a broad range of commonly employed in vitro, ex vivo, and in vivo matrices. For this purpose, a quantitative UHPLC-MS/MS method targeting 72 oxylipins (including 19 SPM) was validated in accordance with recommendations by the International Lipidomics Society. By including sample preparation (solid phase extraction) in all validation processes, a conservative lower limit of quantification (10–100 pg/ml matrix) was found to meet clearly defined criteria for signal/noise, accuracy, precision, selectivity, specificity, matrix effects, recovery, and carry-over for 19 SPM. Our results offer analytically defensible reference data to demonstrate (*i*) the absence of relevant SPM levels in stimulated whole blood and unstimulated cell models; (*ii*) only trace SPM formation in stimulated peripheral blood mononuclear cells, M1-macrophages, neutrophils, and platelet incubations; but (*iii*) significant SPM formation in stimulated M2a-macrophages and neutrophil/platelet co-incubations. In healthy C57BL/6JRj mice, SPM formation was low yet organ-specific, with relevant amounts of dihydroxylated SPM detected in spleen. Taken together, we demonstrate SPM formation to be robust, matrix- and stimulus-dependent, but confined to specific di- and trihydroxylated SPM.

## Graphical abstract

**Figure:**
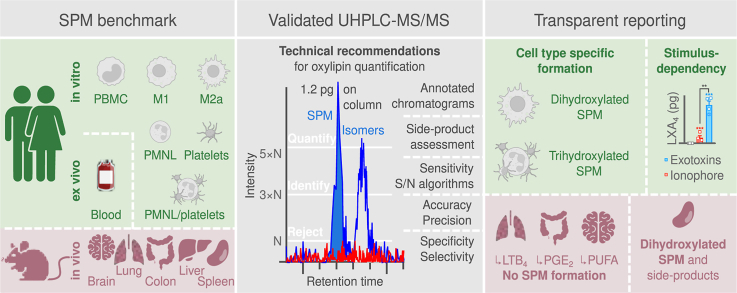

Lipid mediators (LM) encompass polyunsaturated fatty acid (PUFA) metabolites, phospholipids, lysophospholipids, and other bioactive lipids that act as local (autacoid) signaling molecules (1). Within the chemically diverse set of LM, the term “oxylipin” was introduced to specifically describe oxygenase-catalyzed PUFA metabolites (2). In humans, PUFA oxygenation is catalyzed by multiple isoforms of lipoxygenases (LOX), cyclooxygenases (COX), and cytochrome P450 (CYP) at isoform-specific sites (3).

Classical oxylipins such as prostaglandins (PG), thromboxanes (TX), and leukotrienes (LT) regulate basic physiological processes, including the immune response, endothelial function, coagulation, and parturition (4), while aberrant production of these oxylipins leads to pathophysiological processes such as allergic reactions and chronic inflammation (5). Accordingly, the quantification of oxylipins from biological matrices has been proven clinically relevant for estimating the status of many inflammatory diseases, including infectious, cardiovascular, metabolic, neurological, and neoplastic disorders (6).

Unlike the largely pro-inflammatory eicosanoids, the specialized pro-resolving mediators (SPM) are a relatively novel class of oxylipins comprising linear, di-, or trihydroxylated fatty acids for which potent anti-inflammatory and inflammation-resolving features have been demonstrated (7). The anti-inflammatory effects include the suppression of endothelial adhesion molecules (thereby dampening neutrophil invasion into the inflamed tissue), while the pro-resolving features are reflected by elevated efferocytosis, thereby expediting the removal of apoptotic cells that would otherwise obstruct catabasis (i.e., the return to homeostasis) (8). SPM exert these effects via G-protein-coupled receptors, although the extent to which this involves orthosteric activation of SPM-specific orphan receptors (9) as opposed to allosteric modulation of the PGE_2_ receptor EP4 (10) remains to be seen. Similarly, the enzymatic pathways for SPM biosynthesis remain to be conclusively mapped for many SPM but indicate a pivotal role of 15-LOX-1/2 in combination with other LOX isoforms, COX or CYP enzymes (11).

Although numerous studies have validated the anti-inflammatory/pro-resolving effects of exogenously applied SPM (12), the detection of endogenous SPM formation has shown considerable variability across studies (11). This is likely because putative SPM—owing to their high potency—occur at very low concentrations, are accompanied by numerous isomers of unknown potency and must be quantified against a background of precursors that are typically several orders of magnitude more abundant (13). Due to this panoply of unknown but potentially cross-reactive species, technical recommendations deprecate ELISA and instead endorse liquid (LC) or supercritical fluid chromatography (SFC) with detection by mass spectrometry (MS) to achieve the selectivity and sensitivity required for SPM quantification (14). Nevertheless, endogenous SPM formation continues to be reported by ELISA (15, 16, 17). Moreover, even LC-MS/MS-based methods have not always been transparently validated – especially with regard to the limit of detection (LOD) and the lower limit of quantification (LLOQ) – or reported (often failing to provide primary chromatograms) (18). As a consequence, the ensuing inconsistency in findings has obscured the true extent of biological SPM formation (11) and slowed down progress on SPM-based therapies that could address unresolved inflammation-related diseases, which are estimated to account for more than 50% of global mortality (19). Recently, technical recommendations for analyzing oxylipins by LC-MS by the International Lipidomics Society have established a common quality and reporting standard (14). Here, we demonstrate how to apply these standards and—in doing so—provide a comprehensive study of SPM formation in vitro*,* across the commonly applied human primary cell models, and in vivo in mice.

## Material and Methods

### Chemicals

Solvents (water, methanol, ethanol, methyl formate, *n*-hexane) and acetic acid were obtained in MS grade from Thermo Fisher Scientific Inc. Authentic protiated external standards (ES) and deuterated internal standards (IS) were purchased from Cayman Chemical Company (as listed in Supplementary Table S1.

### Analytical instruments

Data acquisition was realized on a Waters Acquity UHPLC coupled to a Sciex QTrap 5500 triple-quadrupole mass spectrometer using a Turbo V ion source interface controlled by AB Sciex ANALYST Version 1.6.3 (2015; build 1569).

### Chromatography

Chromatographic separation was achieved on a Waters Acquity TM UPLC BEH C18 stationary phase (2.1 mm × 100 mm, 1.7 μm) equipped with a pre-column of identical material and heated to 50°C. Analytes were eluted at a flow rate of 0.3 ml/min by a high-pressure gradient consisting of the solvents A (water:methanol:acetic acid; 89.995:9.995:0.01; *v*/*v*) and B (methanol:acetic acid; 99.99:0.01; *v*/*v*). Analytes were eluted using a linear gradient [0–12.5 min (35.6%–84.4% B)] followed by an isocratic plateau [12.51–15.5 min (97.8% B)] and re-equilibration [15.51–17 min (35.6% B)].

### Mass spectrometry

Analytes were detected by triple-quadrupole MS/MS operated in negative mode with scheduled multiple reaction monitoring (sMRM). Ion spray voltage (−4000 V), temperature (500°C), and flow of collision gas (medium), curtain gas (35 psi), as well as ion source gases 1 and 2 (40 psi, each) were set globally. Analyte-dependent parameters (i.e., *m*/*z* of the precursor (Q1) and product ion (Q3), the declustering potential (DP), entrance potential (EP), collision energy (CE), and collision cell exit potential) were optimized by infusion mode (7 μl/min) using primary standards obtained from Cayman Chemical Company as summarized in Supplementary Table S1. Multiple reaction monitoring (MRM) was scheduled with a target scan time of 200 ms, an MRM pause time of 5 ms, and no additional settling time. The sMRM detection windows spanned approximately 60 s, depending on the analyte. MS/MS spectra were acquired by information-dependent acquisition of enhanced product ion (EPI) scans set to a threshold of 5000 cps and an exclusion window of 4 s (max. 3 occurrences).

### Sample preparation

Validation samples and biological material were subjected to identical sample preparation procedures. Biological samples (one volume) were transferred to fresh glass tubes containing two volumes (2 ml) of ice-cold methanol to quench enzymatic LM formation. A methanolic dilution (10 μl) of stable-isotope-labeled (SIL) internal standards (IS) consisting of *d*_8_-5*S*-HETE, *d*_4_-LTB_4_, *d*_5_-LXA_4_, *d*_5_-RvD2, *d*_4_-PGE_2_ (2 pmol each), and *d*_8_-AA (100 pmol) were added. Samples were vortexed and stored at −20°C for 60 min to facilitate protein precipitation. After centrifugation (1200 × *g*; 4°C; 10 min), the supernatant was transferred to fresh glass tubes containing 9 ml of acidified H_2_O (Milli-Q adjusted to pH 3.5 by PBS/HCl).

LM were extracted by solid phase extraction (SPE). SPE columns (Sep-Pak Vac 6cc 500 mg C18; Waters) were conditioned (6 ml methanol), equilibrated (2 ml H_2_O), loaded (12 ml sample), and washed (6 ml H_2_O followed by 6 ml of *n*-hexane). The LM fraction was eluted with 6 ml methyl formate and samples were evaporated under a gentle nitrogen stream at 37°C until complete dryness (TurboVap LV by Biotage; Uppsala, Sweden). The residue was resuspended in 100 μl aqueous methanol (50:50, *v*/*v*) and stored at −20°C until analysis by UHPLC-MS/MS.

### Generation of validation samples

Stock solutions of LM standards were stored at −80°C in the original shipping diluent (ranging from 0.05 to 5 mg/ml) or, when shipped as solids, in ethanol (1 mg/ml). Working solutions were prepared weekly in aqueous methanol (50:50, *v*/*v*). Calibrators were prepared on three different days by serial dilution to yield 10–20,000 pg/ml in logarithmic distribution (10 pg–33.33 pg – 100 pg – …). Quality control samples (QC) were prepared by separate dilutions. Calibrators (1 ml) and QC (1 ml) were subjected to sample preparation as described above.

Accuracy was expressed as the recovered amount relative to the nominal amount (%). Precision was expressed as the relative standard deviation (RSD) of the recovered amounts (%). The resolution *R* of critical pairs was calculated according to the European Pharmacopoeia (20) based on the retention times (Rt) and the full width at half maximum (FWHM) of earlier eluting peak_1_ and later eluting peak_2_.

$$R=1.18\times \frac{{\text{Rt}}_{2}-{\text{Rt}}_{1}}{{\text{FWHM}}_{2}+{\text{FWHM}}_{1}}$$

Recovery was calculated by comparing the peak area of standards spiked before and after SPE. The signal-to-noise ratio (S/N) was calculated manually based on height according to Ph. Eur. 2.02.46./USP 621 (20) and compared to three software-based calculation methods (“peak to peak,” “standard deviation,” and “relative noise”) by SCIEX OS-Q v4.0.

### Generation of in vitro samples

Blood cells were isolated from leukocyte concentrates derived from freshly withdrawn blood (16 I.E. heparin/ml blood) of healthy adult male and female volunteers (18–65 years; with written informed consent; without ancestral, racial or ethnic information) provided by the Institute of Transfusion Medicine at the University Hospital Jena. Protocols for experiments with human blood cells were approved by the Ethics Commission of the Friedrich Schiller University Jena (approval number 5050-01/17). All methods were performed in accordance with the relevant guidelines and regulations.

Platelet-enriched plasma, peripheral blood mononuclear cells (PBMC) and polymorphonuclear leukocytes (PMNL) were separated by density gradient centrifugation using Histopaque (Merck/Sigma-Aldrich, St. Louis, MO, USA; Cat. No. 10771) after sedimentation of erythrocytes by dextran according to previously published procedures (21).

Platelets were purified from the platelet-enriched plasma obtained after sequential centrifugation (2100 × *g*; 15 min; room temperature), discarding the supernatant, and three washing steps [once in PBS (pH 5.9) and twice in a 1:1 (*v*/*v*) mixture of PBS (pH 5.9) and aqueous NaCl solution (0.9% *m*/*v*)].

PBMC were allowed to adhere to Greiner Bio-one flasks (Frickenhausen) in PBS pH 7.4 containing CaCl_2_ (1 mmol/L) and MgCl_2_ (0.5 mmol/L). Non-adherent cells were discarded after 1 h at 37°C and 5% CO_2_.

Monocyte-derived macrophages (MDM) were obtained as described before (22). Briefly, the isolated monocytes from the abovementioned PBMC fraction were differentiated for 6 days towards M0 in RPMI medium (10% fetal calf serum, 2 mmol/L l-glutamine, 100 U/L penicillin, and 100 μg/ml streptomycin) supplemented with granulocyte-macrophage colony-stimulating factor (GM-CSF, 20 ng/ml) or macrophage colony-stimulating factor (M-CSF, 20 ng/ml). M0_GM-CSF_ were polarized for 24 h towards classically activated macrophages (M1) in the presence of LPS (100 ng/ml) and 20 ng/ml IFN-γ. M0_M-CSF_ were polarized for 48 h towards alternatively activated macrophages (M2a) in the presence of IL-4 (20 ng/ml).

Bacterial exotoxins were obtained from *Staphylococcus aureus*-conditioned medium (SACM) according to previously published procedures (23). Thus, the methicillin-susceptible *S. aureus* strain 6850 was cultivated on Columbia blood agar plates at 37°C. A single colony was transferred into BHI medium (50 ml) and shaken for 24 h. After dilution to a defined optical density (OD_600 nm_ = 0.05), incubation for another 24 h, and centrifugation (3350 × *g*; 5 min; room temperature), the supernatant was sterile-filtered through a Millex-GP filter unit (0.22 μm; Millipore) and stored at 4°C.

PBMC (2 × 10^6^ cells), M1-/M2a-MDM (2 × 10^6^ cells), PMNL (10^7^ cells), platelets (2.5 × 10^8^ cells), or co-cultures of PMNL (10^7^ cells) with platelets (2.5 × 10^8^ cells) were incubated in 1 ml PBS containing CaCl_2_ (1 mmol/L). Cells were incubated for 90 min at 37°C and 5% CO_2_ (*i*) without stimulus; (*ii*) with Ca^2+^-ionophore A23187 (0.5 μmol/L); or (*iii*) with SACM (1%).

Peripheral blood was provided by the Institute of Transfusion Medicine at the University Hospital Jena from healthy volunteers as described above. Whole blood (1 ml) containing the anticoagulant Li-heparin was incubated for 90 min at 37°C and 5% CO_2_ (i) without stimulus; (ii) with A21287 (30 μmol/L); or (iii) with SACM (3%).

### Generation of in vivo samples

Male and female C57BL/6JRj mice (3 months of age) were housed at the Bioinstrumentezentrum (BIZ) in a specific pathogen-free environment with a 12 h light/dark cycle and ad libitum access to food and water. Brain, spleen, lung, liver, and colon were collected *postmortem* after CO_2_ inhalation, washed in PBS, and stored at −80°C. Tissues were homogenized in PBS using a FastPrep-24 5G bead beating homogenizer (M.P. Biomedicals). Homogenates were combined with an equal volume methanol to ensure LM extraction. After centrifugation (4°C; 10 min; 21,100 ×*g*), an aliquot of the supernatant corresponding to 30 mg of homogenized organ was transferred to fresh tubes containing two volumes of methanol and spiked with IS as described for validation samples. Storage, SPE, and UHPLC-MS/MS analysis of in vivo samples was identical to that of validation samples.

### Peak integration

Chromatograms were integrated in ANALYST Version 1.6.3 (2015; build 1,569) by AB Sciex (Framingham MA, USA). LM were quantified by linear regression of area ratios (external to internal standard) weighted for heteroscedasticity (1/*c*). Representative chromatograms are shown without peak shape manipulation by smoothing or bunching.

### Complementary chiral and achiral LC-MS analysis

Chiral LC-MS/MS (24) and achiral LC-HRAM were used to confirm the stereochemistry and identity of selected analytes. Detailed chromatographic and mass spectrometric conditions together with representative chromatograms for the complementary chiral and achiral methods are provided in Supplementary Fig. S1 and S2, respectively.

### Statistical analysis

Results are expressed as means ± SEM of *n* biological replicates. The in vitro (n = 4), in vivo (n = 4), and ex vivo (n = 6) sample sizes were selected in line with commonly used group sizes in oxylipin profiling studies and considered sufficient to detect biologically meaningful differences in oxylipin formation under the experimental conditions applied. Statistical significance was calculated by lognormal repeated measures one-way ANOVA with single pooled variance using GraphPad Prism 10 (version 10.6.1). Tukey’s test was used for comparing multiple groups to each other (in vitro stimulus comparison; 6 degrees of freedom). Dunnett’s test was used for comparing multiple groups to a single control group (in vivo organ comparison in reference to spleen, the highest yielding organ; 12 degrees of freedom). ∗*P* < 0.05; ∗∗*P* < 0.01; ∗∗∗*P* < 0.001.

## Results

### Method validation

#### Target analytes and oxylipin nomenclature

The presented method for oxylipin analysis targets a total of 72 analytes, consisting of 19 SPM – including lipoxins (LX), resolvins (Rv), maresins (MaR), and protectins (PD) – as well as 18 monohydroxylated precursors and pathway markers, 9 additional di-/trihydroxylated oxylipins, 4 PUFA, and 22 prostanoids (full list in Supplementary Table S1).

Stereoisomers of oxylipins encompass geometric isomers, other diastereomers, and enantiomers. In the case of reversed-phase LC (RPLC), separation is generally determined by lipophilicity, which in turn is affected by double bond configuration (e.g. *E* vs. *Z*) and partial differences in at least one of multiple asymmetric centers (e.g. *R,R* vs. *S,R*), but not by enantiomerism (e.g. *R,R* vs. *S,S*). Thus, only diastereomers can be separated by RPLC in absence of a chiral selector. The epithet “and enantiomer” is therefore implied in achiral methods, and their longstanding application to chiral metabolites relies on enantioselective formation routes. In the case of some SPM, however, seemingly uniform peaks have been shown by achiral-chiral 2D separation to consist of multiple stereoisomers (25). Hence, we use trivial names that imply stereochemistry only for chromatograms of enantio-pure commercial standards (e.g., LXA_4_ when injecting 5*S*,6*R*,7*E*,9*E*,11*Z*,13*E*,15*S*-triHETE), while emphasizing potential co-eluates in the context of biological samples (e.g., LXA_4_ and related 5,6,15-triHETE).

#### Separation by LC-MS/MS

**Fig. 1:**
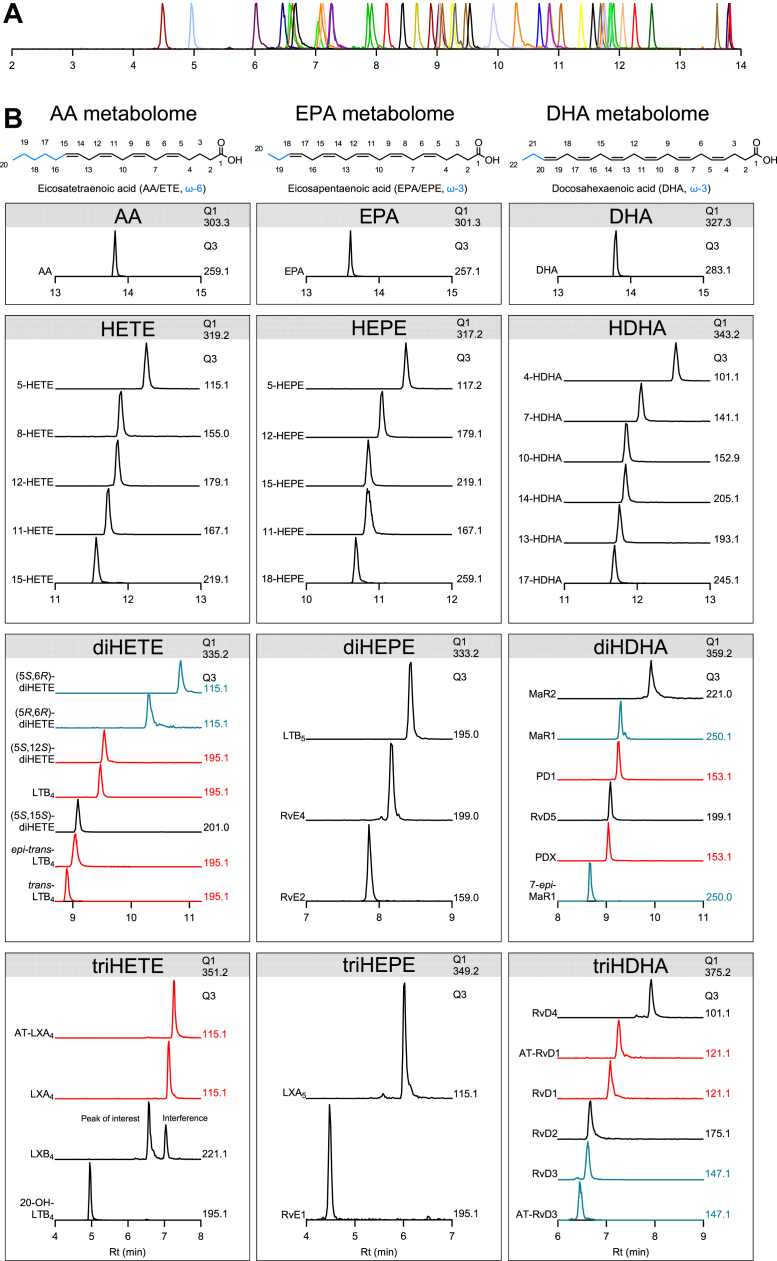
Chromatographic separation of oxylipins analyzed by UHPLC-MS/MS. A: Primary unsmoothed chromatograms (MRM) of all LOX product reference standards. B: Primary chromatograms (XIC) of reference standards grouped by isomer sets (identical Q1) with precursor *m*/*z* shown in the top right of each box. Critical isomers with identical quantifier Q3 product ion are highlighted in color.

Specific and sensitive bioanalysis of low-abundance SPM among higher-abundance oxylipins, including isomers and precursors that exhibit similar hydrophobicity and fragmentation behavior, required both LC and MS/MS, as shown by partially overlapping LOX product peaks (Fig. 1A). Hydroxylation of arachidonic acid (AA; chemically an eicosatetraenoic acid [ETE]), eicosapentaenoic acid (EPA), and docosahexaenoic acid (DHA) yields monohydroxylated isomers (HETE, HEPE, HDHA, respectively), as well as their di- and trihydroxylated metabolites (di-/triHETE, di-/triHEPE, di-/triHDHA). As shown in Figure 1B, these isomer sets contain constitutional isomers and stereoisomers that share a common precursor ion *m*/*z* (Q1) but vary in position/configuration of hydroxy groups/double bonds, with classical SPM being found in diHEPE, diHDHA, triHETE, triHEPE, and triHDHA. By optimizing the collision energy to favor selective Q1/Q3 transitions resulting from α-cleavage next to characteristic hydroxy groups, constitutional isomers were well-separated by MS/MS (inter-analyte crosstalk < 20%). Diastereomers, on the other hand, required LC separation.

#### Specificity

Specificity is the ability to differentiate the analyte from related substances such as isomers (26). Table 1 shows critical diastereomer pairs with the same Q1/Q3 transition. These diastereomers differed in at least a single stereogenic element, such as aspirin-triggered (AT) epimers of LXA_4_, RvD1, and RvD3 that owe their name to formation by acetylated COX (27). All addressed SPM/AT-SPM pairs were resolved by at least *R* ≥ 1.3. Critical diastereomer pairs differing in multiple stereoelements included PD1 and PDX, which have been mistaken in the past (28) but were well-resolved here (*R* = 3.1). However, separation did not correlate with the number of stereogenic differences, as demonstrated by MaR1 and 7-epi-MaR1, the pair with the highest resolution (*R* = 7.3). The classical eicosanoid LTB_4_, on the other hand, partially co-eluted with 5*S*,12*S*-diHETE (*R*_S_ > 0.7), even though this diastereomer pair differs in three stereogenic elements and is often quantified by less specific instruments such as HPLC-UV (29).

**Table 1:** Chromatographic separation of critical diastereomer pairs derived from LOX

Diastereomers	Trivial Name	Structural Differences	Rt (min)	FWHM (min)	Chromatogram	*R*
5,12-diHETE	LTB_4_		9.47	0.06		0.7
	5*S*,12*S*-diHETE		9.54	0.06		
	*trans*-LTB_4_		8.90	0.05		1.4
	*epi-trans*-LTB_4_		9.05	0.08		
5,6-diHETE	5*R*,6*R*-diHETE		10.31	0.06		4.9
	5*S*,6*R*-diHETE		10.85	0.07		
10,17-diHDHA	PDX		9.05	0.04		3.1
	PD1		9.26	0.04		
7,14-diHDHA	7-*epi*-MaR1		8.67	0.05		7.3
	MaR1		9.29	0.05		
5,6,15-triHETE	LXA_4_		7.12	0.07		1.4
	AT-LXA_4_		7.27	0.06		
7,8,17-triHDHA	RvD1		7.08	0.06		1.6
	AT-RvD1		7.26	0.07		
4,11,17-triHDHA	AT-RvD3		6.46	0.07		1.3
	RvD3		6.62	0.07		

#### Selectivity

Selectivity, that is, the ability to differentiate the analyte in presence of unspecific matrix interference (26), was assessed by a two-pronged approach: Firstly, LLOQ-spiked surrogate matrix was compared to oxylipin-free surrogate matrix (PBS containing 1 mmol/L CaCl_2_), i.e. the stimulation medium of in vitro samples. Secondly, the absence of signals was confirmed in unstimulated biological matrix (including M2a-MDM and PMNL/platelet co-incubations capable of SPM formation upon stimulation).

**Fig. 2:**
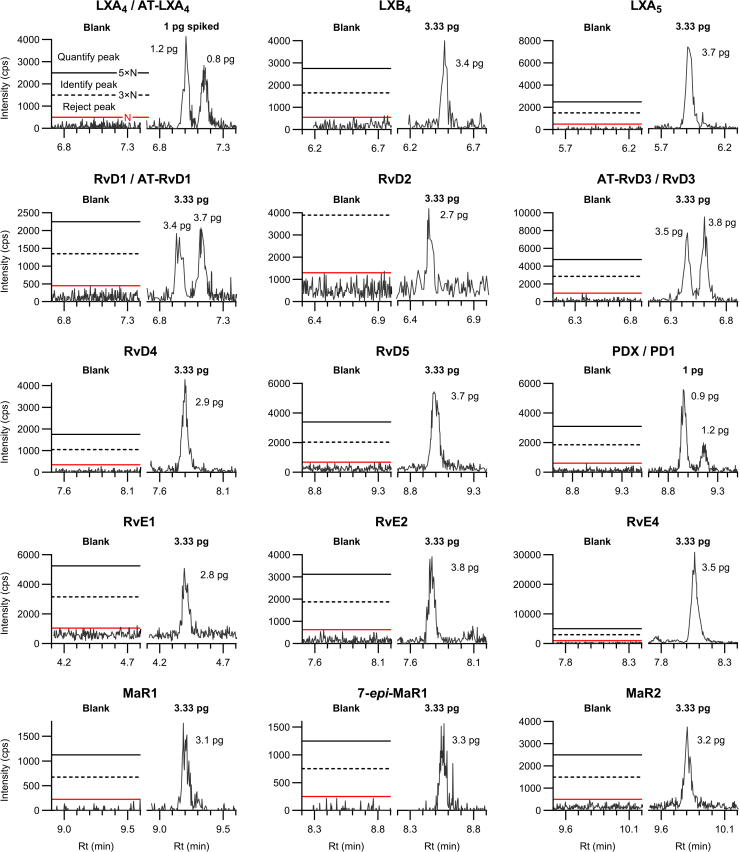
Selectivity and sensitivity of SPM determination. Primary unsmoothed chromatograms of blank PBS matrix and blank PBS matrix spiked with reference standards near the LLOQ. Samples were extracted by SPE and analyzed by UHPLC-MS/MS. Spiked (headers) and recovered amounts (non-bold text) are given as pg on column.

No analytes were detected after SPE of oxylipin-free matrix (PBS). In contrast, SPE of matrix spiked with SPM translating to 1–3.3 pg on-column yielded well-distinguishable peaks that passed both automatic and manual identification (Fig. 2). Similarly, unstimulated in vitro matrices yielded no quantifiable SPM prior to stimulation, as shown for M2a-MDM in Supplementary Fig. S3. Manual integration at the corresponding Rt yielded areas consistently below the peak area of LLOQ samples (<20% for ES; < 5% for IS). The method was therefore deemed selective for the purpose of SPM quantification.

#### Calibration curves

The relationship between the nominal analyte amount and peak areas was evaluated by spiking surrogate matrix (PBS) with 9 different concentrations of external standards (10–20,000 pg/ml; log-linearly distributed) and fixed amounts of internal standards (2 pmol/ml for all deuterated standards except for AA-*d*_8_, which required 100 pmol/ml due to lower ionization efficiency). Extraction, evaporation, and reconstitution in 100 μl reconstitution medium resulted in nominal amounts (on column) of 1–2,000 pg for ES and 66–76 pg for all IS except AA-*d*_8_ (3,125 pg). A robust and reproducible linear relationship was found for SPM after weighting 1/*x* (*R*^2^ ≥ 0.99). The slope, *y*-intercept, *r* value, amounts on column (nominal and found), and resulting accuracy of a typical calibration curve are shown for each SPM in the Supplementary Fig. S4.

#### Sensitivity and range

The range-spanning LLOQ and ULOQ for oxylipins were determined based on S/N, accuracy, and precision. For SPM, setting a conservative ULOQ of 100 pg on column increased accuracy at lower amounts. Based on accuracy, precision, and S/N of calibrators and QC, the LOD ranged between 0.33-3.33 pg and the LLOQ between 1.00-10.00 pg, depending on the type of SPM (Table 2). The highest sensitivities were obtained for LXA_4_, PDX, and PD1 (LOD = 0.33 pg; LLOQ = 1.00 pg). The lowest sensitivities were found for RvD1 (whose S/N fell below 5 at 3.33 pg when selecting a broad noise region) and RvD2 (for which an overall higher baseline undulation close to 1000 cps was observed). Accuracy (100 ± 20%) and precision (RSD < 20%) at 3.33 pg was within recommendation for most SPM (including RvD1 and RvD2) except for a slight deviation in accuracy by RvE4 (124%) and in precision by RvD5 (21% RSD). This degree of imprecision must be considered when interpreting the relevance of SPM analysis near LLOQ concentrations from biological samples. Of note, certain other oxylipins showed lower accuracy and precision near the LLOQ, such as the substrate AA (139%), the monohydroxylated 18-HEPE (65%), and the dihydroxylated *epi-trans*-LTB_4_ (125%). This margin was considered acceptable, since these oxylipins commonly arise in biological samples at higher concentration as shown in the Supplementary Table S2.

**Table 2:** LOD and LLOQ (on column) of SPM as determined from S/N, accuracy, and precision, and S/N

Analyte	Limits (pg)		S/N ± SD			Accuracy ± precision (%) of Calibration samples					Accuracy ± precision (%) of QC		
	LOD	LLOQ	1 pg	3.33 pg	10 pg	1 pg	3.33 pg	10 pg	333.33 pg	500 pg	1 pg	3.33 pg	10 pg
LXA_4_	0.33	1	6 ± 2	16 ± 6	29 ± 15	89 ± 17	107 ± 12	110 ± 7	99 ± 5	97 ± 4	100 ± 10	103 ± 6	102 ± 10
AT-LXA_4_	1	3.33	7 ± 3	15 ± 5	21 ± 5	85 ± 10	98 ± 4	113 ± 8	99 ± 8	99 ± 5	122 ± 17	100 ± 3	103 ± 9
LXB_4_	1	3.33	3 ± 1	5 ± 1	13 ± 5	/ /	95 ± 19	118 ± 18	103 ± 6	101 ± 4	/ /	104 ± 20	116 ± 12
LXA_5_	1	3.33	9 ± 3	22 ± 4	59 ± 17	67 ± 17	105 ± 7	126 ± 12	104 ± 7	100 ± 3	96 ± 10	108 ± 17	107 ± 11
RvD1	3.33	3.33	1 ± 0	3 ± 1	9 ± 4	/ /	88 ± 13	101 ± 11	92 ± 9	94 ± 6	/ /	90 ± 13	98 ± 8
AT-RvD1	1	3.33	3 ± 2	7 ± 1	19 ± 7	/ /	94 ± 15	100 ± 7	94 ± 11	97 ± 4	205 ± 38	109 ± 17	90 ± 8
RvD2	3.33	10	1 ± 0	3 ± 1	9 ± 1	/ /	88 ± 15	111 ± 16	99 ± 10	98 ± 5	/ /	169 ± 5	112 ± 10
RvD3	1	3.33	6 ± 1	11 ± 4	38 ± 15	89 ± 20	97 ± 14	120 ± 12	101 ± 10	101 ± 3	126 ± 15	111 ± 11	108 ± 10
AT-RvD3	1	3.33	5 ± 2	10 ± 4	33 ± 17	87 ± 18	102 ± 9	114 ± 14	102 ± 11	101 ± 4	105 ± 10	101 ± 9	112 ± 12
RvD4	1	3.33	6 ± 1	10 ± 3	11 ± 4	87 ± 13	105 ± 11	105 ± 12	97 ± 11	99 ± 4	108 ± 18	96 ± 9	97 ± 9
RvD5	1	3.33	5 ± 1	11 ± 3	29 ± 5	84 ± 9	114 ± 21	113 ± 11	100 ± 6	101 ± 4	94 ± 11	106 ± 17	98 ± 7
PDX	0.33	1	11 ± 3	30 ± 8	70 ± 11	82 ± 7	105 ± 7	116 ± 6	103 ± 7	104 ± 4	104 ± 10	101 ± 6	105 ± 6
PD1	0.33	1	5 ± 2	12 ± 2	28 ± 6	97 ± 12	102 ± 5	107 ± 9	97 ± 8	98 ± 3	114 ± 9	90 ± 10	98 ± 9
RvE1	1	3.33	2 ± 0	7 ± 1	17 ± 3	69 ± 22	114 ± 16	118 ± 16	101 ± 7	99 ± 5	67 ± 14	98 ± 12	102 ± 15
RvE2	1	3.33	4 ± 1	9 ± 2	21 ± 5	64 ± 19	112 ± 20	113 ± 14	102 ± 8	99 ± 4	60 ± 33	105 ± 17	109 ± 9
RvE4	1	3.33	11 ± 2	34 ± 8	106 ± 18	32 ± 16	124 ± 17	143 ± 11	119 ± 8	112 ± 4	86 ± 11	105 ± 14	112 ± 7
MaR1	1	3.33	9 ± 6	11 ± 4	34 ± 8	92 ± 21	98 ± 8	111 ± 13	96 ± 8	99 ± 3	92 ± 22	99 ± 12	107 ± 9
MaR2	1	3.33	4 ± 0	9 ± 2	21 ± 5	/ /	99 ± 16	99 ± 13	89 ± 8	98 ± 6	/ /	113 ± 13	99 ± 10
7-epi-MaR1	1	3.33	5 ± 2	15 ± 9	43 ± 24	79 ± 19	107 ± 14	115 ± 9	95 ± 7	98 ± 4	74 ± 14	94 ± 10	104 ± 8

/: peak criteria not fulfilled.

#### Signal-to-noise

The S/N is a useful criterion for peak selection, but thresholds and calculation procedures vary depending on the regulatory framework (30, 31, 32). For oxylipins, technical recommendations have settled on manually calculated S/N ≥ 3 (identification) and S/N ≥ 5 (quantification), with S corresponding to the target peak height (*H*) and N to the baseline undulation (*h*), allowing N to be determined from the same chromatogram (the region before or after the target peak) or from a suitable blank (the same region as the target peak) (14). Software or alternative approaches are allowed, provided a similar outcome has been documented.

Our primary approach to determine S/N was therefore manual calculation based on N of the surrogate matrix blank. However, manual calculation based on N of the same chromatogram and three software-based algorithms (“peak-to-peak,” “standard deviation,” and “relative noise”) were also evaluated in anticipation of high sample throughput (rendering manual calculation impractical) and biological chromatograms containing additional stereoisomer peaks (restricting the available noise region).

As shown for LXA_4_ in Supplementary Fig. S5A, B, manual calculation of S/N based on the peak-adjacent region (before vs. after peak of interest) was comparable to S/N based on the putative peak region in the blank (*cf.* Table 2). Automatic calculation via the “peak-to-peak” algorithm provided similar S/N values, proving a useful screening tool for identifying peaks that warrant manual S/N calculation (Supplementary Fig. S5C). On the other hand, automatic calculation via “standard deviation” yielded S/N that were well above manual calculation and did not match users’ visual estimation (Supplementary Fig. S5D). This algorithm was therefore not pursued further. “Relative noise” yielded intermediate values but had the advantage of not requiring the user to define a noise region, thereby increasing S/N objectivity (Supplementary Fig. S5E). “Relative noise” was therefore used as a screening tool for biological samples where closely eluting peaks impeded manual S/N calculation based on the peak-adjacent region. However, the threshold was set to S/N_rel_ > 10 to compensate for approximately 2-fold higher values compared to manual calculation, especially at lower concentrations (Supplementary Fig. S5A).

#### Peak selection criteria

Initial integration was performed automatically (by fixed Rt, MQ3, or AutoPeak) and followed by manual confirmation/correction. Signals were identified as peaks when meeting the following criteria.

(1) Visual identification of a distinguishable peak in the corresponding MRM trace was confirmed using an authentic standard.
(2) Rt matching ± 0.1 min the external standard acquired within the same batch.
(3) S/N > 3 (for identification) and S/N > 5 (for quantification) by manual S/N calculation based on the same chromatogram (excluding neighboring peaks).
(4) Datapoints > 10 across base.
(5) Confirmation of oxylipins discovered for the first time in a given matrix by matching the MS/MS fragmentation pattern obtained by an EPI scan to that of authentic standards.

#### Carry-over

Carry-over, i.e., the unintended transfer of analyte from one analytical run to the next, was investigated by extracting and measuring a ULOQ-spiked sample followed by a blank. While peaks for linoleic acid-derived 9- and 13-HODE and lipophilic PUFA (AA, EPA, DHA) were found in the following sample, carry-over of SPM was below 20% (5% for internal standards) of the respective areas of the LLOQ.

#### Recovery

Recovery, i.e., the proportion of analyte that was available for detection after sample preparation, was investigated by comparing peak areas from a standard solution (10–33 pg on column) that was either injected directly or first extracted by SPE and reconstituted in the same volume. Using fresh SPE cartridges, the average loss in peak area across SPM was 25 ± 8%, with analyte-specific recoveries ranging from 64% to 85%, as shown in the Supplementary Fig. S6. Normalization to the IS partially compensated for incomplete recovery, resulting in accurate back-calculated concentrations. However, re-use of SPE cartridges negatively impacted recovery, leading to a higher average loss and higher variability (46 ± 12%) by the 20^th^ usage cycle. While IS-normalized quantification compensated for accuracy, the reduction in peak intensity by half negatively affected sensitivity. Columns were therefore only used once for biological samples.

#### Matrix effect

Matrix effects measure the alteration of the analyte response caused by interfering matrix components (26). Comparison of blank-matrix spiked after SPE with matrix-free standard solutions yielded no significant matrix effects on peak areas (100 ± 20%) compared to the surrogate matrix PBS (used for in vitro samples). However, detergent-rich homogenization buffers (e.g., NP-40) caused a noticeable shift in Rt (approx. 0.1 min), as shown for PDX in the Supplementary Fig. S7. In vivo samples were therefore homogenized in detergent-free methanol.

### Analysis of oxylipins formed in cells in vitro and ex vivo

The validated method for oxylipin/SPM analysis was applied to human PBMC, M1-MDM, M2a-MDM, PMNL, platelets, co-incubations of PMNL/platelets, and whole blood, with the aim of assessing the SPM profiles in these commonly employed primary blood cells. The incubations were conducted in 1 ml of PBS containing 1 mmol/L CaCl_2_ under three conditions: (*i*) unstimulated, (*ii*) stimulated with A23187 (0.5 μM), and (*iii*) stimulated with exotoxins from *S. aureus* (1% SACM). Higher concentrations of A23187 (30 μM) and SACM (3%) were used to stimulate whole blood ex vivo. Oxylipin formation was quenched after 90 min in 2 volumes of ice-cold methanol, followed by extraction and analysis as described for validation samples.

**Fig. 3:**
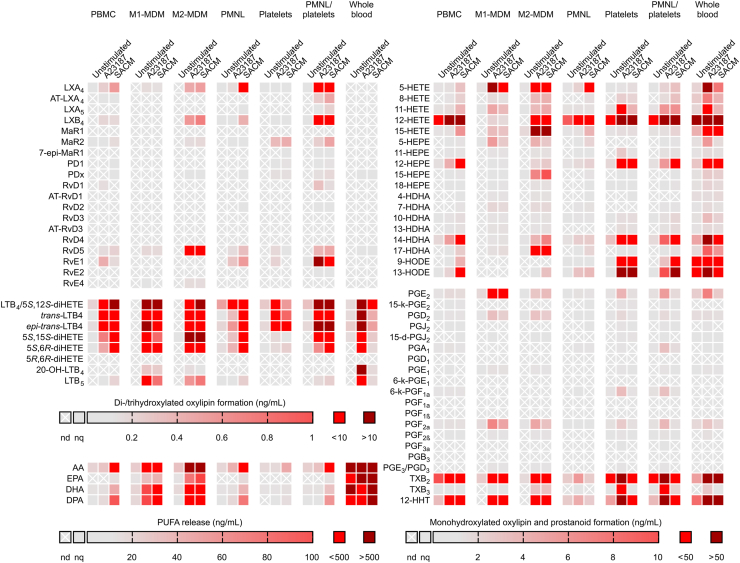
SPM and overall oxylipin profile of primary human blood cells. PBMC (2 × 10^6^ cells), M1-/M2a-MDM (2 × 10^6^ cells), PMNL (1 × 10^7^ cells), platelets (2.5 × 10^8^ cells), or co-cultures of PMNL (1 × 10^7^ cells) with platelets (2.5 × 10^8^ cells) were left unstimulated, stimulated with A23187 (2.5 μmol/L), or stimulated with SACM (1%) for 90 min (n = 4). Whole blood (1 ml) was stimulated with A23187 (30 μmol/L) or stimulated with SACM (3%) for 90 min (n = 6). Oxylipins were extracted by SPE and determined by UHPLC-MS/MS. Results are expressed as means with different color coding for di-/trihydroxylated products (top left), monohydroxylated products and prostanoids (right), and non-hydroxylated precursors (bottom left).

SPM and overall oxylipin formation in response to A23187 and SACM varied according to the cell type and stimulus (Supplementary Fig. S8). Note that SPM in unstimulated cells were below the LLOQ, thus validating the selectivity of the bioanalytical method relative to each of the investigated matrices (Fig. 3).

#### Single cell-type models

In stimulated PBMC (that express 5-LOX, 12-LOX and COX-1 (33)), the most prominent oxylipin was 12-HETE, likely due to inevitable platelet contamination during cell isolation (Fig. 3). COX products dominated (especially TXB_2_ ≈ 12-HHT >> PGE_2_ > PGF_2α_) over 5-LOX products (LTB_4_ > *epi-trans*-LTB_4_ > *trans*-LTB_4_ > 5-HETE). While A23187 skewed oxylipin profiles towards COX products, total oxylipin formation was significantly higher upon stimulation with SACM (Supplementary Table S2). 15-LOX products (mainly 15-HETE) were minute. Only trace signals were observed for SPM (mainly 5,6,15-triHETE matching the Rt of LXA_4_).

M1-MDM produced high amounts of COX products (especially PGE_2_), due to COX-2/mPGES-1 induction during classical polarization (33). 5-LOX activity was also increased (5-HETE > LTB_4_ > *epi-trans*-LTB_4_ > *trans*-LTB_4_), while 12-/15-LOX activity (and resulting SPM formation) was lower than in PBMC (Fig. 3). Unlike PBMC, M1-MDM responded more strongly to A23187 than to SACM (when judged by total oxylipin formation), although differences within individual oxylipins were mostly non-significant.

M2a-MDM (expressing 5-LOX and especially 15-LOX-1 (33)) produced more TXB_2_ and 12-HHT, but significantly less PGE_2_ than M1-MDM (Fig. 3). While 5-LOX activity was slightly decreased versus M1-MDM, 15-LOX activity was significantly higher and indeed highest among all studied cell types, especially in response to SACM. Major products included 15-HETE (94.1 ng) > 17-HDHA (20.3 ng) > 15-HEPE (8.9 ng), but also considerable amounts of dihydroxylated species such as homochiral (i.e. *SS* or *RR*) 5,15-diHETE (12.4 ng) and 7,17-HDHA (1.8 ng), the latter matching the Rt of RvD5. Complementary chiral analysis of RvD5 intermediates (Supplementary Fig. S1A) demonstrated enantio-preferential formation for 7-HDHA and 17-HDHA (10:1 and 32:1, respectively), as well as a single peak matching the reference material for RvD5 (Supplementary Fig. S1B). The ratio of precursor/mono-/dihydroxylated species (100:10:1) was in line with previous reports (13).

PMNL yielded the lowest overall oxylipin formation under the given conditions, with A23187 mainly evoking COX activity (TXB_2_ ≈ 12-HHT > PGE_2_) and SACM evoking substantial 5-LOX activity (5-HETE >> LTB_4_ ≈ 5,15-diHETE > 5,6-diHETE ≈ *epi-trans-*LTB_4_ > *trans*-LTB_4_) (Fig. 3). 15-LOX activity was low. However, considerable amounts of 5,6,15-triHETE were observed, matching the Rt of LXA_4_ (1,8 ng).

Platelets exhibited the highest 12-LOX (12-HETE) and COX activity (12-HHT > TXB_2_ >> PGE_2_ > 6-keto-PGF_1α_ > PGF_2α_) – especially in response to A23187 – due to high 12-LOX and COX-1 expression (Fig. 3). 5-LOX activity was very low (*epi-trans-*LTB_4_ > LTB_4_ ≈ *trans*-LTB_4_ >> 5-HETE) and 15-LOX activity (including SPM formation) was minute. Overall, platelets were more responsive to A23187 than to SACM.

#### Complex cellular models: Cell–cell co-incubations and whole blood

In co-incubations of 5-LOX/15-LOX/COX-1-expressing PMNL and 12-LOX/COX-1-rich platelets, the ratio of 5-/11-/12-/15-HETE after stimulation with A23187 (5:20:700:1) and SACM (2:5:600:1) indicated a clear dominance of 12-LOX (12-HETE), followed by COX (11-HETE), 5-LOX (5-HETE), and very low 15-LOX (15-HETE) activity (Supplementary Table S2). Again, A23187 skewed towards COX activity, while SACM favored the LOX activities. For most analytes, co-incubations produced levels that were comparable to the sum of the respective monocellular incubations. However, formation of trihydroxylated analytes matching LXA_4_ (7.2 ng), LXB_4_ (7.7 ng), and RvE1 (13.0 ng) was 4- to 10-fold higher in co-incubations. Thus, 5-LOX together with 12-LOX activity rather than 15-LOX activity may be required for effective formation of LXs.

Stimulated whole blood produced high oxylipin levels overall, especially in response to A23187, where mainly monohydroxylated derivatives of AA (12-HETE > 5-HETE > 15-HETE > 11-HETE > 8-HETE) and of other PUFA (14-HDHA > 13-HODE > 9-HODE), but also of COX products (12-HHT > TXB_2_ >> PGE_2_) and leukotrienes (LTB_4_ > *trans-*LTB_4_ > *epi-trans-*LTB_4_) were formed. Although this indicated considerable 5-LOX, 12-LOX, and even some 15-LOX activity, no SPM were detectable in stimulated blood.

#### Reporting of specialized pro-resolving mediators

**Fig. 4:**
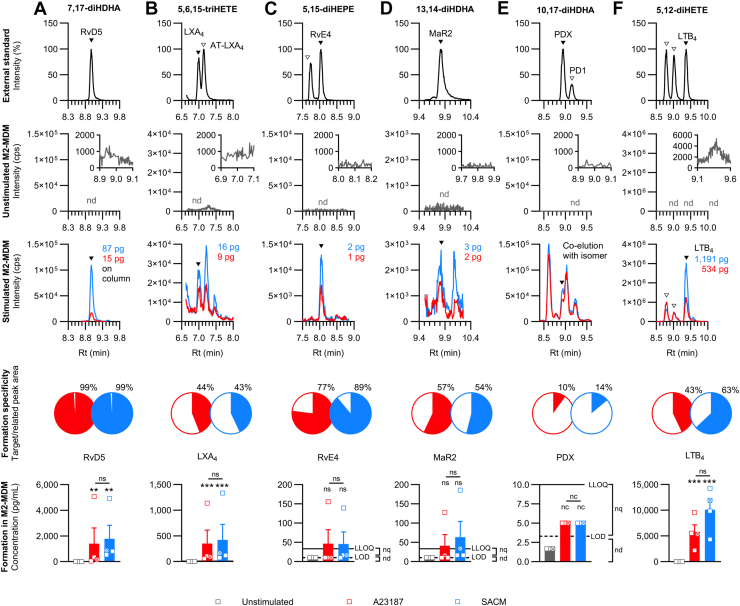
Primary chromatograms and resulting quantitative analysis of SPM obtained in M2-MDM. M2a-MDM (2 × 10^6^ cells/ml) were incubated without stimulus (gray), stimulated with A23187 (2.5 μmol/l), or stimulated with SACM (1%) for 90 min. Oxylipins were extracted by SPE and determined by UHPLC-MS/MS. Chromatograms show a representative donor and indicate the amount found on column. Pie charts show mean semi-quantitative formation specificity (target peak area divided by related peak areas). Bar charts show calculated concentrations (means ± SEM) from each donor (n = 4), indicating the donor whose chromatogram is shown (☒), the limit of detection (LOD; dashed line) and the lower limit of quantification (LLOQ; solid line). Statistical analysis was performed by lognormal RM one-way ANOVA with Tukey’s multiple comparisons test and single pooled variance. ∗*P* < 0.05; ∗∗*P* < 0.01; ∗∗∗*P* < 0.001; nc, *P* not calculated (no variance); nd, non-detectable; nq, non-quantifiable. A: RvD5, (B) (AT-)LXA_4_, (C) RvE4, (D) MaR2, (E) PDX/PD1, and (F) LTB_4_.

**Fig. 5:**
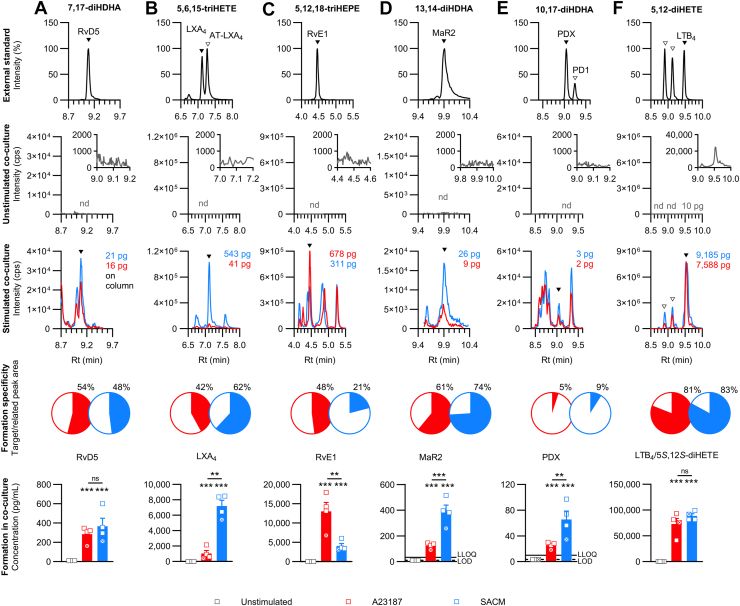
Primary chromatograms and resulting quantitative analysis of SPM obtained in PMNL/platelet co-incubations. Co-incubations of PMNL (1 × 10^7^ cells) with platelets (2.5 × 10^8^ cells) were incubated without stimulus (gray) or stimulated with A23187 (2.5 μmol/L) or stimulated with SACM (1%) for 90 min. Oxylipins were extracted by SPE and determined by UHPLC-MS/MS. Chromatograms show a representative donor and indicate the amount found on column. Pie charts show mean semi-quantitative formation specificity (target peak area divided by related peak areas). Bar charts show calculated concentrations (means ± SEM) from each donor (n = 4), indicating the donor whose chromatogram is shown (☒), the limit of detection (LOD; dashed line) and the lower limit of quantification (LLOQ; solid line). Statistical analysis was performed by lognormal RM one-way ANOVA with Tukey’s multiple comparisons test and single pooled variance. ∗*P* < 0.05; ∗∗*P* < 0.01; ∗∗∗*P* < 0.001; nd, non-detectable; nq, non-quantifiable. (A) RvD5, (B) (AT-)LXA_4_, (C) RvE1, (D) MaR2, (E) PDX/PD1, and (F) LTB_4_.

Our data reveal (*i*) absence of relevant SPM levels in stimulated whole blood and unstimulated cell models under the present experimental and analytical conditions; (*ii*) only trace SPM formation in stimulated PBMC, M1-MDM, and monocellular PMNL or platelet incubations; but (*iii*) significant SPM formation in stimulated M2a-MDM (i.e., RvD5) and PMNL/platelet co-incubations (i.e., LXA_4_, LXB_4_, and RvE1). Representative, unsmoothed chromatograms of these SPM are provided for M2a-MDM (Fig. 4) and PMNL/platelet coincubations (Fig. 5) together with the amounts found on column and the resulting concentrations calculated for each sample.

As shown in Figure 4A, stimulation of M2a-MDM with A23187 (red) or bacterial exotoxins (blue) induced a singular peak that matched the retention time of synthetic RvD5, i.e. 7*S*,17*S*,4*Z*,8*E*,10*Z*,13*Z*,15*E*,19*Z*-diHDHA (black). For this MS/MS transition, the precursor ion (Q1) was largely unspecific and selected for any diHDHA (or even unrelated isobars). However, since conditions in Q2 were optimized to favor α-fragmentation and Q3 selected for monohydroxylated fragments in the methyl-terminal direction of a 7-hydroxylated carbon, peaks were highly indicative of 7,*x*-diHDHA (7 < *x* ≤ 22). The observed signal was more intensive than the corresponding LLOQ-sample (*cf.* Fig. 2) and exceeded the S/N threshold, independent of whether the unstimulated chromatogram (gray) or the adjacent region in the stimulated chromatogram (red/blue) was used to determine matrix-specific noise in M2-MDM (Fig. 4A). Moreover, the semi-quantitative assessment of target peak area to total peak area of related peaks in the same Q1/Q3 trace indicated a high formation specificity of close to 99% towards RvD5 over related isomers. In comparison, PMNL/platelet co-incubations also yielded a quantifiable RvD5 peak, but at lower intensity and with several neighboring peaks indicative of substantial side product formation (Fig. 5A).

The opposite trend in formation-specificity was observed for LXs: Here, M2a-MDM yielded multiple peaks of similar intensity in the trace of 5,*x*,*y*-triHETE (5 < *x*,*y* ≤ 20), with low formation specificity below 50% towards the product matching the Rt of 5*S*,6*R*,7*E*,9*E*,11*Z*,13*E*,15*S*-triHETE (LXA_4_) (Fig. 4B). PMNL/platelet co-incubations, on the other hand, indicated a higher formation capacity but also a higher specificity for LXA_4_ formation, especially when stimulated by SACM (62%) (Fig. 5B). While not sensitive enough for trihydroxylated species, semi-quantitative chiral analysis confirmed the preferential formation of *S*-enantiomers (especially 12*S*-HETE) for enzymatic pathway markers of LXA_4_ biosynthesis (Supplementary Fig. S1D).

Unspecific LXA_4_ formation in M2a-MDM limited the available noise regions to less than 5× FWHM but produced reliable results of S/N > 5 when calculated manually (Supplementary Fig. S5F) or by peak-to-peak algorithm (Supplementary Fig. S5G) – but not by SD algorithm (Supplementary Fig. S5H). Relative S/N also yielded higher values (Supplementary Fig. S5I), as did manual calculation relative to the unstimulated matrix (Supplementary Fig. S5F). This was likely a consequence of N being a function of both intensity-dependent (multiplicative) and -independent (additive) factors, that were differently affected by the elevated baseline observed after stimulation. Thus, relative noise proved useful as a supervised screening tool (S/N_relative_ > 10) but did not replace manual calculation (S/N > 5) based on the immediate peak vicinity in the same chromatogram.

A singular peak in the Q1/Q3 of 5,*x*-diHEPE (5 < *x* ≤ 20) matching the Rt of RvE4 (5*S*,15*S*-6*E*,8*Z*,11*Z*,13*E*,17*Z*-diHEPE) was found in M2a-MDM (Fig. 4C) but not in PMNL/platelet co-incubations (Fig. 3). In matrices where analytes cannot be detected in a significant number of samples, the arithmetic average may be skewed towards outliers. As demonstrated in SACM-stimulated M2a-MDM, RvE4 could be quantified in only one highly responsive donor (>100 pg/ml) but otherwise was non-quantifiable (<33 pg/ml in two donors) or even non-detectable (<10 pg/ml in one donor). Here, showing each replicate in relationship to its LOD/LLOQ or the ratio of detectable-to-undetectable samples describes the robustness of SPM formation more transparently than a potentially misleading arithmetic mean ± SEM (46 ± 31 pg/ml).

Signals corresponding to other trihydroxylated SPM found in the Q1/Q3 of 5,*x*,*y*-triHEPE (5 < *x* ≤ 20), *x*,14,*y*-triHETE (5 < *x* ≤ 20), *x*,*y*,17-triHDHA (5 < *x* ≤ 22), and *x*,12,*y*-triHEPE (5 < *x* ≤ 20) matching the respective Rt of LXA_5_, LXB_4_, RvD1, and RvE1 were only found in PMNL/platelet co-incubations (shown in Fig. 5C for RvE1).

The Q1/Q3 of 13,*x*-diHDHA (13 < *x* ≤ 22) matching MaR2 (13*R*,14*S*-4*Z*,7*Z*,9*E*,11*E*,16*Z*,19*Z*-diHDHA) yielded several peaks close to the LOD in M2-MDM (Fig. 4D) but was robustly observed above the LLOQ in PMNL/platelet co-incubations (Fig. 5D). Interestingly, a characteristically shaped peak was also observed in the Q1/Q3 of MaR1 (7*R*,14*S*-4*Z*,8*E*,10*E*,12*Z*,16*Z*,19*Z*-diHDHA). However, closer evaluation revealed a small but reproducible difference in Rt compared to the synthetic protiated and deuterated standards, indicating that M2a-MDM and PMNL/platelet co-incubations release a *x*,14-diHDHA (2 < *x* ≤ 13) MaR1-like isomer that – albeit not identical in configuration – yielded the same accurate *m*/*z* as synthetic MaR1 (Supplemental Fig. S2).

In the Q1/Q3 of 10,*x*-diHDHA (13 < *x* ≤ 22), a minor peak matched the Rt of PDX (10*S*,17*S*-4*Z*,7*Z*,11*E*,13*Z*,15*E*,19*Z*-diHDHA) that co-eluted with specific interference in M2a-MDM (Fig. 4E) but was resolved in PMNL/platelet co-incubations (Fig. 5E).

Similar to the signals observed for LXA_4_ in M2a-MDM or RvD5 in PMNL/platelet co-incubations, the Q1/Q3 of *x*,12-HETE (2 < *x <* 12) used for LTB_4_ analysis also yielded multiple peaks in M2a-MDM (Fig. 4F) and co-incubations (Fig. 5F). Here, however, the availability of standard materials allowed to match these signals to LTB_4_ (partially coeluting with (5*S*,12*S*)-diHETE at high concentrations) and its stereoisomers *trans*- and *epi-trans*-LTB_4_.

### Oxylipin formation in vivo

**Fig. 6:**
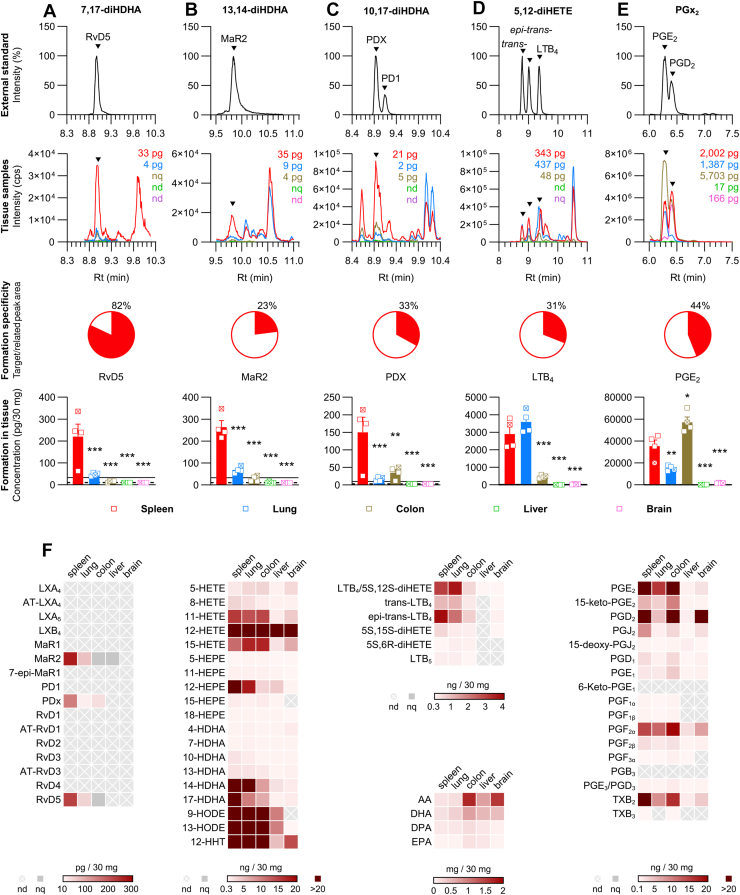
Organ-specific distribution of oxylipins in healthy C57BL/6JRj mice. Brain, colon, liver, lung and spleen were harvested from 3-month-old male and female C57BL/6JRj mice. Oxylipins were extracted by SPE and determined by UHPLC-MS/MS. Chromatograms show a representative donor and indicate the amount found on column. Pie charts show mean semi-quantitative formation specificity in spleen (target peak area divided by related peak areas). Bar charts show calculated concentrations (means ± SEM) from each animal (n = 4), indicating the replicate whose chromatogram is shown (☒), the limit of detection (LOD; dashed line) and the lower limit of quantification (LLOQ; solid line). Statistical analysis was performed by lognormal RM one-way ANOVA with Dunnett’s multiple comparisons test and single pooled variance. ∗*P* < 0.05; ∗∗*P* < 0.01; ∗∗∗*P* < 0.001. Representative chromatograms and resulting amounts of (A) RvD5, (B) MaR2, (C) PDX/PD1, (D) LTB_4_, and (E) PGE_2_. F: Heatmap showing mean oxylipin formation in all screened organs.

Finally, we addressed oxylipin analysis in tissue of healthy C57BL/6JRj mice to investigate SPM occurrence in vivo (Supplementary Table S3). In spleen, peaks matching RvD5 (Fig. 6A), MaR2 (Fig. 6B), and PDX (Fig. 6C) were observed alongside additional signals of comparable intensity, indicating robust – albeit non-specific – formation of dihydroxylated SPM in healthy lymphatic tissue (>200 pg/30 mg tissue) at levels well above those observed in lung, liver, colon, or brain. In contrast, other oxylipins such as LTB_4_ (Fig. 6D) and PGE_2_ (Fig. 6E) showed an organ tropism in favor of lung and colon tissue, respectively, matching the role of these LM in coordinating the pulmonary immune response and gastrointestinal perfusion. The overall LM profile was dominated by PUFA (especially AA in colon and brain), followed by monohydroxylated oxylipins (12-HETE in spleen and lung), COX products (PGE_2_ in colon; PGD_2_ and TXB_2_ in spleen), and 5-LOX products (LTB_4_ in lung and spleen), while trihydroxylated SPM were not quantifiable (Fig. 6F).

## Discussion

SPM have been documented as powerful immunoresolvents that promote the resolution of inflammation. Owing to their high potency, SPM occur at low bioactive concentrations, rendering their detection and analysis challenging. Here, we provide comprehensive information on the systematic assessment of oxylipins, with focus on SPM, produced across a broad range of commonly employed in vitro and ex vivo matrices using a quantitative UHPLC-MS/MS method that was validated in accordance with recommendations by the International Lipidomics Society.

### Standard materials and implications for accuracy

Standard curves were established using authentic—but not certified—reference materials. The lack of standards with certified concentrations is a known limitation in the field of SPM bioanalysis that may cause a systematic error (bias) to overall accuracy. A possible remedy is presented by Dooley *et al.*, who recommend verification of the nominal concentration via UV-vis spectroscopy, provided the molar absorption coefficient is known or can be predicted (34). However, the degree of imprecision introduced by our UV-vis spectrometer when measuring stocks of a presumed shipping concentration of 10 μg in 100 μl led us to favor nominal concentration.

Similarly, IS-normalized standard curves were established using a limited selection of SIL-IS. Ideally, a protiated or carbon-13-labeled analogue of each SPM would compensate for incomplete recovery due to SPE and ion suppression due to matrix effects by matching the chemistry and Rt of the corresponding target analyte. However, structure-matched SIL-IS are not commercially available for every SPM and their use does not always yield the highest accuracy in oxylipin quantification, as has been shown for certain mono- and dihydroxylated species (35). Our selection of SIL-IS consisted of a single diHETE (*d*_4_-LTB_4_) for SPM belonging to diHEPE and diHDHA, a triHDHA (*d*_5_-RvD2) for triHDHA, and a triHETE (*d*_5_-LXA_4_) for triHETE and triHEPE. While this selection was informed by cost/benefit considerations compatible with high-throughput use, increasing the number of SIL-IS has the potential to further improve accuracy and ease identification.

### Endogenous analytes and implications for selectivity

Ideally, all calibrators and QC use a blank biological matrix as a diluent to determine unspecific interference. Such an approach is methodologically challenging for SPM, since eukaryotic matrices often contain trace concentrations of some oxylipins (i.e., specific interference) (36). Bioanalytical guidelines such as ICH M10 (26) recommend the following approaches for analytes that are also endogenous molecules: (*i*) surrogate matrix which can range from inorganic buffers to stripped biological matrices; (*ii*) surrogate analytes such as ^2^H- or ^13^C-labeled standards which are not found in the biological matrix; (*iii*) background subtraction which mathematically corrects for the presence of analytes in the matrix; or (*iv*) a standard addition approach which requires separate standard curves for each biological sample.

Within oxylipin analysis, the surrogate analyte approach (*ii*) is not feasible, because SIL standards are not available for all SPM of interest. Matrix-specific background subtraction (*iii*) is often not practical, because a single method often aims to target multiple matrices (here: multiple cell- and tissue-types) and considerable inter-individual variability is observed even within the same matrix, depending on the work-up procedure (37) and storage conditions (38). Standard addition (*iv*) is incompatible with the high throughput demanded of routine oxylipin bioanalysis (here averaging 2500 samples/month). Hence, we opted for surrogate matrix (*i*) and confirmed the absence of potentially interfering compounds in all unstimulated in vitro matrices. Since samples from mice originated from healthy (unstimulated) animals, this approach could not be pursued in vivo. However, absence of SPM in non-lymphoid tissue indicated adequate selectivity.

### Analyte accumulation and implications for sensitivity

The LLOQ found for most SPM (10–100 pg/ml matrix) was similar to reported methods using the same instrument (20–100 pg/ml with Qtrap5500 (39)), but higher than reported for newer instruments (6–36 pg/ml on Qtrap6500 (40); ∼1 pg/ml with Qtrap6500+ (34, 35, 41); >5 pg/ml with Agilent 6470 (42); >10 pg/ml with Xevo-TQ-XS; (43)). The lower sensitivity on older instruments may be partially explained by instrument contamination: although we did not observe any carry-over for SPM, a decade of high-throughput lipidomics contributed to latent analyte accumulation and less stable baselines compared to pristine conditions. Methodologically, the S/N-, accuracy-, and precision-based LLOQ presented here considered all steps of the bioanalysis (including SPE) and not merely instrumental sensitivity, resulting in a higher LOD than previously reported on the same instrument (44). Higher sensitivity may be achieved by circumventing Rt-specific ion suppression (identifiable in post-column infusion experiments), but may no longer be a priority in SPM bioanalysis, given recent insights into their mechanism of action and potency: While high concentrations of RvD5 in the μM-range displace PGE_2_ from binding at EP4 (K_i_ = 469 ng/ml), low nanomolar concentrations (translating to more than 360 pg/ml) suffice for allosteric modulation of EP4 (10). Such concentrations were easily surpassed in terms of sensitivity (even on a used Qtrap5500) and M2a-MDM formation capacity.

### Chiral and achiral implications for specificity

The presented RP-UHPLC method is achiral and relies on a single Q1/Q3 transition per analyte. Accordingly, peaks attributed to SPM by this method could also have been caused by enantiomers, other isomers, and even non-isomeric isobars sharing the same nominal precursor and fragment *m*/*z*. Here, EPI scans were relied upon to confirm the identity of analytes observed for the first time in an unexpected matrix.

Increasing the number of transitions per analyte improves achiral specificity, since it allows continuous monitoring of additional fragments that ideally arise from α-cleavage next to each hydroxy group. Often, only the most intensive transition is used for quantification (“quantifier”), while each auxiliary transition provides further evidence for the analyte’s constitution (“qualifier”). The deviation of the quantifier/qualifier peak area ratio suggested matrix interfering with PD1 but also confirmed the presence of PDX in renal disease patient serum (0–7 ng/ml) and in plasma of sepsis patient (0–2 ng/ml; one outlier close to 30 ng/ml) (40). Similarly, we observed matching MS/MS spectra for PDX in vivo and in vitro. Unfortunately, concurrent monitoring of twice the number of transitions (qualifier/quantifier) negatively affected the number of data points (when fixing dwell time) or baseline stability (fixed cycle time) for semi-targeted approaches (>70 analytes) on our instrument and was therefore reserved for investigations into specific SPM.

Chiral analysis of oxylipins provides insight into the biosynthetic origin of SPM, since human LOX isoforms typically generate the *S*-enantiomer (except for 12*R*-LOX found in skin) (45). Enantioseparation can be achieved via derivatization followed by GC (46) or directly on polysaccharide-based stationary materials followed by LC (47) or SFC (43, 48). Unfortunately, chiral stationary phases often exhibit a lower selectivity towards fatty acid diastereomers, require non-RP solvents that impede MS-interfacing, or lose efficiency when operated in RP mode, resulting in a lower achiral resolution. An elegant solution is the 2D coupling of achiral and chiral techniques by collecting (heartcutting) fractions of interest, pre-separated on a non-enantioselective stationary phase to be then analyzed on an enantioselective stationary phase (25). However, 2D approaches demand extensive instrumentation and increase the runtime. Likewise, SFC is an excellent separation technique but not part of standard instrumentation or training. Thus, achiral 1D-RP remains the most common analytical mode but relies on dedicated chiral reports to justify stereospecific nomenclature (including trivial names).

Regarding monohydroxylated species, we observed high levels of 12-HETE across platelet-containing matrices, which have previously been shown to consist mainly of the 12*S*-HETE in stimulated blood, while circulating plasma contained the autooxidation product *rac*-12-HETE (49). For dihydroxylated products, we observed peaks that matched the Rt and MS/MS of SPM standards (including RvD5, PDX, and RvE4) predominantly in M2a-MDM. This designation is supported by others showing that the correct enantiomer dominates in signals of these analytes in M2a-MDM, but not in plasma (25). In the same study, M2a-MDM produced a single peak matching the Rt of PD1 (10*R*,17*S*-diHDHA) under RP conditions but separated into a mixture of at least three stereoisomers on a chiral stationary phase (25). Using our RP method, a slight Rt deviation also correctly indicated the mismatch between the dominant 10,17-diHDHA signal and PD1. Similarly, we found a reproducible mismatch for 7,14-diHDHA/MaR1, indicating that M2a-MDM produce higher amounts of a related diastereomer. The absence of MaR1 in primary human blood cells is supported by previous studies that yielded isomers different from synthetic MaR1 when incubating MaR1 precursors with the putative cellular source of MaR1 (MDM) (50) or its putative enzymes (isolated 5-LOX or 15-LOX-1/2) (51).

In PMNL/platelets, we observed dominant peaks for trihydroxylated analytes matching the Rt and MS/MS spectra of LXA_4_, LXB_4_, and RvE1. The designation of LXA_4_ and LXB_4_ is supported by Serhan *et al.*, who demonstrated 12-LOX-mediated formation of LXA_4_ from 5-LOX-derived LTA_4_ (52), and by Lehmann *et al.*, who showed the correct enantiomer to dominate in PMNL/platelet co-incubations (53). A 2D chiral analysis of RvE1 remains to be reported, however, and thus it is possible that the signals we observed in co-cultures may be attributable to isomers. Indeed, the trace we employed was optimized for *x*,12,*y*-triHEPE species and therefore did not differentiate between RvE1 (5*S*,12*R*,18*R,*6*Z*,8*E*,10*E*,14*Z*,16*E-*triHEPE) and possible 6*E*-isomers, which may arise after non-enzymatic hydrolysis of a common RvE1-precursor in PMNL (54).

In vivo, we robustly detected peaks matching dihydroxylated PDX, RvD5, and MaR2 in healthy murine organs with a clear tropism for spleen. Previous reports employing strict quantifier/qualifier criteria have confirmed the presence of PDX (40) and RvD5 (34) in human inflamed plasma and serum standard reference material, respectively, but a conclusive chiral analysis of in vivo SPM formation remains to be reported.

### Changes in reporting practices

The technical recommendations for oxylipin analysis (14) introduce three key innovations: the official deprecation of ELISA for SPM quantification from complex matrices, the formal inclusion of S/N in the peak acceptance criteria, and the requirement for reporting primary chromatograms. The chromatograms must be unsmoothed, include a standard near the LLOQ and a representative biological sample, and display the amount calculated on-column (14). A wide array of studies has utilized ELISA to quantify specific SPM, mostly focusing on RvD1 and RvE1 in patient serum and plasma in response to acute inflammation. The reported levels are similar to what has been shown by LC-MS/MS (low pg/ml range). However, individual studies using ELISA have reported much higher SPM levels, e.g., in serum of COVID-19 patients (RvE1 > 1000 pg/ml) (15). Moreover, cross-reactivity against PUFA and various abundant oxylipins—while validated by the manufacturer to be less than 0.01%—introduces the potential for error due to differences in relative abundance by several orders of magnitude. Moreover, since these studies focus on selected SPM, changes in other SPM (let alone uncharacterized isomers) remain unnoticed.

The S/N is based on the baseline fluctuation N. Accurate determination of height-based N requires a sufficiently wide (5 × FWHM) region of the chromatogram free of additional peaks, which often is not available in bioanalytical methods and therefore is absent from the respective ICH M10 guideline. While the technical recommendations established manual calculation based on unsmoothed chromatograms as the default, software tools remain possible if shown to yield comparable results. Here, we show relative noise (adjusted by a factor of 2) as suitable for identifying critical peaks that require manual S/N calculation, or as an alternative when closely eluting peaks prevent manual calculation.

Providing primary chromatograms in publications resembles the longstanding practice of providing scans of exemplary Western blots. These chromatograms not only provide support for the detectability of SPM and their resulting levels but also evidence for the presence/absence and relative quantity of related compounds: Although only those peaks for which matching authentic standards are available can be quantified, the absence of additional peaks provides insight into the specificity (and likely enzymatic origin) of SPM formation, while side peaks indicate non-specific (auto-)oxidation—information that is crucially missing when assessed by deprecated techniques such as ELISA. Our chromatograms of oxylipins formed in M2a-MDM demonstrate specific formation of a dominating isomer for RvD5 and RvE4, but unspecific formation of multiple isomers for lipoxins—of which LXA_4_ appears to be only a minor side product. Conversely, PMNL/platelets specifically formed LXA_4_, whereas an almost equally intensive peak was detected close to RvD5. Side product formation is not apparent from bar charts or tables listing only the assigned products. This may account for false positive ELISA reports, since their cross-reactivity is unknown.

## Conclusion

Recommendations by the International Lipidomics Society were followed to validate a bioanalytical quantification method for 19 SPM and 53 related oxylipins using SPE and UHPLC-MS/MS. Our method demonstrated robust formation of trihydroxylated SPM in stimulated co-incubations of human PMNL and platelets, and of dihydroxylated SPM in human M2a-MDM and murine splenic tissue. Importantly, a mid-field mass spectrometer (QTrap 5500) was sufficient to demonstrate SPM formation well above a transparently validated LLOQ based on the S/N, accuracy, and precision. Analysis of other cell types or tissues did not yield specific formation (singular peaks) or significant amounts of SPM. Similarly, no SPM were observed in unstimulated cells or in stimulated human blood under the present experimental and analytical conditions. Taken together, this confirms that SPM are biosynthesized in vitro and in vivo—but also that biosynthesis is tightly regulated and dependent on select stimulatory conditions, cell types, and tissue origin. Future research will need to address the precise GPCR targeted by SPM and their downstream signal transduction mechanisms (via orthosteric orphan receptor activation or via allosteric EP4 modulation) and determine whether endogenous SPM levels are sufficient to influence biological outcomes or require exogenous intervention to promote the LM class-switch toward pro-resolving mediators.

## Data availability

All data are contained within the manuscript and supplementary information.

## Supplemental data

This article contains supplemental data.

## Conflict of interest

The authors declare that they have no conflicts of interest with the contents of this article.
