# Supplementary material for: Validated bioanalysis of oxylipins confirms specialized pro-resolving mediator formation in vitro and in vivo
Source: J Lipid Res. 2026 Jun 8;67(7):101075. doi: 10.1016/j.jlr.2026.101075 (PMC13380050; doi:10.1016/j.jlr.2026.101075)

SUPPLEMENTARY FIGURES

Validated bioanalysis of oxylipins confirms specialized pro-resolving mediator formation *in vitro* and *in vivo*

Robert K. Hofstetter[a], Markus Werner[a], Patrick Schädel[a], Mareike Wichmann-Costaganna[a], Katrin Fischer[a], Vera Bruggink[a], Katharina P.L. Meyer[a], Lukas Peltner[a], Kerstin Günther[a], Vivien Bachmann[a], Hannes Engelbrecht[a], Clemens Gutjahr[a], Bill Perkowski[a], Gregor Griebel[a], Nur Banu Bal[b], Nico Ueberschaar[c], Paul M. Jordan[a,d], and Oliver Werz[a,d]**

Table of contents

	Title	Page
**Fig. S1**	Chiral analysis of SPM precursors	2
**Fig. S2**	MaR1 signals in M2a-MDM	3
**Fig. S3**	Chromatograms of unstimulated M2a-MDM compared to solvent blanks	4
**Fig. S4**	SPM calibration curves showing slope, *y*-intercept, R^2 value, accuracy, signal-to-noise ratios, and chromatograms	24
**Fig. S5**	Comparison of S/N calculation methods	82
**Fig. S6**	Recovery	91
**Fig. S7**	Matrix effects of the homogenization buffer on the retention time	92
**Fig. S8**	Principal component analysis of *in vitro* and *ex vivo* oxylipin profiles	93

Fig. S1: Chiral analysis of SPM precursors

Synthetic reference material of racemic SPM precursors was obtained from Cayman Chemical Company (Ann Arbor, MI, USA). Where available, peaks were designated as *S*- and *R*-enantiomers (EN) using enantiopure reference material from the same supplier. Chiral separation was achieved using a Daicel Chiralpak AD-RH (150 × 4.6 mm; 5 µm) stationary phase with mobile phase A (water:acetonitrile:formic acid; 98:2:0.1) and B (acetonitrile) and the following gradient of B: 0.0-0.2 min (50%), 0.2-13.0 min (50%→98%), 13.0-18.0 (100%), 18.0-21.0 min (50%). Flow rate: 0.4 mL/min (25 °C). Data was acquired on a Waters Acquity UHPLC (Milford, MA, USA) coupled to a Sciex QTrap 5500 triple-quadrupole mass spectrometer (Framingham MA, USA) using a Turbo V ion source interface controlled by AB Sciex ANALYST Version 1.6.3 (2015; build 1569). Global and analyte-specific MS parameters were identical to the achiral method described in the manuscript. Chromatograms were compared to unstimulated and stimulated cells (1% SACM; 90 min).

A) DHA metabolism to oxylipins.
Putative pathway of RvD5 biosynthesis.

B) Chiral analysis of oxylipins in M2a-MDM.
Stimulation of human M2a-MDM (2×10^6 cells/mL) resulted in enantio-preferential formation, indicating enzymatic formation.

C) AA metabolism to oxylipins.
Putative pathway of LXA_4 biosynthesis.

D) Chiral analysis of oxylipins in co-incubations of PMNL/platelets.
Stimulation of human PMNL (1×10^7 cells) and platelet (2.5×10^8 cells) co-incubations preferentially yielded the *S*-enantiomer, indicating enzymatic formation of SPM precursors. Note the formation of considerable amounts of 12*S*-HETE even under unstimulated conditions, as well as a non-HETE product of the same *m*/*z* that likely corresponded to an isobaric epoxyeicosatrienoic acid (EET). Due to the lower sensitivity of the chiral method LXA_4 could not be quantified.

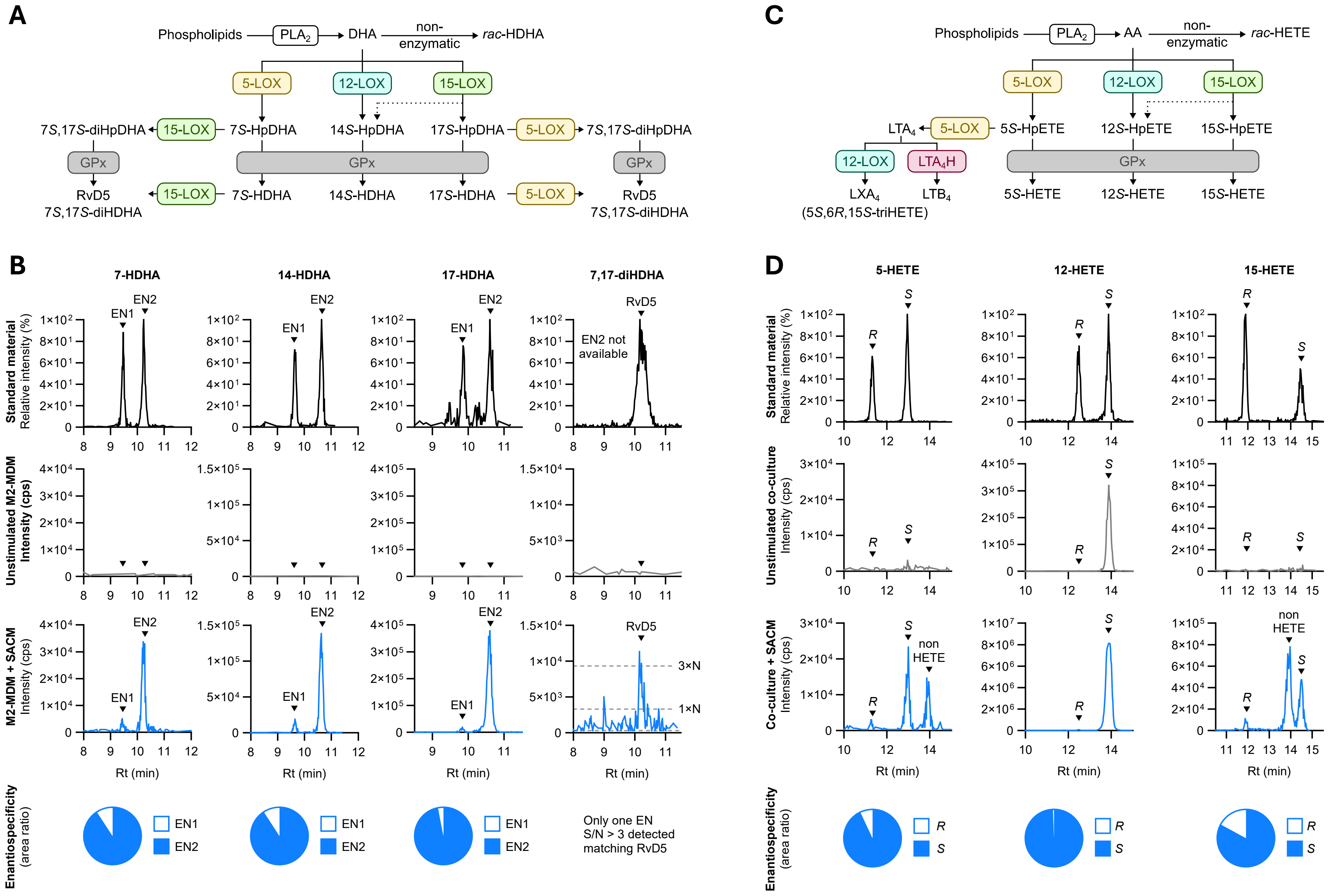

Fig. S2: MaR1 signals in M2a-MDM

Representative chromatograms of synthetic and M2a-MDM-derived MaR1. M2a-MDM (2 × 10^6 cells/mL) were stimulated with SACM (1%) for 90 min. Synthetic reference material of maresin 1 (MaR1) with the stereochemistry of 4*Z*,7*R*,8*E*,10*E*,12*Z*,14*S*,16*Z*,19*Z*-dihydroxydocosahexaenoic acid was obtained from Cayman Chemical Company (Ann Arbor, MI, USA; Item No. 10878). Samples were extracted by SPE and compared on two different LC-MS platforms.

A) Evidence for the distinctness of synthetic MaR1 and the biological product. Samples were analyzed by UHPLC-MS/MS on a Waters Acquity UHPLC (Milford, MA, USA) coupled to a Sciex QTrap 5500 triple-quadrupole mass spectrometer (Framingham MA, USA) as described in the main text. Two different lots of the synthetic reference standard were run at the beginning (reference standard 1) and end (reference standard 2) of the batch, with biological samples obtained from four different donors running in-between. While the reference standards yielded consistent retention time maxima (9.17 min), a robust shift in retention time was observed in biological samples (9.22-9.25 min). Mean peak regions as defined by the full width at half maximum (FWHM; indicated in gray) did not match the synthetic reference material, indicating distinct configuration, conformation, or even non-isomeric isobars.

B) Evidence for the similarity between synthetic MaR1 and the biological product. Pooled samples were analyzed by UHPLC coupled with high resolution mass spectrometry using a Thermo (Bremen, Germany) Vanquish VF-P10-A binary pump, VF-A10-A auto sampler which was set to 10 °C and which was equipped with a 25 µL injection syringe and a 100 µL sample loop. The column was kept at 25 °C within the column compartment VH-C10-A. The same column, eluent, and gradient was used as in A). Mass spectra were recorded with Thermo (Bremen, German) Exploris 480 orbitrap mass spectrometer coupled to a heated electrospray source (HESI). For monitoring a full scan mode was selected with the following parameters. Polarity: negative; scan range: 200 to 1000 *m*/*z*, resolution: 240,000; AGC target: “standard”; maximum IT: “auto”. General settings: sheath gas flow rate: 50; auxiliary gas flow rate: 10; sweep gas flow rate: 1; spray voltage: 2.5 kV; capillary temperature: 325 °C; S-lens RF level: 50; auxiliary gas heater temperature: 350 °C. For MS2 mode, the following settings were made: mass range: 50-387 u; resolution: 7.500; NCE: 15.

The observed *m*/*z* of the biological product (brown) matched the calculated Q1 (359.2 ± 0.2) and Q3 (250.1211 ± 5 ppm) of the synthetic reference standard (blue), indicating that the biological product was not an unrelated compound, but an isomeric isobar of synthetic MaR1.

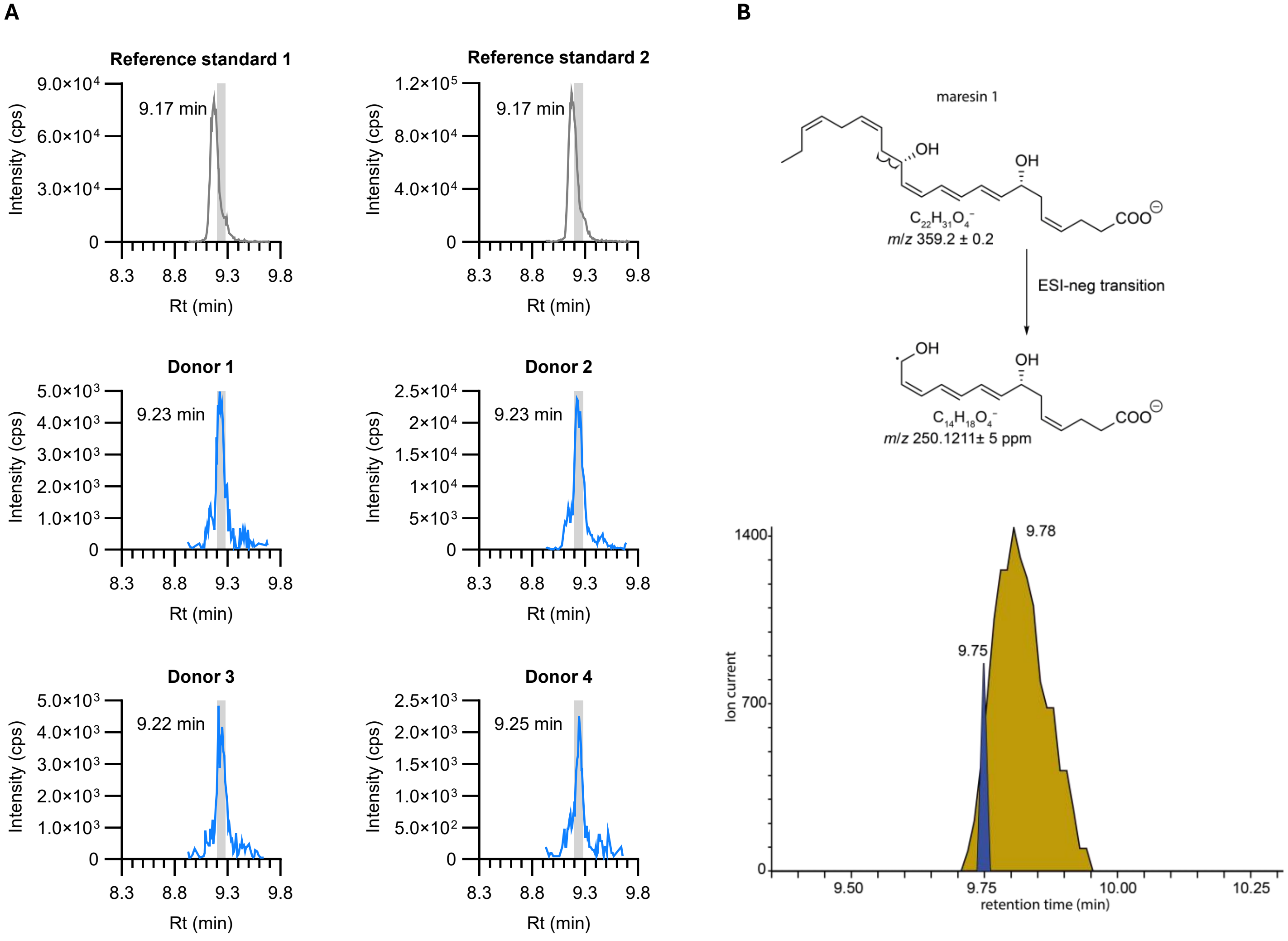

Fig. S3: Chromatograms of unstimulated M2-MDM: LXA_4

M2-MDM were obtained as described in the methods section. Cells were incubated for 90 min in PBS containing 1 mM $CaCl_2$ and then extracted by SPE as described in the methods section. Chromatograms were compared to injections of blank solvent (without prior SPE). Expected retention times of SPM were obtained by comparison with a standard mix in blank solvent (no SPE) and quality control samples yielding 1.00 and 3.33 pg on column (extracted with SPE).

Legend

M2 (N1)	M2 (N2)	M2 (N3)	M2 (N4)
Blank (N1)	Blank (N2)	Blank (N3)	Blank (N4)
Standard mix	1 pg on column	3.33 pg on column	

LXA_4

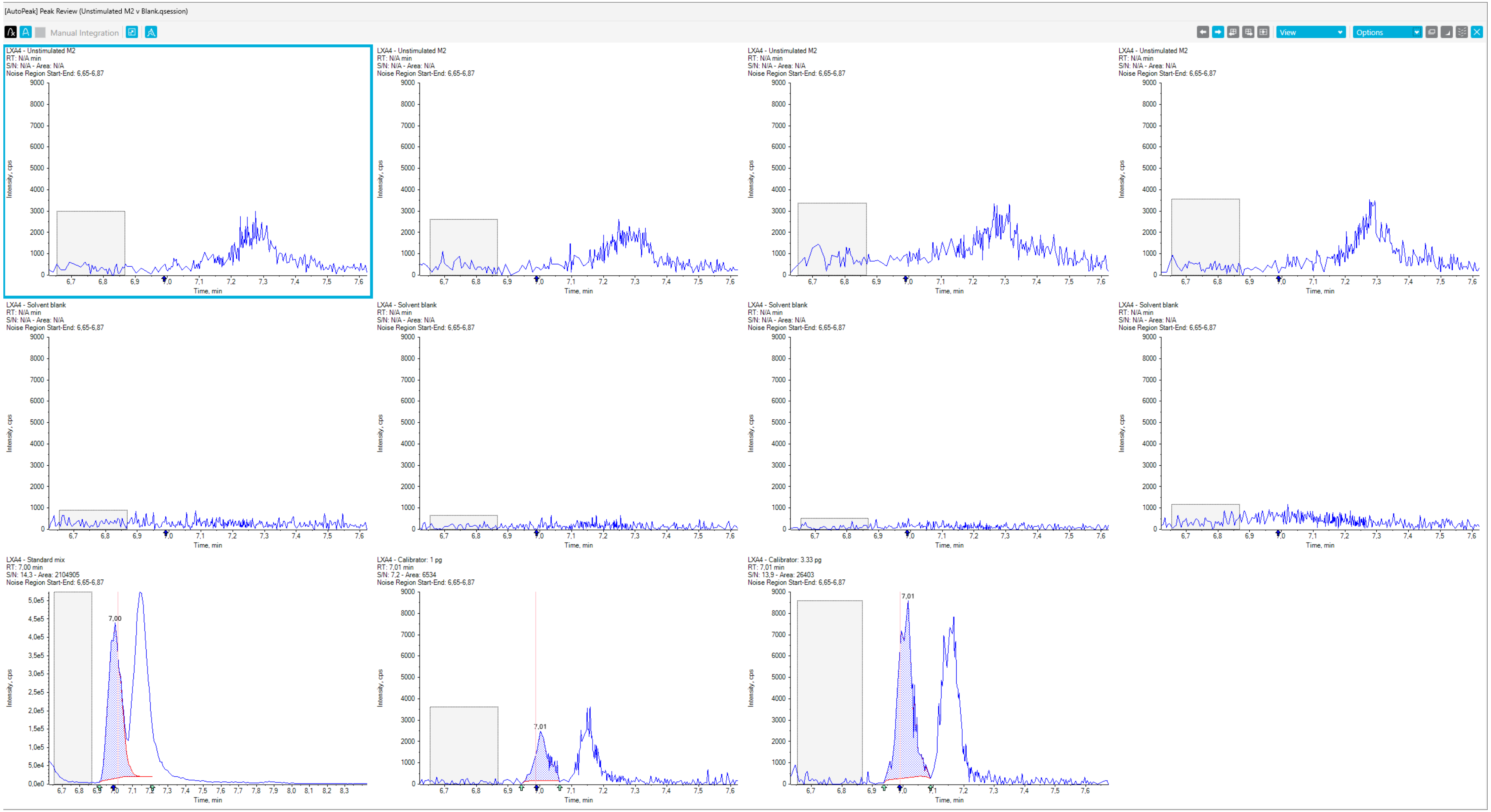
[AutoPeak] Peak Review (Unstimulated M2 v Blank.qsession)
Manual Integration
View
Options
LXA4 - Unstimulated M2
RT: N/A min
S/N: N/A - Area: N/A
Noise Region Start-End: 6,65-6,87
LXA4 - Solvent blank
LXA4 - Standard mix
RT: 7,00 min
S/N: 14,3 - Area: 2104905
LXA4 - Calibrator: 1 pg
RT: 7,01 min
S/N: 7,2 - Area: 6534
LXA4 - Calibrator: 3.33 pg
RT: 7,01 min
S/N: 13,9 - Area: 26403
Intensity, cps
Time, min

AT-LXA_4

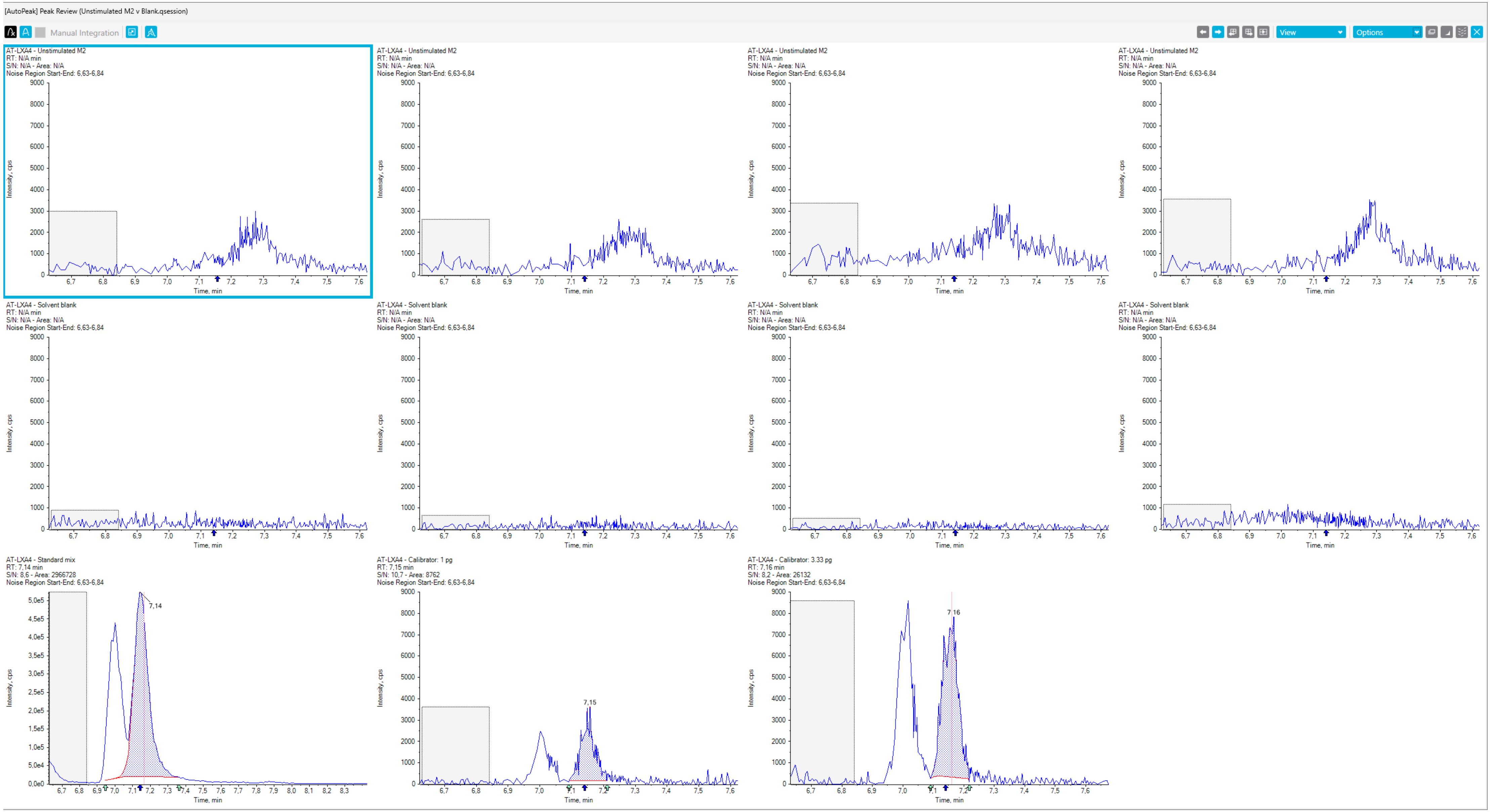
[AutoPeak] Peak Review (Unstimulated M2 v Blank.qsession)
Manual Integration
View
Options
AT-LXA4 - Unstimulated M2
RT: N/A min
S/N: N/A - Area: N/A
Noise Region Start-End: 6,63-6,84
AT-LXA4 - Solvent blank
RT: N/A min
S/N: N/A - Area: N/A
Noise Region Start-End: 6,63-6,84
AT-LXA4 - Standard mix
RT: 7,14 min
S/N: 8,6 - Area: 2966728
Noise Region Start-End: 6,63-6,84
AT-LXA4 - Calibrator: 1 pg
RT: 7,15 min
S/N: 10,7 - Area: 8762
Noise Region Start-End: 6,63-6,84
AT-LXA4 - Calibrator: 3.33 pg
RT: 7,16 min
S/N: 8,2 - Area: 26132
Noise Region Start-End: 6,63-6,84
Intensity, cps
Time, min

LXB_4

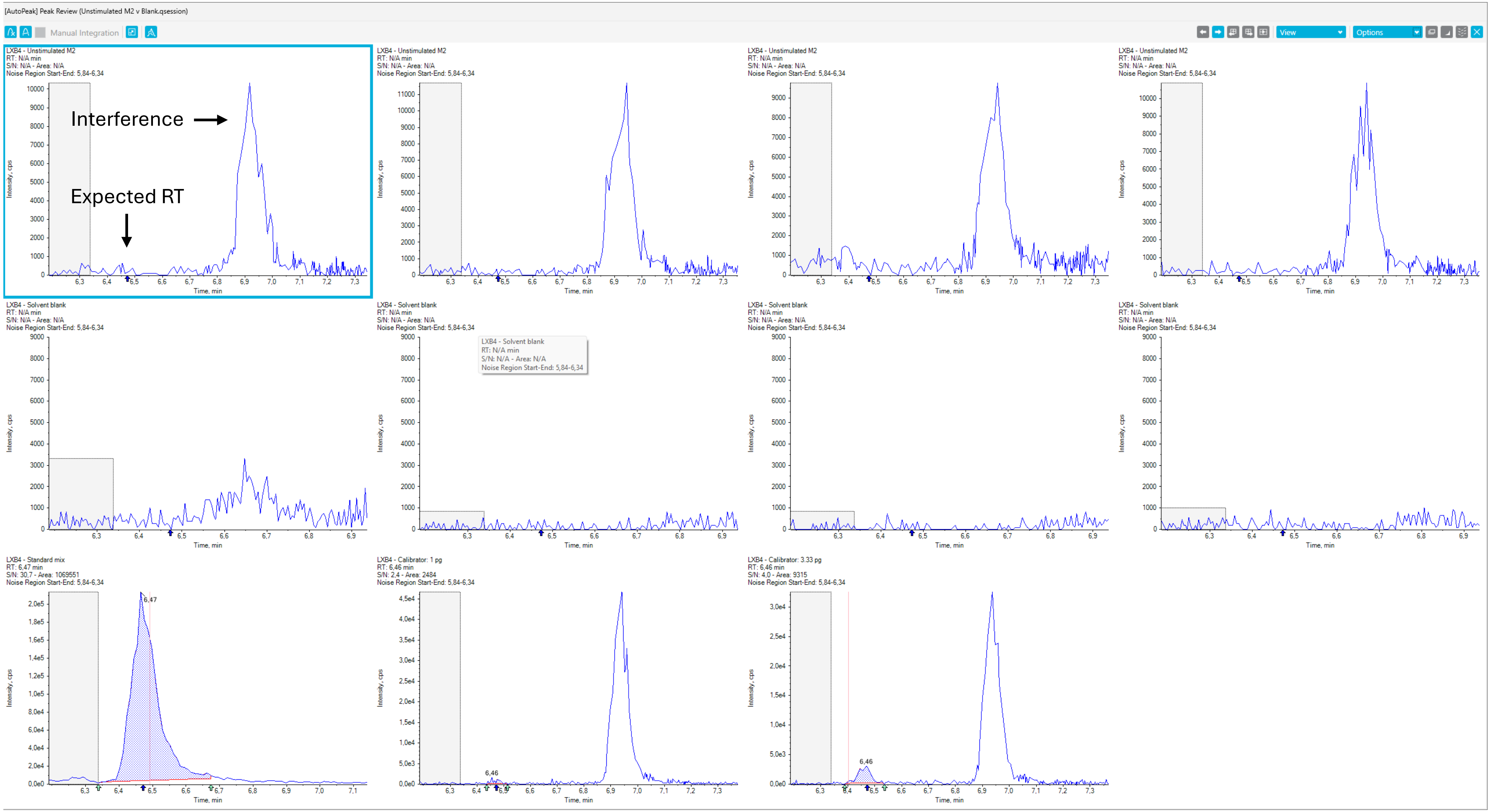
[AutoPeak] Peak Review (Unstimulated M2 v Blank.qsession)
Manual Integration
View
Options
LXB4 - Unstimulated M2
RT: N/A min
S/N: N/A - Area: N/A
Noise Region Start-End: 5,84-6,34
Interference
Expected RT
LXB4 - Solvent blank
LXB4 - Standard mix
RT: 6,47 min
S/N: 30,7 - Area: 1069551
LXB4 - Calibrator: 1 pg
RT: 6,46 min
S/N: 2,4 - Area: 2484
LXB4 - Calibrator: 3.33 pg
RT: 6,46 min
S/N: 4,0 - Area: 9315
Intensity, cps
Time, min

RvD1

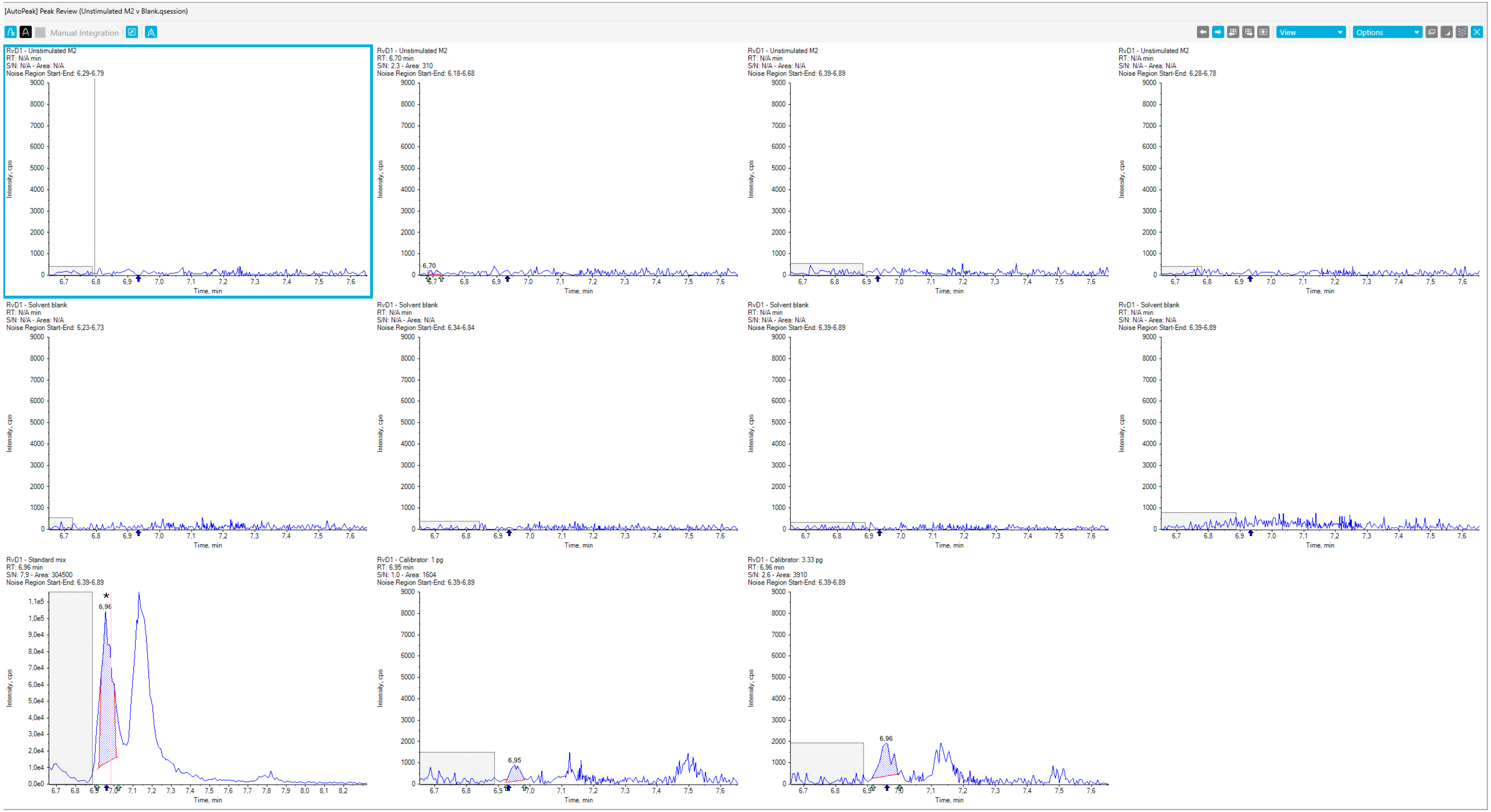

*Standard mix:
Integration indicates acceptable Rt region for peak maximum to warrant identification ("retention time match"), not quantitation

AT-RvD1

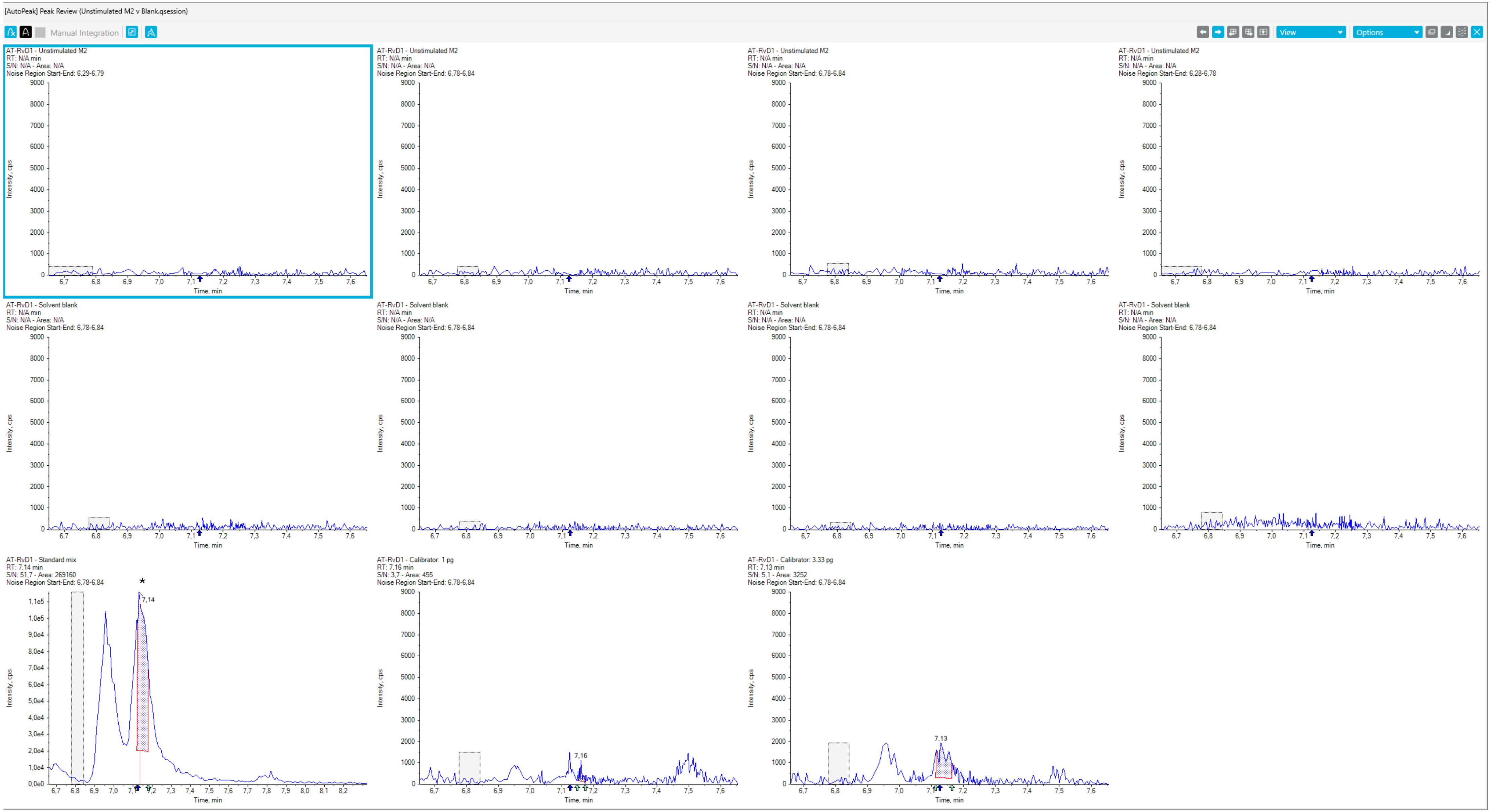

*Standard mix:
Integration indicates acceptable Rt region for peak maximum to warrant identification ("retention time match"), not quantitation

RvD2

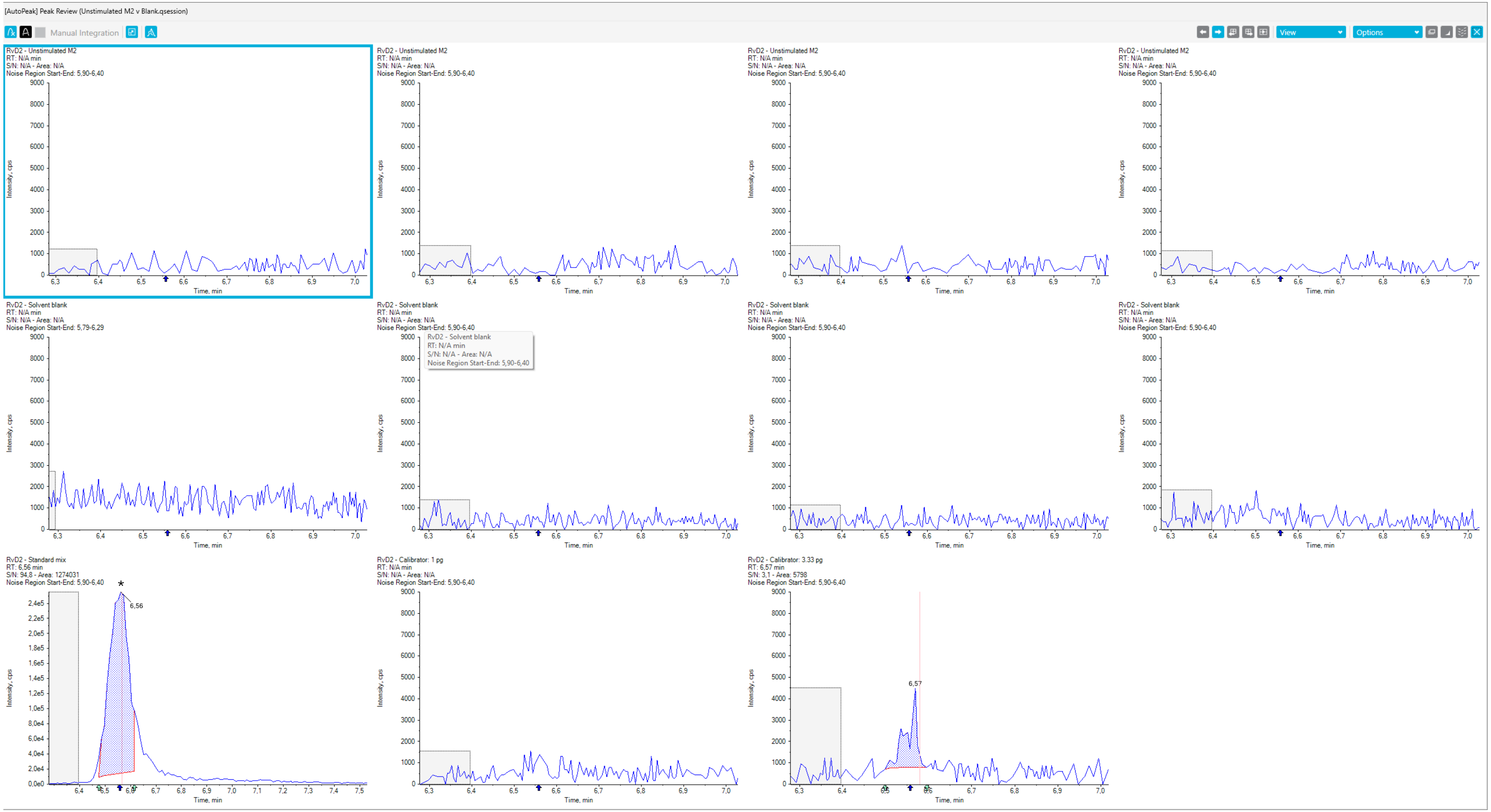

*Standard mix:
Integration indicates acceptable Rt region for peak maximum to warrant identification ("retention time match"), not quantitation

AT-RvD3

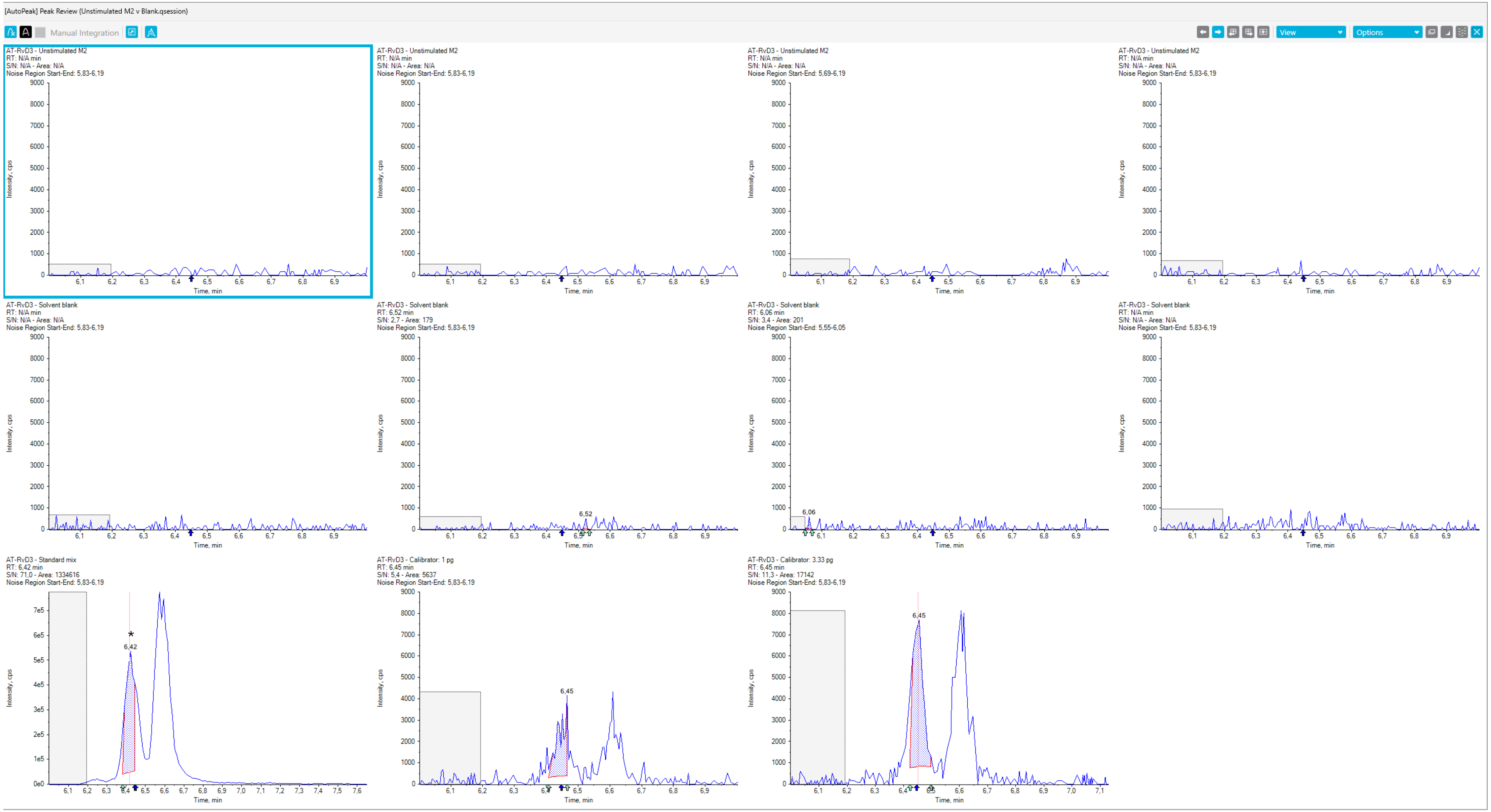

*Standard mix:
Integration indicates acceptable Rt region for peak maximum to warrant identification ("retention time match"), not quantitation

RvD3

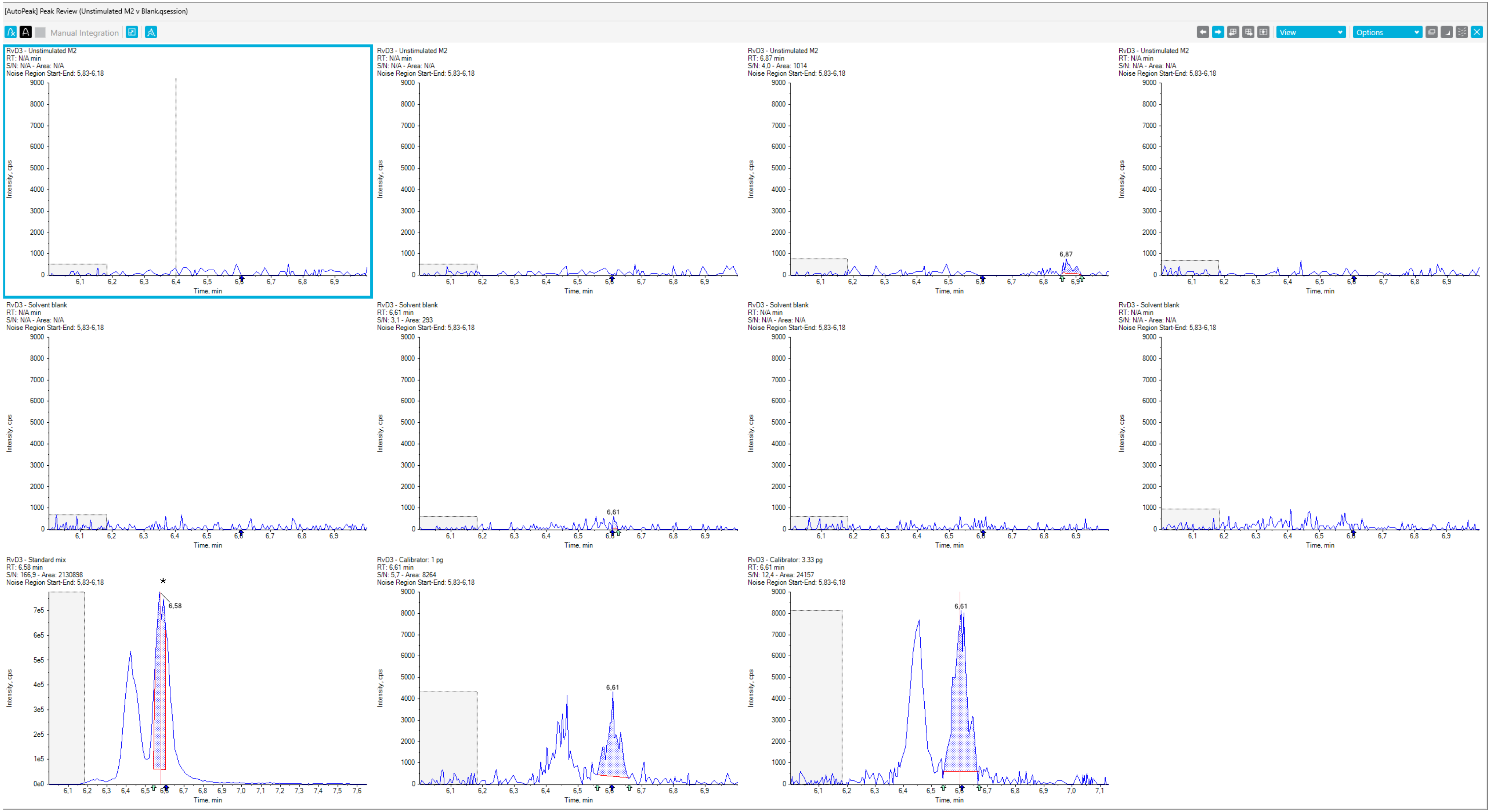

*Standard mix:
Integration indicates acceptable Rt region for peak maximum to warrant identification (“retention time match”), not quantitation

RvD4

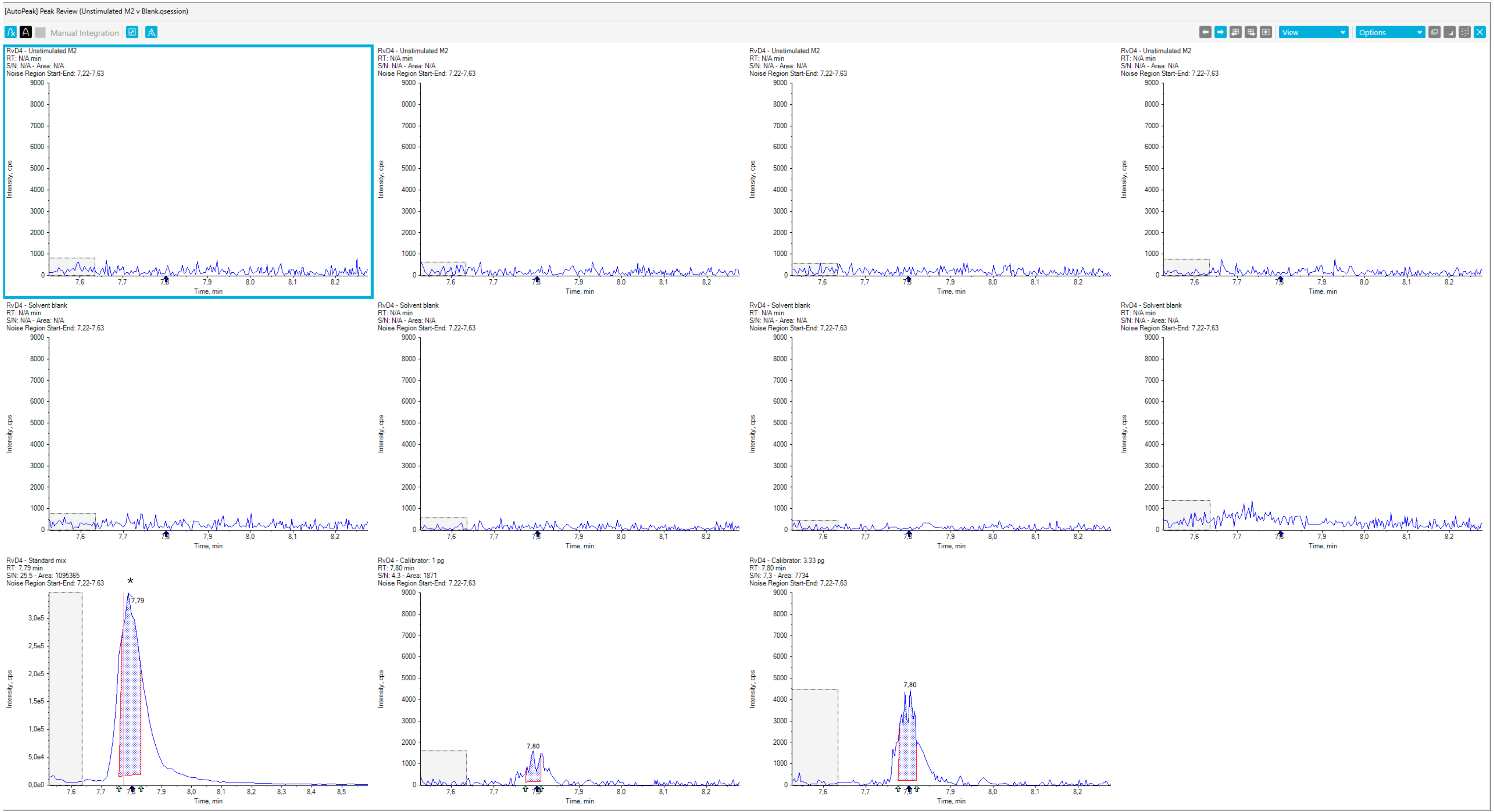

*Standard mix:
Integration indicates acceptable Rt region for peak maximum to warrant identification ("retention time match"), not quantitation

MaR1

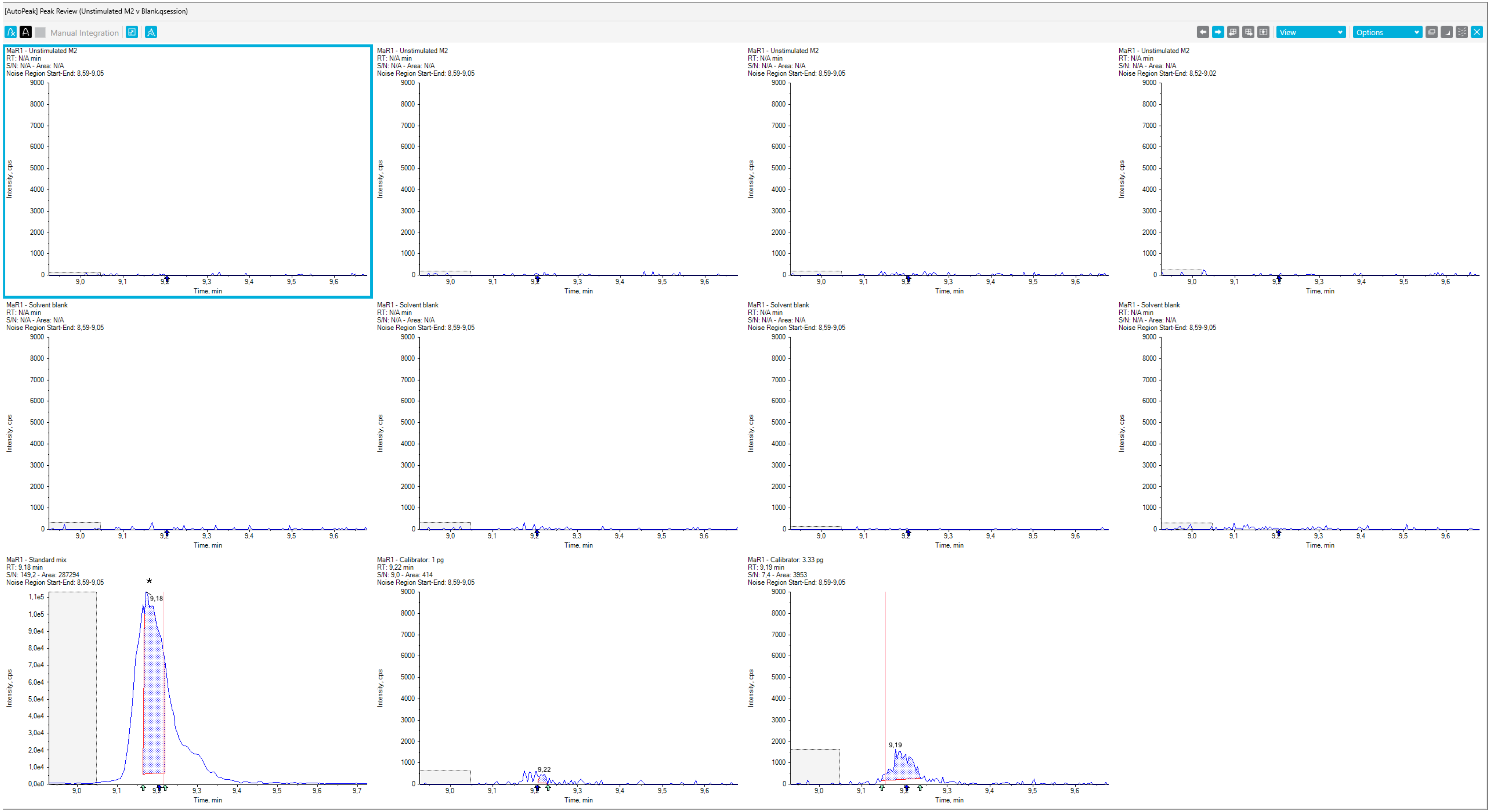

*Standard mix:
Integration indicates acceptable Rt region for peak maximum to warrant identification (“retention time match”), not quantitation

MaR2

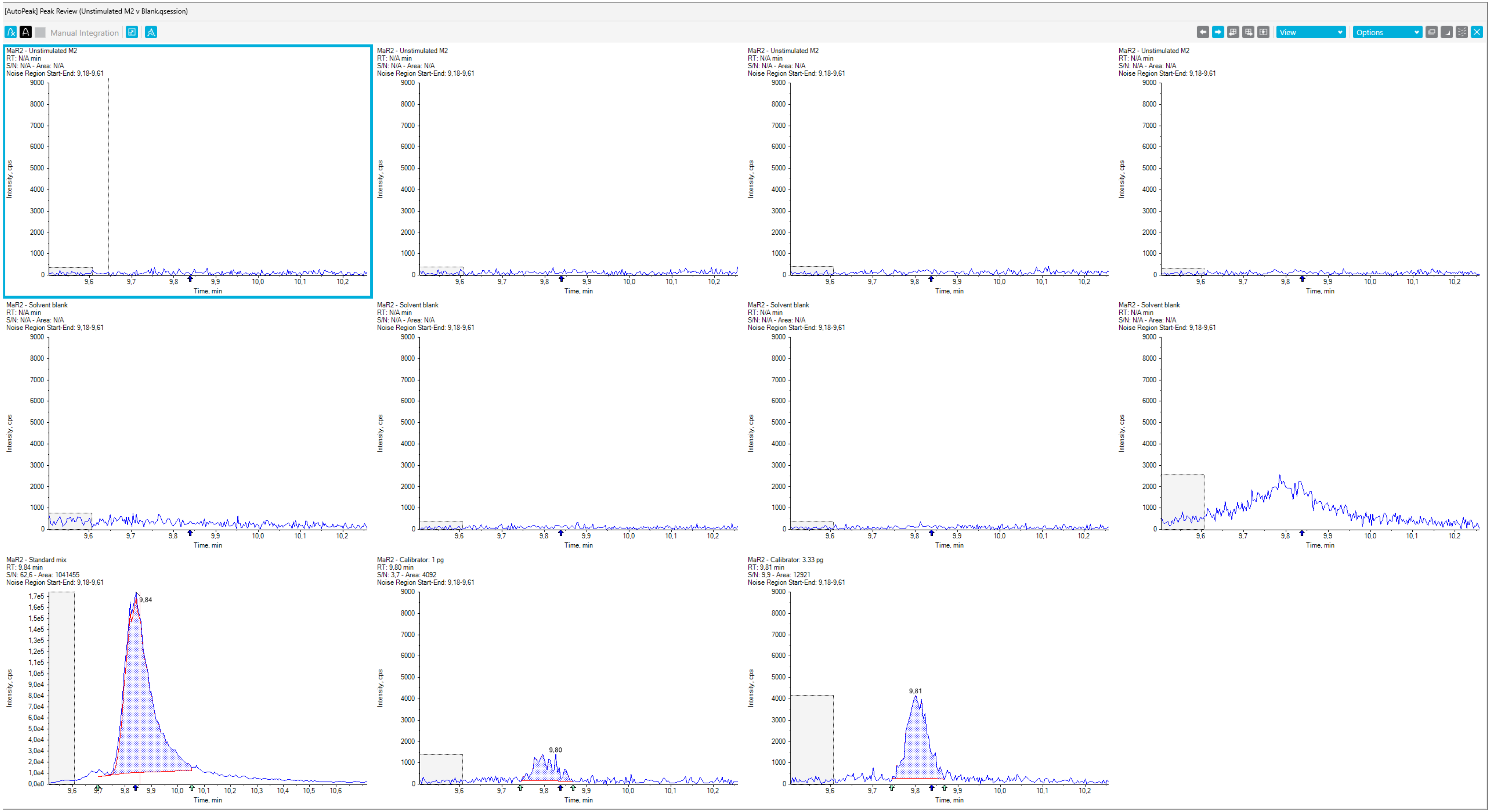
[AutoPeak] Peak Review (Unstimulated M2 v Blank.qsession)
Manual Integration
View
Options
MaR2 - Unstimulated M2
RT: N/A min
S/N: N/A - Area: N/A
Noise Region Start-End: 9,18-9,61
MaR2 - Solvent blank
MaR2 - Standard mix
RT: 9,84 min
S/N: 62,6 - Area: 1041455
MaR2 - Calibrator: 1 pg
RT: 9,80 min
S/N: 3,7 - Area: 4092
MaR2 - Calibrator: 3.33 pg
RT: 9,81 min
S/N: 9,9 - Area: 12921
Intensity, cps
Time, min

7-*epi*-MaR1

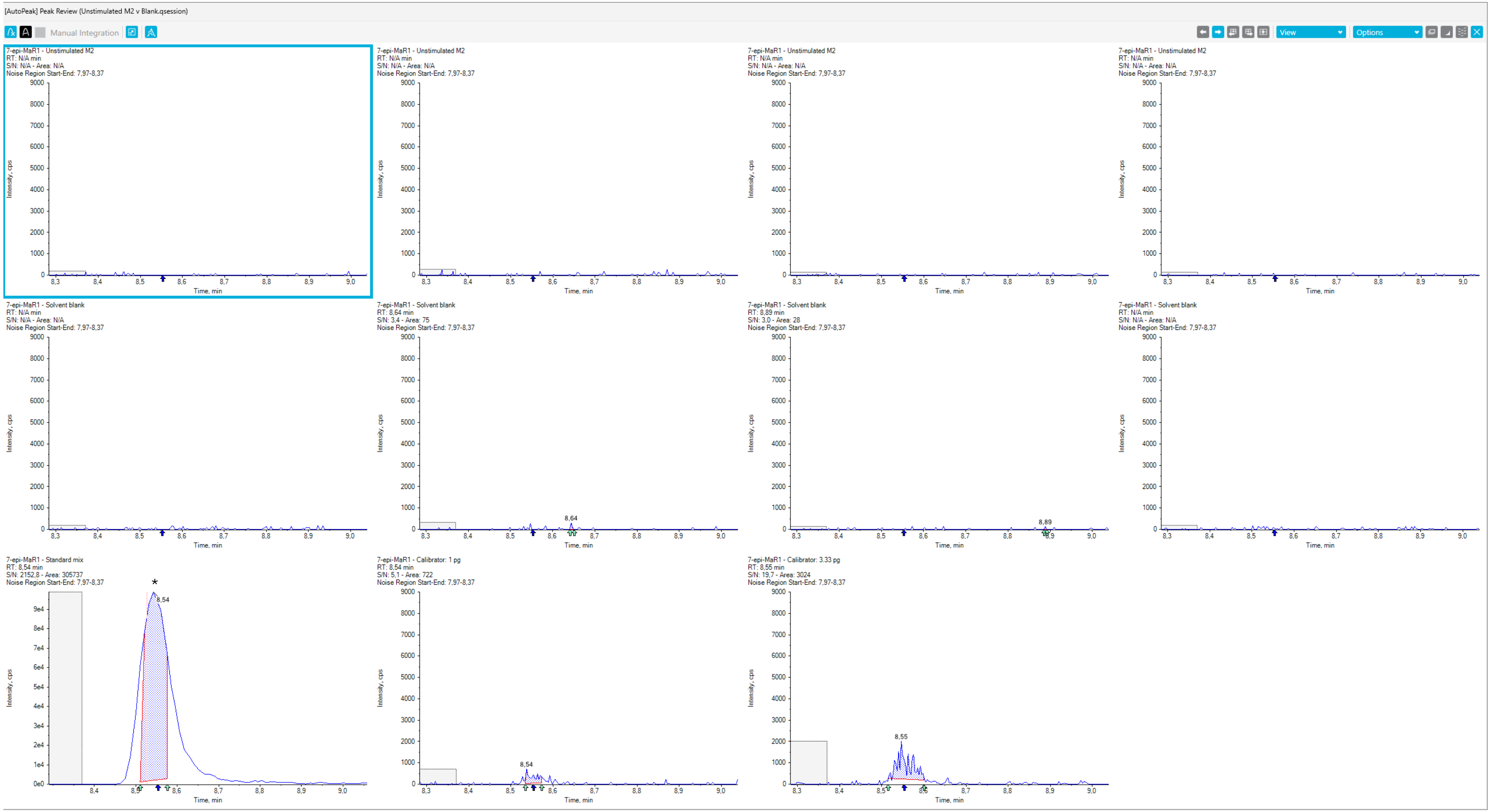

*Standard mix:
Integration indicates acceptable Rt region for peak maximum to warrant identification (“retention time match”), not quantitation

PDX

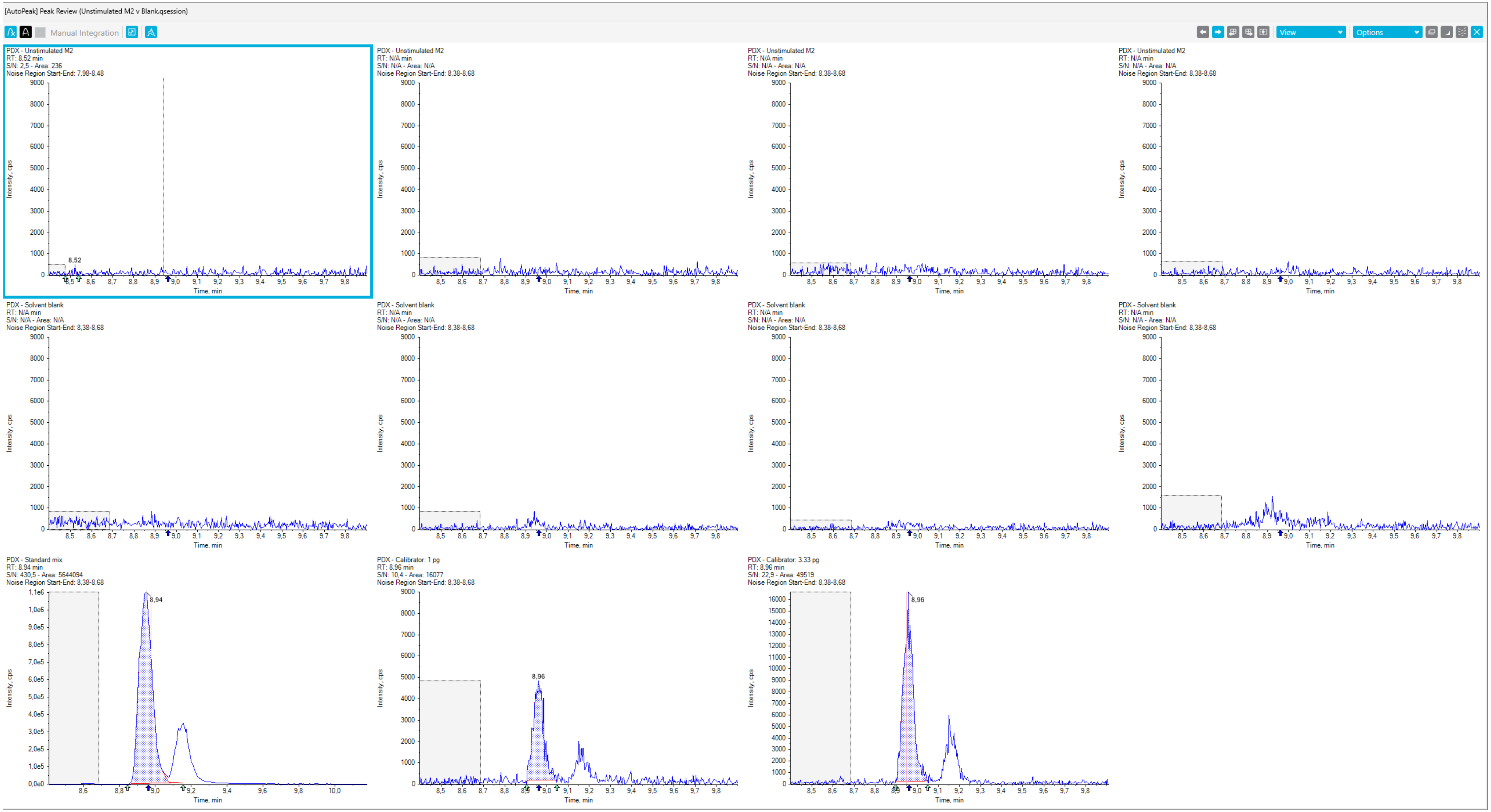
[AutoPeak] Peak Review (Unstimulated M2 v Blank.qsession)
Manual Integration
View
Options
PDX - Unstimulated M2
RT: 8,52 min
S/N: 2,5 - Area: 236
Noise Region Start-End: 7,98-8,48
PDX - Unstimulated M2
RT: N/A min
S/N: N/A - Area: N/A
Noise Region Start-End: 8,38-8,68
PDX - Solvent blank
RT: N/A min
S/N: N/A - Area: N/A
Noise Region Start-End: 8,38-8,68
PDX - Standard mix
RT: 8,94 min
S/N: 430,5 - Area: 5644094
Noise Region Start-End: 8,38-8,68
PDX - Calibrator: 1 pg
RT: 8,96 min
S/N: 10,4 - Area: 16077
Noise Region Start-End: 8,38-8,68
PDX - Calibrator: 3.33 pg
RT: 8,96 min
S/N: 22,9 - Area: 49519
Noise Region Start-End: 8,38-8,68
Intensity, cps
Time, min

PD1

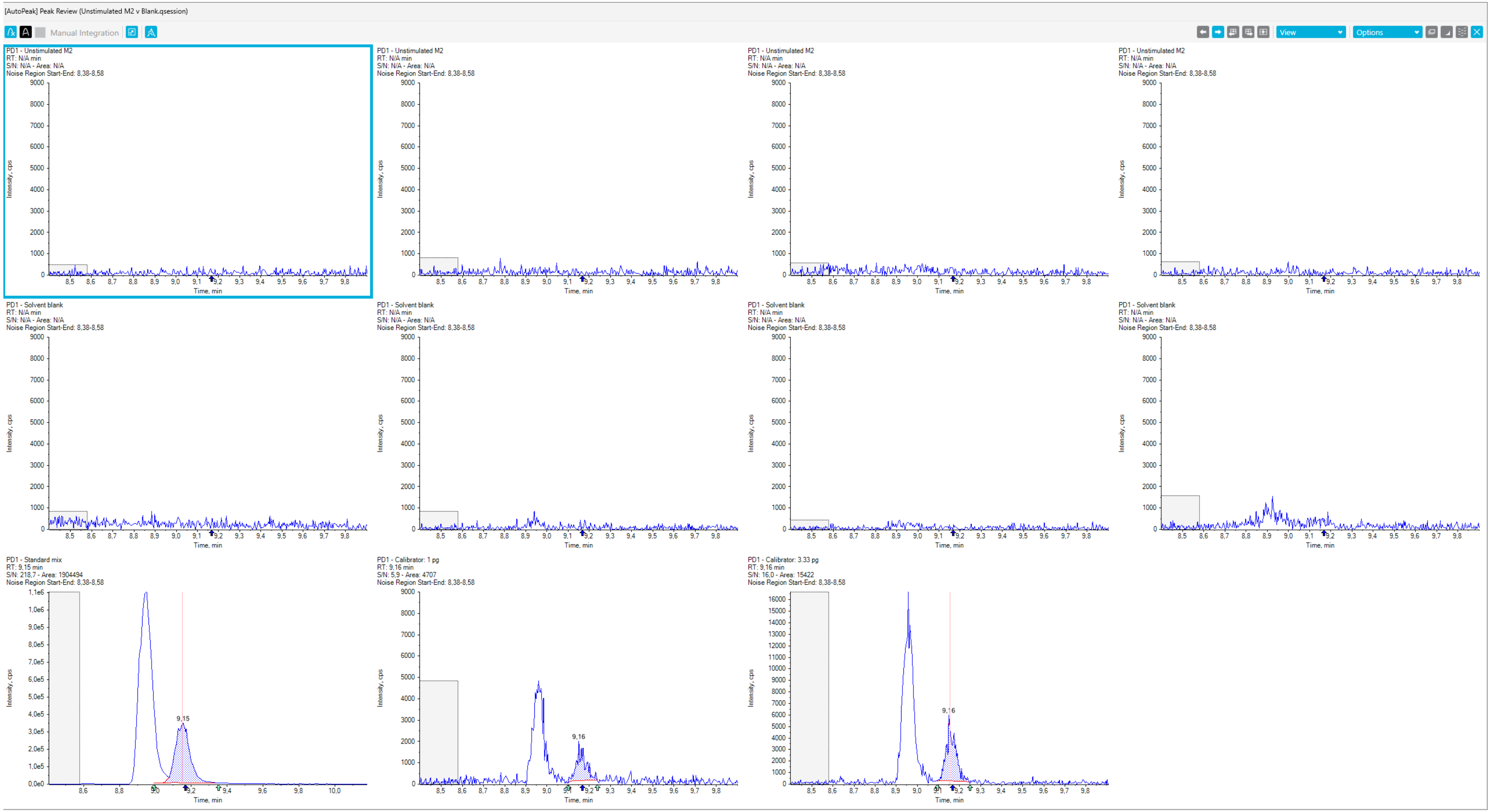
[AutoPeak] Peak Review (Unstimulated M2 v Blank.qsession)
Manual Integration
View
Options
PD1 - Unstimulated M2
RT: N/A min
S/N: N/A - Area: N/A
Noise Region Start-End: 8,38-8,58
PD1 - Solvent blank
RT: N/A min
S/N: N/A - Area: N/A
Noise Region Start-End: 8,38-8,58
PD1 - Standard mix
RT: 9,15 min
S/N: 218,7 - Area: 1904494
Noise Region Start-End: 8,38-8,58
PD1 - Calibrator: 1 pg
RT: 9,16 min
S/N: 5,9 - Area: 4707
Noise Region Start-End: 8,38-8,58
PD1 - Calibrator: 3.33 pg
RT: 9,16 min
S/N: 16,0 - Area: 15422
Noise Region Start-End: 8,38-8,58
Intensity, cps
Time, min

RvD5

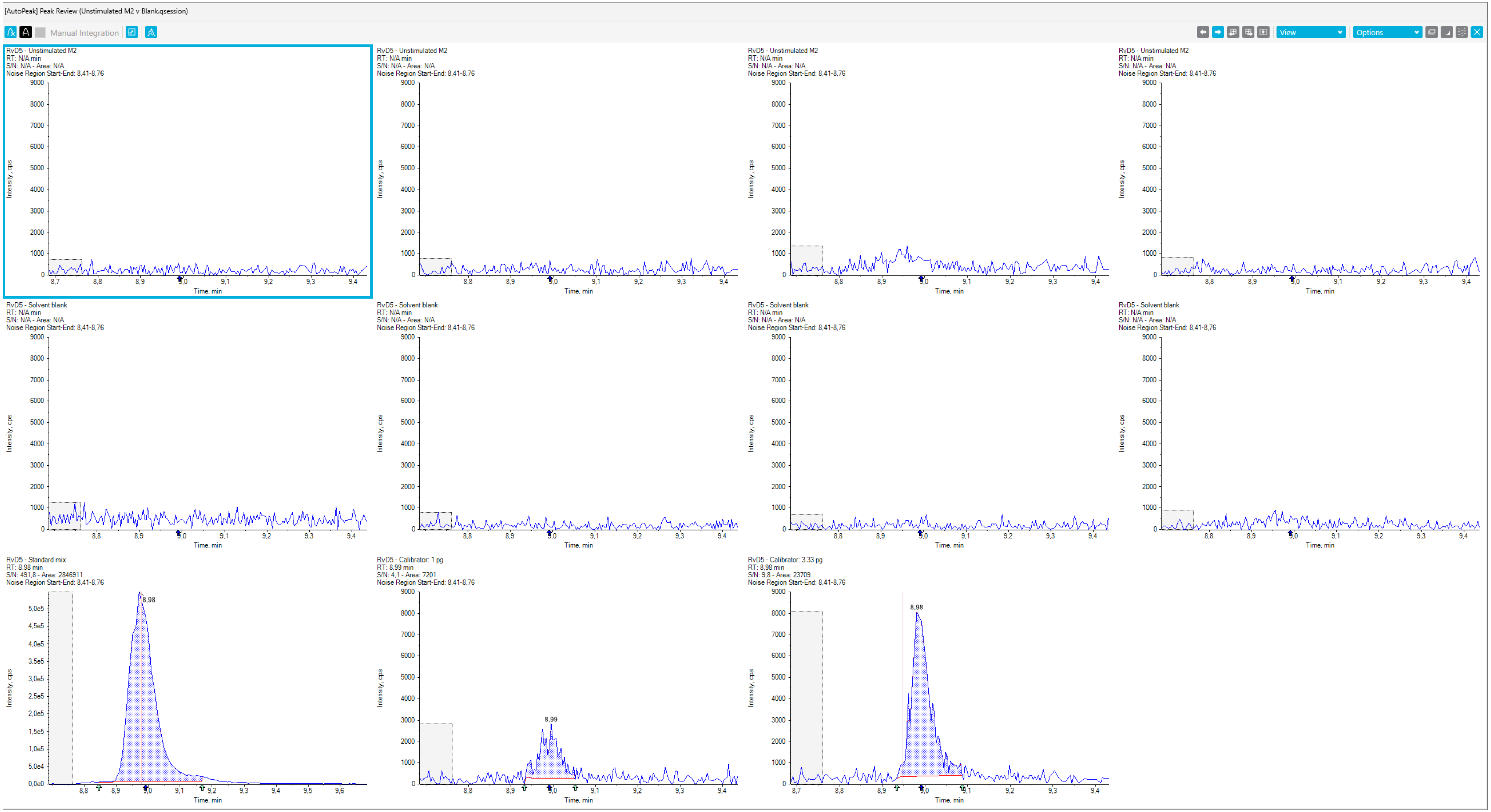
[AutoPeak] Peak Review (Unstimulated M2 v Blank.qsession)
Manual Integration
View
Options
RvD5 - Unstimulated M2
RT: N/A min
S/N: N/A - Area: N/A
Noise Region Start-End: 8,41-8,76
RvD5 - Solvent blank
RT: N/A min
S/N: N/A - Area: N/A
Noise Region Start-End: 8,41-8,76
RvD5 - Standard mix
RT: 8,98 min
S/N: 491,8 - Area: 2846911
Noise Region Start-End: 8,41-8,76
RvD5 - Calibrator: 1 pg
RT: 8,99 min
S/N: 4,1 - Area: 7201
Noise Region Start-End: 8,41-8,76
RvD5 - Calibrator: 3.33 pg
RT: 8,98 min
S/N: 9,8 - Area: 23709
Noise Region Start-End: 8,41-8,76
Intensity, cps
Time, min

RvE1

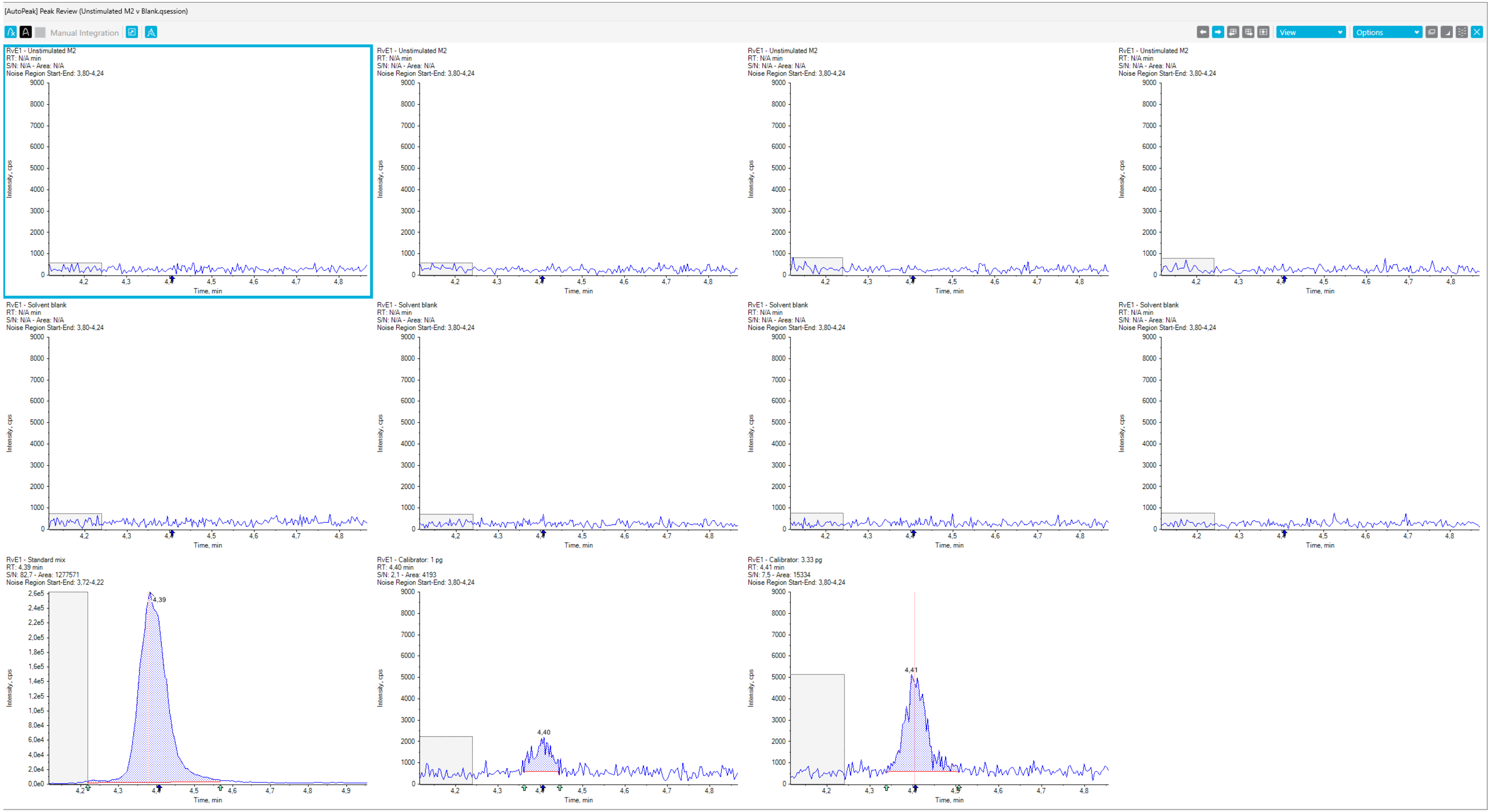
[AutoPeak] Peak Review (Unstimulated M2 v Blank.qsession)
Manual Integration
View
Options
RvE1 - Unstimulated M2
RT: N/A min
S/N: N/A - Area: N/A
Noise Region Start-End: 3,80-4,24
RvE1 - Solvent blank
RT: N/A min
S/N: N/A - Area: N/A
Noise Region Start-End: 3,80-4,24
RvE1 - Standard mix
RT: 4,39 min
S/N: 82,7 - Area: 1277571
Noise Region Start-End: 3,72-4,22
RvE1 - Calibrator: 1 pg
RT: 4,40 min
S/N: 2,1 - Area: 4193
Noise Region Start-End: 3,80-4,24
RvE1 - Calibrator: 3.33 pg
RT: 4,41 min
S/N: 7,5 - Area: 15334
Noise Region Start-End: 3,80-4,24
Intensity, cps
Time, min

RvE2

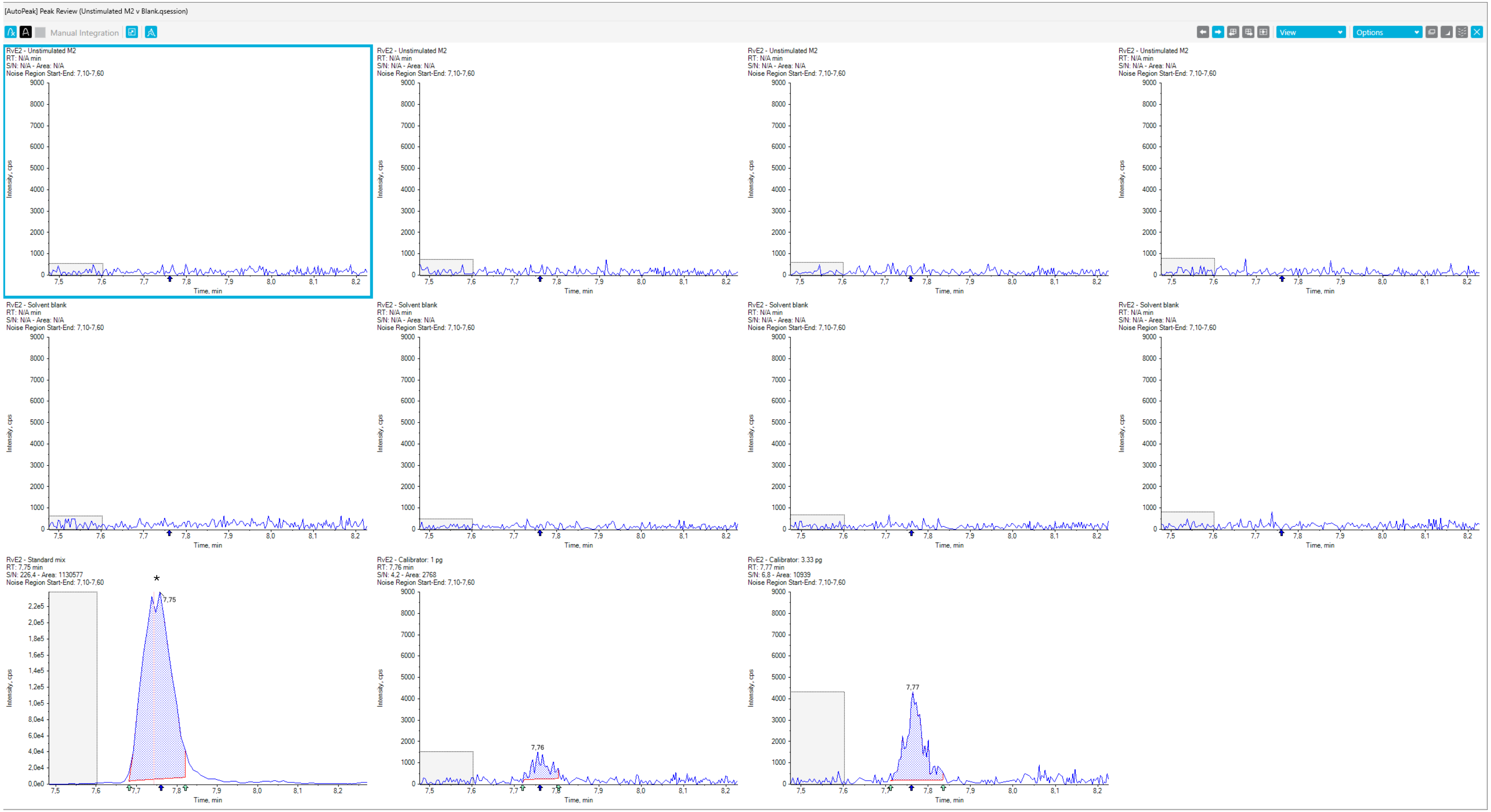

*Standard mix:
Integration indicates acceptable Rt region for peak maximum to warrant identification (“retention time match”), not quantitation

LXA_5

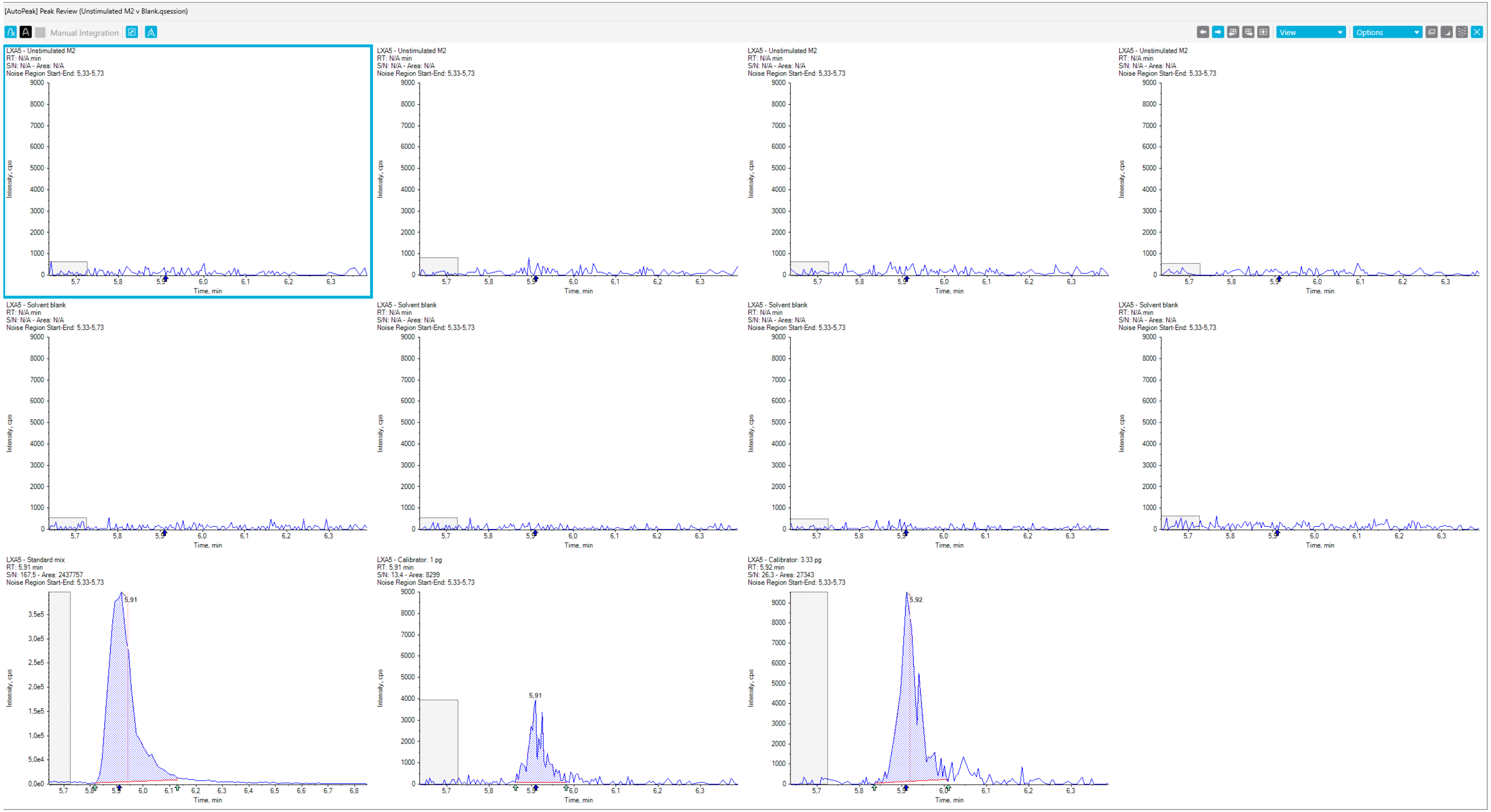
[AutoPeak] Peak Review (Unstimulated M2 v Blank.qsession)
Manual Integration
View
Options
LXA5 - Unstimulated M2
RT: N/A min
S/N: N/A - Area: N/A
Noise Region Start-End: 5,33-5,73
LXA5 - Solvent blank
RT: N/A min
S/N: N/A - Area: N/A
Noise Region Start-End: 5,33-5,73
LXA5 - Standard mix
RT: 5,91 min
S/N: 167,5 - Area: 2437757
Noise Region Start-End: 5,33-5,73
LXA5 - Calibrator: 1 pg
RT: 5,91 min
S/N: 13,4 - Area: 8299
Noise Region Start-End: 5,33-5,73
LXA5 - Calibrator: 3.33 pg
RT: 5,92 min
S/N: 26,3 - Area: 27343
Noise Region Start-End: 5,33-5,73
Intensity, cps
Time, min

RvE4

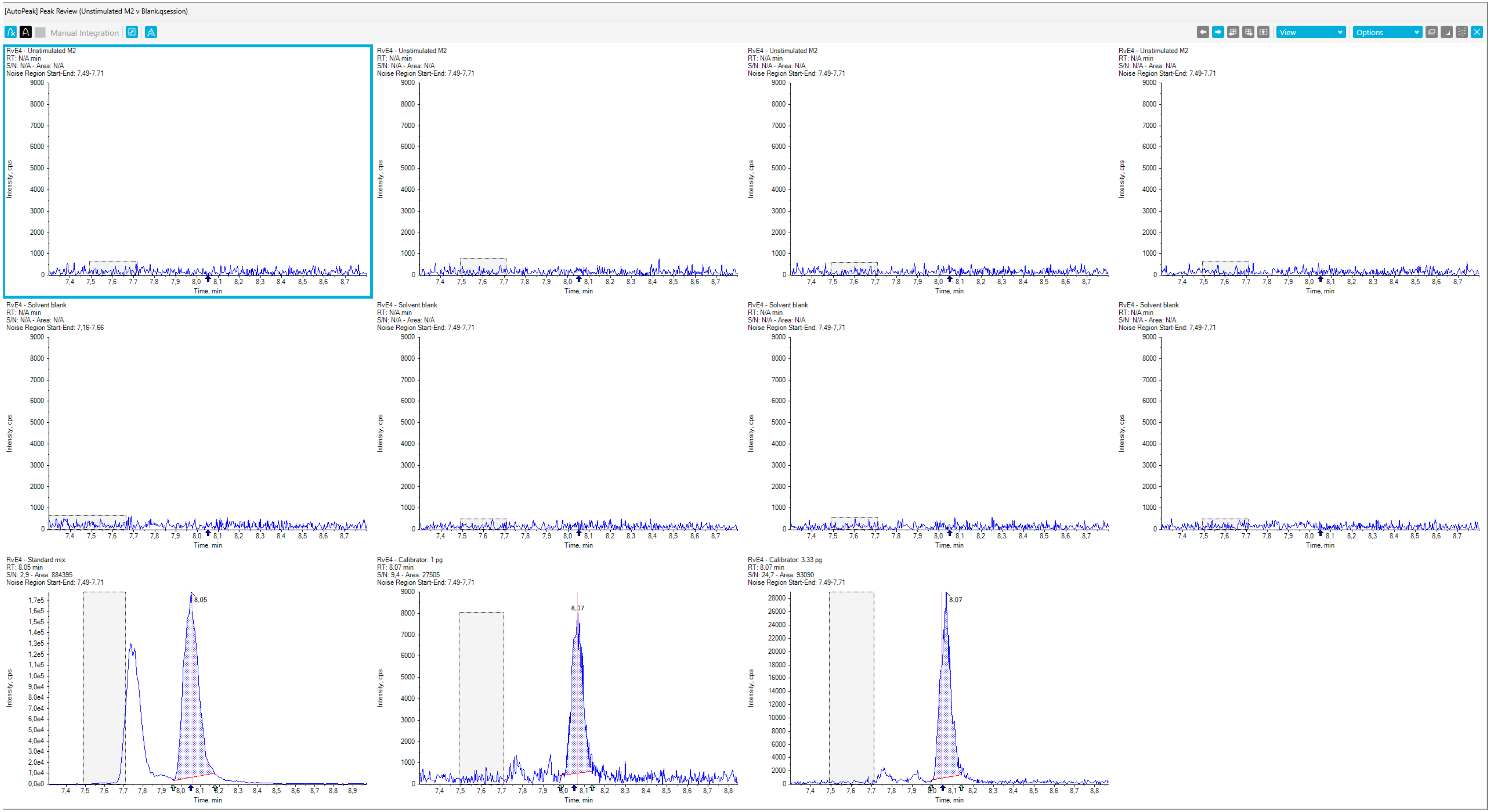

[AutoPeak] Peak Review (Unstimulated M2 v Blank.qsession)
Manual Integration
View
Options
RvE4 - Unstimulated M2
RT: N/A min
S/N: N/A - Area: N/A
Noise Region Start-End: 7,49-7,71
RvE4 - Solvent blank
RT: N/A min
S/N: N/A - Area: N/A
Noise Region Start-End: 7,16-7,66
Noise Region Start-End: 7,49-7,71
RvE4 - Standard mix
RT: 8,05 min
S/N: 2,9 - Area: 884395
Noise Region Start-End: 7,49-7,71
RvE4 - Calibrator: 1 pg
RT: 8,07 min
S/N: 9,4 - Area: 27505
Noise Region Start-End: 7,49-7,71
RvE4 - Calibrator: 3.33 pg
RT: 8,07 min
S/N: 24,7 - Area: 93090
Noise Region Start-End: 7,49-7,71
Intensity, cps
Time, min

Fig. S4: Calibration curves

SPM calibration curves showing slope, *y*-intercept, R^2 value, accuracy, signal-to-noise ratios, and chromatograms.

Legend

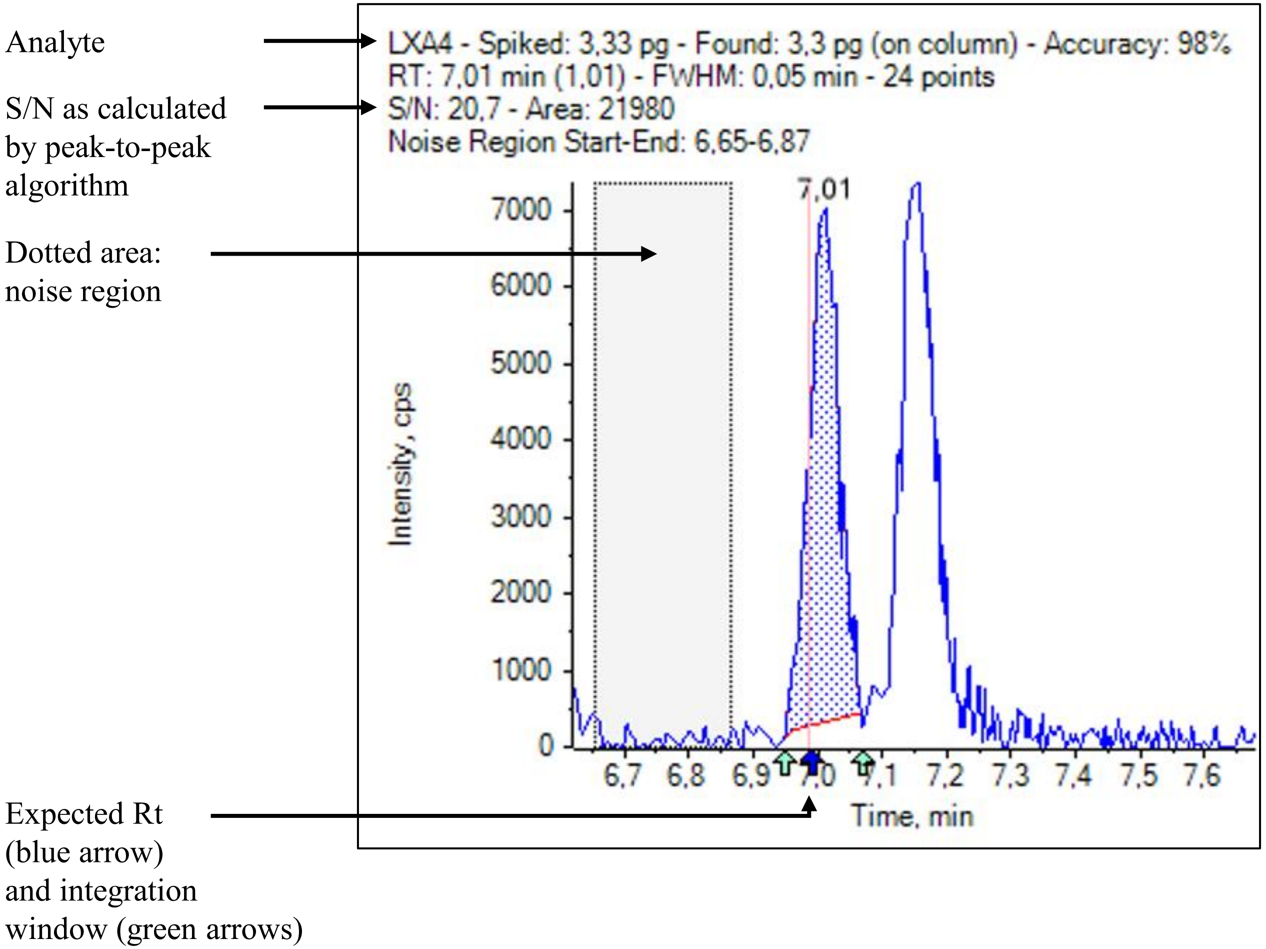

LXA_4

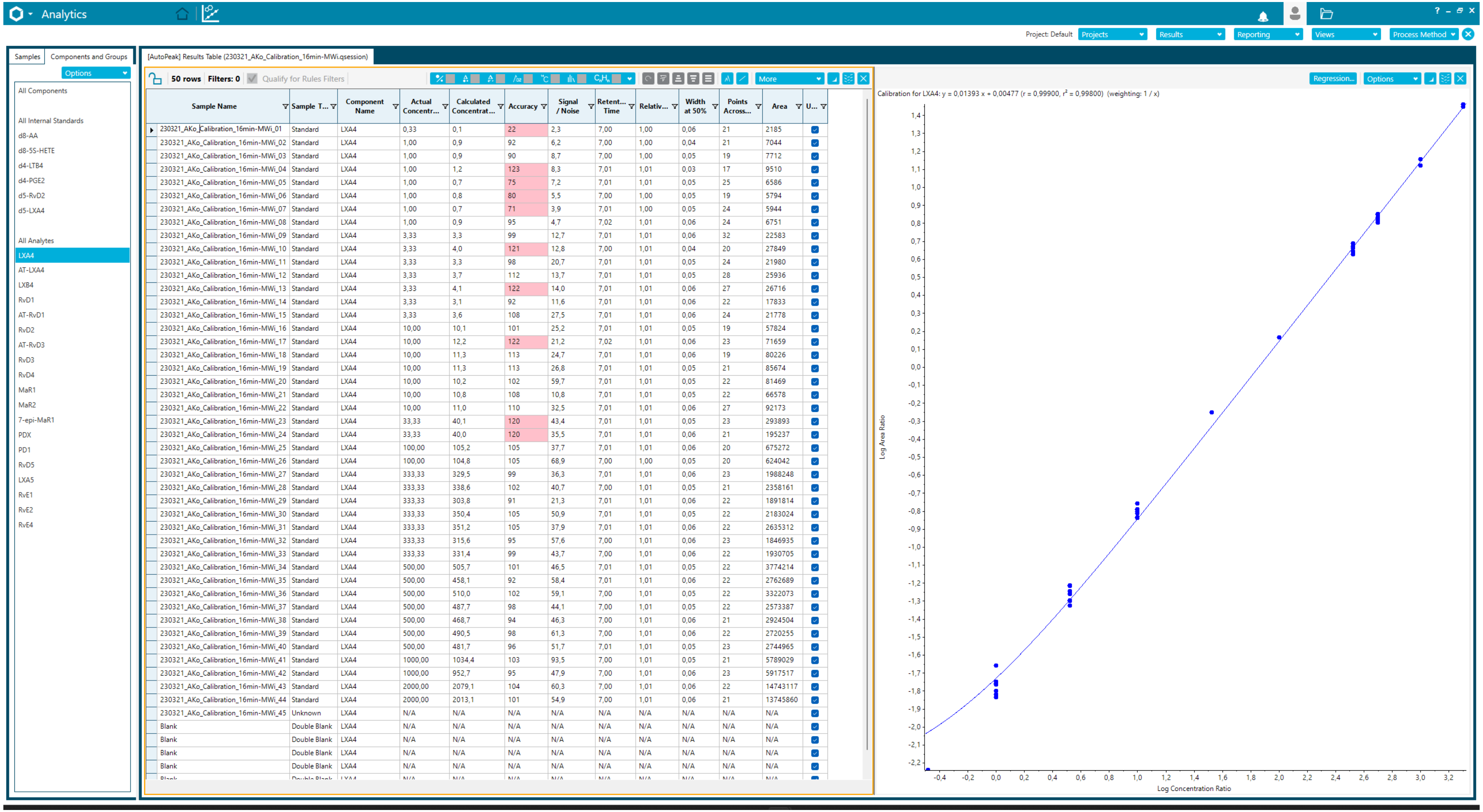

Sample Name	Sample T...	Component Name	Actual Concentr...	Calculated Concentrat...	Accuracy	Signal / Noise	Retent... Time	Relativ...	Width at 50%	Points Across...	Area	U...
230321_AKo_Calibration_16min-MWi_01	Standard	LXA4	0,33	0,1	22	2,3	7,00	1,00	0,06	21	2185	☑
230321_AKo_Calibration_16min-MWi_02	Standard	LXA4	1,00	0,9	92	6,2	7,00	1,00	0,04	21	7044	☑
230321_AKo_Calibration_16min-MWi_03	Standard	LXA4	1,00	0,9	90	8,7	7,00	1,00	0,05	19	7712	☑
230321_AKo_Calibration_16min-MWi_04	Standard	LXA4	1,00	1,2	123	8,3	7,01	1,01	0,03	17	9510	☑
230321_AKo_Calibration_16min-MWi_05	Standard	LXA4	1,00	0,7	75	7,2	7,01	1,01	0,05	25	6586	☑
230321_AKo_Calibration_16min-MWi_06	Standard	LXA4	1,00	0,8	80	5,5	7,00	1,00	0,05	19	5794	☑
230321_AKo_Calibration_16min-MWi_07	Standard	LXA4	1,00	0,7	71	3,9	7,01	1,00	0,05	24	5944	☑
230321_AKo_Calibration_16min-MWi_08	Standard	LXA4	1,00	0,9	95	4,7	7,02	1,01	0,06	24	6751	☑
230321_AKo_Calibration_16min-MWi_09	Standard	LXA4	3,33	3,3	99	12,7	7,01	1,01	0,06	32	22583	☑
230321_AKo_Calibration_16min-MWi_10	Standard	LXA4	3,33	4,0	121	12,8	7,00	1,01	0,04	20	27849	☑
230321_AKo_Calibration_16min-MWi_11	Standard	LXA4	3,33	3,3	98	20,7	7,01	1,01	0,05	24	21980	☑
230321_AKo_Calibration_16min-MWi_12	Standard	LXA4	3,33	3,7	112	13,7	7,01	1,01	0,05	28	25936	☑
230321_AKo_Calibration_16min-MWi_13	Standard	LXA4	3,33	4,1	122	14,0	7,01	1,01	0,06	27	26716	☑
230321_AKo_Calibration_16min-MWi_14	Standard	LXA4	3,33	3,1	92	11,6	7,01	1,01	0,06	22	17833	☑
230321_AKo_Calibration_16min-MWi_15	Standard	LXA4	3,33	3,6	108	27,5	7,01	1,01	0,06	24	21778	☑
230321_AKo_Calibration_16min-MWi_16	Standard	LXA4	10,00	10,1	101	25,2	7,01	1,01	0,05	19	57824	☑
230321_AKo_Calibration_16min-MWi_17	Standard	LXA4	10,00	12,2	122	21,2	7,02	1,01	0,06	23	71659	☑
230321_AKo_Calibration_16min-MWi_18	Standard	LXA4	10,00	11,3	113	24,7	7,01	1,01	0,06	19	80226	☑
230321_AKo_Calibration_16min-MWi_19	Standard	LXA4	10,00	11,3	113	26,8	7,01	1,01	0,05	21	85674	☑
230321_AKo_Calibration_16min-MWi_20	Standard	LXA4	10,00	10,2	102	59,7	7,01	1,01	0,05	22	81469	☑
230321_AKo_Calibration_16min-MWi_21	Standard	LXA4	10,00	10,8	108	10,8	7,01	1,01	0,05	22	66578	☑
230321_AKo_Calibration_16min-MWi_22	Standard	LXA4	10,00	11,0	110	32,5	7,01	1,01	0,06	27	92173	☑
230321_AKo_Calibration_16min-MWi_23	Standard	LXA4	33,33	40,1	120	43,4	7,01	1,01	0,05	23	293893	☑
230321_AKo_Calibration_16min-MWi_24	Standard	LXA4	33,33	40,0	120	35,5	7,01	1,01	0,06	21	195237	☑
230321_AKo_Calibration_16min-MWi_25	Standard	LXA4	100,00	105,2	105	37,7	7,01	1,01	0,06	20	675272	☑
230321_AKo_Calibration_16min-MWi_26	Standard	LXA4	100,00	104,8	105	68,9	7,00	1,00	0,05	20	624042	☑
230321_AKo_Calibration_16min-MWi_27	Standard	LXA4	333,33	329,5	99	36,3	7,01	1,01	0,06	23	1988248	☑
230321_AKo_Calibration_16min-MWi_28	Standard	LXA4	333,33	338,6	102	40,7	7,00	1,01	0,05	21	2358161	☑
230321_AKo_Calibration_16min-MWi_29	Standard	LXA4	333,33	303,8	91	21,3	7,01	1,01	0,06	22	1891814	☑
230321_AKo_Calibration_16min-MWi_30	Standard	LXA4	333,33	350,4	105	50,9	7,01	1,01	0,05	22	2183024	☑
230321_AKo_Calibration_16min-MWi_31	Standard	LXA4	333,33	351,2	105	37,9	7,01	1,01	0,06	22	2635312	☑
230321_AKo_Calibration_16min-MWi_32	Standard	LXA4	333,33	315,6	95	57,6	7,00	1,01	0,06	23	1846935	☑
230321_AKo_Calibration_16min-MWi_33	Standard	LXA4	333,33	331,4	99	43,7	7,00	1,01	0,06	22	1930705	☑
230321_AKo_Calibration_16min-MWi_34	Standard	LXA4	500,00	505,7	101	46,5	7,01	1,01	0,05	22	3774214	☑
230321_AKo_Calibration_16min-MWi_35	Standard	LXA4	500,00	458,1	92	58,4	7,01	1,01	0,06	22	2762689	☑
230321_AKo_Calibration_16min-MWi_36	Standard	LXA4	500,00	510,0	102	59,1	7,00	1,01	0,05	22	3322073	☑
230321_AKo_Calibration_16min-MWi_37	Standard	LXA4	500,00	487,7	98	44,1	7,00	1,01	0,05	22	2573387	☑
230321_AKo_Calibration_16min-MWi_38	Standard	LXA4	500,00	468,7	94	46,3	7,00	1,01	0,06	21	2924504	☑
230321_AKo_Calibration_16min-MWi_39	Standard	LXA4	500,00	490,5	98	61,3	7,00	1,01	0,06	22	2720255	☑
230321_AKo_Calibration_16min-MWi_40	Standard	LXA4	500,00	481,7	96	51,7	7,01	1,01	0,05	23	2744965	☑
230321_AKo_Calibration_16min-MWi_41	Standard	LXA4	1000,00	1034,4	103	93,5	7,00	1,01	0,05	21	5789029	☑
230321_AKo_Calibration_16min-MWi_42	Standard	LXA4	1000,00	952,7	95	47,9	7,00	1,01	0,06	23	5917517	☑
230321_AKo_Calibration_16min-MWi_43	Standard	LXA4	2000,00	2079,1	104	60,3	7,00	1,01	0,06	22	14743117	☑
230321_AKo_Calibration_16min-MWi_44	Standard	LXA4	2000,00	2013,1	101	54,9	7,00	1,01	0,06	21	13745860	☑
230321_AKo_Calibration_16min-MWi_45	Unknown	LXA4	N/A	N/A	N/A	N/A	N/A	N/A	N/A	N/A	N/A	☑
Blank	Double Blank	LXA4	N/A	N/A	N/A	N/A	N/A	N/A	N/A	N/A	N/A	☑
Blank	Double Blank	LXA4	N/A	N/A	N/A	N/A	N/A	N/A	N/A	N/A	N/A	☑
Blank	Double Blank	LXA4	N/A	N/A	N/A	N/A	N/A	N/A	N/A	N/A	N/A	☑
Blank	Double Blank	LXA4	N/A	N/A	N/A	N/A	N/A	N/A	N/A	N/A	N/A	☑
Blank	Double Blank	LXA4	N/A	N/A	N/A	N/A	N/A	N/A	N/A	N/A	N/A	☑

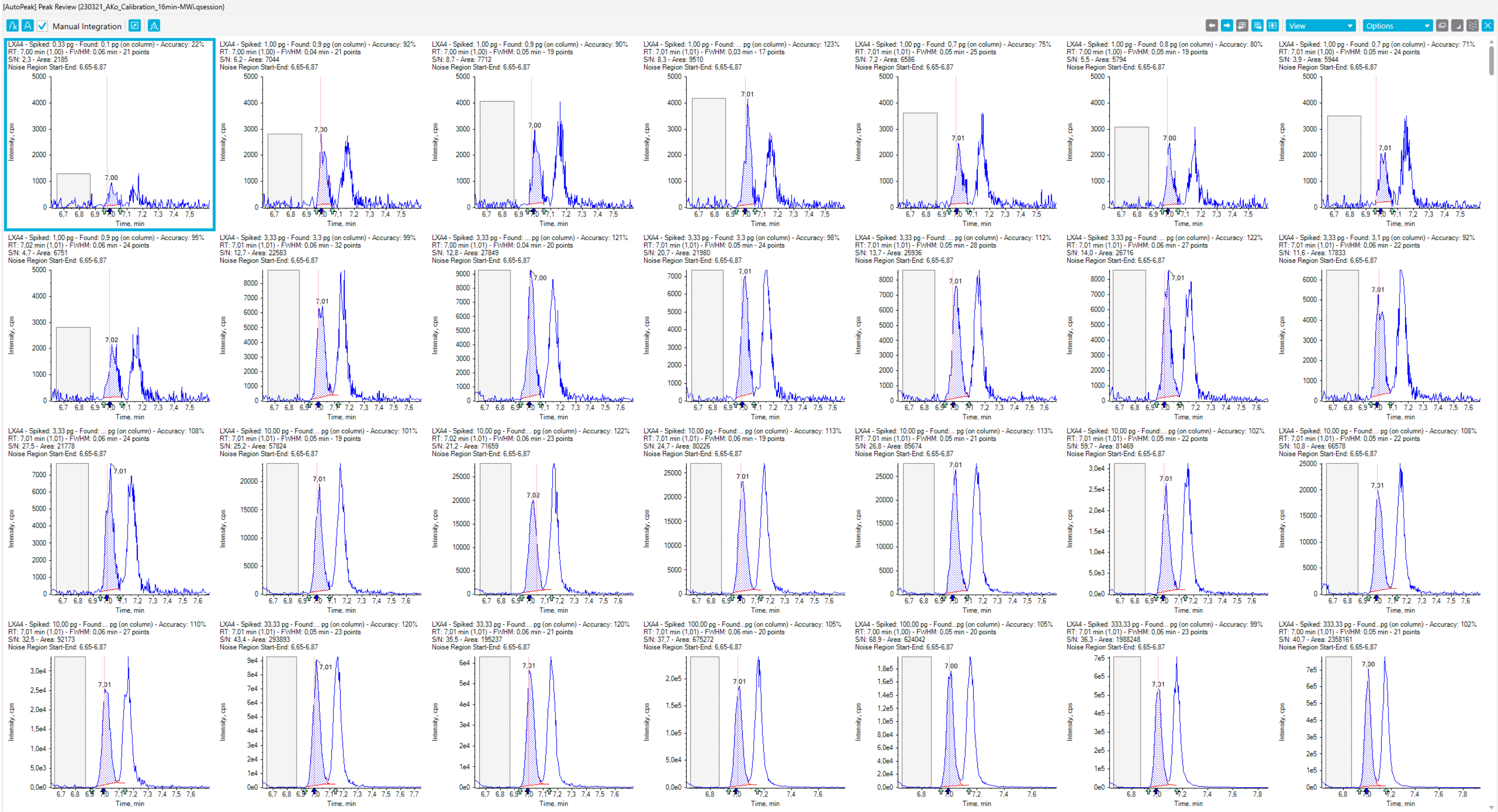
[AutoPeak] Peak Review (230321_AKo_Calibration_16min-MWi.qsession)
Manual Integration
View
Options

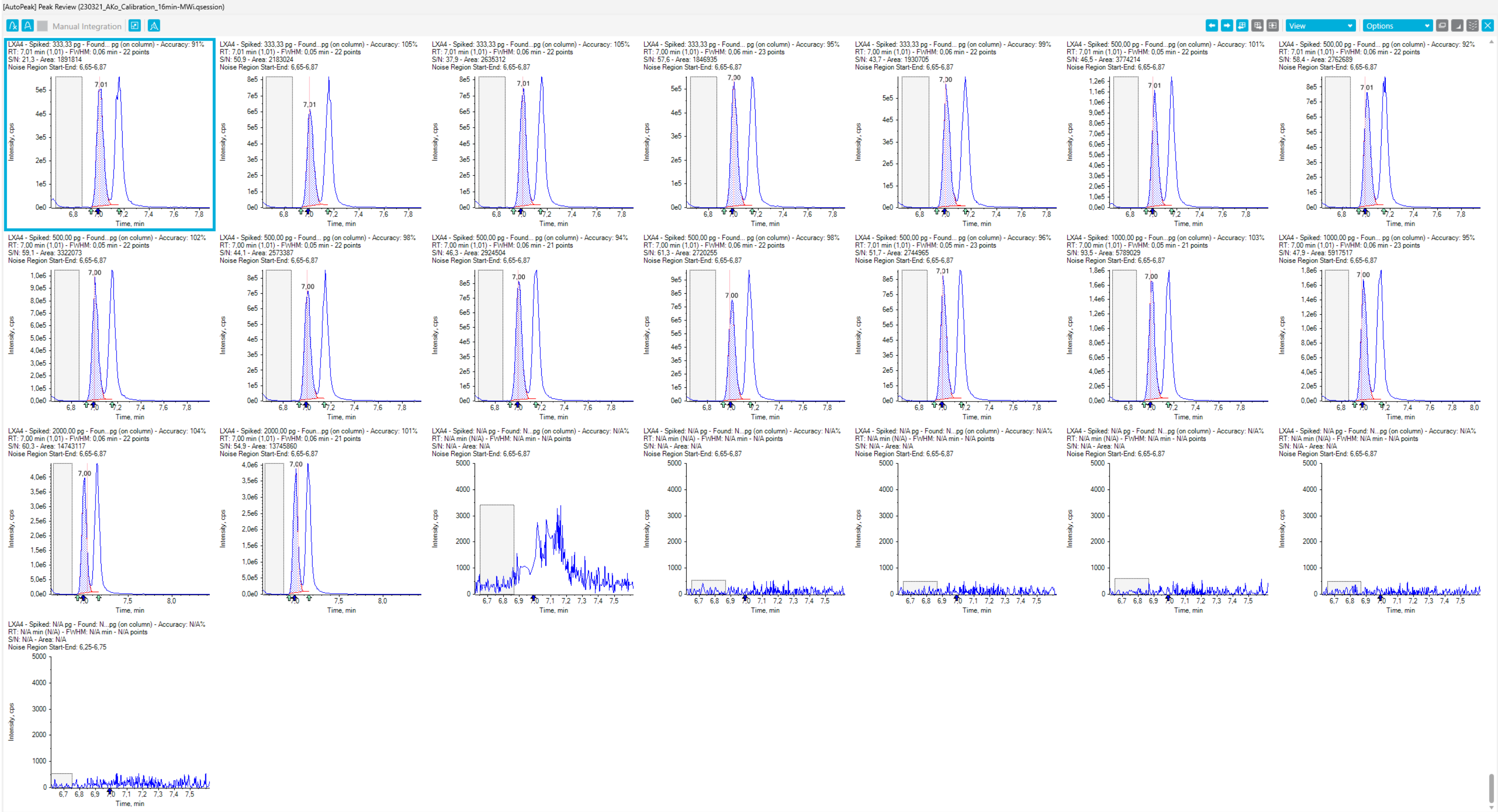
[AutoPeak] Peak Review (230321_AKo_Calibration_16min-MWi.qsession)
Manual Integration
View
Options

AT-LXA$_4$

Analytics

Project: Default | Projects | Results | Reporting | Views | Process Method

Samples | Components and Groups

Options

All Components

All Internal Standards
d8-AA
d8-5S-HETE
d4-LTB4
d4-PGE2
d5-RvD2
d5-LXA4

All Analytes
LXA4
AT-LXA4
LXB4
RvD1
AT-RvD1
RvD2
AT-RvD3
RvD3
RvD4
MaR1
MaR2
7-epi-MaR1
PDX
PD1
RvD5
LXA5
RvE1
RvE2
RvE4

[AutoPeak] Results Table (230321_AKo_Calibration_16min-MWi.qsession)

50 rows | Filters: 0 | Qualify for Rules Filters | More

Sample Name	Sample T...	Component Name	Actual Concentr...	Calculated Concentrat...	Accuracy	Signal / Noise	Retent... Time	Relativ...	Width at 50%	Points Across...	Area	U...
230321_AKo_Calibration_16min-MWi_01	Standard	AT-LXA4	0,33	N/A	N/A	N/A	N/A	N/A	N/A	N/A	N/A	✓
230321_AKo_Calibration_16min-MWi_02	Standard	AT-LXA4	1,00	0,8	82	4,8	7,16	1,03	0,05	41	7510	✓
230321_AKo_Calibration_16min-MWi_03	Standard	AT-LXA4	1,00	0,9	86	11,7	7,16	1,03	0,04	42	8645	✓
230321_AKo_Calibration_16min-MWi_04	Standard	AT-LXA4	1,00	0,8	77	5,5	7,15	1,03	0,05	42	7772	✓
230321_AKo_Calibration_16min-MWi_05	Standard	AT-LXA4	1,00	0,9	91	10,6	7,15	1,03	0,04	42	8732	✓
230321_AKo_Calibration_16min-MWi_06	Standard	AT-LXA4	1,00	0,7	73	6,8	7,16	1,03	0,03	29	6313	✓
230321_AKo_Calibration_16min-MWi_07	Standard	AT-LXA4	1,00	1,0	104	6,9	7,15	1,03	0,05	50	9052	✓
230321_AKo_Calibration_16min-MWi_08	Standard	AT-LXA4	1,00	0,8	80	5,4	7,16	1,03	0,05	32	6947	✓
230321_AKo_Calibration_16min-MWi_09	Standard	AT-LXA4	3,33	3,3	98	22,7	7,16	1,03	0,05	52	25995	✓
230321_AKo_Calibration_16min-MWi_10	Standard	AT-LXA4	3,33	3,2	96	11,5	7,15	1,03	0,06	30	26163	✓
230321_AKo_Calibration_16min-MWi_11	Standard	AT-LXA4	3,33	3,1	93	15,9	7,16	1,03	0,06	40	24163	✓
230321_AKo_Calibration_16min-MWi_12	Standard	AT-LXA4	3,33	3,5	105	11,9	7,16	1,03	0,06	44	28278	✓
230321_AKo_Calibration_16min-MWi_13	Standard	AT-LXA4	3,33	3,3	100	8,1	7,16	1,03	0,06	39	25776	✓
230321_AKo_Calibration_16min-MWi_14	Standard	AT-LXA4	3,33	3,3	98	12,6	7,15	1,03	0,06	52	21942	✓
230321_AKo_Calibration_16min-MWi_15	Standard	AT-LXA4	3,33	3,3	99	20,3	7,16	1,03	0,06	37	23305	✓
230321_AKo_Calibration_16min-MWi_16	Standard	AT-LXA4	10,00	11,1	111	18,1	7,16	1,03	0,05	59	73176	✓
230321_AKo_Calibration_16min-MWi_17	Standard	AT-LXA4	10,00	13,1	131	25,0	7,17	1,03	0,05	49	89456	✓
230321_AKo_Calibration_16min-MWi_18	Standard	AT-LXA4	10,00	10,8	108	24,1	7,16	1,03	0,05	49	89008	✓
230321_AKo_Calibration_16min-MWi_19	Standard	AT-LXA4	10,00	11,1	111	19,9	7,16	1,03	0,06	41	97806	✓
230321_AKo_Calibration_16min-MWi_20	Standard	AT-LXA4	10,00	11,4	114	23,3	7,16	1,03	0,06	39	105163	✓
230321_AKo_Calibration_16min-MWi_21	Standard	AT-LXA4	10,00	10,8	108	11,4	7,16	1,03	0,05	36	77336	✓
230321_AKo_Calibration_16min-MWi_22	Standard	AT-LXA4	10,00	10,7	107	27,5	7,16	1,03	0,05	42	104123	✓
230321_AKo_Calibration_16min-MWi_23	Standard	AT-LXA4	33,33	39,2	118	28,3	7,16	1,03	0,06	48	334184	✓
230321_AKo_Calibration_16min-MWi_24	Standard	AT-LXA4	33,33	36,5	110	20,1	7,16	1,03	0,05	41	207722	✓
230321_AKo_Calibration_16min-MWi_25	Standard	AT-LXA4	100,00	111,7	112	25,1	7,16	1,03	0,05	42	834745	✓
230321_AKo_Calibration_16min-MWi_26	Standard	AT-LXA4	100,00	105,9	106	25,2	7,16	1,03	0,06	44	733673	✓
230321_AKo_Calibration_16min-MWi_27	Standard	AT-LXA4	333,33	335,1	101	32,4	7,16	1,03	0,05	39	2353346	✓
230321_AKo_Calibration_16min-MWi_28	Standard	AT-LXA4	333,33	335,7	101	29,9	7,15	1,03	0,05	36	2720152	✓
230321_AKo_Calibration_16min-MWi_29	Standard	AT-LXA4	333,33	295,0	89	14,4	7,16	1,03	0,06	36	2138520	✓
230321_AKo_Calibration_16min-MWi_30	Standard	AT-LXA4	333,33	383,2	115	25,4	7,16	1,03	0,05	35	2778075	✓
230321_AKo_Calibration_16min-MWi_31	Standard	AT-LXA4	333,33	334,2	100	22,9	7,16	1,03	0,06	35	2918535	✓
230321_AKo_Calibration_16min-MWi_32	Standard	AT-LXA4	333,33	303,1	91	30,7	7,15	1,03	0,06	36	2064356	✓
230321_AKo_Calibration_16min-MWi_33	Standard	AT-LXA4	333,33	328,3	98	33,0	7,16	1,03	0,06	36	2225683	✓
230321_AKo_Calibration_16min-MWi_34	Standard	AT-LXA4	500,00	499,3	100	30,8	7,16	1,03	0,05	36	4336642	✓
230321_AKo_Calibration_16min-MWi_35	Standard	AT-LXA4	500,00	458,1	92	28,1	7,16	1,03	0,06	36	3215040	✓
230321_AKo_Calibration_16min-MWi_36	Standard	AT-LXA4	500,00	510,1	102	34,4	7,15	1,03	0,06	36	3866780	✓
230321_AKo_Calibration_16min-MWi_37	Standard	AT-LXA4	500,00	473,2	95	20,6	7,15	1,03	0,05	35	2906325	✓
230321_AKo_Calibration_16min-MWi_38	Standard	AT-LXA4	500,00	487,3	97	24,6	7,15	1,03	0,06	36	3538219	✓
230321_AKo_Calibration_16min-MWi_39	Standard	AT-LXA4	500,00	529,4	106	32,6	7,15	1,03	0,06	35	3416954	✓
230321_AKo_Calibration_16min-MWi_40	Standard	AT-LXA4	500,00	490,9	98	25,5	7,15	1,03	0,06	34	3255371	✓
230321_AKo_Calibration_16min-MWi_41	Standard	AT-LXA4	1000,00	1010,2	101	33,8	7,15	1,03	0,05	32	6578941	✓
230321_AKo_Calibration_16min-MWi_42	Standard	AT-LXA4	1000,00	975,6	98	26,8	7,15	1,03	0,06	34	7052400	✓
230321_AKo_Calibration_16min-MWi_43	Standard	AT-LXA4	2000,00	2074,9	104	26,0	7,15	1,03	0,06	35	17122225	✓
230321_AKo_Calibration_16min-MWi_44	Standard	AT-LXA4	2000,00	1975,7	99	31,3	7,15	1,03	0,06	34	15699501	✓
230321_AKo_Calibration_16min-MWi_45	Unknown	AT-LXA4	N/A	N/A	N/A	N/A	N/A	N/A	N/A	N/A	N/A	✓
Blank	Double Blank	AT-LXA4	N/A	N/A	N/A	N/A	N/A	N/A	N/A	N/A	N/A	✓
Blank	Double Blank	AT-LXA4	N/A	N/A	N/A	N/A	N/A	N/A	N/A	N/A	N/A	✓
Blank	Double Blank	AT-LXA4	N/A	N/A	N/A	N/A	N/A	N/A	N/A	N/A	N/A	✓
Blank	Double Blank	AT-LXA4	N/A	N/A	N/A	N/A	N/A	N/A	N/A	N/A	N/A	✓
Blank	Double Blank	AT-LXA4	N/A	N/A	N/A	N/A	N/A	N/A	N/A	N/A	N/A	✓

Regression... | Options

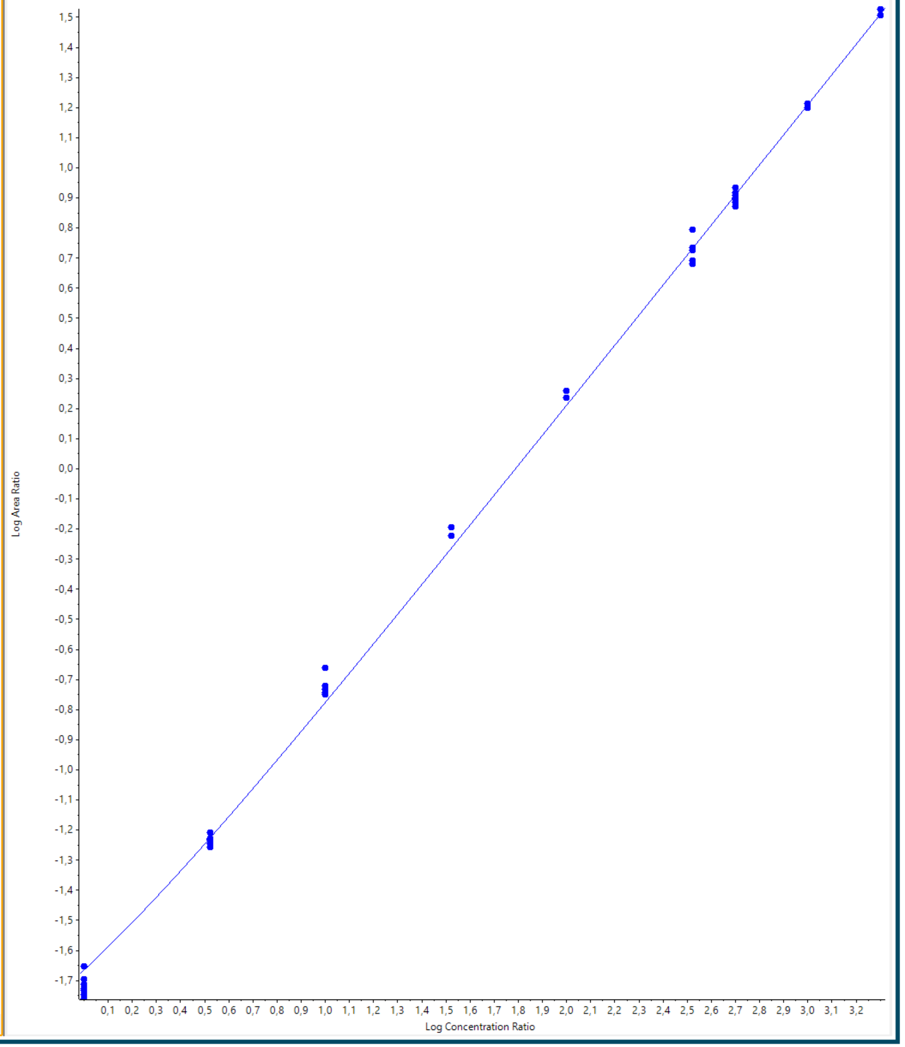

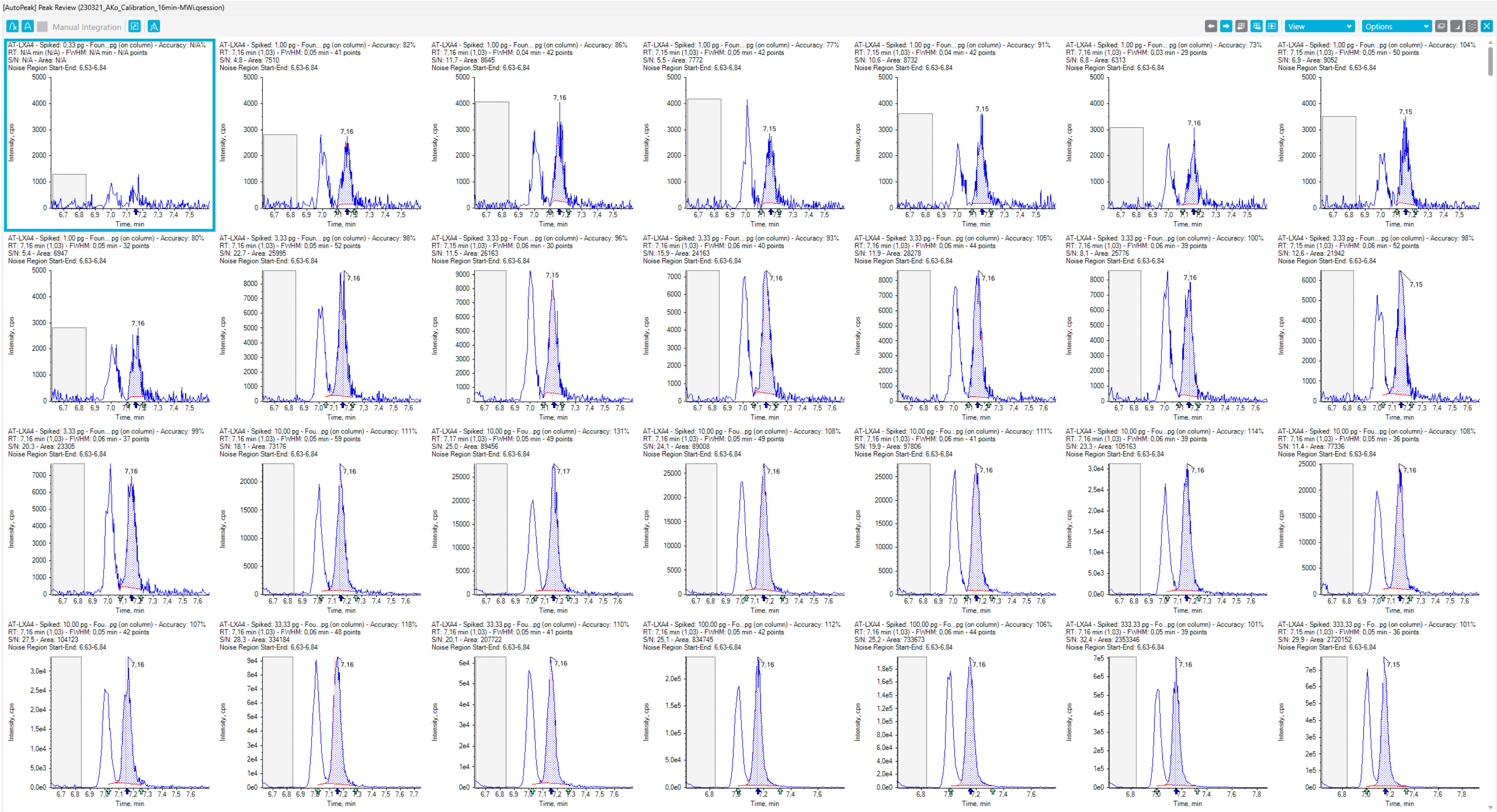
[AutoPeak] Peak Review (230321_AKo_Calibration_16min-MWi.qsession)
Manual Integration
View
Options

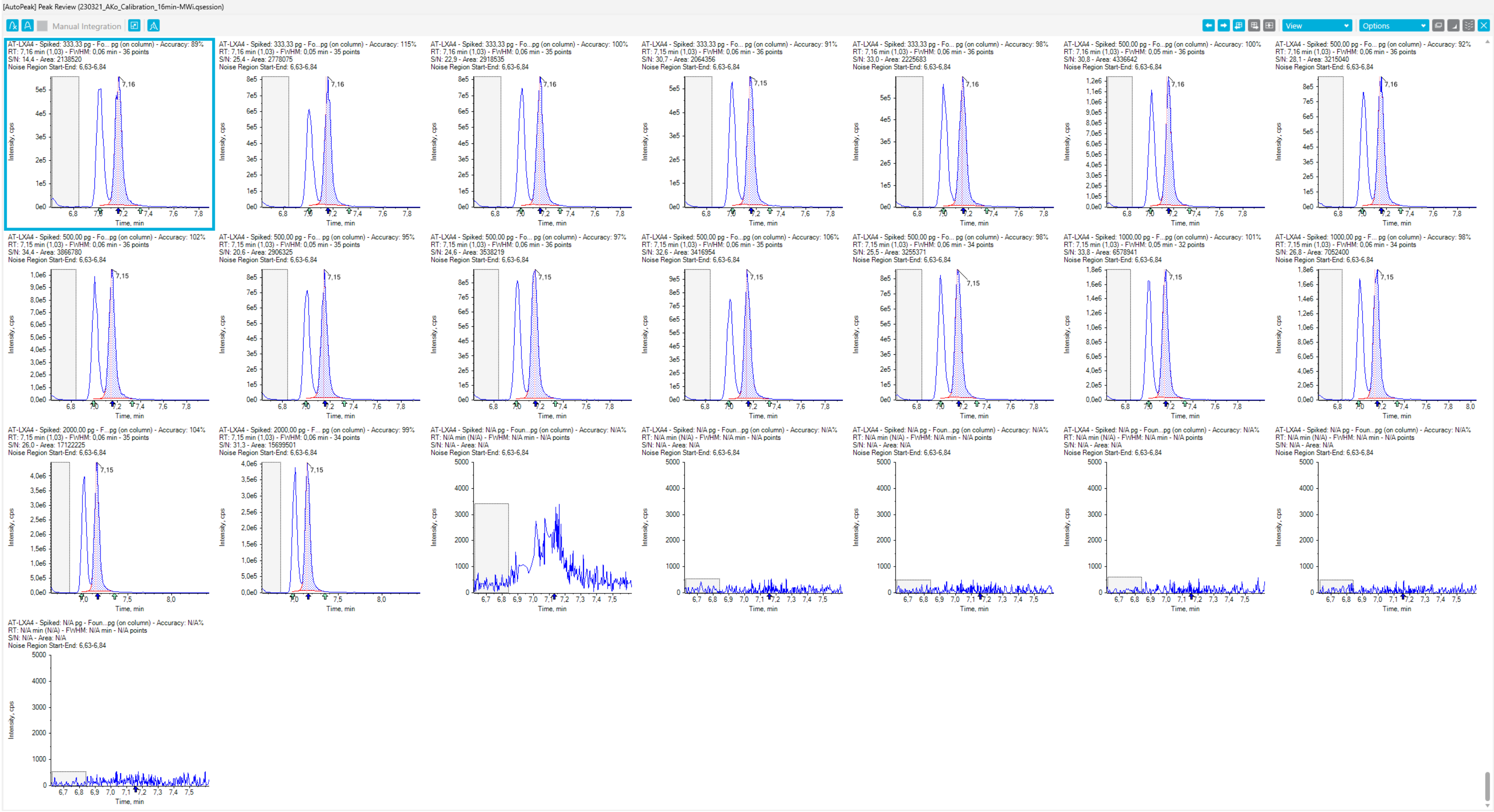
[AutoPeak] Peak Review (230321_AKo_Calibration_16min-MWi.qsession)
Manual Integration
View
Options
AT-LXA4 - Spiked: 333,33 pg - Fo... pg (on column) - Accuracy: 89%
RT: 7,16 min (1,03) - FWHM: 0,06 min - 36 points
S/N: 14,4 - Area: 2138520
Noise Region Start-End: 6,63-6,84
AT-LXA4 - Spiked: 333,33 pg - Fo...pg (on column) - Accuracy: 115%
RT: 7,16 min (1,03) - FWHM: 0,05 min - 35 points
S/N: 25,4 - Area: 2778075
AT-LXA4 - Spiked: 333,33 pg - Fo...pg (on column) - Accuracy: 100%
RT: 7,16 min (1,03) - FWHM: 0,06 min - 35 points
S/N: 22,9 - Area: 2918535
AT-LXA4 - Spiked: 333,33 pg - Fo... pg (on column) - Accuracy: 91%
RT: 7,15 min (1,03) - FWHM: 0,06 min - 36 points
S/N: 30,7 - Area: 2064356
AT-LXA4 - Spiked: 333,33 pg - Fo... pg (on column) - Accuracy: 98%
RT: 7,16 min (1,03) - FWHM: 0,06 min - 36 points
S/N: 33,0 - Area: 2225683
AT-LXA4 - Spiked: 500,00 pg - Fo...pg (on column) - Accuracy: 100%
RT: 7,16 min (1,03) - FWHM: 0,05 min - 36 points
S/N: 30,8 - Area: 4336642
AT-LXA4 - Spiked: 500,00 pg - Fo... pg (on column) - Accuracy: 92%
RT: 7,16 min (1,03) - FWHM: 0,06 min - 36 points
S/N: 28,1 - Area: 3215040
AT-LXA4 - Spiked: 500,00 pg - Fo...pg (on column) - Accuracy: 102%
RT: 7,15 min (1,03) - FWHM: 0,06 min - 36 points
S/N: 34,4 - Area: 3866780
AT-LXA4 - Spiked: 500,00 pg - Fo...pg (on column) - Accuracy: 95%
RT: 7,15 min (1,03) - FWHM: 0,05 min - 35 points
S/N: 20,6 - Area: 2906325
AT-LXA4 - Spiked: 500,00 pg - Fo... pg (on column) - Accuracy: 97%
RT: 7,15 min (1,03) - FWHM: 0,06 min - 36 points
S/N: 24,6 - Area: 3538219
AT-LXA4 - Spiked: 500,00 pg - Fo...pg (on column) - Accuracy: 106%
RT: 7,15 min (1,03) - FWHM: 0,06 min - 35 points
S/N: 32,6 - Area: 3416954
AT-LXA4 - Spiked: 500,00 pg - Fo...pg (on column) - Accuracy: 98%
RT: 7,15 min (1,03) - FWHM: 0,06 min - 34 points
S/N: 25,5 - Area: 3255371
AT-LXA4 - Spiked: 1000,00 pg - F...pg (on column) - Accuracy: 101%
RT: 7,15 min (1,03) - FWHM: 0,05 min - 32 points
S/N: 33,8 - Area: 6578941
AT-LXA4 - Spiked: 1000,00 pg - F... pg (on column) - Accuracy: 98%
RT: 7,15 min (1,03) - FWHM: 0,06 min - 34 points
S/N: 26,8 - Area: 7052400
AT-LXA4 - Spiked: 2000,00 pg - F...pg (on column) - Accuracy: 104%
RT: 7,15 min (1,03) - FWHM: 0,06 min - 35 points
S/N: 26,0 - Area: 17122225
AT-LXA4 - Spiked: 2000,00 pg - F... pg (on column) - Accuracy: 99%
RT: 7,15 min (1,03) - FWHM: 0,06 min - 34 points
S/N: 31,3 - Area: 15699501
AT-LXA4 - Spiked: N/A pg - Foun...pg (on column) - Accuracy: N/A%
RT: N/A min (N/A) - FWHM: N/A min - N/A points
S/N: N/A - Area: N/A
Intensity, cps
Time, min

LXB$_4$

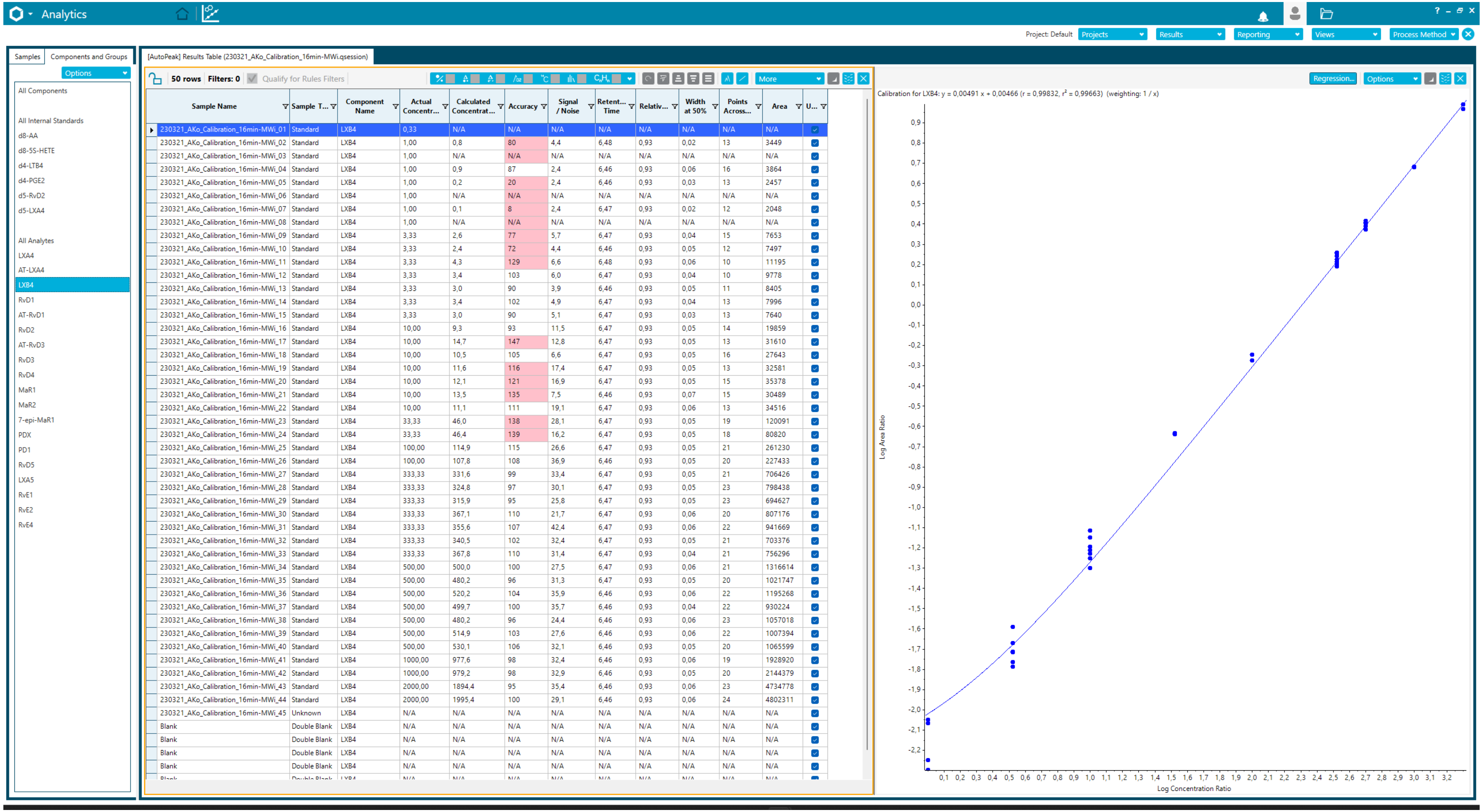

Sample Name	Sample T...	Component Name	Actual Concentr...	Calculated Concentrat...	Accuracy	Signal / Noise	Retent... Time	Relativ...	Width at 50%	Points Across...	Area	U...
230321_AKo_Calibration_16min-MWi_01	Standard	LXB4	0,33	N/A	N/A	N/A	N/A	N/A	N/A	N/A	N/A	☑
230321_AKo_Calibration_16min-MWi_02	Standard	LXB4	1,00	0,8	80	4,4	6,48	0,93	0,02	13	3449	☑
230321_AKo_Calibration_16min-MWi_03	Standard	LXB4	1,00	N/A	N/A	N/A	N/A	N/A	N/A	N/A	N/A	☑
230321_AKo_Calibration_16min-MWi_04	Standard	LXB4	1,00	0,9	87	2,4	6,46	0,93	0,06	16	3864	☑
230321_AKo_Calibration_16min-MWi_05	Standard	LXB4	1,00	0,2	20	2,4	6,46	0,93	0,03	13	2457	☑
230321_AKo_Calibration_16min-MWi_06	Standard	LXB4	1,00	N/A	N/A	N/A	N/A	N/A	N/A	N/A	N/A	☑
230321_AKo_Calibration_16min-MWi_07	Standard	LXB4	1,00	0,1	8	2,4	6,47	0,93	0,02	12	2048	☑
230321_AKo_Calibration_16min-MWi_08	Standard	LXB4	1,00	N/A	N/A	N/A	N/A	N/A	N/A	N/A	N/A	☑
230321_AKo_Calibration_16min-MWi_09	Standard	LXB4	3,33	2,6	77	5,7	6,47	0,93	0,04	15	7653	☑
230321_AKo_Calibration_16min-MWi_10	Standard	LXB4	3,33	2,4	72	4,4	6,46	0,93	0,05	12	7497	☑
230321_AKo_Calibration_16min-MWi_11	Standard	LXB4	3,33	4,3	129	6,6	6,48	0,93	0,06	10	11195	☑
230321_AKo_Calibration_16min-MWi_12	Standard	LXB4	3,33	3,4	103	6,0	6,47	0,93	0,04	10	9778	☑
230321_AKo_Calibration_16min-MWi_13	Standard	LXB4	3,33	3,0	90	3,9	6,46	0,93	0,05	11	8405	☑
230321_AKo_Calibration_16min-MWi_14	Standard	LXB4	3,33	3,4	102	4,9	6,47	0,93	0,04	13	7996	☑
230321_AKo_Calibration_16min-MWi_15	Standard	LXB4	3,33	3,0	90	5,1	6,47	0,93	0,03	13	7640	☑
230321_AKo_Calibration_16min-MWi_16	Standard	LXB4	10,00	9,3	93	11,5	6,47	0,93	0,05	14	19859	☑
230321_AKo_Calibration_16min-MWi_17	Standard	LXB4	10,00	14,7	147	12,8	6,47	0,93	0,05	13	31610	☑
230321_AKo_Calibration_16min-MWi_18	Standard	LXB4	10,00	10,5	105	6,6	6,47	0,93	0,05	16	27643	☑
230321_AKo_Calibration_16min-MWi_19	Standard	LXB4	10,00	11,6	116	17,4	6,47	0,93	0,05	13	32581	☑
230321_AKo_Calibration_16min-MWi_20	Standard	LXB4	10,00	12,1	121	16,9	6,47	0,93	0,05	15	35378	☑
230321_AKo_Calibration_16min-MWi_21	Standard	LXB4	10,00	13,5	135	7,5	6,46	0,93	0,07	15	30489	☑
230321_AKo_Calibration_16min-MWi_22	Standard	LXB4	10,00	11,1	111	19,1	6,47	0,93	0,06	13	34516	☑
230321_AKo_Calibration_16min-MWi_23	Standard	LXB4	33,33	46,0	138	28,1	6,47	0,93	0,05	19	120091	☑
230321_AKo_Calibration_16min-MWi_24	Standard	LXB4	33,33	46,4	139	16,2	6,47	0,93	0,05	18	80820	☑
230321_AKo_Calibration_16min-MWi_25	Standard	LXB4	100,00	114,9	115	26,6	6,47	0,93	0,05	21	261230	☑
230321_AKo_Calibration_16min-MWi_26	Standard	LXB4	100,00	107,8	108	36,9	6,46	0,93	0,05	20	227433	☑
230321_AKo_Calibration_16min-MWi_27	Standard	LXB4	333,33	331,6	99	33,4	6,47	0,93	0,05	21	706426	☑
230321_AKo_Calibration_16min-MWi_28	Standard	LXB4	333,33	324,8	97	30,1	6,47	0,93	0,05	23	798438	☑
230321_AKo_Calibration_16min-MWi_29	Standard	LXB4	333,33	315,9	95	25,8	6,47	0,93	0,05	23	694627	☑
230321_AKo_Calibration_16min-MWi_30	Standard	LXB4	333,33	367,1	110	21,7	6,47	0,93	0,06	20	807176	☑
230321_AKo_Calibration_16min-MWi_31	Standard	LXB4	333,33	355,6	107	42,4	6,47	0,93	0,06	22	941669	☑
230321_AKo_Calibration_16min-MWi_32	Standard	LXB4	333,33	340,5	102	32,4	6,47	0,93	0,05	21	703376	☑
230321_AKo_Calibration_16min-MWi_33	Standard	LXB4	333,33	367,8	110	31,4	6,47	0,93	0,04	21	756296	☑
230321_AKo_Calibration_16min-MWi_34	Standard	LXB4	500,00	500,0	100	27,5	6,47	0,93	0,06	21	1316614	☑
230321_AKo_Calibration_16min-MWi_35	Standard	LXB4	500,00	480,2	96	31,3	6,47	0,93	0,05	20	1021747	☑
230321_AKo_Calibration_16min-MWi_36	Standard	LXB4	500,00	520,2	104	35,9	6,46	0,93	0,06	22	1195268	☑
230321_AKo_Calibration_16min-MWi_37	Standard	LXB4	500,00	499,7	100	35,7	6,46	0,93	0,04	22	930224	☑
230321_AKo_Calibration_16min-MWi_38	Standard	LXB4	500,00	480,2	96	24,4	6,46	0,93	0,06	23	1057018	☑
230321_AKo_Calibration_16min-MWi_39	Standard	LXB4	500,00	514,9	103	27,6	6,46	0,93	0,06	22	1007394	☑
230321_AKo_Calibration_16min-MWi_40	Standard	LXB4	500,00	530,1	106	32,1	6,46	0,93	0,05	20	1065599	☑
230321_AKo_Calibration_16min-MWi_41	Standard	LXB4	1000,00	977,6	98	32,4	6,46	0,93	0,06	19	1928920	☑
230321_AKo_Calibration_16min-MWi_42	Standard	LXB4	1000,00	979,2	98	32,9	6,46	0,93	0,05	20	2144379	☑
230321_AKo_Calibration_16min-MWi_43	Standard	LXB4	2000,00	1894,4	95	35,4	6,46	0,93	0,06	23	4734778	☑
230321_AKo_Calibration_16min-MWi_44	Standard	LXB4	2000,00	1995,4	100	29,1	6,46	0,93	0,06	24	4802311	☑
230321_AKo_Calibration_16min-MWi_45	Unknown	LXB4	N/A	N/A	N/A	N/A	N/A	N/A	N/A	N/A	N/A	☑
Blank	Double Blank	LXB4	N/A	N/A	N/A	N/A	N/A	N/A	N/A	N/A	N/A	☑
Blank	Double Blank	LXB4	N/A	N/A	N/A	N/A	N/A	N/A	N/A	N/A	N/A	☑
Blank	Double Blank	LXB4	N/A	N/A	N/A	N/A	N/A	N/A	N/A	N/A	N/A	☑
Blank	Double Blank	LXB4	N/A	N/A	N/A	N/A	N/A	N/A	N/A	N/A	N/A	☑

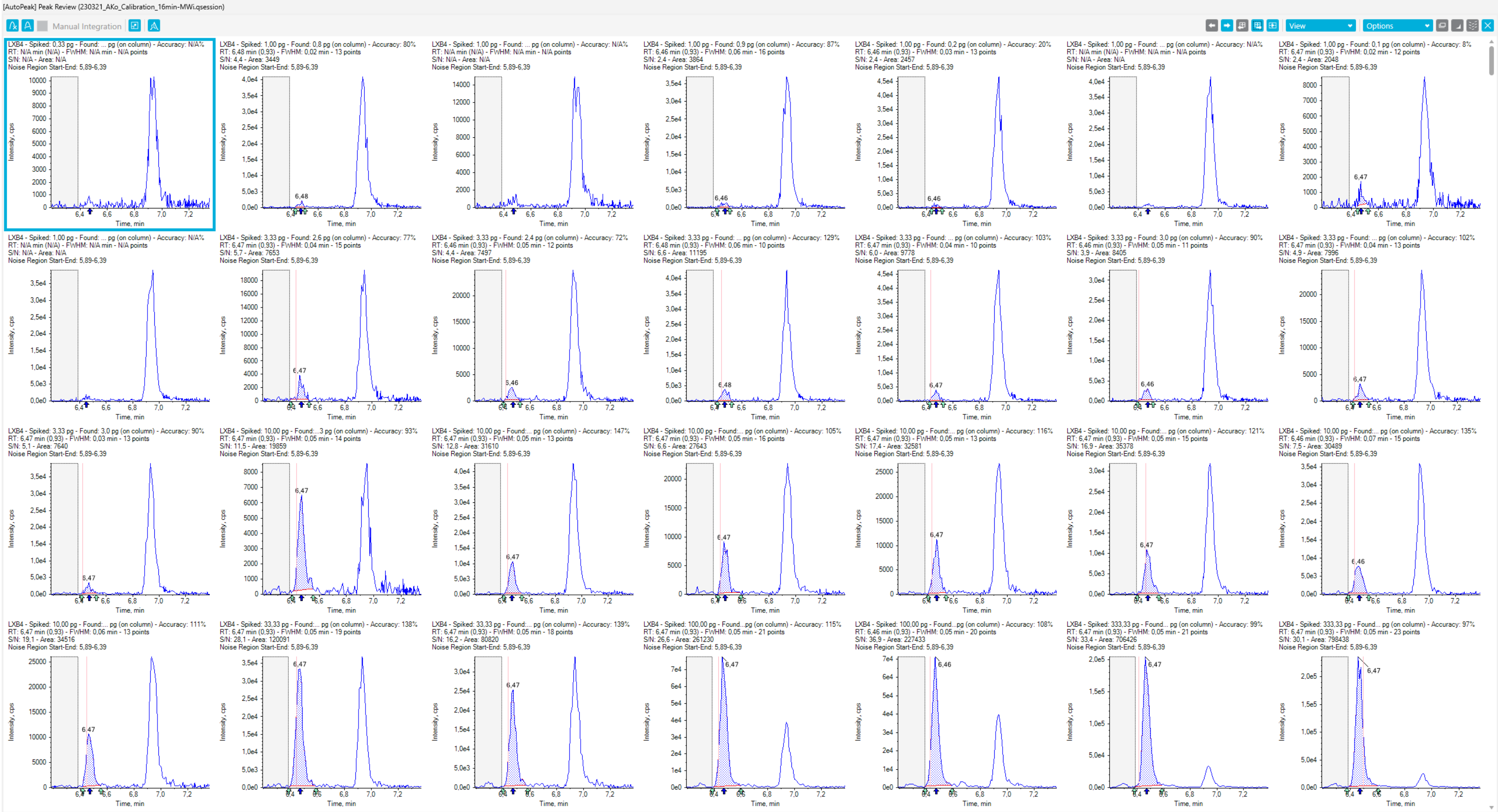
[AutoPeak] Peak Review (230321_AKo_Calibration_16min-MWi.qsession)
Manual Integration
View
Options

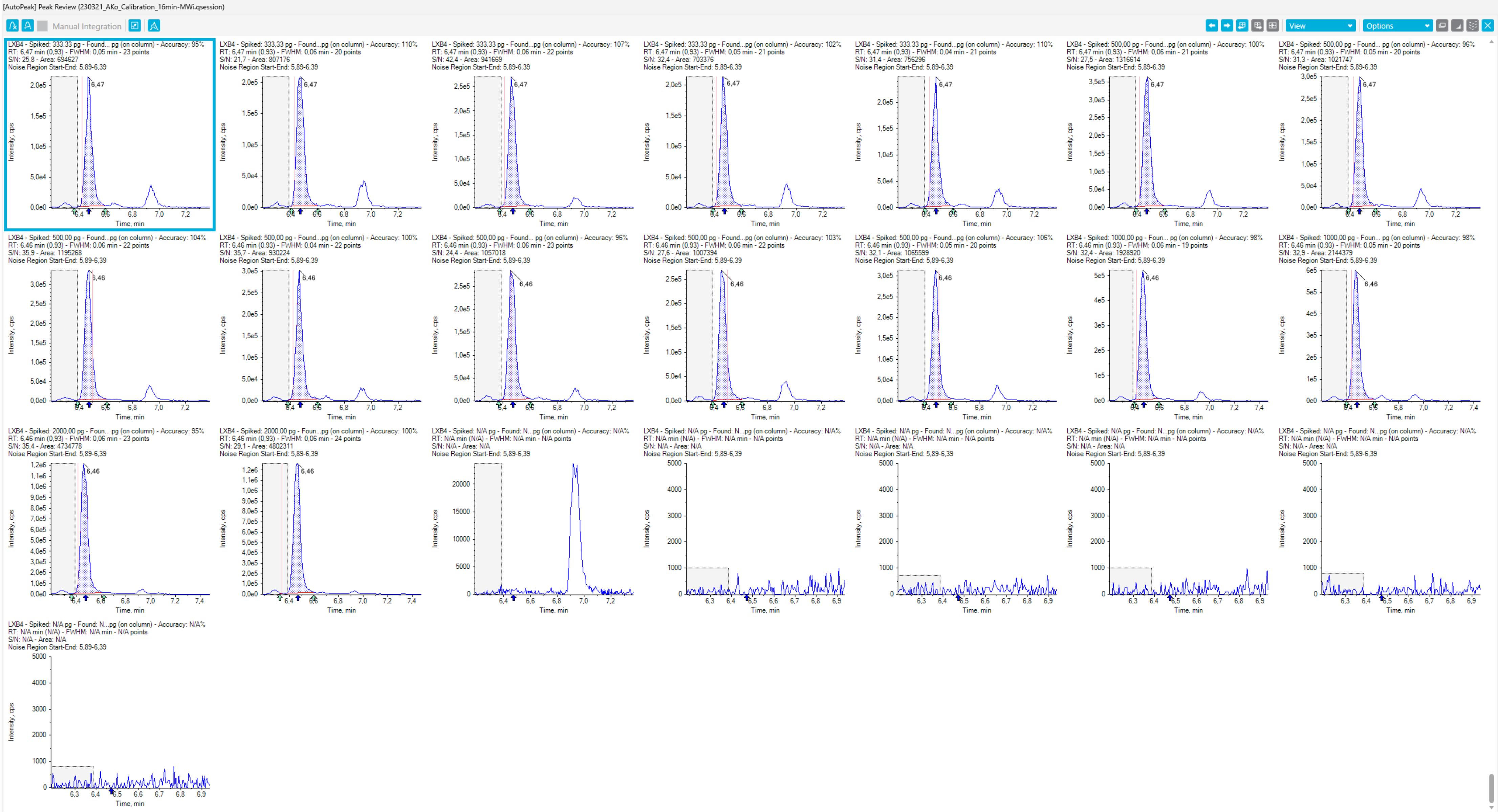
[AutoPeak] Peak Review (230321_AKo_Calibration_16min-MWi.qsession)
Manual Integration
View
Options
LXB4 - Spiked: 333,33 pg - Found... pg (on column) - Accuracy: 95%
RT: 6,47 min (0,93) - FWHM: 0,05 min - 23 points
S/N: 25,8 - Area: 694627
Noise Region Start-End: 5,89-6,39
LXB4 - Spiked: 333,33 pg - Found...pg (on column) - Accuracy: 110%
RT: 6,47 min (0,93) - FWHM: 0,06 min - 20 points
S/N: 21,7 - Area: 807176
Noise Region Start-End: 5,89-6,39
LXB4 - Spiked: 333,33 pg - Found...pg (on column) - Accuracy: 107%
RT: 6,47 min (0,93) - FWHM: 0,06 min - 22 points
S/N: 42,4 - Area: 941669
Noise Region Start-End: 5,89-6,39
LXB4 - Spiked: 333,33 pg - Found...pg (on column) - Accuracy: 102%
RT: 6,47 min (0,93) - FWHM: 0,05 min - 21 points
S/N: 32,4 - Area: 703376
Noise Region Start-End: 5,89-6,39
LXB4 - Spiked: 333,33 pg - Found...pg (on column) - Accuracy: 110%
RT: 6,47 min (0,93) - FWHM: 0,04 min - 21 points
S/N: 31,4 - Area: 756296
Noise Region Start-End: 5,89-6,39
LXB4 - Spiked: 500,00 pg - Found...pg (on column) - Accuracy: 100%
RT: 6,47 min (0,93) - FWHM: 0,06 min - 21 points
S/N: 27,5 - Area: 1316614
Noise Region Start-End: 5,89-6,39
LXB4 - Spiked: 500,00 pg - Found... pg (on column) - Accuracy: 96%
RT: 6,47 min (0,93) - FWHM: 0,05 min - 20 points
S/N: 31,3 - Area: 1021747
Noise Region Start-End: 5,89-6,39
LXB4 - Spiked: 500,00 pg - Found...pg (on column) - Accuracy: 104%
RT: 6,46 min (0,93) - FWHM: 0,06 min - 22 points
S/N: 35,9 - Area: 1195268
Noise Region Start-End: 5,89-6,39
LXB4 - Spiked: 500,00 pg - Found...pg (on column) - Accuracy: 100%
RT: 6,46 min (0,93) - FWHM: 0,04 min - 22 points
S/N: 35,7 - Area: 930224
Noise Region Start-End: 5,89-6,39
LXB4 - Spiked: 500,00 pg - Found... pg (on column) - Accuracy: 96%
RT: 6,46 min (0,93) - FWHM: 0,06 min - 23 points
S/N: 24,4 - Area: 1057018
Noise Region Start-End: 5,89-6,39
LXB4 - Spiked: 500,00 pg - Found...pg (on column) - Accuracy: 103%
RT: 6,46 min (0,93) - FWHM: 0,06 min - 22 points
S/N: 27,6 - Area: 1007394
Noise Region Start-End: 5,89-6,39
LXB4 - Spiked: 500,00 pg - Found...pg (on column) - Accuracy: 106%
RT: 6,46 min (0,93) - FWHM: 0,05 min - 20 points
S/N: 32,1 - Area: 1065599
Noise Region Start-End: 5,89-6,39
LXB4 - Spiked: 1000,00 pg - Foun... pg (on column) - Accuracy: 98%
RT: 6,46 min (0,93) - FWHM: 0,06 min - 19 points
S/N: 32,4 - Area: 1928920
Noise Region Start-End: 5,89-6,39
LXB4 - Spiked: 1000,00 pg - Foun... pg (on column) - Accuracy: 98%
RT: 6,46 min (0,93) - FWHM: 0,05 min - 20 points
S/N: 32,9 - Area: 2144379
Noise Region Start-End: 5,89-6,39
LXB4 - Spiked: 2000,00 pg - Foun... pg (on column) - Accuracy: 95%
RT: 6,46 min (0,93) - FWHM: 0,06 min - 23 points
S/N: 35,4 - Area: 4734778
Noise Region Start-End: 5,89-6,39
LXB4 - Spiked: 2000,00 pg - Foun...pg (on column) - Accuracy: 100%
RT: 6,46 min (0,93) - FWHM: 0,06 min - 24 points
S/N: 29,1 - Area: 4802311
Noise Region Start-End: 5,89-6,39
LXB4 - Spiked: N/A pg - Found: N...pg (on column) - Accuracy: N/A%
RT: N/A min (N/A) - FWHM: N/A min - N/A points
S/N: N/A - Area: N/A
Noise Region Start-End: 5,89-6,39
LXB4 - Spiked: N/A pg - Found: N...pg (on column) - Accuracy: N/A%
RT: N/A min (N/A) - FWHM: N/A min - N/A points
S/N: N/A - Area: N/A
Noise Region Start-End: 5,89-6,39
LXB4 - Spiked: N/A pg - Found: N...pg (on column) - Accuracy: N/A%
RT: N/A min (N/A) - FWHM: N/A min - N/A points
S/N: N/A - Area: N/A
Noise Region Start-End: 5,89-6,39
LXB4 - Spiked: N/A pg - Found: N...pg (on column) - Accuracy: N/A%
RT: N/A min (N/A) - FWHM: N/A min - N/A points
S/N: N/A - Area: N/A
Noise Region Start-End: 5,89-6,39
LXB4 - Spiked: N/A pg - Found: N...pg (on column) - Accuracy: N/A%
RT: N/A min (N/A) - FWHM: N/A min - N/A points
S/N: N/A - Area: N/A
Noise Region Start-End: 5,89-6,39
LXB4 - Spiked: N/A pg - Found: N...pg (on column) - Accuracy: N/A%
RT: N/A min (N/A) - FWHM: N/A min - N/A points
S/N: N/A - Area: N/A
Noise Region Start-End: 5,89-6,39
Intensity, cps
Time, min

RvD1

[AutoPeak] Results Table (230321_AKo_Calibration_16min-MWi.qsession)

50 rows Filters: 0 Qualify for Rules Filters

Sample Name	Sample T...	Component Name	Actual Concentr...	Calculated Concentrat...	Accuracy	Signal / Noise	Retent... Time	Relativ...	Width at 50%	Points Across...	Area	U...
230321_AKo_Calibration_16min-MWi_01	Standard	RvD1	0,33	N/A	N/A	N/A	N/A	N/A	N/A	N/A	N/A	☑
230321_AKo_Calibration_16min-MWi_02	Standard	RvD1	1,00	1,5	147	0,9	6,96	1,07	0,08	12	2365	☑
230321_AKo_Calibration_16min-MWi_03	Standard	RvD1	1,00	1,2	119	1,3	6,97	1,07	0,06	18	2350	☑
230321_AKo_Calibration_16min-MWi_04	Standard	RvD1	1,00	0,9	91	1,3	6,96	1,07	0,05	12	1768	☑
230321_AKo_Calibration_16min-MWi_05	Standard	RvD1	1,00	1,2	121	1,0	6,95	1,07	0,04	15	2309	☑
230321_AKo_Calibration_16min-MWi_06	Standard	RvD1	1,00	1,1	110	1,1	6,96	1,07	0,07	12	2008	☑
230321_AKo_Calibration_16min-MWi_07	Standard	RvD1	1,00	1,2	117	1,3	6,96	1,07	0,07	18	2219	☑
230321_AKo_Calibration_16min-MWi_08	Standard	RvD1	1,00	1,3	127	1,5	6,95	1,07	0,04	10	2420	☑
230321_AKo_Calibration_16min-MWi_09	Standard	RvD1	3,33	2,3	70	4,2	6,96	1,07	0,05	13	3989	☑
230321_AKo_Calibration_16min-MWi_10	Standard	RvD1	3,33	3,4	103	3,7	6,95	1,07	0,07	14	5970	☑
230321_AKo_Calibration_16min-MWi_11	Standard	RvD1	3,33	2,8	84	2,9	6,96	1,07	0,06	15	4806	☑
230321_AKo_Calibration_16min-MWi_12	Standard	RvD1	3,33	2,7	80	2,0	6,96	1,07	0,08	10	4795	☑
230321_AKo_Calibration_16min-MWi_13	Standard	RvD1	3,33	3,1	94	2,9	6,96	1,07	0,05	12	5011	☑
230321_AKo_Calibration_16min-MWi_14	Standard	RvD1	3,33	3,5	104	2,3	6,97	1,07	0,04	12	4516	☑
230321_AKo_Calibration_16min-MWi_15	Standard	RvD1	3,33	2,7	82	1,4	6,96	1,07	0,05	24	4400	☑
230321_AKo_Calibration_16min-MWi_16	Standard	RvD1	10,00	10,8	108	15,4	6,96	1,07	0,05	18	15006	☑
230321_AKo_Calibration_16min-MWi_17	Standard	RvD1	10,00	11,9	119	10,3	6,97	1,07	0,04	15	17160	☑
230321_AKo_Calibration_16min-MWi_18	Standard	RvD1	10,00	9,3	93	8,1	6,96	1,07	0,05	15	17013	☑
230321_AKo_Calibration_16min-MWi_19	Standard	RvD1	10,00	10,8	108	3,2	6,96	1,07	0,04	15	18640	☑
230321_AKo_Calibration_16min-MWi_20	Standard	RvD1	10,00	8,5	85	10,3	6,97	1,07	0,04	17	15627	☑
230321_AKo_Calibration_16min-MWi_21	Standard	RvD1	10,00	10,1	101	7,9	6,96	1,07	0,03	14	15108	☑
230321_AKo_Calibration_16min-MWi_22	Standard	RvD1	10,00	9,4	94	9,7	6,96	1,07	0,06	20	18531	☑
230321_AKo_Calibration_16min-MWi_23	Standard	RvD1	33,33	33,9	102	11,6	6,96	1,07	0,05	18	61480	☑
230321_AKo_Calibration_16min-MWi_24	Standard	RvD1	33,33	36,3	109	14,8	6,96	1,07	0,05	16	51850	☑
230321_AKo_Calibration_16min-MWi_25	Standard	RvD1	100,00	103,8	104	32,0	6,96	1,07	0,05	17	155340	☑
230321_AKo_Calibration_16min-MWi_26	Standard	RvD1	100,00	110,0	110	23,6	6,96	1,07	0,05	19	153963	☑
230321_AKo_Calibration_16min-MWi_27	Standard	RvD1	333,33	299,3	90	36,5	6,96	1,07	0,05	20	430536	☑
230321_AKo_Calibration_16min-MWi_28	Standard	RvD1	333,33	327,8	98	23,8	6,96	1,07	0,06	19	516144	☑
230321_AKo_Calibration_16min-MWi_29	Standard	RvD1	333,33	282,0	85	18,2	6,96	1,07	0,06	20	451821	☑
230321_AKo_Calibration_16min-MWi_30	Standard	RvD1	333,33	342,9	103	35,4	6,96	1,07	0,05	21	483890	☑
230321_AKo_Calibration_16min-MWi_31	Standard	RvD1	333,33	336,0	101	30,6	6,96	1,07	0,06	21	597573	☑
230321_AKo_Calibration_16min-MWi_32	Standard	RvD1	333,33	261,5	78	32,7	6,96	1,07	0,05	21	416272	☑
230321_AKo_Calibration_16min-MWi_33	Standard	RvD1	333,33	292,0	88	30,6	6,96	1,07	0,06	20	464548	☑
230321_AKo_Calibration_16min-MWi_34	Standard	RvD1	500,00	459,4	92	46,8	6,96	1,07	0,06	21	835068	☑
230321_AKo_Calibration_16min-MWi_35	Standard	RvD1	500,00	453,3	91	37,7	6,96	1,07	0,05	21	651819	☑
230321_AKo_Calibration_16min-MWi_36	Standard	RvD1	500,00	528,0	106	41,8	6,96	1,07	0,05	21	800633	☑
230321_AKo_Calibration_16min-MWi_37	Standard	RvD1	500,00	477,1	95	30,2	6,96	1,07	0,06	21	631029	☑
230321_AKo_Calibration_16min-MWi_38	Standard	RvD1	500,00	440,4	88	39,9	6,95	1,07	0,06	20	680318	☑
230321_AKo_Calibration_16min-MWi_39	Standard	RvD1	500,00	448,1	90	34,5	6,96	1,07	0,06	20	664834	☑
230321_AKo_Calibration_16min-MWi_40	Standard	RvD1	500,00	465,3	93	34,7	6,96	1,07	0,06	20	634246	☑
230321_AKo_Calibration_16min-MWi_41	Standard	RvD1	1000,00	1016,8	102	41,9	6,96	1,07	0,05	21	1389949	☑
230321_AKo_Calibration_16min-MWi_42	Standard	RvD1	1000,00	994,6	99	38,8	6,95	1,07	0,05	21	1553843	☑
230321_AKo_Calibration_16min-MWi_43	Standard	RvD1	2000,00	2067,2	103	38,6	6,95	1,07	0,05	21	3740695	☑
230321_AKo_Calibration_16min-MWi_44	Standard	RvD1	2000,00	2324,5	116	40,6	6,95	1,07	0,05	21	3795293	☑
230321_AKo_Calibration_16min-MWi_45	Unknown	RvD1	N/A	N/A	N/A	N/A	N/A	N/A	N/A	N/A	N/A	☑
Blank	Double Blank	RvD1	N/A	N/A	N/A	N/A	N/A	N/A	N/A	N/A	N/A	☑
Blank	Double Blank	RvD1	N/A	N/A	N/A	N/A	N/A	N/A	N/A	N/A	N/A	☑
Blank	Double Blank	RvD1	N/A	N/A	N/A	N/A	N/A	N/A	N/A	N/A	N/A	☑
Blank	Double Blank	RvD1	N/A	N/A	N/A	N/A	N/A	N/A	N/A	N/A	N/A	☑
[illegible]	[illegible]	[illegible]	[illegible]	[illegible]	[illegible]	[illegible]	[illegible]	[illegible]	[illegible]	[illegible]	[illegible]	[illegible]

Calibration for RvD1: y = 0,01222 x + 0,00385 (r = 0,99521, r^2 = 0,99044) (weighting: 1 / x)

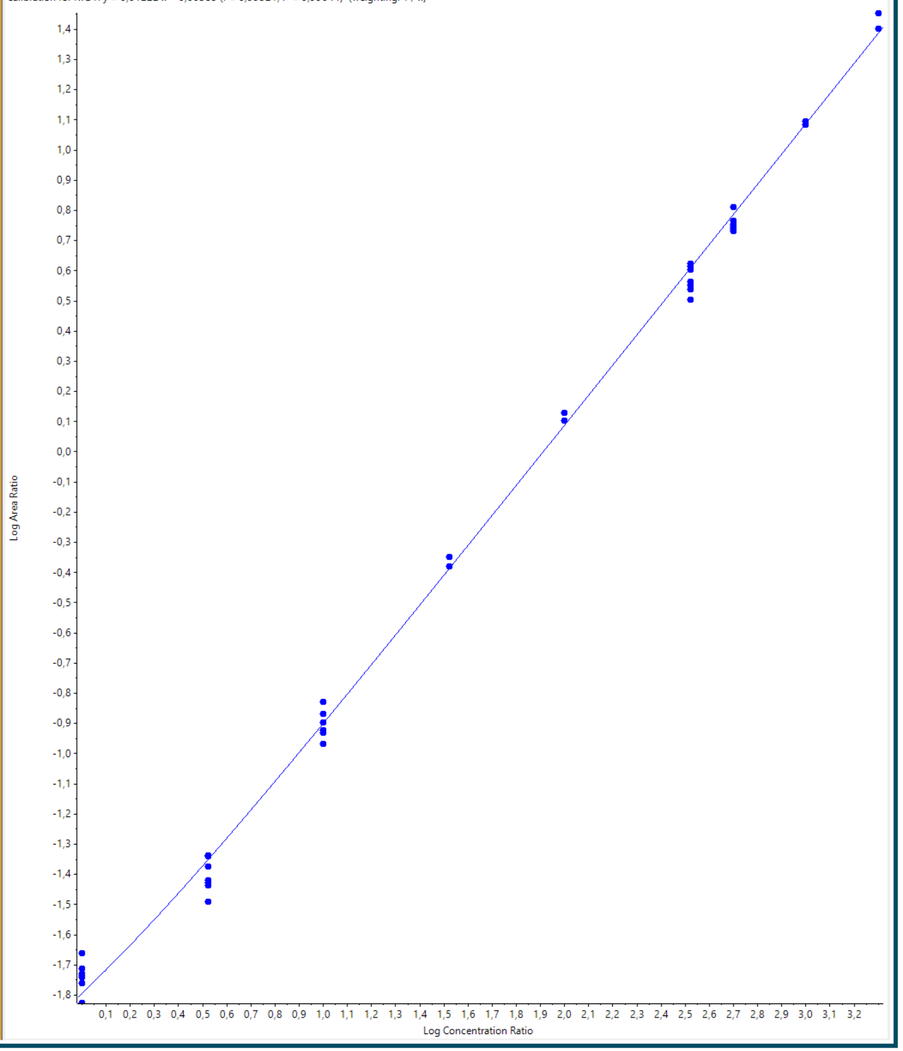

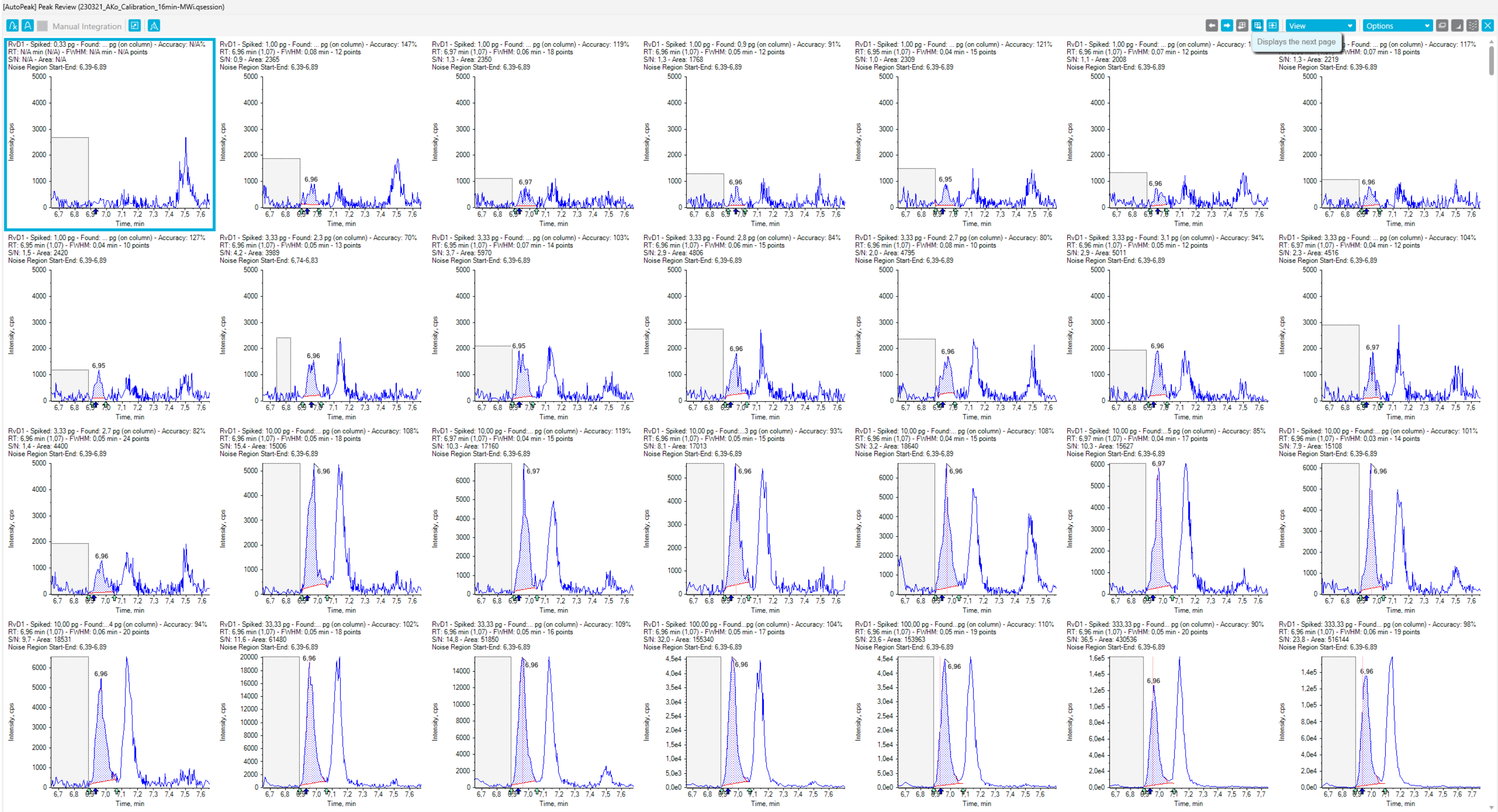
[AutoPeak] Peak Review (230321_AKo_Calibration_16min-MWi.qsession)
Manual Integration
View
Options
Displays the next page
RvD1 - Spiked: 0,33 pg - Found: ... pg (on column) - Accuracy: N/A%
RvD1 - Spiked: 1,00 pg - Found: ... pg (on column) - Accuracy: 147%
RvD1 - Spiked: 3,33 pg - Found: 2,3 pg (on column) - Accuracy: 70%
RvD1 - Spiked: 10,00 pg - Found:... pg (on column) - Accuracy: 108%
RvD1 - Spiked: 33,33 pg - Found:... pg (on column) - Accuracy: 102%
RvD1 - Spiked: 100,00 pg - Found:... pg (on column) - Accuracy: 104%
RvD1 - Spiked: 333,33 pg - Found:... pg (on column) - Accuracy: 98%
Noise Region Start-End: 6,39-6,89
Intensity, cps
Time, min

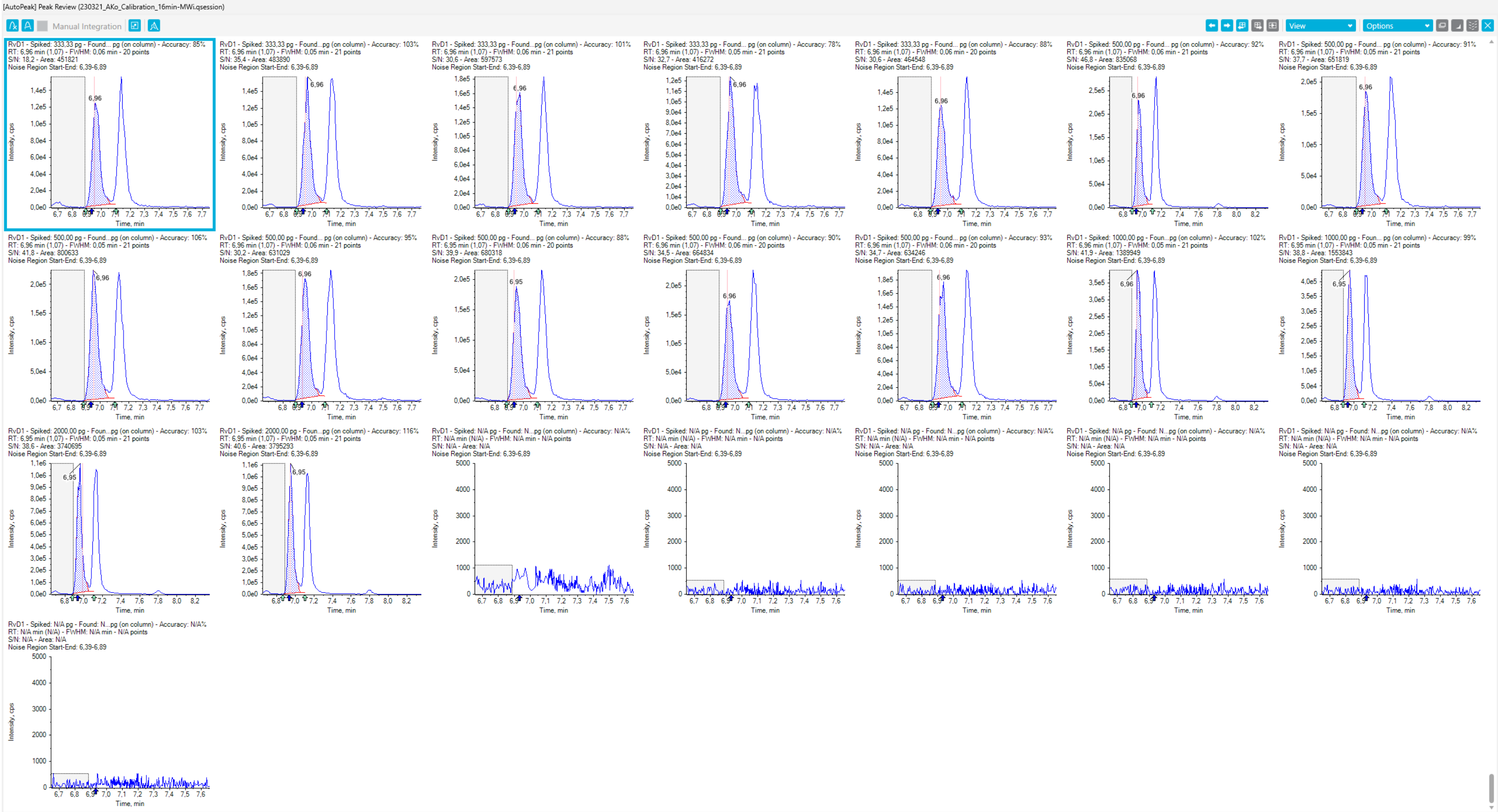
[AutoPeak] Peak Review (230321_AKo_Calibration_16min-MWi.qsession)
Manual Integration
View
Options
RvD1 - Spiked: 333,33 pg - Found... pg (on column) - Accuracy: 85%
RT: 6,96 min (1,07) - FWHM: 0,06 min - 20 points
S/N: 18,2 - Area: 451821
Noise Region Start-End: 6,39-6,89
RvD1 - Spiked: 333,33 pg - Found...pg (on column) - Accuracy: 103%
RT: 6,96 min (1,07) - FWHM: 0,06 min - 21 points
S/N: 35,4 - Area: 483890
Noise Region Start-End: 6,39-6,89
RvD1 - Spiked: 333,33 pg - Found...pg (on column) - Accuracy: 101%
RT: 6,96 min (1,07) - FWHM: 0,06 min - 21 points
S/N: 30,6 - Area: 597573
Noise Region Start-End: 6,39-6,89
RvD1 - Spiked: 333,33 pg - Found... pg (on column) - Accuracy: 78%
RT: 6,96 min (1,07) - FWHM: 0,05 min - 21 points
S/N: 32,7 - Area: 416272
Noise Region Start-End: 6,39-6,89
RvD1 - Spiked: 333,33 pg - Found... pg (on column) - Accuracy: 88%
RT: 6,96 min (1,07) - FWHM: 0,06 min - 20 points
S/N: 30,6 - Area: 464548
Noise Region Start-End: 6,39-6,89
RvD1 - Spiked: 500,00 pg - Found... pg (on column) - Accuracy: 92%
RT: 6,96 min (1,07) - FWHM: 0,06 min - 21 points
S/N: 46,8 - Area: 835068
Noise Region Start-End: 6,39-6,89
RvD1 - Spiked: 500,00 pg - Found... pg (on column) - Accuracy: 91%
RT: 6,96 min (1,07) - FWHM: 0,05 min - 21 points
S/N: 37,7 - Area: 651819
Noise Region Start-End: 6,39-6,89
RvD1 - Spiked: 500,00 pg - Found...pg (on column) - Accuracy: 106%
RT: 6,96 min (1,07) - FWHM: 0,05 min - 21 points
S/N: 41,8 - Area: 800633
Noise Region Start-End: 6,39-6,89
RvD1 - Spiked: 500,00 pg - Found...pg (on column) - Accuracy: 95%
RT: 6,96 min (1,07) - FWHM: 0,06 min - 21 points
S/N: 30,2 - Area: 631029
Noise Region Start-End: 6,39-6,89
RvD1 - Spiked: 500,00 pg - Found...pg (on column) - Accuracy: 88%
RT: 6,95 min (1,07) - FWHM: 0,06 min - 20 points
S/N: 39,9 - Area: 680318
Noise Region Start-End: 6,39-6,89
RvD1 - Spiked: 500,00 pg - Found...pg (on column) - Accuracy: 90%
RT: 6,96 min (1,07) - FWHM: 0,06 min - 20 points
S/N: 34,5 - Area: 664834
Noise Region Start-End: 6,39-6,89
RvD1 - Spiked: 500,00 pg - Found...pg (on column) - Accuracy: 93%
RT: 6,96 min (1,07) - FWHM: 0,06 min - 20 points
S/N: 34,7 - Area: 634246
Noise Region Start-End: 6,39-6,89
RvD1 - Spiked: 1000,00 pg - Foun...pg (on column) - Accuracy: 102%
RT: 6,96 min (1,07) - FWHM: 0,05 min - 21 points
S/N: 41,9 - Area: 1389949
Noise Region Start-End: 6,39-6,89
RvD1 - Spiked: 1000,00 pg - Foun... pg (on column) - Accuracy: 99%
RT: 6,95 min (1,07) - FWHM: 0,05 min - 21 points
S/N: 38,8 - Area: 1553843
Noise Region Start-End: 6,39-6,89
RvD1 - Spiked: 2000,00 pg - Foun...pg (on column) - Accuracy: 103%
RT: 6,95 min (1,07) - FWHM: 0,05 min - 21 points
S/N: 38,6 - Area: 3740695
Noise Region Start-End: 6,39-6,89
RvD1 - Spiked: 2000,00 pg - Foun...pg (on column) - Accuracy: 116%
RT: 6,95 min (1,07) - FWHM: 0,06 min - 21 points
S/N: 40,6 - Area: 3795293
Noise Region Start-End: 6,39-6,89
RvD1 - Spiked: N/A pg - Found: N...pg (on column) - Accuracy: N/A%
RT: N/A min (N/A) - FWHM: N/A min - N/A points
S/N: N/A - Area: N/A
Noise Region Start-End: 6,39-6,89
RvD1 - Spiked: N/A pg - Found: N...pg (on column) - Accuracy: N/A%
RT: N/A min (N/A) - FWHM: N/A min - N/A points
S/N: N/A - Area: N/A
Noise Region Start-End: 6,39-6,89
RvD1 - Spiked: N/A pg - Found: N...pg (on column) - Accuracy: N/A%
RT: N/A min (N/A) - FWHM: N/A min - N/A points
S/N: N/A - Area: N/A
Noise Region Start-End: 6,39-6,89
RvD1 - Spiked: N/A pg - Found: N...pg (on column) - Accuracy: N/A%
RT: N/A min (N/A) - FWHM: N/A min - N/A points
S/N: N/A - Area: N/A
Noise Region Start-End: 6,39-6,89
RvD1 - Spiked: N/A pg - Found: N...pg (on column) - Accuracy: N/A%
RT: N/A min (N/A) - FWHM: N/A min - N/A points
S/N: N/A - Area: N/A
Noise Region Start-End: 6,39-6,89
RvD1 - Spiked: N/A pg - Found: N...pg (on column) - Accuracy: N/A%
RT: N/A min (N/A) - FWHM: N/A min - N/A points
S/N: N/A - Area: N/A
Noise Region Start-End: 6,39-6,89
Intensity, cps
Time, min

AT-RvD1

[AutoPeak] Results Table (230321_AKo_Calibration_16min-MWi.qsession)

50 rows Filters: 0 Qualify for Rules Filters

Sample Name	Sample T...	Component Name	Actual Concentr...	Calculated Concentrat...	Accuracy	Signal / Noise	Retent... Time	Relativ...	Width at 50%	Points Across...	Area	U...
230321_AKo_Calibration_16min-MWi_01	Standard	AT-RvD1	0,33	N/A	N/A	N/A	N/A	N/A	N/A	N/A	N/A	✓
230321_AKo_Calibration_16min-MWi_02	Standard	AT-RvD1	1,00	1,3	129	2,1	7,14	1,09	0,04	36	1690	✓
230321_AKo_Calibration_16min-MWi_03	Standard	AT-RvD1	1,00	1,1	114	6,2	7,15	1,10	0,06	27	1742	✓
230321_AKo_Calibration_16min-MWi_04	Standard	AT-RvD1	1,00	1,1	114	3,1	7,14	1,10	0,05	33	1616	✓
230321_AKo_Calibration_16min-MWi_05	Standard	AT-RvD1	1,00	1,0	104	4,9	7,13	1,09	0,04	32	1533	✓
230321_AKo_Calibration_16min-MWi_06	Standard	AT-RvD1	1,00	1,0	104	2,4	7,13	1,09	0,03	16	1439	✓
230321_AKo_Calibration_16min-MWi_07	Standard	AT-RvD1	1,00	1,2	115	2,2	7,14	1,10	0,06	36	1694	✓
230321_AKo_Calibration_16min-MWi_08	Standard	AT-RvD1	1,00	1,3	130	2,1	7,14	1,09	0,06	34	1963	✓
230321_AKo_Calibration_16min-MWi_09	Standard	AT-RvD1	3,33	2,9	88	6,8	7,14	1,09	0,05	21	4539	✓
230321_AKo_Calibration_16min-MWi_10	Standard	AT-RvD1	3,33	3,7	110	7,2	7,13	1,09	0,05	19	6044	✓
230321_AKo_Calibration_16min-MWi_11	Standard	AT-RvD1	3,33	2,6	77	9,1	7,13	1,09	0,02	19	4055	✓
230321_AKo_Calibration_16min-MWi_12	Standard	AT-RvD1	3,33	3,4	101	6,5	7,13	1,09	0,04	25	5542	✓
230321_AKo_Calibration_16min-MWi_13	Standard	AT-RvD1	3,33	3,2	95	5,3	7,13	1,09	0,05	25	4726	✓
230321_AKo_Calibration_16min-MWi_14	Standard	AT-RvD1	3,33	3,8	113	7,2	7,14	1,09	0,02	22	4650	✓
230321_AKo_Calibration_16min-MWi_15	Standard	AT-RvD1	3,33	2,5	76	6,4	7,14	1,09	0,06	23	3740	✓
230321_AKo_Calibration_16min-MWi_16	Standard	AT-RvD1	10,00	11,2	112	22,4	7,14	1,10	0,06	28	15831	✓
230321_AKo_Calibration_16min-MWi_17	Standard	AT-RvD1	10,00	10,1	101	14,0	7,15	1,10	0,05	25	14859	✓
230321_AKo_Calibration_16min-MWi_18	Standard	AT-RvD1	10,00	9,3	93	11,8	7,14	1,10	0,05	22	17149	✓
230321_AKo_Calibration_16min-MWi_19	Standard	AT-RvD1	10,00	9,1	91	13,3	7,14	1,10	0,05	22	16032	✓
230321_AKo_Calibration_16min-MWi_20	Standard	AT-RvD1	10,00	10,6	106	26,9	7,14	1,10	0,05	25	19553	✓
230321_AKo_Calibration_16min-MWi_21	Standard	AT-RvD1	10,00	9,7	97	14,1	7,14	1,10	0,06	22	14819	✓
230321_AKo_Calibration_16min-MWi_22	Standard	AT-RvD1	10,00	10,0	100	28,4	7,14	1,09	0,05	23	19867	✓
230321_AKo_Calibration_16min-MWi_23	Standard	AT-RvD1	33,33	33,5	100	44,6	7,14	1,09	0,05	18	63341	✓
230321_AKo_Calibration_16min-MWi_24	Standard	AT-RvD1	33,33	30,8	92	39,8	7,14	1,10	0,05	20	45826	✓
230321_AKo_Calibration_16min-MWi_25	Standard	AT-RvD1	100,00	91,5	91	45,8	7,14	1,10	0,06	19	143911	✓
230321_AKo_Calibration_16min-MWi_26	Standard	AT-RvD1	100,00	99,4	99	81,6	7,13	1,09	0,05	27	146338	✓
230321_AKo_Calibration_16min-MWi_27	Standard	AT-RvD1	333,33	327,9	98	195,3	7,14	1,10	0,05	29	497152	✓
230321_AKo_Calibration_16min-MWi_28	Standard	AT-RvD1	333,33	319,4	96	284,4	7,13	1,10	0,05	21	530336	✓
230321_AKo_Calibration_16min-MWi_29	Standard	AT-RvD1	333,33	277,3	83	175,2	7,14	1,10	0,05	27	468303	✓
230321_AKo_Calibration_16min-MWi_30	Standard	AT-RvD1	333,33	374,2	112	280,4	7,14	1,10	0,05	27	556636	✓
230321_AKo_Calibration_16min-MWi_31	Standard	AT-RvD1	333,33	326,3	98	370,6	7,14	1,10	0,05	26	611799	✓
230321_AKo_Calibration_16min-MWi_32	Standard	AT-RvD1	333,33	264,2	79	132,4	7,14	1,10	0,06	27	443323	✓
230321_AKo_Calibration_16min-MWi_33	Standard	AT-RvD1	333,33	302,1	91	176,6	7,14	1,10	0,05	26	506642	✓
230321_AKo_Calibration_16min-MWi_34	Standard	AT-RvD1	500,00	503,7	101	392,3	7,14	1,10	0,05	28	965600	✓
230321_AKo_Calibration_16min-MWi_35	Standard	AT-RvD1	500,00	495,3	99	344,5	7,14	1,10	0,06	29	751034	✓
230321_AKo_Calibration_16min-MWi_36	Standard	AT-RvD1	500,00	489,2	98	265,6	7,13	1,09	0,06	27	782316	✓
230321_AKo_Calibration_16min-MWi_37	Standard	AT-RvD1	500,00	483,1	97	277,7	7,13	1,10	0,06	26	673870	✓
230321_AKo_Calibration_16min-MWi_38	Standard	AT-RvD1	500,00	451,6	90	229,8	7,13	1,10	0,05	26	735690	✓
230321_AKo_Calibration_16min-MWi_39	Standard	AT-RvD1	500,00	503,3	101	517,7	7,13	1,10	0,05	25	787491	✓
230321_AKo_Calibration_16min-MWi_40	Standard	AT-RvD1	500,00	466,9	93	293,2	7,14	1,10	0,05	25	671123	✓
230321_AKo_Calibration_16min-MWi_41	Standard	AT-RvD1	1000,00	998,0	100	639,3	7,13	1,09	0,06	24	1439310	✓
230321_AKo_Calibration_16min-MWi_42	Standard	AT-RvD1	1000,00	966,7	97	319,2	7,13	1,10	0,06	27	1593374	✓
230321_AKo_Calibration_16min-MWi_43	Standard	AT-RvD1	2000,00	2080,5	104	380,9	7,13	1,09	0,06	25	3972909	✓
230321_AKo_Calibration_16min-MWi_44	Standard	AT-RvD1	2000,00	2215,3	111	347,6	7,13	1,10	0,06	24	3817030	✓
230321_AKo_Calibration_16min-MWi_45	Unknown	AT-RvD1	N/A	N/A	N/A	N/A	N/A	N/A	N/A	N/A	N/A	✓
Blank	Double Blank	AT-RvD1	N/A	N/A	N/A	N/A	N/A	N/A	N/A	N/A	N/A	✓
Blank	Double Blank	AT-RvD1	N/A	N/A	N/A	N/A	N/A	N/A	N/A	N/A	N/A	✓
Blank	Double Blank	AT-RvD1	N/A	N/A	N/A	N/A	N/A	N/A	N/A	N/A	N/A	✓
Blank	Double Blank	AT-RvD1	N/A	N/A	N/A	N/A	N/A	N/A	N/A	N/A	N/A	✓
Blank	Double Blank	AT-RvD1	N/A	N/A	N/A	N/A	N/A	N/A	N/A	N/A	N/A	✓

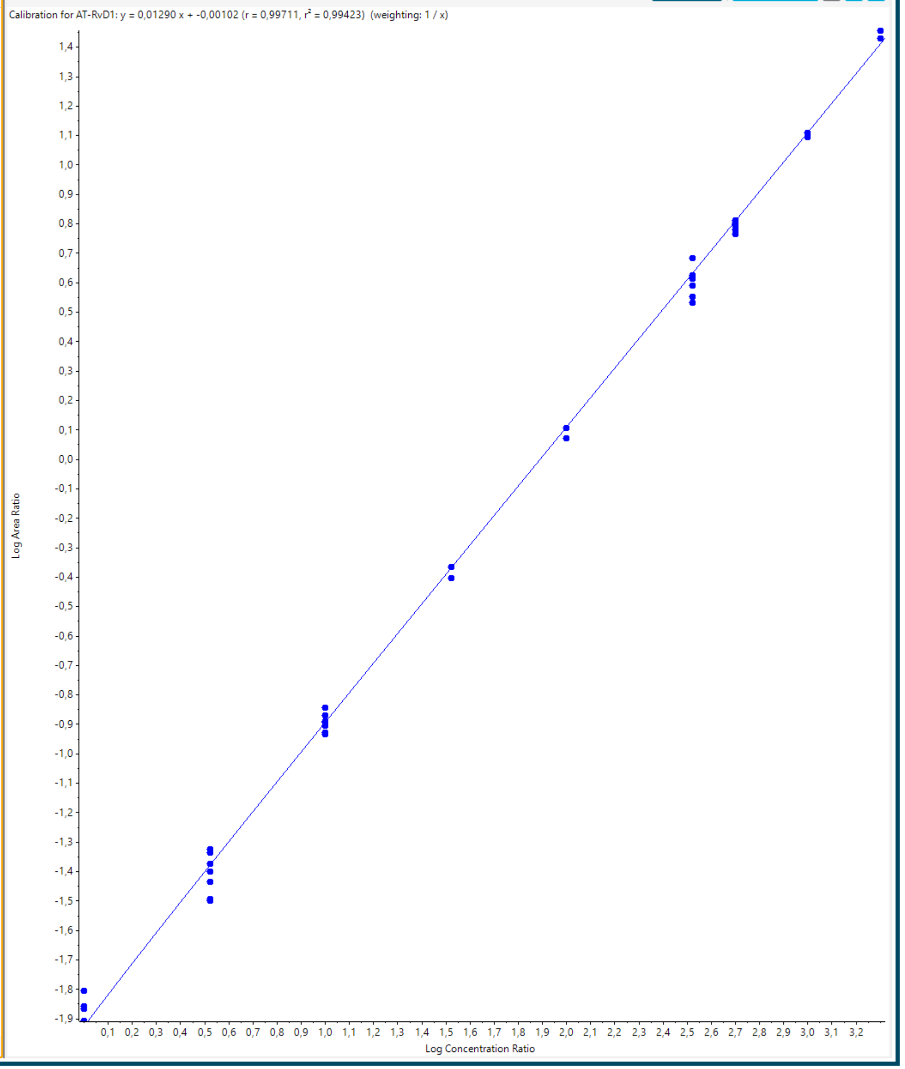

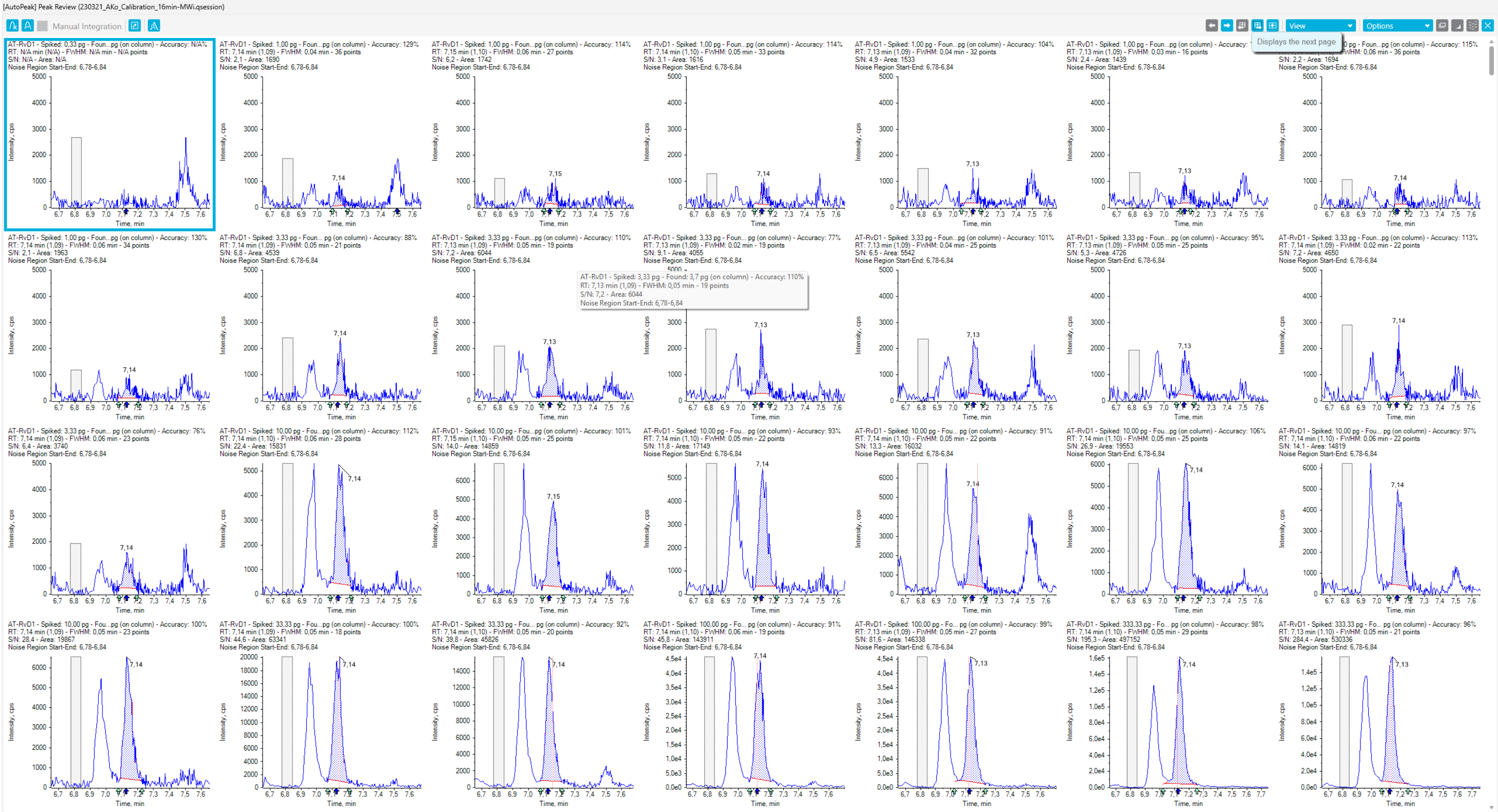
[AutoPeak] Peak Review (230321_AKo_Calibration_16min-MWi.qsession)

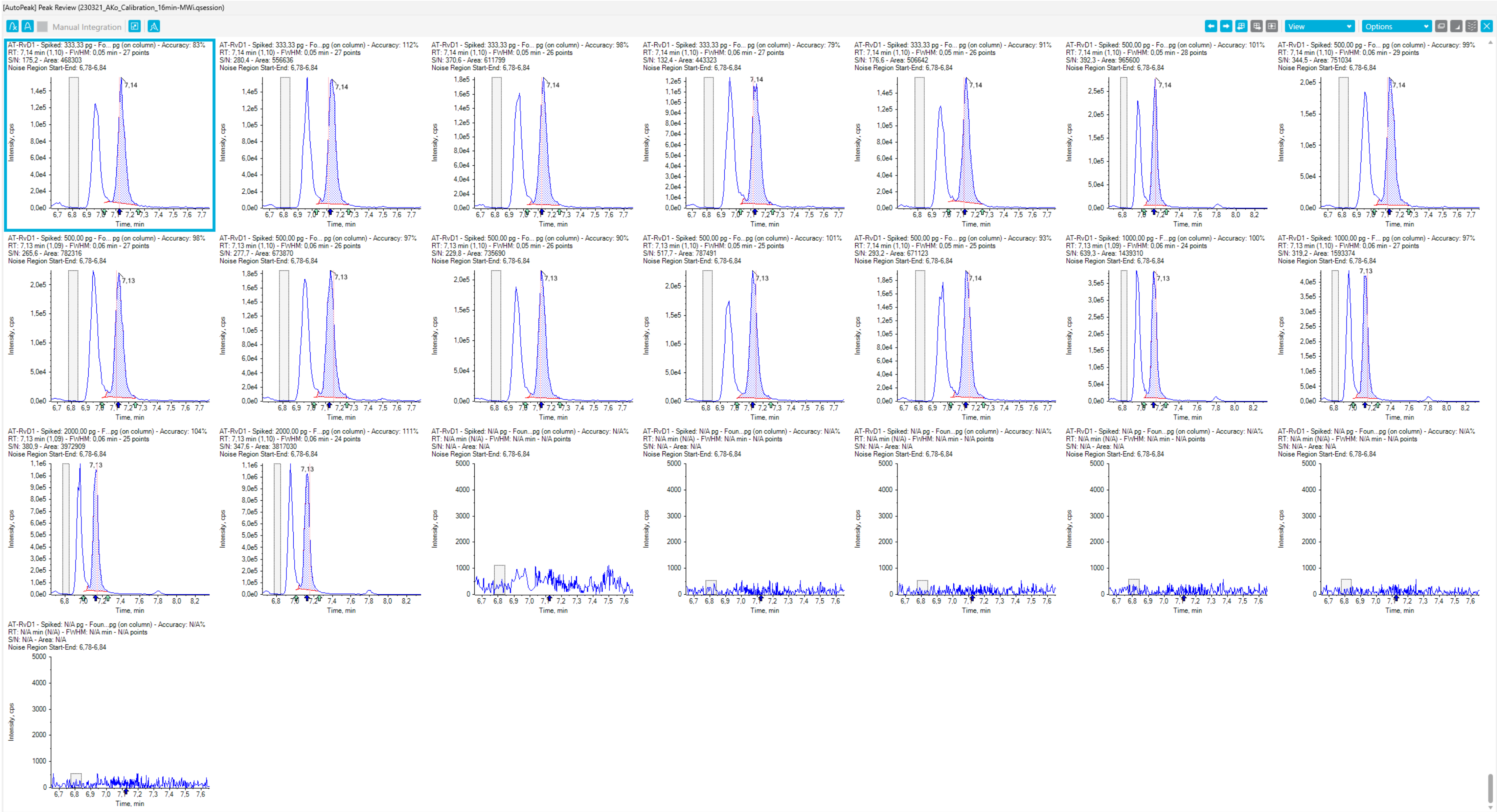
[AutoPeak] Peak Review (230321_AKo_Calibration_16min-MWi.qsession)
Manual Integration
View
Options

RvD2

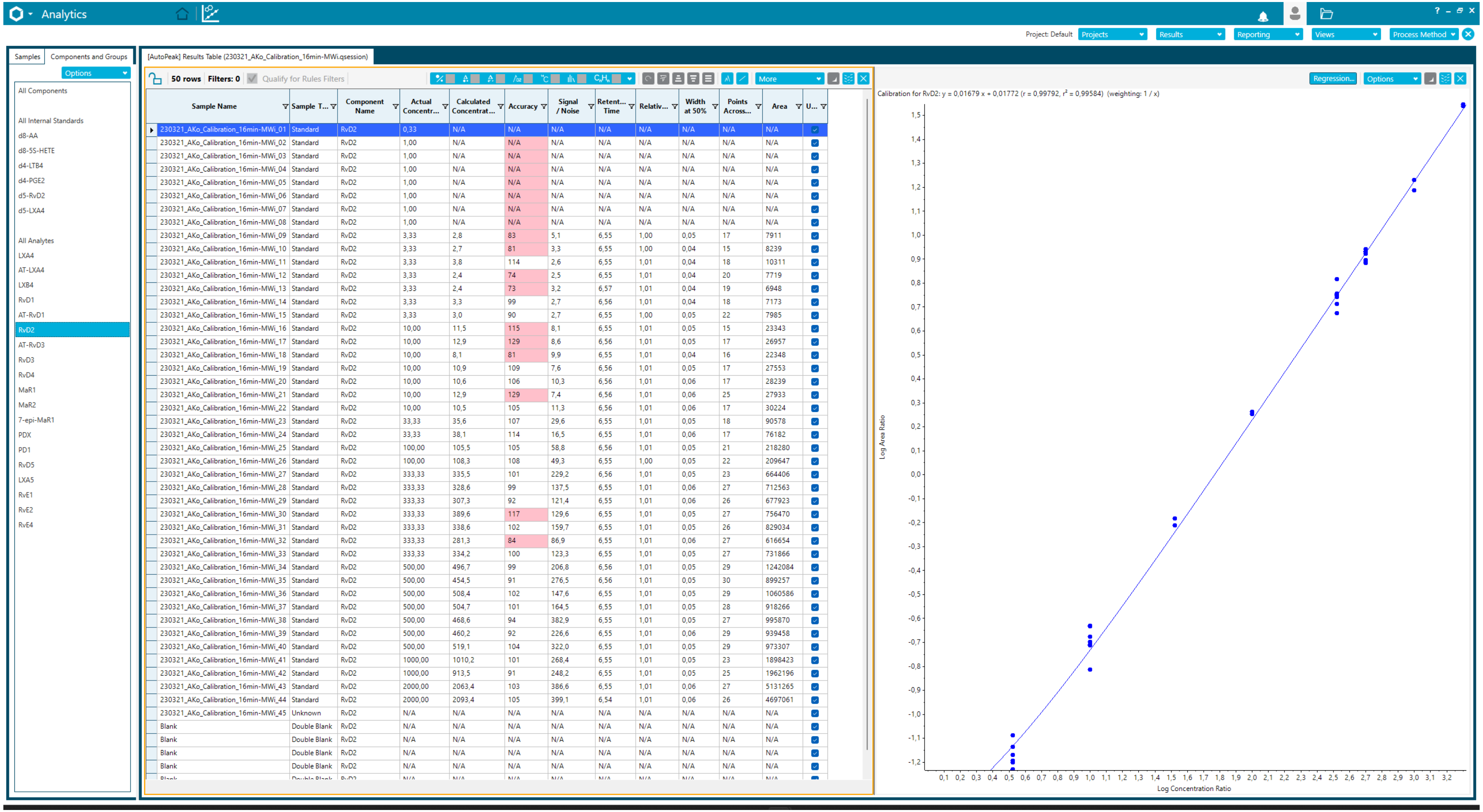

[AutoPeak] Results Table (230321_AKo_Calibration_16min-MWi.qsession)

50 rows Filters: 0 Qualify for Rules Filters

Sample Name	Sample T...	Component Name	Actual Concentr...	Calculated Concentrat...	Accuracy	Signal / Noise	Retent... Time	Relativ...	Width at 50%	Points Across...	Area	U...
230321_AKo_Calibration_16min-MWi_01	Standard	RvD2	0,33	N/A	N/A	N/A	N/A	N/A	N/A	N/A	N/A	☑
230321_AKo_Calibration_16min-MWi_02	Standard	RvD2	1,00	N/A	N/A	N/A	N/A	N/A	N/A	N/A	N/A	☑
230321_AKo_Calibration_16min-MWi_03	Standard	RvD2	1,00	N/A	N/A	N/A	N/A	N/A	N/A	N/A	N/A	☑
230321_AKo_Calibration_16min-MWi_04	Standard	RvD2	1,00	N/A	N/A	N/A	N/A	N/A	N/A	N/A	N/A	☑
230321_AKo_Calibration_16min-MWi_05	Standard	RvD2	1,00	N/A	N/A	N/A	N/A	N/A	N/A	N/A	N/A	☑
230321_AKo_Calibration_16min-MWi_06	Standard	RvD2	1,00	N/A	N/A	N/A	N/A	N/A	N/A	N/A	N/A	☑
230321_AKo_Calibration_16min-MWi_07	Standard	RvD2	1,00	N/A	N/A	N/A	N/A	N/A	N/A	N/A	N/A	☑
230321_AKo_Calibration_16min-MWi_08	Standard	RvD2	1,00	N/A	N/A	N/A	N/A	N/A	N/A	N/A	N/A	☑
230321_AKo_Calibration_16min-MWi_09	Standard	RvD2	3,33	2,8	83	5,1	6,55	1,00	0,05	17	7911	☑
230321_AKo_Calibration_16min-MWi_10	Standard	RvD2	3,33	2,7	81	3,3	6,55	1,00	0,04	15	8239	☑
230321_AKo_Calibration_16min-MWi_11	Standard	RvD2	3,33	3,8	114	2,6	6,55	1,01	0,04	18	10311	☑
230321_AKo_Calibration_16min-MWi_12	Standard	RvD2	3,33	2,4	74	2,5	6,55	1,01	0,04	20	7719	☑
230321_AKo_Calibration_16min-MWi_13	Standard	RvD2	3,33	2,4	73	3,2	6,57	1,01	0,04	19	6948	☑
230321_AKo_Calibration_16min-MWi_14	Standard	RvD2	3,33	3,3	99	2,7	6,56	1,01	0,04	18	7173	☑
230321_AKo_Calibration_16min-MWi_15	Standard	RvD2	3,33	3,0	90	2,7	6,55	1,00	0,05	22	7985	☑
230321_AKo_Calibration_16min-MWi_16	Standard	RvD2	10,00	11,5	115	8,1	6,55	1,01	0,05	15	23343	☑
230321_AKo_Calibration_16min-MWi_17	Standard	RvD2	10,00	12,9	129	8,6	6,56	1,01	0,05	17	26957	☑
230321_AKo_Calibration_16min-MWi_18	Standard	RvD2	10,00	8,1	81	9,9	6,55	1,01	0,04	16	22348	☑
230321_AKo_Calibration_16min-MWi_19	Standard	RvD2	10,00	10,9	109	7,6	6,56	1,01	0,05	17	27553	☑
230321_AKo_Calibration_16min-MWi_20	Standard	RvD2	10,00	10,6	106	10,3	6,56	1,01	0,06	17	28239	☑
230321_AKo_Calibration_16min-MWi_21	Standard	RvD2	10,00	12,9	129	7,4	6,56	1,01	0,06	25	27933	☑
230321_AKo_Calibration_16min-MWi_22	Standard	RvD2	10,00	10,5	105	11,3	6,56	1,01	0,06	17	30224	☑
230321_AKo_Calibration_16min-MWi_23	Standard	RvD2	33,33	35,6	107	29,6	6,55	1,01	0,05	18	90578	☑
230321_AKo_Calibration_16min-MWi_24	Standard	RvD2	33,33	38,1	114	16,5	6,55	1,01	0,06	17	76182	☑
230321_AKo_Calibration_16min-MWi_25	Standard	RvD2	100,00	105,5	105	58,8	6,56	1,01	0,05	21	218280	☑
230321_AKo_Calibration_16min-MWi_26	Standard	RvD2	100,00	108,3	108	49,3	6,55	1,00	0,05	22	209647	☑
230321_AKo_Calibration_16min-MWi_27	Standard	RvD2	333,33	335,5	101	229,2	6,56	1,01	0,05	23	664406	☑
230321_AKo_Calibration_16min-MWi_28	Standard	RvD2	333,33	328,6	99	137,5	6,55	1,01	0,06	27	712563	☑
230321_AKo_Calibration_16min-MWi_29	Standard	RvD2	333,33	307,3	92	121,4	6,55	1,01	0,06	26	677923	☑
230321_AKo_Calibration_16min-MWi_30	Standard	RvD2	333,33	389,6	117	129,6	6,55	1,01	0,05	27	756470	☑
230321_AKo_Calibration_16min-MWi_31	Standard	RvD2	333,33	338,6	102	159,7	6,55	1,01	0,05	26	829034	☑
230321_AKo_Calibration_16min-MWi_32	Standard	RvD2	333,33	281,3	84	86,9	6,55	1,01	0,06	27	616654	☑
230321_AKo_Calibration_16min-MWi_33	Standard	RvD2	333,33	334,2	100	123,3	6,55	1,01	0,05	27	731866	☑
230321_AKo_Calibration_16min-MWi_34	Standard	RvD2	500,00	496,7	99	206,8	6,56	1,01	0,05	29	1242084	☑
230321_AKo_Calibration_16min-MWi_35	Standard	RvD2	500,00	454,5	91	276,5	6,56	1,01	0,05	30	899257	☑
230321_AKo_Calibration_16min-MWi_36	Standard	RvD2	500,00	508,4	102	147,6	6,55	1,01	0,05	29	1060586	☑
230321_AKo_Calibration_16min-MWi_37	Standard	RvD2	500,00	504,7	101	164,5	6,55	1,01	0,05	28	918266	☑
230321_AKo_Calibration_16min-MWi_38	Standard	RvD2	500,00	468,6	94	382,9	6,55	1,01	0,05	27	995870	☑
230321_AKo_Calibration_16min-MWi_39	Standard	RvD2	500,00	460,2	92	226,6	6,55	1,01	0,06	29	939458	☑
230321_AKo_Calibration_16min-MWi_40	Standard	RvD2	500,00	519,1	104	322,0	6,55	1,01	0,05	29	973307	☑
230321_AKo_Calibration_16min-MWi_41	Standard	RvD2	1000,00	1010,2	101	268,4	6,55	1,01	0,05	23	1898423	☑
230321_AKo_Calibration_16min-MWi_42	Standard	RvD2	1000,00	913,5	91	248,2	6,55	1,01	0,05	25	1962196	☑
230321_AKo_Calibration_16min-MWi_43	Standard	RvD2	2000,00	2063,4	103	386,6	6,55	1,01	0,06	27	5131265	☑
230321_AKo_Calibration_16min-MWi_44	Standard	RvD2	2000,00	2093,4	105	399,1	6,54	1,01	0,06	26	4697061	☑
230321_AKo_Calibration_16min-MWi_45	Unknown	RvD2	N/A	N/A	N/A	N/A	N/A	N/A	N/A	N/A	N/A	☑
Blank	Double Blank	RvD2	N/A	N/A	N/A	N/A	N/A	N/A	N/A	N/A	N/A	☑
Blank	Double Blank	RvD2	N/A	N/A	N/A	N/A	N/A	N/A	N/A	N/A	N/A	☑
Blank	Double Blank	RvD2	N/A	N/A	N/A	N/A	N/A	N/A	N/A	N/A	N/A	☑
Blank	Double Blank	RvD2	N/A	N/A	N/A	N/A	N/A	N/A	N/A	N/A	N/A	☑
Blank	Double Blank	RvD2	N/A	N/A	N/A	N/A	N/A	N/A	N/A	N/A	N/A	☑

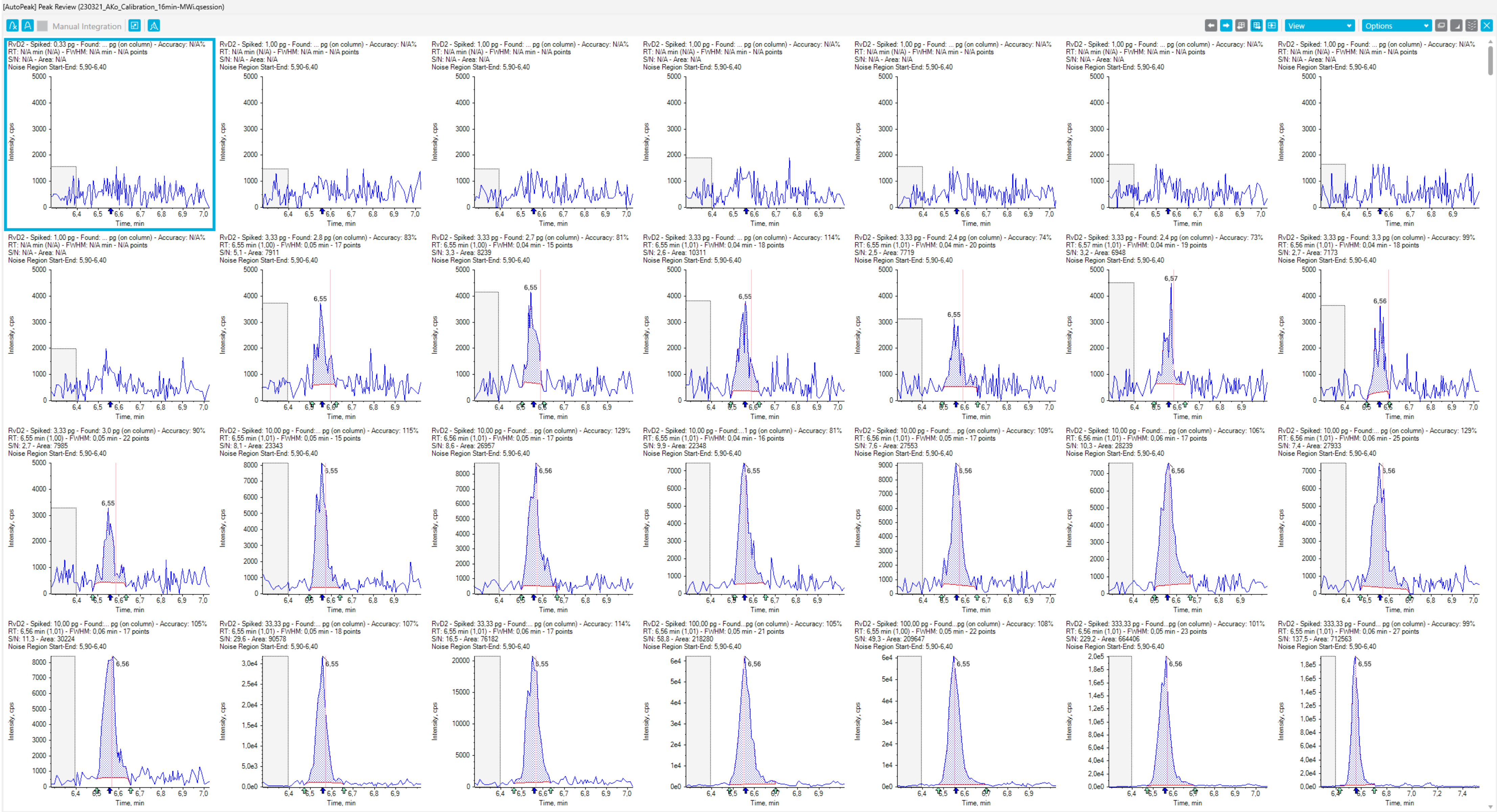
[AutoPeak] Peak Review (230321_AKo_Calibration_16min-MWi.qsession)
Manual Integration
View
Options

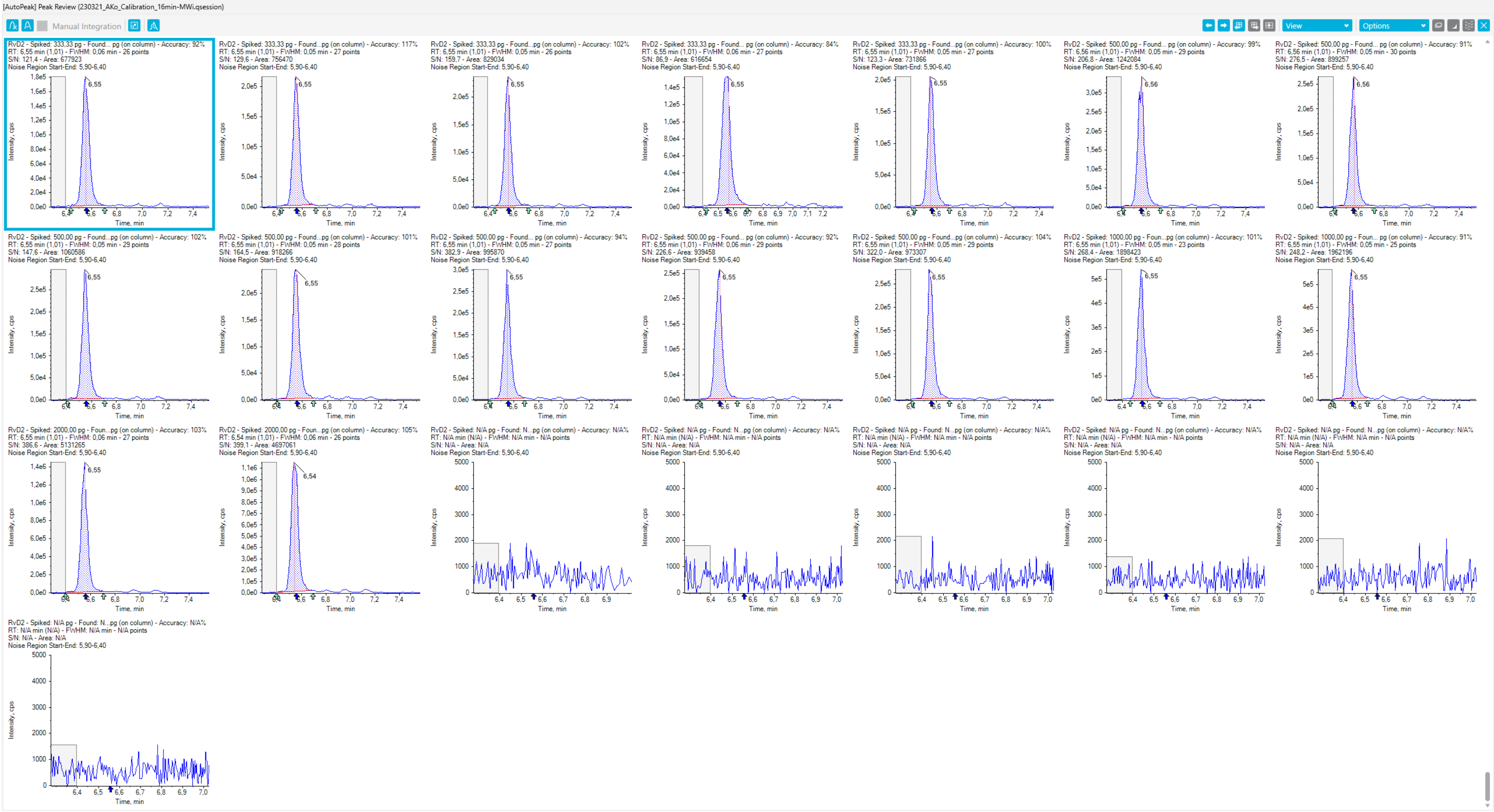

AT-RvD3

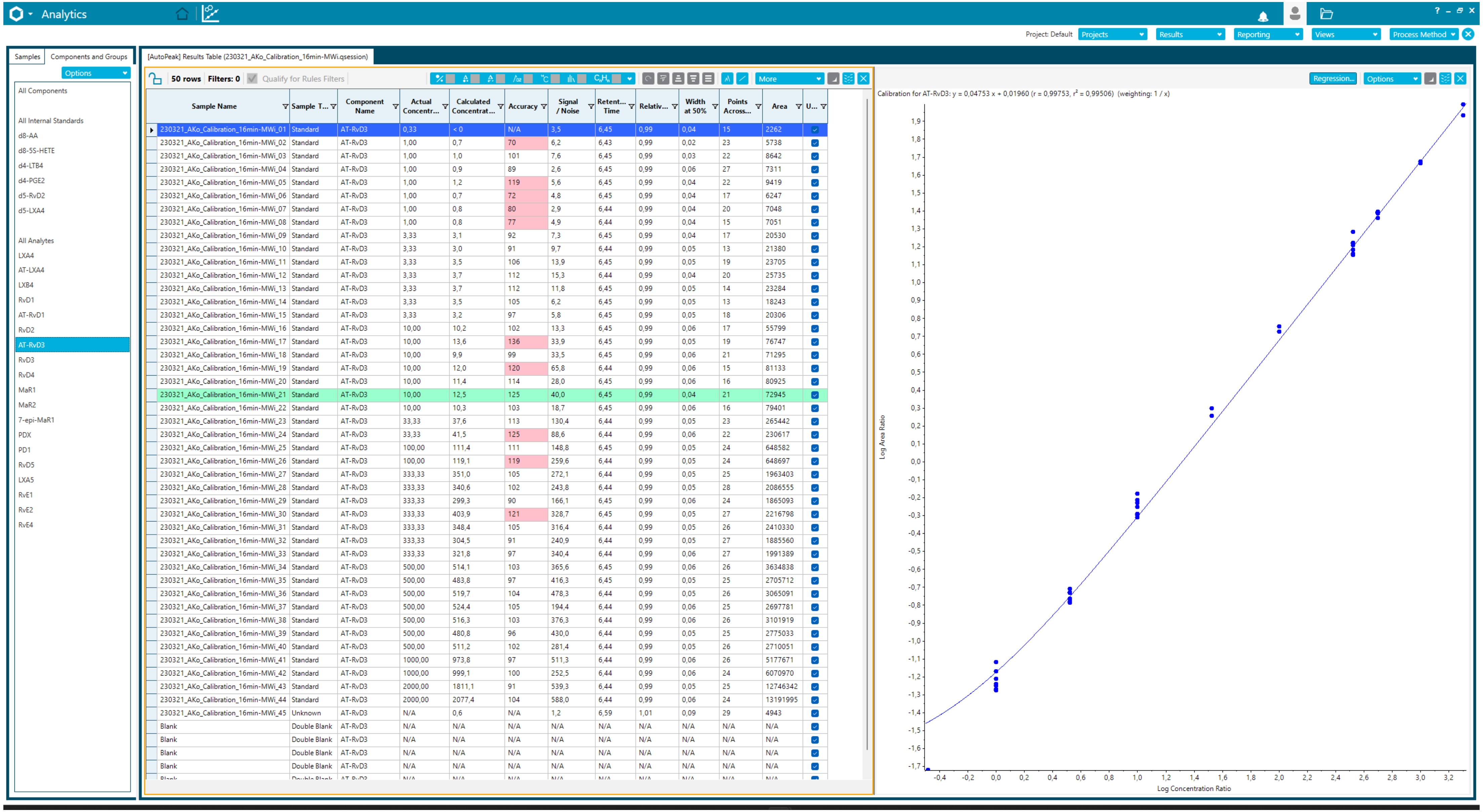

[AutoPeak] Results Table (230321_AKo_Calibration_16min-MWi.qsession)

50 rows | Filters: 0 | Qualify for Rules Filters

Sample Name	Sample T...	Component Name	Actual Concentr...	Calculated Concentrat...	Accuracy	Signal / Noise	Retent... Time	Relativ...	Width at 50%	Points Across...	Area	U...
230321_AKo_Calibration_16min-MWi_01	Standard	AT-RvD3	0,33	< 0	N/A	3,5	6,45	0,99	0,04	15	2262	☑
230321_AKo_Calibration_16min-MWi_02	Standard	AT-RvD3	1,00	0,7	70	6,2	6,43	0,99	0,02	23	5738	☑
230321_AKo_Calibration_16min-MWi_03	Standard	AT-RvD3	1,00	1,0	101	7,6	6,45	0,99	0,03	22	8642	☑
230321_AKo_Calibration_16min-MWi_04	Standard	AT-RvD3	1,00	0,9	89	2,6	6,45	0,99	0,06	27	7311	☑
230321_AKo_Calibration_16min-MWi_05	Standard	AT-RvD3	1,00	1,2	119	5,6	6,45	0,99	0,04	22	9419	☑
230321_AKo_Calibration_16min-MWi_06	Standard	AT-RvD3	1,00	0,7	72	4,8	6,45	0,99	0,04	17	6247	☑
230321_AKo_Calibration_16min-MWi_07	Standard	AT-RvD3	1,00	0,8	80	2,9	6,44	0,99	0,04	20	7048	☑
230321_AKo_Calibration_16min-MWi_08	Standard	AT-RvD3	1,00	0,8	77	4,9	6,44	0,99	0,04	15	7051	☑
230321_AKo_Calibration_16min-MWi_09	Standard	AT-RvD3	3,33	3,1	92	7,3	6,45	0,99	0,04	17	20530	☑
230321_AKo_Calibration_16min-MWi_10	Standard	AT-RvD3	3,33	3,0	91	9,7	6,44	0,99	0,05	13	21380	☑
230321_AKo_Calibration_16min-MWi_11	Standard	AT-RvD3	3,33	3,5	106	13,9	6,45	0,99	0,05	19	23705	☑
230321_AKo_Calibration_16min-MWi_12	Standard	AT-RvD3	3,33	3,7	112	15,3	6,44	0,99	0,04	20	25735	☑
230321_AKo_Calibration_16min-MWi_13	Standard	AT-RvD3	3,33	3,7	112	11,8	6,45	0,99	0,05	14	23284	☑
230321_AKo_Calibration_16min-MWi_14	Standard	AT-RvD3	3,33	3,5	105	6,2	6,45	0,99	0,05	13	18243	☑
230321_AKo_Calibration_16min-MWi_15	Standard	AT-RvD3	3,33	3,2	97	5,8	6,45	0,99	0,05	18	20306	☑
230321_AKo_Calibration_16min-MWi_16	Standard	AT-RvD3	10,00	10,2	102	13,3	6,45	0,99	0,06	17	55799	☑
230321_AKo_Calibration_16min-MWi_17	Standard	AT-RvD3	10,00	13,6	136	33,9	6,45	0,99	0,05	19	76747	☑
230321_AKo_Calibration_16min-MWi_18	Standard	AT-RvD3	10,00	9,9	99	33,5	6,45	0,99	0,06	21	71295	☑
230321_AKo_Calibration_16min-MWi_19	Standard	AT-RvD3	10,00	12,0	120	65,8	6,44	0,99	0,06	15	81133	☑
230321_AKo_Calibration_16min-MWi_20	Standard	AT-RvD3	10,00	11,4	114	28,0	6,45	0,99	0,06	16	80925	☑
230321_AKo_Calibration_16min-MWi_21	Standard	AT-RvD3	10,00	12,5	125	40,0	6,45	0,99	0,04	21	72945	☑
230321_AKo_Calibration_16min-MWi_22	Standard	AT-RvD3	10,00	10,3	103	18,7	6,45	0,99	0,06	16	79401	☑
230321_AKo_Calibration_16min-MWi_23	Standard	AT-RvD3	33,33	37,6	113	130,4	6,44	0,99	0,05	23	265442	☑
230321_AKo_Calibration_16min-MWi_24	Standard	AT-RvD3	33,33	41,5	125	88,6	6,44	0,99	0,06	22	230617	☑
230321_AKo_Calibration_16min-MWi_25	Standard	AT-RvD3	100,00	111,4	111	148,8	6,45	0,99	0,05	24	648582	☑
230321_AKo_Calibration_16min-MWi_26	Standard	AT-RvD3	100,00	119,1	119	259,6	6,44	0,99	0,05	24	648697	☑
230321_AKo_Calibration_16min-MWi_27	Standard	AT-RvD3	333,33	351,0	105	272,1	6,44	0,99	0,05	25	1963403	☑
230321_AKo_Calibration_16min-MWi_28	Standard	AT-RvD3	333,33	340,6	102	243,8	6,44	0,99	0,05	28	2086555	☑
230321_AKo_Calibration_16min-MWi_29	Standard	AT-RvD3	333,33	299,3	90	166,1	6,45	0,99	0,06	24	1865093	☑
230321_AKo_Calibration_16min-MWi_30	Standard	AT-RvD3	333,33	403,9	121	328,7	6,45	0,99	0,05	27	2216798	☑
230321_AKo_Calibration_16min-MWi_31	Standard	AT-RvD3	333,33	348,4	105	316,4	6,44	0,99	0,05	26	2410330	☑
230321_AKo_Calibration_16min-MWi_32	Standard	AT-RvD3	333,33	304,5	91	240,9	6,44	0,99	0,05	27	1885560	☑
230321_AKo_Calibration_16min-MWi_33	Standard	AT-RvD3	333,33	321,8	97	340,4	6,44	0,99	0,06	27	1991389	☑
230321_AKo_Calibration_16min-MWi_34	Standard	AT-RvD3	500,00	514,1	103	365,6	6,45	0,99	0,06	26	3634838	☑
230321_AKo_Calibration_16min-MWi_35	Standard	AT-RvD3	500,00	483,8	97	416,3	6,45	0,99	0,05	25	2705712	☑
230321_AKo_Calibration_16min-MWi_36	Standard	AT-RvD3	500,00	519,7	104	478,3	6,44	0,99	0,05	26	3065091	☑
230321_AKo_Calibration_16min-MWi_37	Standard	AT-RvD3	500,00	524,4	105	194,4	6,44	0,99	0,06	25	2697781	☑
230321_AKo_Calibration_16min-MWi_38	Standard	AT-RvD3	500,00	516,3	103	376,3	6,44	0,99	0,06	26	3101919	☑
230321_AKo_Calibration_16min-MWi_39	Standard	AT-RvD3	500,00	480,8	96	430,0	6,44	0,99	0,05	25	2775033	☑
230321_AKo_Calibration_16min-MWi_40	Standard	AT-RvD3	500,00	511,2	102	281,4	6,44	0,99	0,05	26	2710051	☑
230321_AKo_Calibration_16min-MWi_41	Standard	AT-RvD3	1000,00	973,8	97	511,3	6,44	0,99	0,06	26	5177671	☑
230321_AKo_Calibration_16min-MWi_42	Standard	AT-RvD3	1000,00	999,1	100	252,5	6,44	0,99	0,06	24	6070970	☑
230321_AKo_Calibration_16min-MWi_43	Standard	AT-RvD3	2000,00	1811,1	91	539,3	6,44	0,99	0,05	25	12746342	☑
230321_AKo_Calibration_16min-MWi_44	Standard	AT-RvD3	2000,00	2077,4	104	588,0	6,44	0,99	0,06	24	13191995	☑
230321_AKo_Calibration_16min-MWi_45	Unknown	AT-RvD3	N/A	0,6	N/A	1,2	6,59	1,01	0,09	29	4943	☑
Blank	Double Blank	AT-RvD3	N/A	N/A	N/A	N/A	N/A	N/A	N/A	N/A	N/A	☑
Blank	Double Blank	AT-RvD3	N/A	N/A	N/A	N/A	N/A	N/A	N/A	N/A	N/A	☑
Blank	Double Blank	AT-RvD3	N/A	N/A	N/A	N/A	N/A	N/A	N/A	N/A	N/A	☑
Blank	Double Blank	AT-RvD3	N/A	N/A	N/A	N/A	N/A	N/A	N/A	N/A	N/A	☑
Blank	Double Blank	AT-RvD3	N/A	N/A	N/A	N/A	N/A	N/A	N/A	N/A	N/A	☑

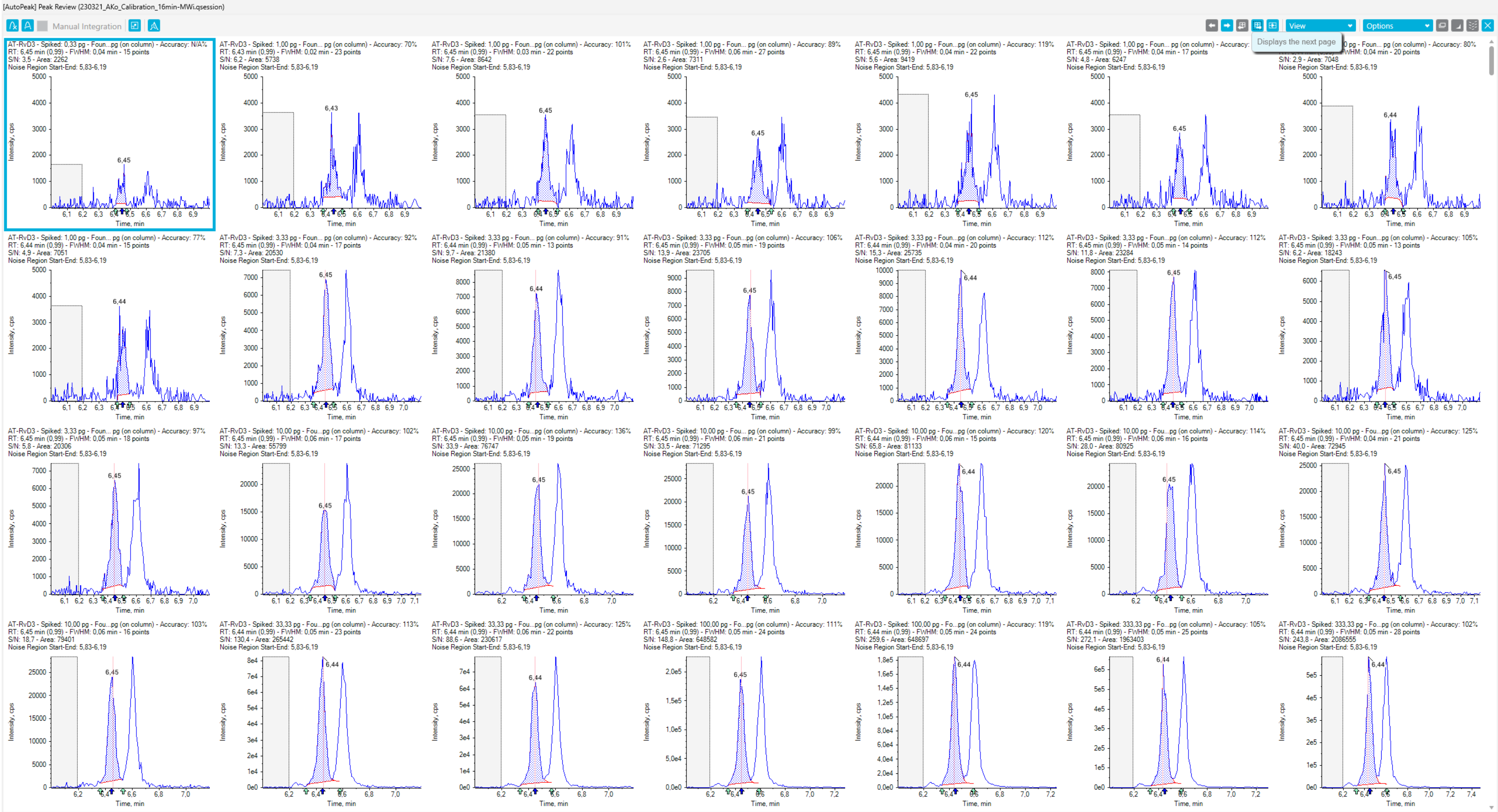

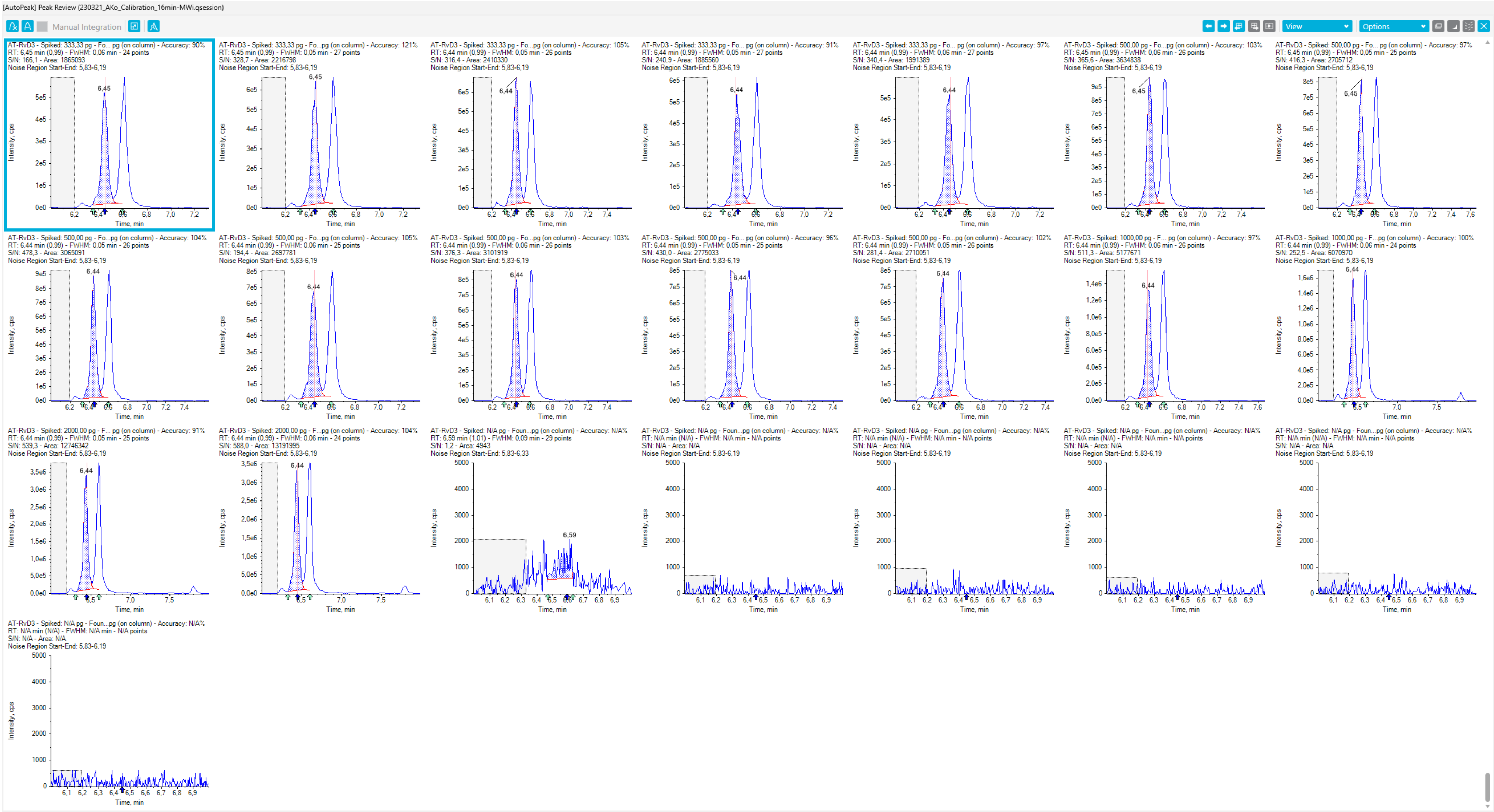

RvD3

[AutoPeak] Results Table (230321_AKo_Calibration_16min-MWi.qsession)

50 rows Filters: 0 Qualify for Rules Filters

Sample Name	Sample T...	Component Name	Actual Concentr...	Calculated Concentrat...	Accuracy	Signal / Noise	Retent... Time	Relativ...	Width at 50%	Points Across...	Area	U...
230321_AKo_Calibration_16min-MWi_01	Standard	RvD3	0,33	< 0	N/A	2,8	6,61	1,01	0,04	16	2343	☑
230321_AKo_Calibration_16min-MWi_02	Standard	RvD3	1,00	0,8	77	6,4	6,60	1,01	0,05	21	6788	☑
230321_AKo_Calibration_16min-MWi_03	Standard	RvD3	1,00	0,9	87	6,9	6,61	1,01	0,05	24	8738	☑
230321_AKo_Calibration_16min-MWi_04	Standard	RvD3	1,00	1,0	100	7,7	6,60	1,01	0,05	26	8931	☑
230321_AKo_Calibration_16min-MWi_05	Standard	RvD3	1,00	1,1	106	5,9	6,61	1,01	0,04	26	9715	☑
230321_AKo_Calibration_16min-MWi_06	Standard	RvD3	1,00	0,7	70	6,3	6,61	1,01	0,03	20	6838	☑
230321_AKo_Calibration_16min-MWi_07	Standard	RvD3	1,00	1,2	118	3,5	6,61	1,01	0,05	25	10330	☑
230321_AKo_Calibration_16min-MWi_08	Standard	RvD3	1,00	0,6	64	4,6	6,61	1,01	0,03	23	7009	☑
230321_AKo_Calibration_16min-MWi_09	Standard	RvD3	3,33	2,4	72	7,9	6,60	1,01	0,04	16	18549	☑
230321_AKo_Calibration_16min-MWi_10	Standard	RvD3	3,33	3,4	103	11,9	6,60	1,01	0,05	18	26772	☑
230321_AKo_Calibration_16min-MWi_11	Standard	RvD3	3,33	3,8	114	17,5	6,61	1,01	0,05	20	28289	☑
230321_AKo_Calibration_16min-MWi_12	Standard	RvD3	3,33	3,2	97	12,4	6,61	1,01	0,05	20	25516	☑
230321_AKo_Calibration_16min-MWi_13	Standard	RvD3	3,33	3,6	108	12,6	6,61	1,01	0,05	15	25229	☑
230321_AKo_Calibration_16min-MWi_14	Standard	RvD3	3,33	2,9	88	5,5	6,60	1,01	0,05	18	17436	☑
230321_AKo_Calibration_16min-MWi_15	Standard	RvD3	3,33	3,3	98	6,7	6,61	1,01	0,05	24	23129	☑
230321_AKo_Calibration_16min-MWi_16	Standard	RvD3	10,00	11,8	118	21,7	6,61	1,01	0,05	21	71836	☑
230321_AKo_Calibration_16min-MWi_17	Standard	RvD3	10,00	13,8	138	40,7	6,61	1,01	0,06	24	87142	☑
230321_AKo_Calibration_16min-MWi_18	Standard	RvD3	10,00	12,1	121	45,0	6,60	1,01	0,05	25	96436	☑
230321_AKo_Calibration_16min-MWi_19	Standard	RvD3	10,00	11,5	115	65,9	6,60	1,01	0,06	22	87074	☑
230321_AKo_Calibration_16min-MWi_20	Standard	RvD3	10,00	12,5	125	33,7	6,60	1,01	0,07	20	99526	☑
230321_AKo_Calibration_16min-MWi_21	Standard	RvD3	10,00	12,1	121	39,3	6,60	1,01	0,05	22	79487	☑
230321_AKo_Calibration_16min-MWi_22	Standard	RvD3	10,00	9,9	99	22,3	6,60	1,01	0,05	20	85634	☑
230321_AKo_Calibration_16min-MWi_23	Standard	RvD3	33,33	36,5	110	124,5	6,60	1,01	0,06	26	289012	☑
230321_AKo_Calibration_16min-MWi_24	Standard	RvD3	33,33	40,3	121	110,7	6,61	1,01	0,05	22	250961	☑
230321_AKo_Calibration_16min-MWi_25	Standard	RvD3	100,00	115,3	115	180,9	6,61	1,01	0,05	25	751514	☑
230321_AKo_Calibration_16min-MWi_26	Standard	RvD3	100,00	118,5	119	251,9	6,60	1,01	0,06	26	722817	☑
230321_AKo_Calibration_16min-MWi_27	Standard	RvD3	333,33	363,0	109	287,7	6,61	1,01	0,05	28	2273654	☑
230321_AKo_Calibration_16min-MWi_28	Standard	RvD3	333,33	330,7	99	243,0	6,60	1,01	0,06	29	2268309	☑
230321_AKo_Calibration_16min-MWi_29	Standard	RvD3	333,33	300,4	90	189,7	6,61	1,01	0,05	28	2096336	☑
230321_AKo_Calibration_16min-MWi_30	Standard	RvD3	333,33	394,6	118	337,6	6,61	1,01	0,06	26	2424743	☑
230321_AKo_Calibration_16min-MWi_31	Standard	RvD3	333,33	327,3	98	305,8	6,60	1,01	0,06	27	2536089	☑
230321_AKo_Calibration_16min-MWi_32	Standard	RvD3	333,33	305,9	92	277,5	6,60	1,01	0,05	27	2120652	☑
230321_AKo_Calibration_16min-MWi_33	Standard	RvD3	333,33	332,3	100	393,4	6,60	1,01	0,06	28	2302532	☑
230321_AKo_Calibration_16min-MWi_34	Standard	RvD3	500,00	497,1	99	358,2	6,61	1,01	0,06	27	3935015	☑
230321_AKo_Calibration_16min-MWi_35	Standard	RvD3	500,00	488,7	98	422,9	6,61	1,01	0,06	27	3060409	☑
230321_AKo_Calibration_16min-MWi_36	Standard	RvD3	500,00	529,5	106	499,7	6,60	1,01	0,06	29	3496908	☑
230321_AKo_Calibration_16min-MWi_37	Standard	RvD3	500,00	518,1	104	232,4	6,60	1,01	0,06	29	2984060	☑
230321_AKo_Calibration_16min-MWi_38	Standard	RvD3	500,00	511,2	102	405,3	6,60	1,01	0,06	29	3438654	☑
230321_AKo_Calibration_16min-MWi_39	Standard	RvD3	500,00	478,6	96	429,3	6,60	1,01	0,06	29	3092958	☑
230321_AKo_Calibration_16min-MWi_40	Standard	RvD3	500,00	506,9	101	298,3	6,60	1,01	0,06	27	3008817	☑
230321_AKo_Calibration_16min-MWi_41	Standard	RvD3	1000,00	1013,4	101	603,8	6,60	1,01	0,06	27	6033089	☑
230321_AKo_Calibration_16min-MWi_42	Standard	RvD3	1000,00	1004,6	100	259,6	6,60	1,01	0,06	28	6834860	☑
230321_AKo_Calibration_16min-MWi_43	Standard	RvD3	2000,00	1837,7	92	595,9	6,60	1,01	0,06	27	14482029	☑
230321_AKo_Calibration_16min-MWi_44	Standard	RvD3	2000,00	2037,6	102	630,5	6,60	1,01	0,06	27	14488389	☑
230321_AKo_Calibration_16min-MWi_45	Unknown	RvD3	N/A	0,5	N/A	2,5	6,59	1,01	0,09	22	5044	☑
Blank	Double Blank	RvD3	N/A	N/A	N/A	N/A	N/A	N/A	N/A	N/A	N/A	☑
Blank	Double Blank	RvD3	N/A	N/A	N/A	N/A	N/A	N/A	N/A	N/A	N/A	☑
Blank	Double Blank	RvD3	N/A	N/A	N/A	N/A	N/A	N/A	N/A	N/A	N/A	☑
Blank	Double Blank	RvD3	N/A	N/A	N/A	N/A	N/A	N/A	N/A	N/A	N/A	☑
Blank	Double Blank	RvD3	N/A	N/A	N/A	N/A	N/A	N/A	N/A	N/A	N/A	☑

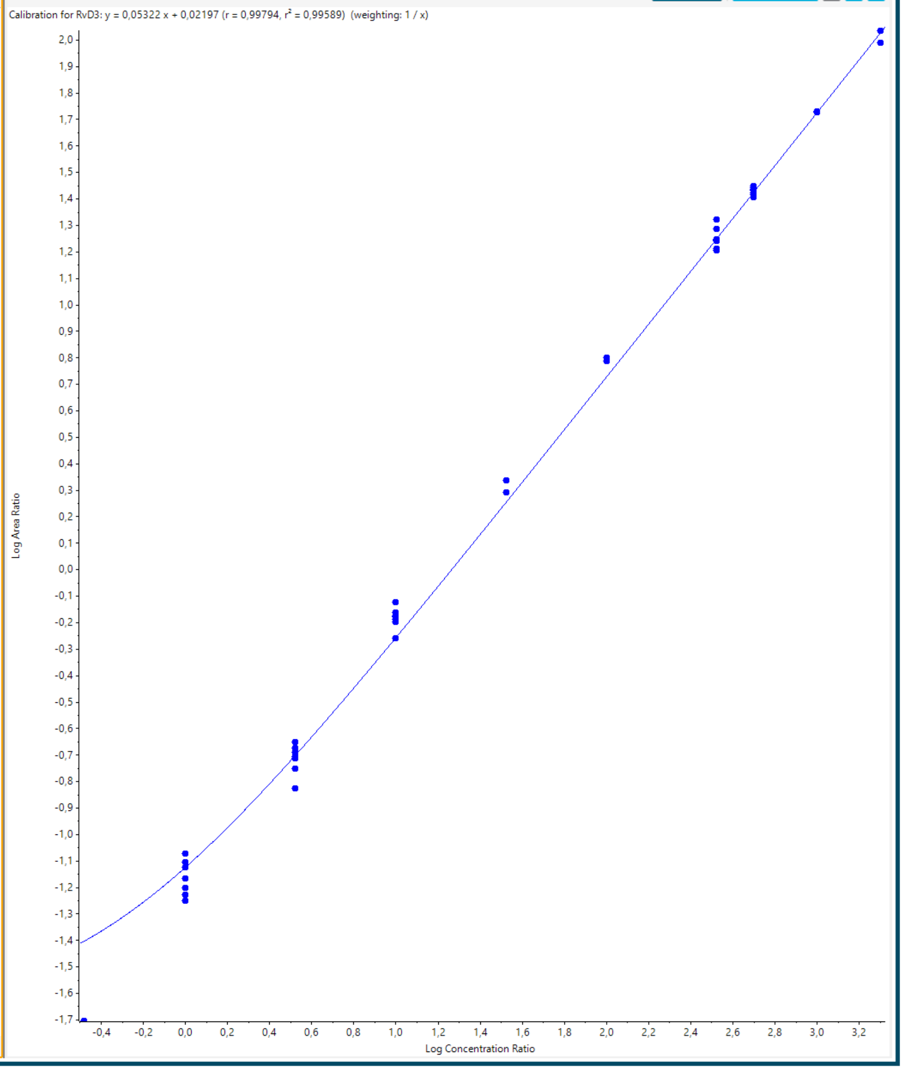

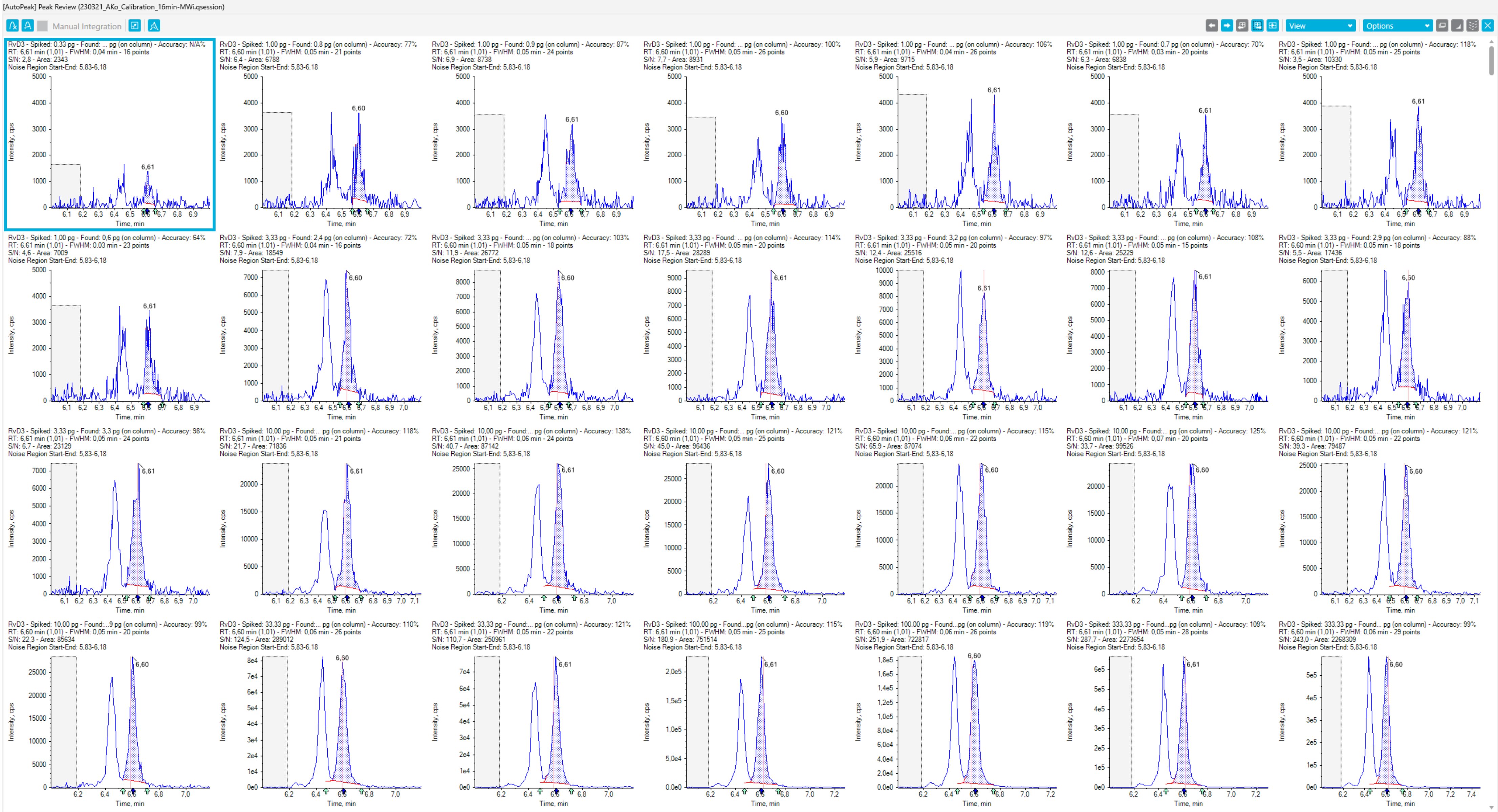
[AutoPeak] Peak Review (230321_AKo_Calibration_16min-MWi.qsession)
Manual Integration
View
Options

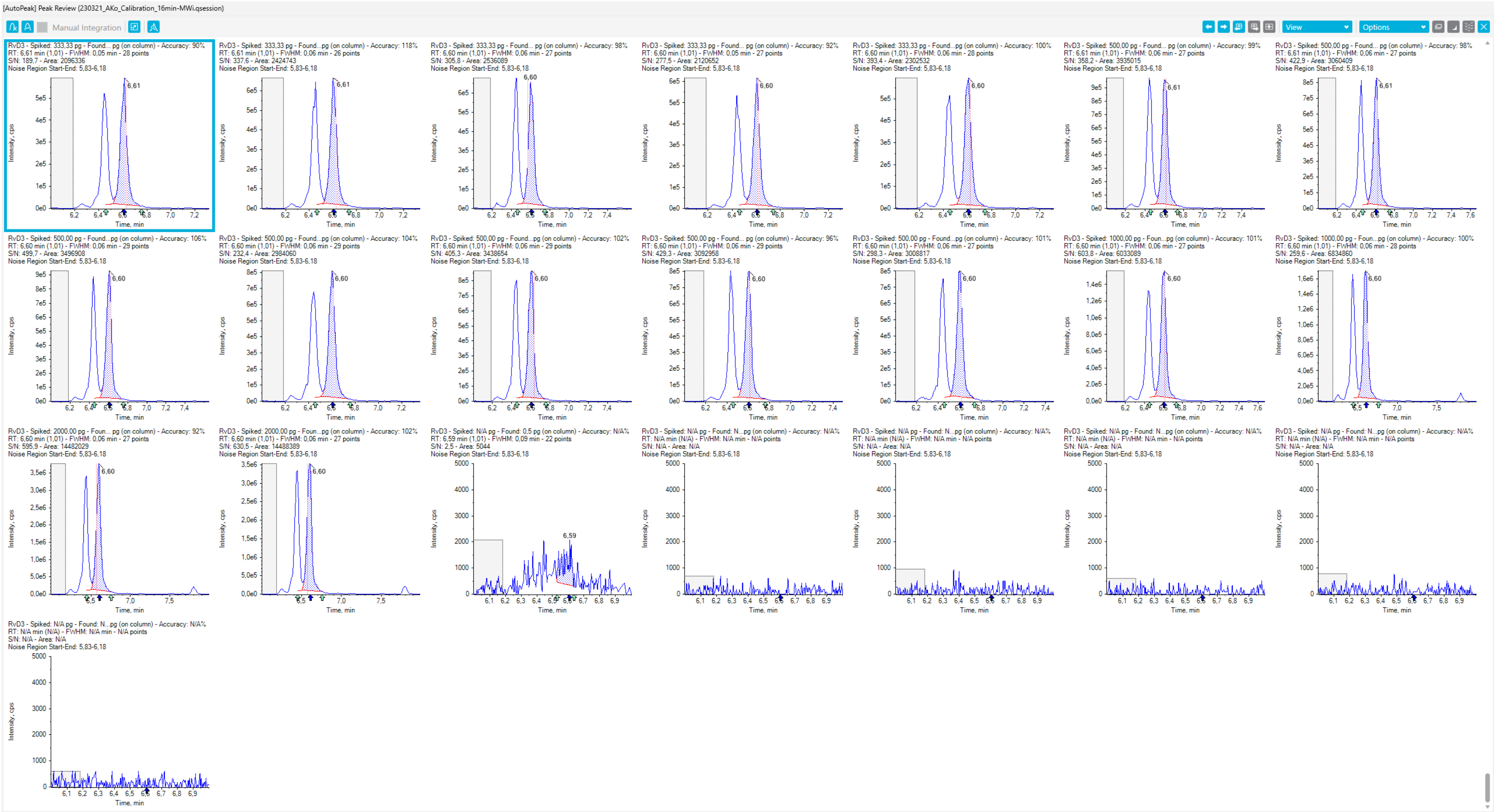
[AutoPeak] Peak Review (230321_AKo_Calibration_16min-MWi.qsession)
Manual Integration
View
Options
RvD3 - Spiked: 333,33 pg - Found... pg (on column) - Accuracy: 90%
RT: 6,61 min (1,01) - FWHM: 0,05 min - 28 points
S/N: 189,7 - Area: 2096336
Noise Region Start-End: 5,83-6,18
RvD3 - Spiked: 333,33 pg - Found...pg (on column) - Accuracy: 118%
RT: 6,61 min (1,01) - FWHM: 0,06 min - 26 points
S/N: 337,6 - Area: 2424743
Noise Region Start-End: 5,83-6,18
RvD3 - Spiked: 333,33 pg - Found... pg (on column) - Accuracy: 98%
RT: 6,60 min (1,01) - FWHM: 0,06 min - 27 points
S/N: 305,8 - Area: 2536089
Noise Region Start-End: 5,83-6,18
RvD3 - Spiked: 333,33 pg - Found... pg (on column) - Accuracy: 92%
RT: 6,60 min (1,01) - FWHM: 0,05 min - 27 points
S/N: 277,5 - Area: 2120652
Noise Region Start-End: 5,83-6,18
RvD3 - Spiked: 333,33 pg - Found...pg (on column) - Accuracy: 100%
RT: 6,60 min (1,01) - FWHM: 0,06 min - 28 points
S/N: 393,4 - Area: 2302532
Noise Region Start-End: 5,83-6,18
RvD3 - Spiked: 500,00 pg - Found... pg (on column) - Accuracy: 99%
RT: 6,61 min (1,01) - FWHM: 0,06 min - 27 points
S/N: 358,2 - Area: 3935015
Noise Region Start-End: 5,83-6,18
RvD3 - Spiked: 500,00 pg - Found... pg (on column) - Accuracy: 98%
RT: 6,61 min (1,01) - FWHM: 0,06 min - 27 points
S/N: 422,9 - Area: 3060409
Noise Region Start-End: 5,83-6,18
RvD3 - Spiked: 500,00 pg - Found...pg (on column) - Accuracy: 106%
RT: 6,60 min (1,01) - FWHM: 0,06 min - 29 points
S/N: 499,7 - Area: 3496908
Noise Region Start-End: 5,83-6,18
RvD3 - Spiked: 500,00 pg - Found...pg (on column) - Accuracy: 104%
RT: 6,60 min (1,01) - FWHM: 0,06 min - 29 points
S/N: 232,4 - Area: 2984060
Noise Region Start-End: 5,83-6,18
RvD3 - Spiked: 500,00 pg - Found...pg (on column) - Accuracy: 102%
RT: 6,60 min (1,01) - FWHM: 0,06 min - 29 points
S/N: 405,3 - Area: 3438654
Noise Region Start-End: 5,83-6,18
RvD3 - Spiked: 500,00 pg - Found... pg (on column) - Accuracy: 96%
RT: 6,60 min (1,01) - FWHM: 0,06 min - 29 points
S/N: 429,3 - Area: 3092958
Noise Region Start-End: 5,83-6,18
RvD3 - Spiked: 500,00 pg - Found...pg (on column) - Accuracy: 101%
RT: 6,60 min (1,01) - FWHM: 0,06 min - 27 points
S/N: 298,3 - Area: 3008817
Noise Region Start-End: 5,83-6,18
RvD3 - Spiked: 1000,00 pg - Foun...pg (on column) - Accuracy: 101%
RT: 6,60 min (1,01) - FWHM: 0,06 min - 27 points
S/N: 603,8 - Area: 6033089
Noise Region Start-End: 5,83-6,18
RvD3 - Spiked: 1000,00 pg - Foun...pg (on column) - Accuracy: 100%
RT: 6,60 min (1,01) - FWHM: 0,06 min - 28 points
S/N: 259,6 - Area: 6834860
Noise Region Start-End: 5,83-6,18
RvD3 - Spiked: 2000,00 pg - Foun... pg (on column) - Accuracy: 92%
RT: 6,60 min (1,01) - FWHM: 0,06 min - 27 points
S/N: 595,9 - Area: 14482029
Noise Region Start-End: 5,83-6,18
RvD3 - Spiked: 2000,00 pg - Foun...pg (on column) - Accuracy: 102%
RT: 6,60 min (1,01) - FWHM: 0,06 min - 27 points
S/N: 630,5 - Area: 14488389
Noise Region Start-End: 5,83-6,18
RvD3 - Spiked: N/A pg - Found: 0,5 pg (on column) - Accuracy: N/A%
RT: 6,59 min (1,01) - FWHM: 0,09 min - 22 points
S/N: 2,5 - Area: 5044
Noise Region Start-End: 5,83-6,18
RvD3 - Spiked: N/A pg - Found: N...pg (on column) - Accuracy: N/A%
RT: N/A min (N/A) - FWHM: N/A min - N/A points
S/N: N/A - Area: N/A
Noise Region Start-End: 5,83-6,18
RvD3 - Spiked: N/A pg - Found: N...pg (on column) - Accuracy: N/A%
RT: N/A min (N/A) - FWHM: N/A min - N/A points
S/N: N/A - Area: N/A
Noise Region Start-End: 5,83-6,18
RvD3 - Spiked: N/A pg - Found: N...pg (on column) - Accuracy: N/A%
RT: N/A min (N/A) - FWHM: N/A min - N/A points
S/N: N/A - Area: N/A
Noise Region Start-End: 5,83-6,18
RvD3 - Spiked: N/A pg - Found: N...pg (on column) - Accuracy: N/A%
RT: N/A min (N/A) - FWHM: N/A min - N/A points
S/N: N/A - Area: N/A
Noise Region Start-End: 5,83-6,18
RvD3 - Spiked: N/A pg - Found: N...pg (on column) - Accuracy: N/A%
RT: N/A min (N/A) - FWHM: N/A min - N/A points
S/N: N/A - Area: N/A
Noise Region Start-End: 5,83-6,18
Intensity, cps
Time, min

RvD4

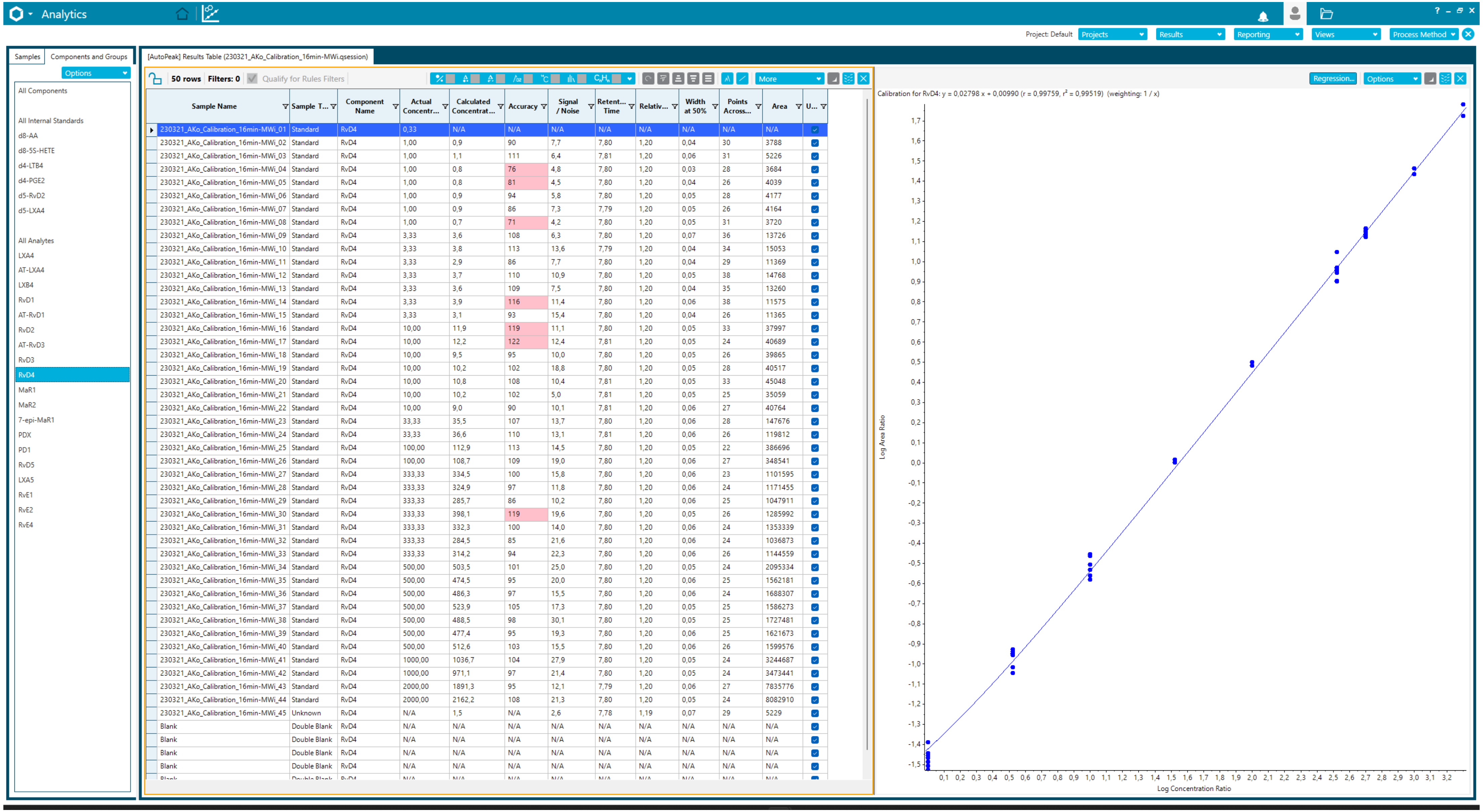

Sample Name	Sample T...	Component Name	Actual Concentr...	Calculated Concentrat...	Accuracy	Signal / Noise	Retent... Time	Relativ...	Width at 50%	Points Across...	Area	U...
230321_AKo_Calibration_16min-MWi_01	Standard	RvD4	0,33	N/A	N/A	N/A	N/A	N/A	N/A	N/A	N/A	☑
230321_AKo_Calibration_16min-MWi_02	Standard	RvD4	1,00	0,9	90	7,7	7,80	1,20	0,04	30	3788	☑
230321_AKo_Calibration_16min-MWi_03	Standard	RvD4	1,00	1,1	111	6,4	7,81	1,20	0,06	31	5226	☑
230321_AKo_Calibration_16min-MWi_04	Standard	RvD4	1,00	0,8	76	4,8	7,80	1,20	0,03	28	3684	☑
230321_AKo_Calibration_16min-MWi_05	Standard	RvD4	1,00	0,8	81	4,5	7,80	1,20	0,04	26	4039	☑
230321_AKo_Calibration_16min-MWi_06	Standard	RvD4	1,00	0,9	94	5,8	7,80	1,20	0,05	28	4177	☑
230321_AKo_Calibration_16min-MWi_07	Standard	RvD4	1,00	0,9	86	7,3	7,79	1,20	0,05	26	4164	☑
230321_AKo_Calibration_16min-MWi_08	Standard	RvD4	1,00	0,7	71	4,2	7,80	1,20	0,05	31	3720	☑
230321_AKo_Calibration_16min-MWi_09	Standard	RvD4	3,33	3,6	108	6,3	7,80	1,20	0,07	36	13726	☑
230321_AKo_Calibration_16min-MWi_10	Standard	RvD4	3,33	3,8	113	13,6	7,79	1,20	0,04	34	15053	☑
230321_AKo_Calibration_16min-MWi_11	Standard	RvD4	3,33	2,9	86	7,7	7,80	1,20	0,04	29	11369	☑
230321_AKo_Calibration_16min-MWi_12	Standard	RvD4	3,33	3,7	110	10,9	7,80	1,20	0,05	38	14768	☑
230321_AKo_Calibration_16min-MWi_13	Standard	RvD4	3,33	3,6	109	7,5	7,80	1,20	0,04	35	13260	☑
230321_AKo_Calibration_16min-MWi_14	Standard	RvD4	3,33	3,9	116	11,4	7,80	1,20	0,06	38	11575	☑
230321_AKo_Calibration_16min-MWi_15	Standard	RvD4	3,33	3,1	93	15,4	7,80	1,20	0,04	26	11365	☑
230321_AKo_Calibration_16min-MWi_16	Standard	RvD4	10,00	11,9	119	11,1	7,80	1,20	0,05	33	37997	☑
230321_AKo_Calibration_16min-MWi_17	Standard	RvD4	10,00	12,2	122	12,4	7,81	1,20	0,05	24	40689	☑
230321_AKo_Calibration_16min-MWi_18	Standard	RvD4	10,00	9,5	95	10,0	7,80	1,20	0,05	26	39865	☑
230321_AKo_Calibration_16min-MWi_19	Standard	RvD4	10,00	10,2	102	18,8	7,80	1,20	0,05	28	40517	☑
230321_AKo_Calibration_16min-MWi_20	Standard	RvD4	10,00	10,8	108	10,4	7,81	1,20	0,05	33	45048	☑
230321_AKo_Calibration_16min-MWi_21	Standard	RvD4	10,00	10,2	102	5,0	7,81	1,20	0,05	25	35059	☑
230321_AKo_Calibration_16min-MWi_22	Standard	RvD4	10,00	9,0	90	10,1	7,81	1,20	0,06	27	40764	☑
230321_AKo_Calibration_16min-MWi_23	Standard	RvD4	33,33	35,5	107	13,7	7,80	1,20	0,06	28	147676	☑
230321_AKo_Calibration_16min-MWi_24	Standard	RvD4	33,33	36,6	110	13,1	7,81	1,20	0,06	26	119812	☑
230321_AKo_Calibration_16min-MWi_25	Standard	RvD4	100,00	112,9	113	14,5	7,80	1,20	0,05	22	386696	☑
230321_AKo_Calibration_16min-MWi_26	Standard	RvD4	100,00	108,7	109	19,0	7,80	1,20	0,06	27	348541	☑
230321_AKo_Calibration_16min-MWi_27	Standard	RvD4	333,33	334,5	100	15,8	7,80	1,20	0,06	23	1101595	☑
230321_AKo_Calibration_16min-MWi_28	Standard	RvD4	333,33	324,9	97	11,8	7,80	1,20	0,06	24	1171455	☑
230321_AKo_Calibration_16min-MWi_29	Standard	RvD4	333,33	285,7	86	10,2	7,80	1,20	0,06	25	1047911	☑
230321_AKo_Calibration_16min-MWi_30	Standard	RvD4	333,33	398,1	119	19,6	7,80	1,20	0,05	26	1285992	☑
230321_AKo_Calibration_16min-MWi_31	Standard	RvD4	333,33	332,3	100	14,0	7,80	1,20	0,06	24	1353339	☑
230321_AKo_Calibration_16min-MWi_32	Standard	RvD4	333,33	284,5	85	21,6	7,80	1,20	0,06	24	1036873	☑
230321_AKo_Calibration_16min-MWi_33	Standard	RvD4	333,33	314,2	94	22,3	7,80	1,20	0,06	26	1144559	☑
230321_AKo_Calibration_16min-MWi_34	Standard	RvD4	500,00	503,5	101	25,0	7,80	1,20	0,05	24	2095334	☑
230321_AKo_Calibration_16min-MWi_35	Standard	RvD4	500,00	474,5	95	20,0	7,80	1,20	0,06	25	1562181	☑
230321_AKo_Calibration_16min-MWi_36	Standard	RvD4	500,00	486,3	97	15,5	7,80	1,20	0,06	24	1688307	☑
230321_AKo_Calibration_16min-MWi_37	Standard	RvD4	500,00	523,9	105	17,3	7,80	1,20	0,05	25	1586273	☑
230321_AKo_Calibration_16min-MWi_38	Standard	RvD4	500,00	488,5	98	30,1	7,80	1,20	0,05	25	1727481	☑
230321_AKo_Calibration_16min-MWi_39	Standard	RvD4	500,00	477,4	95	19,3	7,80	1,20	0,06	25	1621673	☑
230321_AKo_Calibration_16min-MWi_40	Standard	RvD4	500,00	512,6	103	15,5	7,80	1,20	0,06	26	1599576	☑
230321_AKo_Calibration_16min-MWi_41	Standard	RvD4	1000,00	1036,7	104	27,9	7,80	1,20	0,05	24	3244687	☑
230321_AKo_Calibration_16min-MWi_42	Standard	RvD4	1000,00	971,1	97	21,4	7,80	1,20	0,05	24	3473441	☑
230321_AKo_Calibration_16min-MWi_43	Standard	RvD4	2000,00	1891,3	95	12,1	7,79	1,20	0,06	27	7835776	☑
230321_AKo_Calibration_16min-MWi_44	Standard	RvD4	2000,00	2162,2	108	21,3	7,80	1,20	0,05	24	8082910	☑
230321_AKo_Calibration_16min-MWi_45	Unknown	RvD4	N/A	1,5	N/A	2,6	7,78	1,19	0,07	29	5229	☑
Blank	Double Blank	RvD4	N/A	N/A	N/A	N/A	N/A	N/A	N/A	N/A	N/A	☑
Blank	Double Blank	RvD4	N/A	N/A	N/A	N/A	N/A	N/A	N/A	N/A	N/A	☑
Blank	Double Blank	RvD4	N/A	N/A	N/A	N/A	N/A	N/A	N/A	N/A	N/A	☑
Blank	Double Blank	RvD4	N/A	N/A	N/A	N/A	N/A	N/A	N/A	N/A	N/A	☑
Blank	Double Blank	RvD4	N/A	N/A	N/A	N/A	N/A	N/A	N/A	N/A	N/A	☑

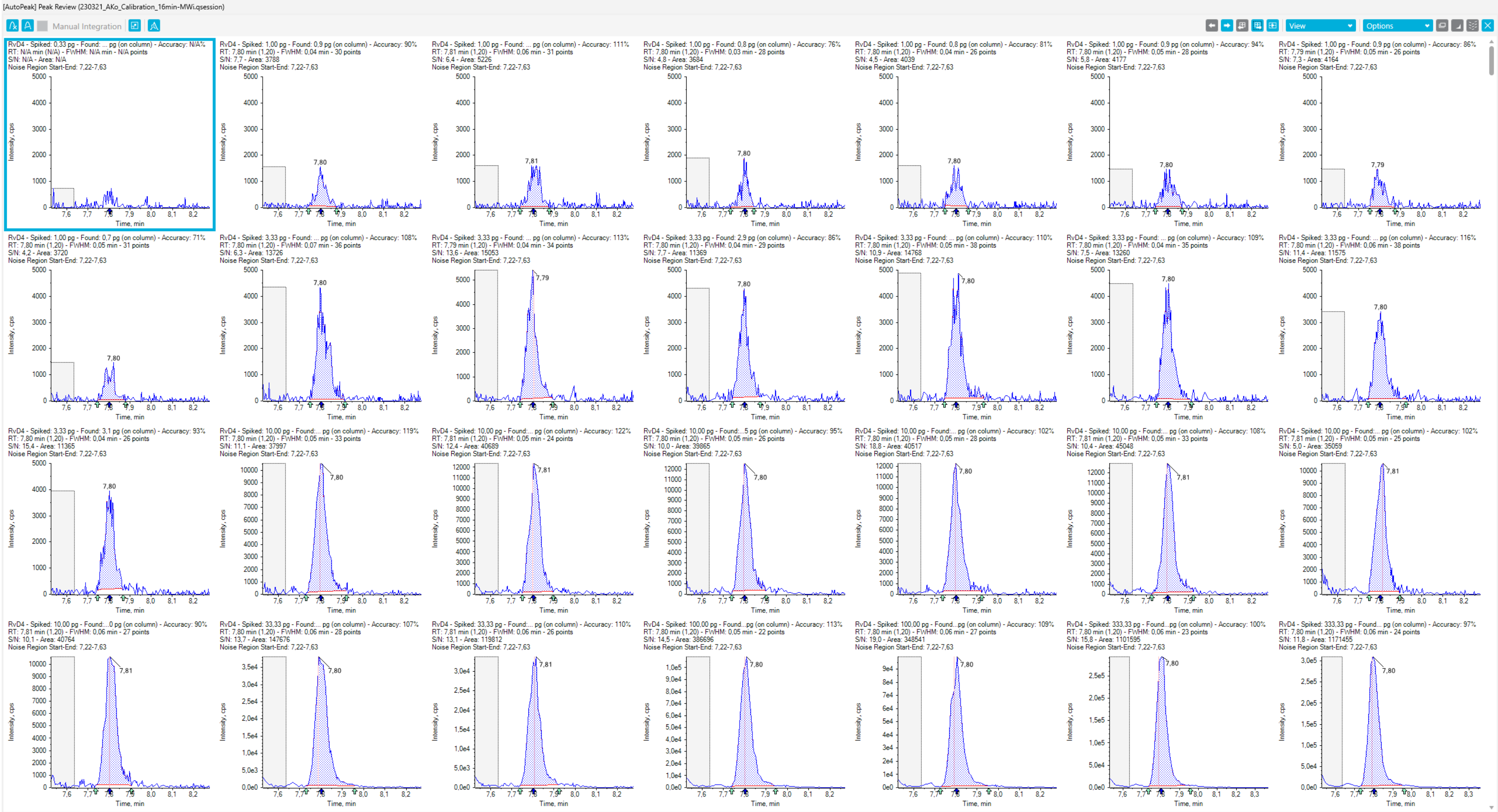
[AutoPeak] Peak Review (230321_AKo_Calibration_16min-MWi.qsession)
Manual Integration
View
Options

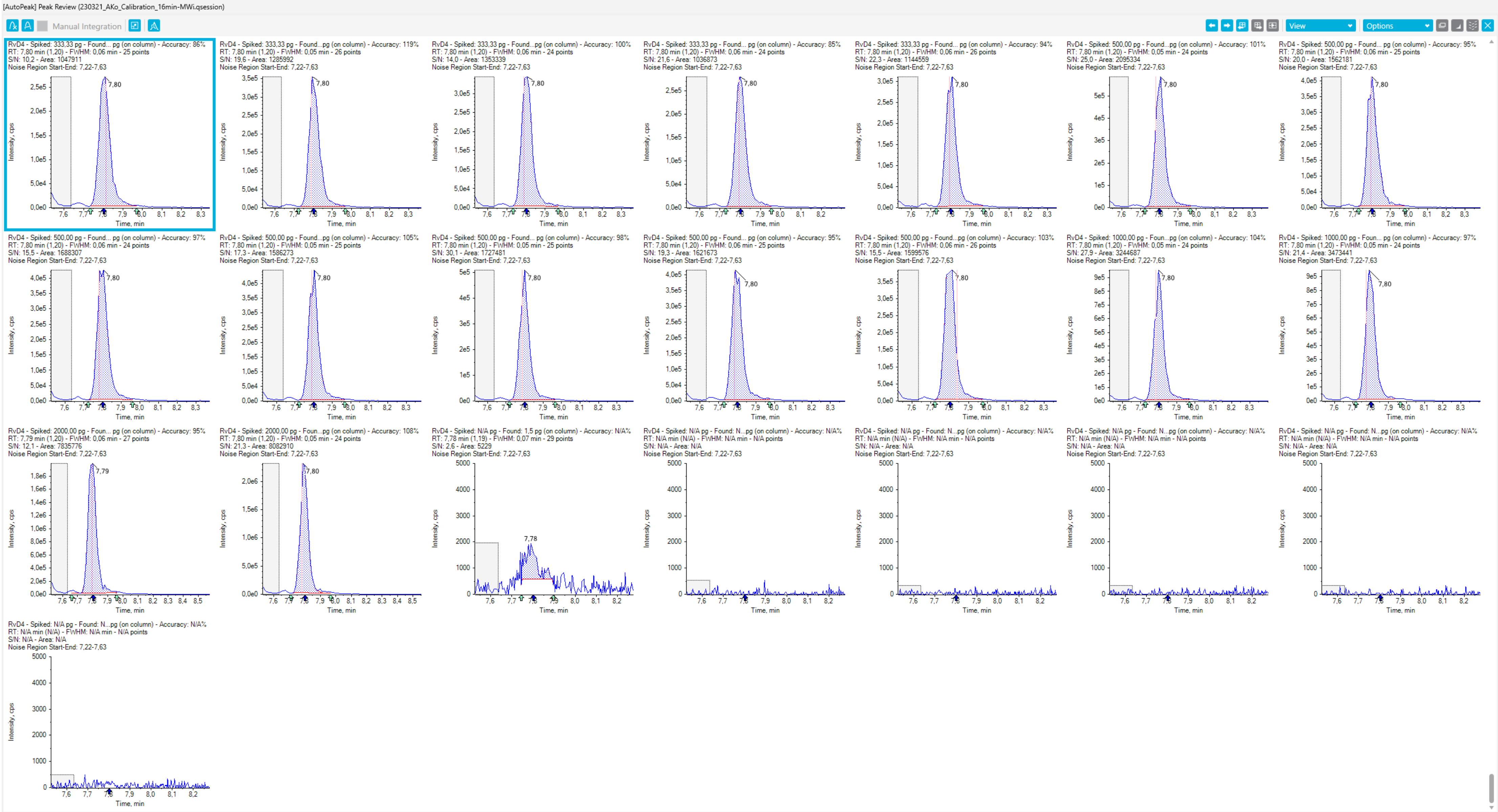
[AutoPeak] Peak Review (230321_AKo_Calibration_16min-MWi.qsession)
Manual Integration
View
Options
RvD4 - Spiked: 333,33 pg - Found... pg (on column) - Accuracy: 86%
RT: 7,80 min (1,20) - FWHM: 0,06 min - 25 points
S/N: 10,2 - Area: 1047911
Noise Region Start-End: 7,22-7,63
RvD4 - Spiked: 333,33 pg - Found...pg (on column) - Accuracy: 119%
RT: 7,80 min (1,20) - FWHM: 0,05 min - 26 points
S/N: 19,6 - Area: 1285992
Noise Region Start-End: 7,22-7,63
RvD4 - Spiked: 333,33 pg - Found...pg (on column) - Accuracy: 100%
RT: 7,80 min (1,20) - FWHM: 0,06 min - 24 points
S/N: 14,0 - Area: 1353339
Noise Region Start-End: 7,22-7,63
RvD4 - Spiked: 333,33 pg - Found... pg (on column) - Accuracy: 85%
RT: 7,80 min (1,20) - FWHM: 0,06 min - 24 points
S/N: 21,6 - Area: 1036873
Noise Region Start-End: 7,22-7,63
RvD4 - Spiked: 333,33 pg - Found... pg (on column) - Accuracy: 94%
RT: 7,80 min (1,20) - FWHM: 0,06 min - 26 points
S/N: 22,3 - Area: 1144559
Noise Region Start-End: 7,22-7,63
RvD4 - Spiked: 500,00 pg - Found...pg (on column) - Accuracy: 101%
RT: 7,80 min (1,20) - FWHM: 0,05 min - 24 points
S/N: 25,0 - Area: 2095334
Noise Region Start-End: 7,22-7,63
RvD4 - Spiked: 500,00 pg - Found... pg (on column) - Accuracy: 95%
RT: 7,80 min (1,20) - FWHM: 0,06 min - 25 points
S/N: 20,0 - Area: 1562181
Noise Region Start-End: 7,22-7,63
RvD4 - Spiked: 500,00 pg - Found... pg (on column) - Accuracy: 97%
RT: 7,80 min (1,20) - FWHM: 0,06 min - 24 points
S/N: 15,5 - Area: 1688307
Noise Region Start-End: 7,22-7,63
RvD4 - Spiked: 500,00 pg - Found...pg (on column) - Accuracy: 105%
RT: 7,80 min (1,20) - FWHM: 0,05 min - 25 points
S/N: 17,3 - Area: 1586273
Noise Region Start-End: 7,22-7,63
RvD4 - Spiked: 500,00 pg - Found... pg (on column) - Accuracy: 98%
RT: 7,80 min (1,20) - FWHM: 0,05 min - 25 points
S/N: 30,1 - Area: 1727481
Noise Region Start-End: 7,22-7,63
RvD4 - Spiked: 500,00 pg - Found... pg (on column) - Accuracy: 95%
RT: 7,80 min (1,20) - FWHM: 0,06 min - 25 points
S/N: 19,3 - Area: 1621673
Noise Region Start-End: 7,22-7,63
RvD4 - Spiked: 500,00 pg - Found...pg (on column) - Accuracy: 103%
RT: 7,80 min (1,20) - FWHM: 0,06 min - 26 points
S/N: 15,5 - Area: 1599576
Noise Region Start-End: 7,22-7,63
RvD4 - Spiked: 1000,00 pg - Foun...pg (on column) - Accuracy: 104%
RT: 7,80 min (1,20) - FWHM: 0,05 min - 24 points
S/N: 27,9 - Area: 3244687
Noise Region Start-End: 7,22-7,63
RvD4 - Spiked: 1000,00 pg - Foun... pg (on column) - Accuracy: 97%
RT: 7,80 min (1,20) - FWHM: 0,05 min - 24 points
S/N: 21,4 - Area: 3473441
Noise Region Start-End: 7,22-7,63
RvD4 - Spiked: 2000,00 pg - Foun... pg (on column) - Accuracy: 95%
RT: 7,79 min (1,20) - FWHM: 0,06 min - 27 points
S/N: 12,1 - Area: 7835776
Noise Region Start-End: 7,22-7,63
RvD4 - Spiked: 2000,00 pg - Foun... pg (on column) - Accuracy: 108%
RT: 7,80 min (1,20) - FWHM: 0,05 min - 24 points
S/N: 21,3 - Area: 8082910
Noise Region Start-End: 7,22-7,63
RvD4 - Spiked: N/A pg - Found: 1,5 pg (on column) - Accuracy: N/A%
RT: 7,78 min (1,19) - FWHM: 0,07 min - 29 points
S/N: 2,6 - Area: 5229
Noise Region Start-End: 7,22-7,63
RvD4 - Spiked: N/A pg - Found: N...pg (on column) - Accuracy: N/A%
RT: N/A min (N/A) - FWHM: N/A min - N/A points
S/N: N/A - Area: N/A
Noise Region Start-End: 7,22-7,63
Intensity, cps
Time, min

MaR1

Analytics

Project: Default | Projects | Results | Reporting | Views | Process Method

Samples | Components and Groups

Options

All Components

All Internal Standards
- d8-AA
- d8-5S-HETE
- d4-LTB4
- d4-PGE2
- d5-RvD2
- d5-LXA4

All Analytes
- LXA4
- AT-LXA4
- LXB4
- RvD1
- AT-RvD1
- RvD2
- AT-RvD3
- RvD3
- RvD4
- MaR1
- MaR2
- 7-epi-MaR1
- PDX
- PD1
- RvD5
- LXA5
- RvE1
- RvE2
- RvE4

[AutoPeak] Results Table (230321_AKo_Calibration_16min-MWi.qsession)

50 rows Filters: 0 Qualify for Rules Filters More

Sample Name	Sample T...	Component Name	Actual Concentr...	Calculated Concentrat...	Accuracy	Signal / Noise	Retent... Time	Relativ...	Width at 50%	Points Across...	Area	U...
230321_AKo_Calibration_16min-MWi_01	Standard	MaR1	0,33	0,3	84	2,3	9,21	0,98	0,04	13	390	☑
230321_AKo_Calibration_16min-MWi_02	Standard	MaR1	1,00	0,6	60	4,9	9,20	0,98	0,02	16	869	☑
230321_AKo_Calibration_16min-MWi_03	Standard	MaR1	1,00	1,1	113	8,3	9,20	0,98	0,03	25	1758	☑
230321_AKo_Calibration_16min-MWi_04	Standard	MaR1	1,00	0,8	81	4,8	9,20	0,98	0,06	21	1271	☑
230321_AKo_Calibration_16min-MWi_05	Standard	MaR1	1,00	0,8	80	11,9	9,19	0,98	0,05	19	1239	☑
230321_AKo_Calibration_16min-MWi_06	Standard	MaR1	1,00	1,0	97	5,9	9,21	0,98	0,05	23	1262	☑
230321_AKo_Calibration_16min-MWi_07	Standard	MaR1	1,00	1,2	120	22,8	9,19	0,98	0,04	23	1790	☑
230321_AKo_Calibration_16min-MWi_08	Standard	MaR1	1,00	0,9	94	7,3	9,19	0,98	0,05	18	1327	☑
230321_AKo_Calibration_16min-MWi_09	Standard	MaR1	3,33	3,1	94	13,8	9,20	0,98	0,02	34	4058	☑
230321_AKo_Calibration_16min-MWi_10	Standard	MaR1	3,33	3,4	102	13,4	9,20	0,98	0,05	25	5509	☑
230321_AKo_Calibration_16min-MWi_11	Standard	MaR1	3,33	3,3	100	12,0	9,20	0,98	0,05	29	4642	☑
230321_AKo_Calibration_16min-MWi_12	Standard	MaR1	3,33	2,9	86	4,4	9,20	0,98	0,08	32	3829	☑
230321_AKo_Calibration_16min-MWi_13	Standard	MaR1	3,33	3,7	110	8,3	9,20	0,98	0,05	44	5749	☑
230321_AKo_Calibration_16min-MWi_14	Standard	MaR1	3,33	3,3	101	8,8	9,19	0,98	0,06	23	3115	☑
230321_AKo_Calibration_16min-MWi_15	Standard	MaR1	3,33	3,2	95	18,0	9,20	0,98	0,04	29	4732	☑
230321_AKo_Calibration_16min-MWi_16	Standard	MaR1	10,00	12,0	120	44,4	9,19	0,98	0,06	49	15099	☑
230321_AKo_Calibration_16min-MWi_17	Standard	MaR1	10,00	12,7	127	25,1	9,20	0,98	0,05	44	17764	☑
230321_AKo_Calibration_16min-MWi_18	Standard	MaR1	10,00	8,8	88	24,3	9,20	0,98	0,04	30	13086	☑
230321_AKo_Calibration_16min-MWi_19	Standard	MaR1	10,00	11,1	111	39,9	9,20	0,98	0,06	45	17381	☑
230321_AKo_Calibration_16min-MWi_20	Standard	MaR1	10,00	10,5	105	41,1	9,20	0,98	0,05	40	19672	☑
230321_AKo_Calibration_16min-MWi_21	Standard	MaR1	10,00	11,8	118	36,6	9,20	0,98	0,05	44	16467	☑
230321_AKo_Calibration_16min-MWi_22	Standard	MaR1	10,00	10,7	107	29,3	9,20	0,98	0,05	42	19459	☑
230321_AKo_Calibration_16min-MWi_23	Standard	MaR1	33,33	38,4	115	111,0	9,19	0,98	0,06	41	63038	☑
230321_AKo_Calibration_16min-MWi_24	Standard	MaR1	33,33	38,5	115	56,8	9,20	0,98	0,06	42	45658	☑
230321_AKo_Calibration_16min-MWi_25	Standard	MaR1	100,00	104,9	105	120,0	9,20	0,98	0,05	38	159948	☑
230321_AKo_Calibration_16min-MWi_26	Standard	MaR1	100,00	102,4	102	181,1	9,20	0,98	0,05	37	157406	☑
230321_AKo_Calibration_16min-MWi_27	Standard	MaR1	333,33	339,6	102	315,8	9,20	0,98	0,05	36	490587	☑
230321_AKo_Calibration_16min-MWi_28	Standard	MaR1	333,33	303,9	91	124,1	9,20	0,98	0,07	32	480270	☑
230321_AKo_Calibration_16min-MWi_29	Standard	MaR1	333,33	285,3	86	203,7	9,20	0,98	0,06	36	409507	☑
230321_AKo_Calibration_16min-MWi_30	Standard	MaR1	333,33	339,3	102	164,2	9,20	0,98	0,06	31	482193	☑
230321_AKo_Calibration_16min-MWi_31	Standard	MaR1	333,33	362,5	109	367,6	9,20	0,98	0,06	34	575599	☑
230321_AKo_Calibration_16min-MWi_32	Standard	MaR1	333,33	297,9	89	416,5	9,20	0,98	0,05	35	376765	☑
230321_AKo_Calibration_16min-MWi_33	Standard	MaR1	333,33	309,8	93	214,3	9,20	0,98	0,05	30	424638	☑
230321_AKo_Calibration_16min-MWi_34	Standard	MaR1	500,00	498,5	100	205,4	9,20	0,98	0,05	31	762574	☑
230321_AKo_Calibration_16min-MWi_35	Standard	MaR1	500,00	488,5	98	227,9	9,20	0,98	0,05	32	643975	☑
230321_AKo_Calibration_16min-MWi_36	Standard	MaR1	500,00	483,8	97	317,0	9,19	0,98	0,06	31	700199	☑
230321_AKo_Calibration_16min-MWi_37	Standard	MaR1	500,00	527,1	105	249,3	9,19	0,98	0,05	31	676419	☑
230321_AKo_Calibration_16min-MWi_38	Standard	MaR1	500,00	478,9	96	212,0	9,20	0,98	0,06	31	724163	☑
230321_AKo_Calibration_16min-MWi_39	Standard	MaR1	500,00	490,7	98	272,0	9,20	0,98	0,06	31	707837	☑
230321_AKo_Calibration_16min-MWi_40	Standard	MaR1	500,00	492,0	98	213,2	9,20	0,98	0,05	32	694933	☑
230321_AKo_Calibration_16min-MWi_41	Standard	MaR1	1000,00	976,2	98	245,9	9,20	0,98	0,05	30	1365576	☑
230321_AKo_Calibration_16min-MWi_42	Standard	MaR1	1000,00	1011,0	101	320,8	9,20	0,98	0,06	32	1394423	☑
230321_AKo_Calibration_16min-MWi_43	Standard	MaR1	2000,00	2191,1	110	381,2	9,19	0,98	0,06	29	3493882	☑
230321_AKo_Calibration_16min-MWi_44	Standard	MaR1	2000,00	1933,0	97	371,7	9,19	0,98	0,06	30	3041767	☑
230321_AKo_Calibration_16min-MWi_45	Unknown	MaR1	N/A	1,4	N/A	5,2	9,19	0,98	0,03	28	1862	☑
Blank	Double Blank	MaR1	N/A	N/A	N/A	N/A	N/A	N/A	N/A	N/A	N/A	☑
Blank	Double Blank	MaR1	N/A	N/A	N/A	N/A	N/A	N/A	N/A	N/A	N/A	☑
Blank	Double Blank	MaR1	N/A	N/A	N/A	N/A	N/A	N/A	N/A	N/A	N/A	☑
Blank	Double Blank	MaR1	N/A	N/A	N/A	N/A	N/A	N/A	N/A	N/A	N/A	☑
Blank	Double Blank	MaR1	N/A	N/A	N/A	N/A	N/A	N/A	N/A	N/A	N/A	☑

Regression... Options

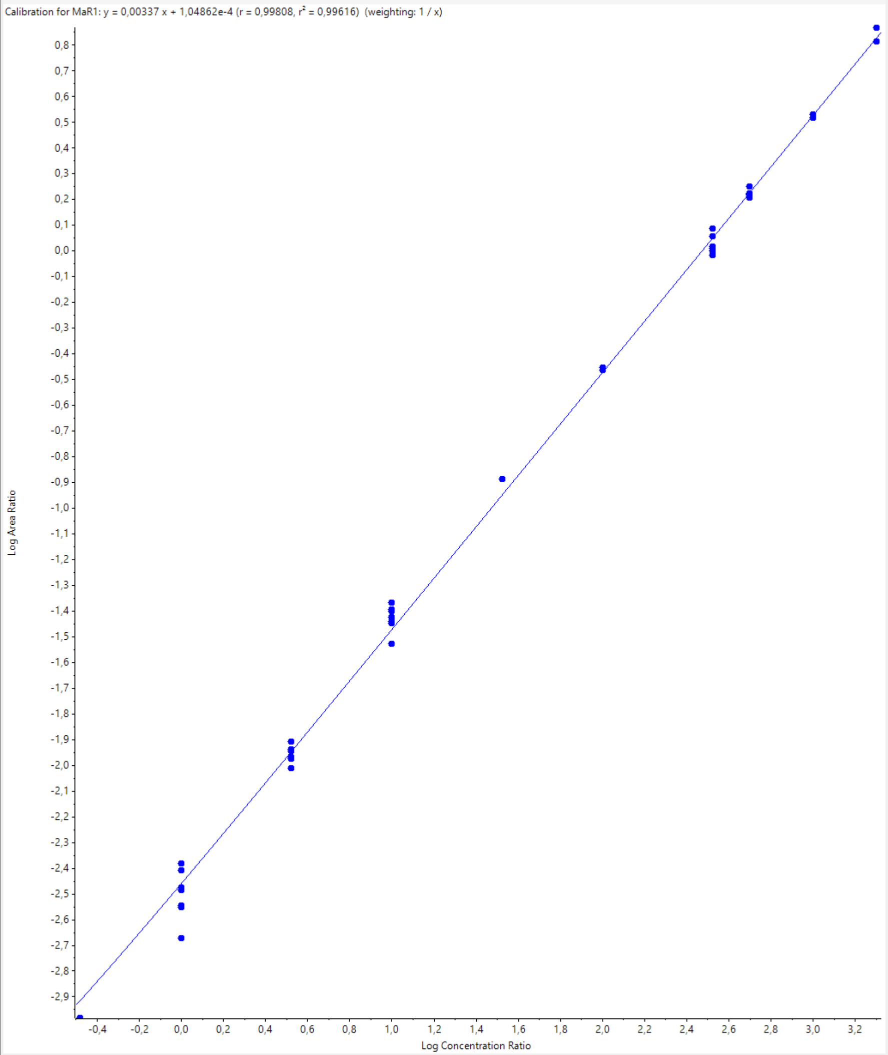

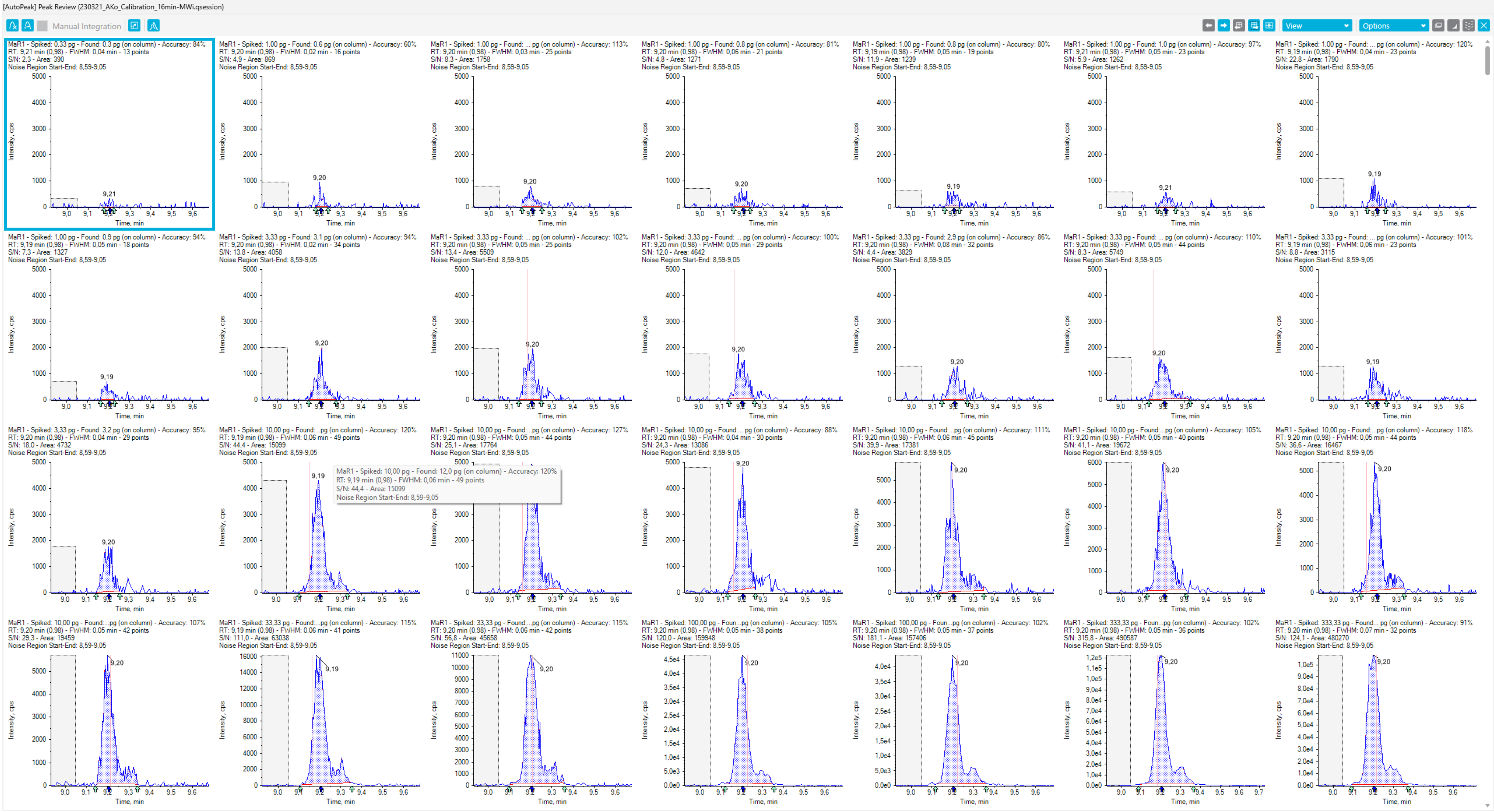
[AutoPeak] Peak Review (230321_AKo_Calibration_16min-MWi.qsession)
Manual Integration
View
Options
MaR1 - Spiked: 0,33 pg - Found: 0,3 pg (on column) - Accuracy: 84%
RT: 9,21 min (0,98) - FWHM: 0,04 min - 13 points
S/N: 2,3 - Area: 390
Noise Region Start-End: 8,59-9,05
MaR1 - Spiked: 1,00 pg - Found: 0,6 pg (on column) - Accuracy: 60%
RT: 9,20 min (0,98) - FWHM: 0,02 min - 16 points
S/N: 4,9 - Area: 869
Noise Region Start-End: 8,59-9,05
MaR1 - Spiked: 1,00 pg - Found: ... pg (on column) - Accuracy: 113%
RT: 9,20 min (0,98) - FWHM: 0,03 min - 25 points
S/N: 8,3 - Area: 1758
Noise Region Start-End: 8,59-9,05
MaR1 - Spiked: 1,00 pg - Found: 0,8 pg (on column) - Accuracy: 81%
RT: 9,20 min (0,98) - FWHM: 0,06 min - 21 points
S/N: 4,8 - Area: 1271
Noise Region Start-End: 8,59-9,05
MaR1 - Spiked: 1,00 pg - Found: 0,8 pg (on column) - Accuracy: 80%
RT: 9,19 min (0,98) - FWHM: 0,05 min - 19 points
S/N: 11,9 - Area: 1239
Noise Region Start-End: 8,59-9,05
MaR1 - Spiked: 1,00 pg - Found: 1,0 pg (on column) - Accuracy: 97%
RT: 9,21 min (0,98) - FWHM: 0,05 min - 23 points
S/N: 5,9 - Area: 1262
Noise Region Start-End: 8,59-9,05
MaR1 - Spiked: 1,00 pg - Found: ... pg (on column) - Accuracy: 120%
RT: 9,19 min (0,98) - FWHM: 0,04 min - 23 points
S/N: 22,8 - Area: 1790
Noise Region Start-End: 8,59-9,05
MaR1 - Spiked: 1,00 pg - Found: 0,9 pg (on column) - Accuracy: 94%
RT: 9,19 min (0,98) - FWHM: 0,05 min - 18 points
S/N: 7,3 - Area: 1327
Noise Region Start-End: 8,59-9,05
MaR1 - Spiked: 3,33 pg - Found: 3,1 pg (on column) - Accuracy: 94%
RT: 9,20 min (0,98) - FWHM: 0,02 min - 34 points
S/N: 13,8 - Area: 4058
Noise Region Start-End: 8,59-9,05
MaR1 - Spiked: 3,33 pg - Found: ... pg (on column) - Accuracy: 102%
RT: 9,20 min (0,98) - FWHM: 0,05 min - 25 points
S/N: 13,4 - Area: 5509
Noise Region Start-End: 8,59-9,05
MaR1 - Spiked: 3,33 pg - Found: ... pg (on column) - Accuracy: 100%
RT: 9,20 min (0,98) - FWHM: 0,05 min - 29 points
S/N: 12,0 - Area: 4642
Noise Region Start-End: 8,59-9,05
MaR1 - Spiked: 3,33 pg - Found: 2,9 pg (on column) - Accuracy: 86%
RT: 9,20 min (0,98) - FWHM: 0,08 min - 32 points
S/N: 4,4 - Area: 3829
Noise Region Start-End: 8,59-9,05
MaR1 - Spiked: 3,33 pg - Found: ... pg (on column) - Accuracy: 110%
RT: 9,20 min (0,98) - FWHM: 0,05 min - 44 points
S/N: 8,3 - Area: 5749
Noise Region Start-End: 8,59-9,05
MaR1 - Spiked: 3,33 pg - Found: ... pg (on column) - Accuracy: 101%
RT: 9,19 min (0,98) - FWHM: 0,06 min - 23 points
S/N: 8,8 - Area: 3115
Noise Region Start-End: 8,59-9,05
MaR1 - Spiked: 3,33 pg - Found: 3,2 pg (on column) - Accuracy: 95%
RT: 9,20 min (0,98) - FWHM: 0,04 min - 29 points
S/N: 18,0 - Area: 4732
Noise Region Start-End: 8,59-9,05
MaR1 - Spiked: 10,00 pg - Found:...pg (on column) - Accuracy: 120%
RT: 9,19 min (0,98) - FWHM: 0,06 min - 49 points
S/N: 44,4 - Area: 15099
Noise Region Start-End: 8,59-9,05
MaR1 - Spiked: 10,00 pg - Found: 12,0 pg (on column) - Accuracy: 120%
RT: 9,19 min (0,98) - FWHM: 0,06 min - 49 points
S/N: 44,4 - Area: 15099
Noise Region Start-End: 8,59-9,05
MaR1 - Spiked: 10,00 pg - Found:...pg (on column) - Accuracy: 127%
RT: 9,20 min (0,98) - FWHM: 0,05 min - 44 points
S/N: 25,1 - Area: 17764
Noise Region Start-End: 8,59-9,05
MaR1 - Spiked: 10,00 pg - Found:... pg (on column) - Accuracy: 88%
RT: 9,20 min (0,98) - FWHM: 0,04 min - 30 points
S/N: 24,3 - Area: 13086
Noise Region Start-End: 8,59-9,05
MaR1 - Spiked: 10,00 pg - Found:...pg (on column) - Accuracy: 111%
RT: 9,20 min (0,98) - FWHM: 0,06 min - 45 points
S/N: 39,9 - Area: 17381
Noise Region Start-End: 8,59-9,05
MaR1 - Spiked: 10,00 pg - Found:...pg (on column) - Accuracy: 105%
RT: 9,20 min (0,98) - FWHM: 0,05 min - 40 points
S/N: 41,1 - Area: 19672
Noise Region Start-End: 8,59-9,05
MaR1 - Spiked: 10,00 pg - Found:...pg (on column) - Accuracy: 118%
RT: 9,20 min (0,98) - FWHM: 0,05 min - 44 points
S/N: 36,6 - Area: 16467
Noise Region Start-End: 8,59-9,05
MaR1 - Spiked: 10,00 pg - Found:...pg (on column) - Accuracy: 107%
RT: 9,20 min (0,98) - FWHM: 0,05 min - 42 points
S/N: 29,3 - Area: 19459
Noise Region Start-End: 8,59-9,05
MaR1 - Spiked: 33,33 pg - Found:...pg (on column) - Accuracy: 115%
RT: 9,19 min (0,98) - FWHM: 0,06 min - 41 points
S/N: 111,0 - Area: 63038
Noise Region Start-End: 8,59-9,05
MaR1 - Spiked: 33,33 pg - Found:...pg (on column) - Accuracy: 115%
RT: 9,20 min (0,98) - FWHM: 0,06 min - 42 points
S/N: 56,8 - Area: 45658
Noise Region Start-End: 8,59-9,05
MaR1 - Spiked: 100,00 pg - Foun...pg (on column) - Accuracy: 105%
RT: 9,20 min (0,98) - FWHM: 0,05 min - 38 points
S/N: 120,0 - Area: 159948
Noise Region Start-End: 8,59-9,05
MaR1 - Spiked: 100,00 pg - Foun...pg (on column) - Accuracy: 102%
RT: 9,20 min (0,98) - FWHM: 0,05 min - 37 points
S/N: 181,1 - Area: 157406
Noise Region Start-End: 8,59-9,05
MaR1 - Spiked: 333,33 pg - Foun...pg (on column) - Accuracy: 102%
RT: 9,20 min (0,98) - FWHM: 0,05 min - 36 points
S/N: 315,8 - Area: 490587
Noise Region Start-End: 8,59-9,05
MaR1 - Spiked: 333,33 pg - Found... pg (on column) - Accuracy: 91%
RT: 9,20 min (0,98) - FWHM: 0,07 min - 32 points
S/N: 124,1 - Area: 480270
Noise Region Start-End: 8,59-9,05
Intensity, cps
Time, min

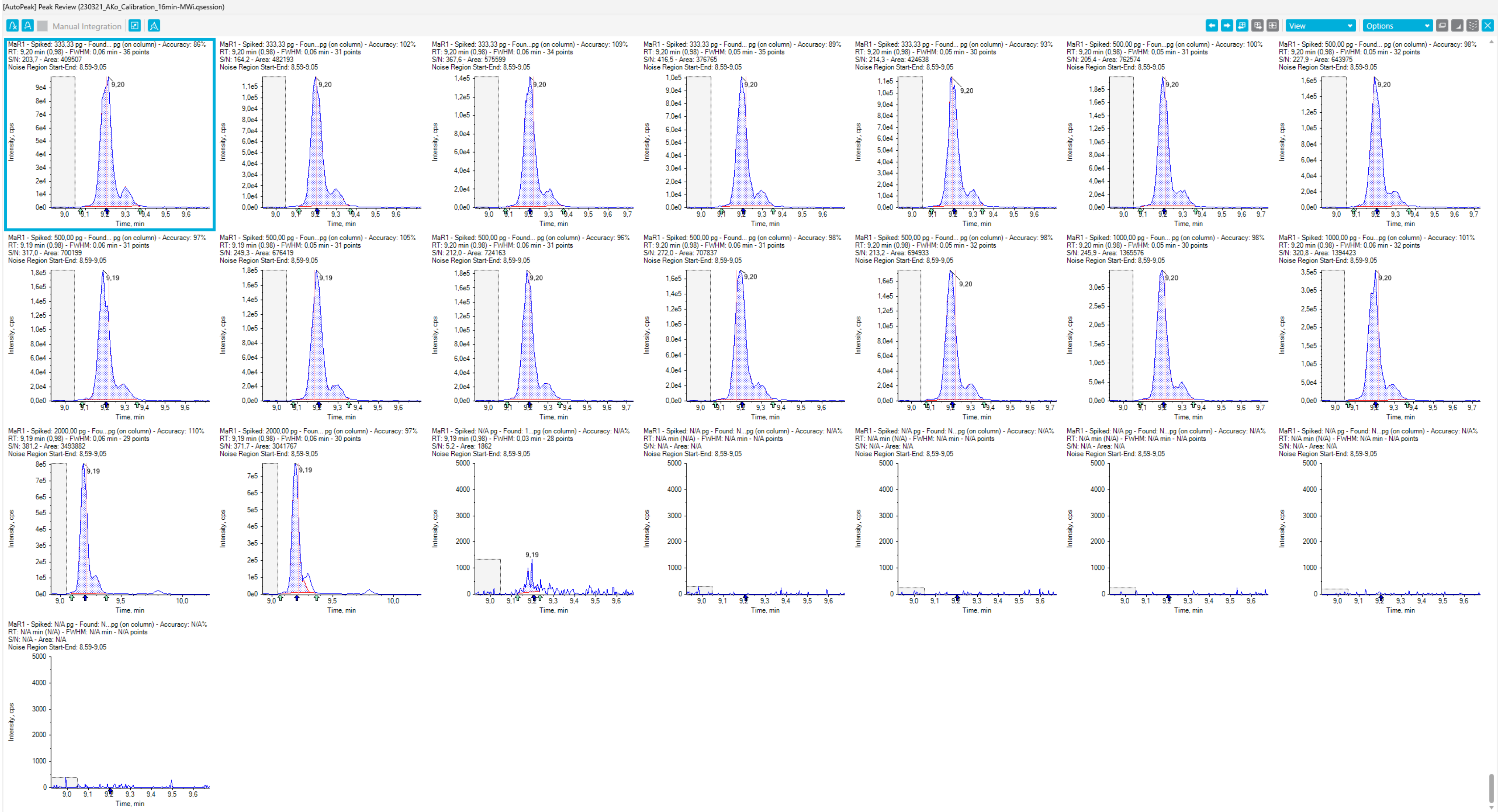
[AutoPeak] Peak Review (230321_AKo_Calibration_16min-MWi.qsession)
Manual Integration
View
Options
MaR1 - Spiked: 333,33 pg - Found... pg (on column) - Accuracy: 86%
RT: 9,20 min (0,98) - FWHM: 0,06 min - 36 points
S/N: 203,7 - Area: 409507
Noise Region Start-End: 8,59-9,05
MaR1 - Spiked: 333,33 pg - Foun...pg (on column) - Accuracy: 102%
RT: 9,20 min (0,98) - FWHM: 0,06 min - 31 points
S/N: 164,2 - Area: 482193
MaR1 - Spiked: 333,33 pg - Foun...pg (on column) - Accuracy: 109%
RT: 9,20 min (0,98) - FWHM: 0,06 min - 34 points
S/N: 367,6 - Area: 575599
MaR1 - Spiked: 333,33 pg - Found... pg (on column) - Accuracy: 89%
RT: 9,20 min (0,98) - FWHM: 0,05 min - 35 points
S/N: 416,5 - Area: 376765
MaR1 - Spiked: 333,33 pg - Found... pg (on column) - Accuracy: 93%
RT: 9,20 min (0,98) - FWHM: 0,05 min - 30 points
S/N: 214,3 - Area: 424638
MaR1 - Spiked: 500,00 pg - Foun...pg (on column) - Accuracy: 100%
RT: 9,20 min (0,98) - FWHM: 0,05 min - 31 points
S/N: 205,4 - Area: 762574
MaR1 - Spiked: 500,00 pg - Found... pg (on column) - Accuracy: 98%
RT: 9,20 min (0,98) - FWHM: 0,05 min - 32 points
S/N: 227,9 - Area: 643975
MaR1 - Spiked: 500,00 pg - Found... pg (on column) - Accuracy: 97%
RT: 9,19 min (0,98) - FWHM: 0,06 min - 31 points
S/N: 317,0 - Area: 700199
MaR1 - Spiked: 500,00 pg - Foun...pg (on column) - Accuracy: 105%
RT: 9,19 min (0,98) - FWHM: 0,05 min - 31 points
S/N: 249,3 - Area: 676419
MaR1 - Spiked: 500,00 pg - Found... pg (on column) - Accuracy: 96%
RT: 9,20 min (0,98) - FWHM: 0,06 min - 31 points
S/N: 212,0 - Area: 724163
MaR1 - Spiked: 500,00 pg - Found... pg (on column) - Accuracy: 98%
RT: 9,20 min (0,98) - FWHM: 0,06 min - 31 points
S/N: 272,0 - Area: 707837
MaR1 - Spiked: 500,00 pg - Found... pg (on column) - Accuracy: 98%
RT: 9,20 min (0,98) - FWHM: 0,05 min - 32 points
S/N: 213,2 - Area: 694933
MaR1 - Spiked: 1000,00 pg - Foun... pg (on column) - Accuracy: 98%
RT: 9,20 min (0,98) - FWHM: 0,05 min - 30 points
S/N: 245,9 - Area: 1365576
MaR1 - Spiked: 1000,00 pg - Fou...pg (on column) - Accuracy: 101%
RT: 9,20 min (0,98) - FWHM: 0,06 min - 32 points
S/N: 320,8 - Area: 1394423
MaR1 - Spiked: 2000,00 pg - Fou...pg (on column) - Accuracy: 110%
RT: 9,19 min (0,98) - FWHM: 0,06 min - 29 points
S/N: 381,2 - Area: 3493882
MaR1 - Spiked: 2000,00 pg - Foun... pg (on column) - Accuracy: 97%
RT: 9,19 min (0,98) - FWHM: 0,06 min - 30 points
S/N: 371,7 - Area: 3041767
MaR1 - Spiked: N/A pg - Found: 1...pg (on column) - Accuracy: N/A%
RT: 9,19 min (0,98) - FWHM: 0,03 min - 28 points
S/N: 5,2 - Area: 1862
MaR1 - Spiked: N/A pg - Found: N...pg (on column) - Accuracy: N/A%
RT: N/A min (N/A) - FWHM: N/A min - N/A points
S/N: N/A - Area: N/A
Intensity, cps
Time, min

MaR2

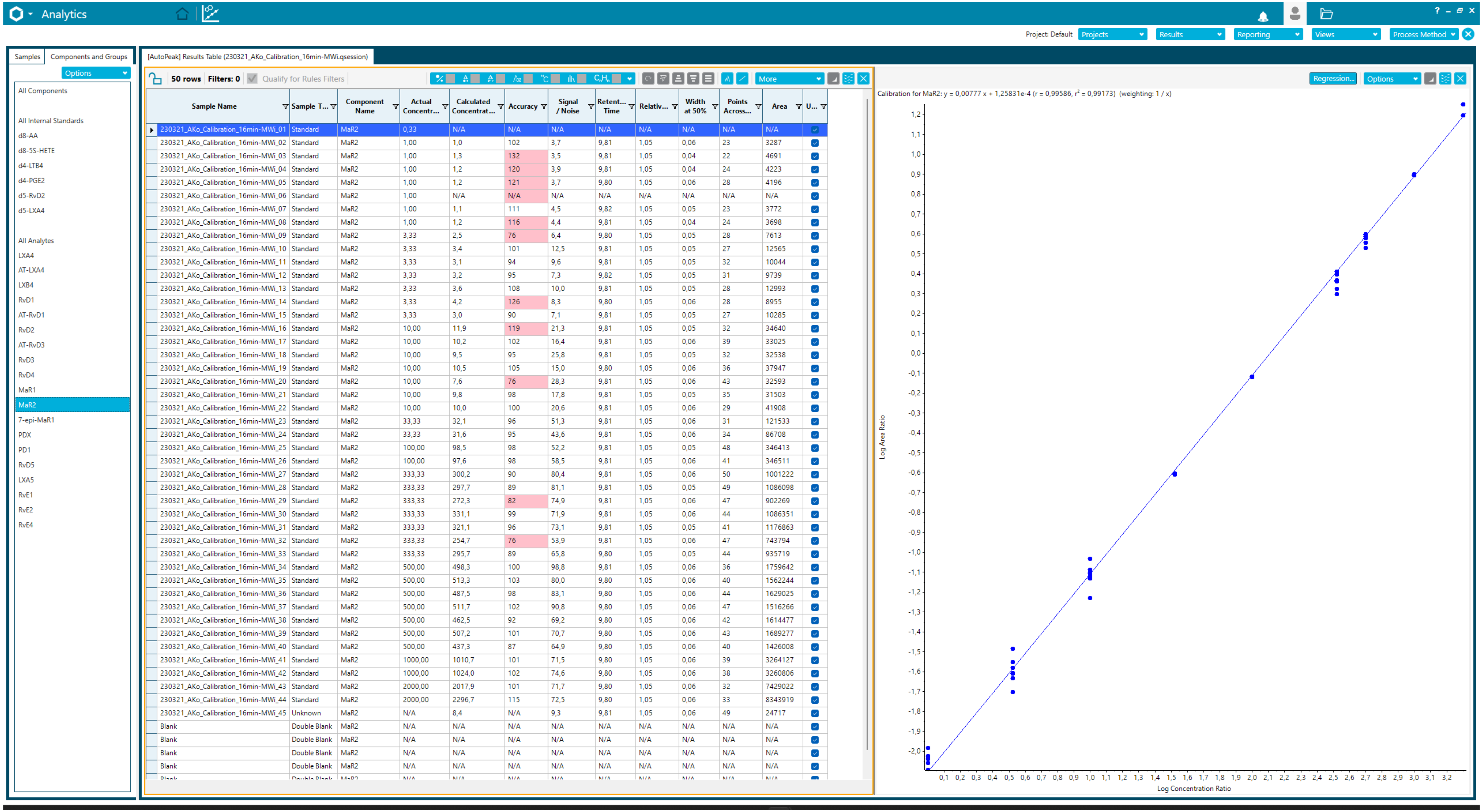

Sample Name	Sample T...	Component Name	Actual Concentr...	Calculated Concentrat...	Accuracy	Signal / Noise	Retent... Time	Relativ...	Width at 50%	Points Across...	Area	U...
230321_AKo_Calibration_16min-MWi_01	Standard	MaR2	0,33	N/A	N/A	N/A	N/A	N/A	N/A	N/A	N/A	☑
230321_AKo_Calibration_16min-MWi_02	Standard	MaR2	1,00	1,0	102	3,7	9,81	1,05	0,06	23	3287	☑
230321_AKo_Calibration_16min-MWi_03	Standard	MaR2	1,00	1,3	132	3,5	9,81	1,05	0,04	22	4691	☑
230321_AKo_Calibration_16min-MWi_04	Standard	MaR2	1,00	1,2	120	3,9	9,81	1,05	0,04	24	4223	☑
230321_AKo_Calibration_16min-MWi_05	Standard	MaR2	1,00	1,2	121	3,7	9,80	1,05	0,06	28	4196	☑
230321_AKo_Calibration_16min-MWi_06	Standard	MaR2	1,00	N/A	N/A	N/A	N/A	N/A	N/A	N/A	N/A	☑
230321_AKo_Calibration_16min-MWi_07	Standard	MaR2	1,00	1,1	111	4,5	9,82	1,05	0,05	23	3772	☑
230321_AKo_Calibration_16min-MWi_08	Standard	MaR2	1,00	1,2	116	4,4	9,81	1,05	0,04	24	3698	☑
230321_AKo_Calibration_16min-MWi_09	Standard	MaR2	3,33	2,5	76	6,4	9,80	1,05	0,05	28	7613	☑
230321_AKo_Calibration_16min-MWi_10	Standard	MaR2	3,33	3,4	101	12,5	9,81	1,05	0,05	27	12565	☑
230321_AKo_Calibration_16min-MWi_11	Standard	MaR2	3,33	3,1	94	9,6	9,81	1,05	0,05	32	10044	☑
230321_AKo_Calibration_16min-MWi_12	Standard	MaR2	3,33	3,2	95	7,3	9,82	1,05	0,05	31	9739	☑
230321_AKo_Calibration_16min-MWi_13	Standard	MaR2	3,33	3,6	108	10,0	9,81	1,05	0,05	28	12993	☑
230321_AKo_Calibration_16min-MWi_14	Standard	MaR2	3,33	4,2	126	8,3	9,80	1,05	0,06	28	8955	☑
230321_AKo_Calibration_16min-MWi_15	Standard	MaR2	3,33	3,0	90	7,1	9,81	1,05	0,05	27	10285	☑
230321_AKo_Calibration_16min-MWi_16	Standard	MaR2	10,00	11,9	119	21,3	9,81	1,05	0,05	32	34640	☑
230321_AKo_Calibration_16min-MWi_17	Standard	MaR2	10,00	10,2	102	16,4	9,81	1,05	0,06	39	33025	☑
230321_AKo_Calibration_16min-MWi_18	Standard	MaR2	10,00	9,5	95	25,8	9,81	1,05	0,05	32	32538	☑
230321_AKo_Calibration_16min-MWi_19	Standard	MaR2	10,00	10,5	105	15,0	9,80	1,05	0,06	36	37947	☑
230321_AKo_Calibration_16min-MWi_20	Standard	MaR2	10,00	7,6	76	28,3	9,81	1,05	0,06	43	32593	☑
230321_AKo_Calibration_16min-MWi_21	Standard	MaR2	10,00	9,8	98	17,8	9,81	1,05	0,05	35	31503	☑
230321_AKo_Calibration_16min-MWi_22	Standard	MaR2	10,00	10,0	100	20,6	9,81	1,05	0,06	29	41908	☑
230321_AKo_Calibration_16min-MWi_23	Standard	MaR2	33,33	32,1	96	51,3	9,81	1,05	0,06	31	121533	☑
230321_AKo_Calibration_16min-MWi_24	Standard	MaR2	33,33	31,6	95	43,6	9,81	1,05	0,06	34	86708	☑
230321_AKo_Calibration_16min-MWi_25	Standard	MaR2	100,00	98,5	98	52,2	9,81	1,05	0,05	48	346413	☑
230321_AKo_Calibration_16min-MWi_26	Standard	MaR2	100,00	97,6	98	58,5	9,81	1,05	0,06	41	346511	☑
230321_AKo_Calibration_16min-MWi_27	Standard	MaR2	333,33	300,2	90	80,4	9,81	1,05	0,06	50	1001222	☑
230321_AKo_Calibration_16min-MWi_28	Standard	MaR2	333,33	297,7	89	81,1	9,81	1,05	0,05	49	1086098	☑
230321_AKo_Calibration_16min-MWi_29	Standard	MaR2	333,33	272,3	82	74,9	9,81	1,05	0,06	47	902269	☑
230321_AKo_Calibration_16min-MWi_30	Standard	MaR2	333,33	331,1	99	71,9	9,81	1,05	0,06	44	1086351	☑
230321_AKo_Calibration_16min-MWi_31	Standard	MaR2	333,33	321,1	96	73,1	9,81	1,05	0,05	41	1176863	☑
230321_AKo_Calibration_16min-MWi_32	Standard	MaR2	333,33	254,7	76	53,9	9,81	1,05	0,06	47	743794	☑
230321_AKo_Calibration_16min-MWi_33	Standard	MaR2	333,33	295,7	89	65,8	9,80	1,05	0,05	44	935719	☑
230321_AKo_Calibration_16min-MWi_34	Standard	MaR2	500,00	498,3	100	98,8	9,81	1,05	0,06	36	1759642	☑
230321_AKo_Calibration_16min-MWi_35	Standard	MaR2	500,00	513,3	103	80,0	9,80	1,05	0,06	40	1562244	☑
230321_AKo_Calibration_16min-MWi_36	Standard	MaR2	500,00	487,5	98	83,1	9,80	1,05	0,06	44	1629025	☑
230321_AKo_Calibration_16min-MWi_37	Standard	MaR2	500,00	511,7	102	90,8	9,80	1,05	0,06	47	1516266	☑
230321_AKo_Calibration_16min-MWi_38	Standard	MaR2	500,00	462,5	92	69,2	9,80	1,05	0,06	42	1614477	☑
230321_AKo_Calibration_16min-MWi_39	Standard	MaR2	500,00	507,2	101	70,7	9,80	1,05	0,06	43	1689277	☑
230321_AKo_Calibration_16min-MWi_40	Standard	MaR2	500,00	437,3	87	64,9	9,80	1,05	0,06	40	1426008	☑
230321_AKo_Calibration_16min-MWi_41	Standard	MaR2	1000,00	1010,7	101	71,5	9,80	1,05	0,06	39	3264127	☑
230321_AKo_Calibration_16min-MWi_42	Standard	MaR2	1000,00	1024,0	102	74,6	9,80	1,05	0,06	38	3260806	☑
230321_AKo_Calibration_16min-MWi_43	Standard	MaR2	2000,00	2017,9	101	71,7	9,80	1,05	0,06	32	7429022	☑
230321_AKo_Calibration_16min-MWi_44	Standard	MaR2	2000,00	2296,7	115	72,5	9,80	1,05	0,06	33	8343919	☑
230321_AKo_Calibration_16min-MWi_45	Unknown	MaR2	N/A	8,4	N/A	9,3	9,81	1,05	0,06	49	24717	☑
Blank	Double Blank	MaR2	N/A	N/A	N/A	N/A	N/A	N/A	N/A	N/A	N/A	☑
Blank	Double Blank	MaR2	N/A	N/A	N/A	N/A	N/A	N/A	N/A	N/A	N/A	☑
Blank	Double Blank	MaR2	N/A	N/A	N/A	N/A	N/A	N/A	N/A	N/A	N/A	☑
Blank	Double Blank	MaR2	N/A	N/A	N/A	N/A	N/A	N/A	N/A	N/A	N/A	☑
Blank	Double Blank	MaR2	N/A	N/A	N/A	N/A	N/A	N/A	N/A	N/A	N/A	☑

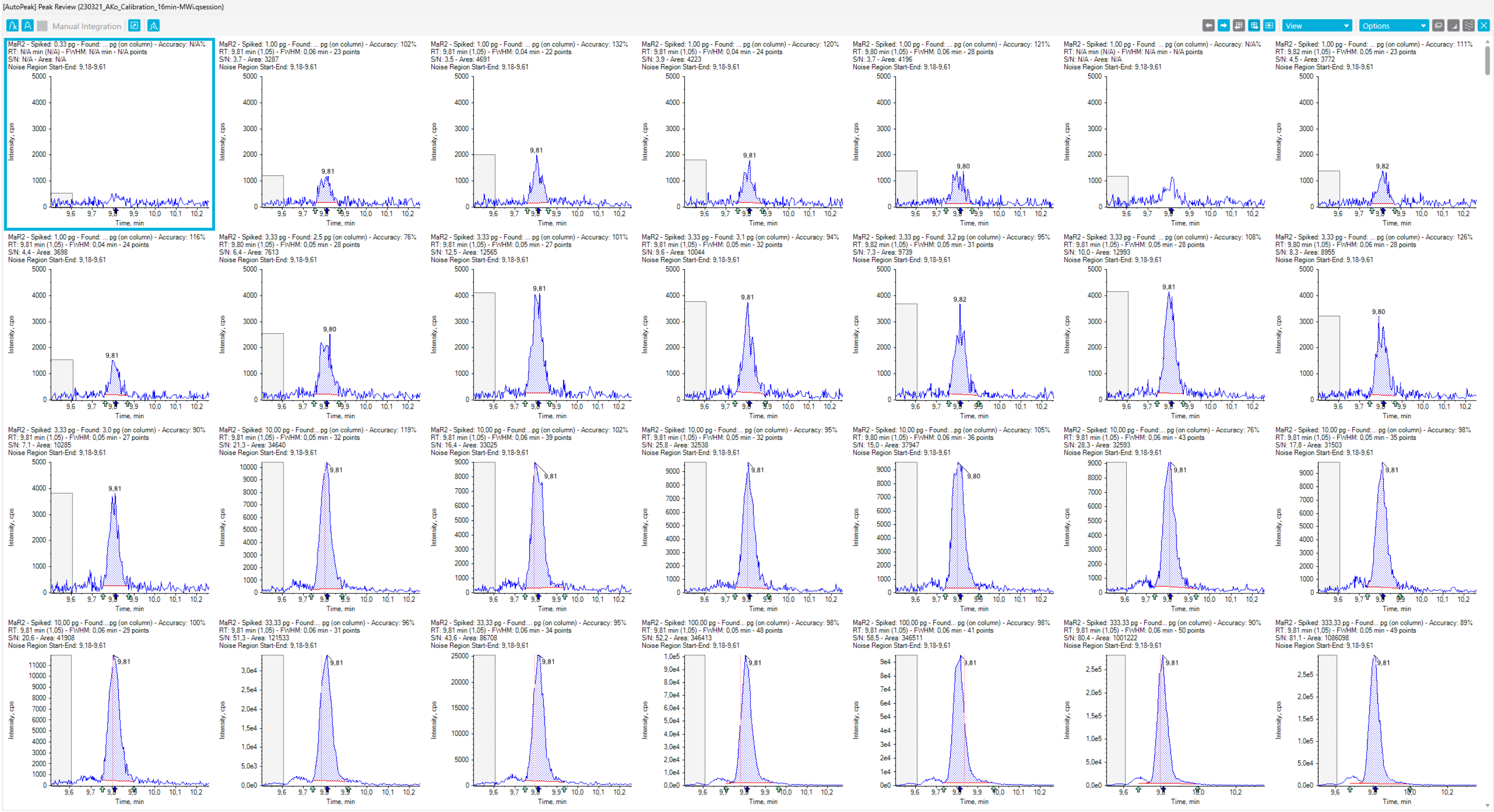
[AutoPeak] Peak Review (230321_AKo_Calibration_16min-MWi.qsession)
Manual Integration
View
Options
MaR2 - Spiked: 0,33 pg - Found: ... pg (on column) - Accuracy: N/A% RT: N/A min (N/A) - FWHM: N/A min - N/A points S/N: N/A - Area: N/A Noise Region Start-End: 9,18-9,61
MaR2 - Spiked: 1,00 pg - Found: ... pg (on column) - Accuracy: 102% RT: 9,81 min (1,05) - FWHM: 0,06 min - 23 points S/N: 3,7 - Area: 3287 Noise Region Start-End: 9,18-9,61
MaR2 - Spiked: 1,00 pg - Found: ... pg (on column) - Accuracy: 132% RT: 9,81 min (1,05) - FWHM: 0,04 min - 22 points S/N: 3,5 - Area: 4691 Noise Region Start-End: 9,18-9,61
MaR2 - Spiked: 1,00 pg - Found: ... pg (on column) - Accuracy: 120% RT: 9,81 min (1,05) - FWHM: 0,04 min - 24 points S/N: 3,9 - Area: 4223 Noise Region Start-End: 9,18-9,61
MaR2 - Spiked: 1,00 pg - Found: ... pg (on column) - Accuracy: 121% RT: 9,80 min (1,05) - FWHM: 0,06 min - 28 points S/N: 3,7 - Area: 4196 Noise Region Start-End: 9,18-9,61
MaR2 - Spiked: 1,00 pg - Found: ... pg (on column) - Accuracy: N/A% RT: N/A min (N/A) - FWHM: N/A min - N/A points S/N: N/A - Area: N/A Noise Region Start-End: 9,18-9,61
MaR2 - Spiked: 1,00 pg - Found: ... pg (on column) - Accuracy: 111% RT: 9,82 min (1,05) - FWHM: 0,05 min - 23 points S/N: 4,5 - Area: 3772 Noise Region Start-End: 9,18-9,61
MaR2 - Spiked: 1,00 pg - Found: ... pg (on column) - Accuracy: 116% RT: 9,81 min (1,05) - FWHM: 0,04 min - 24 points S/N: 4,4 - Area: 3698 Noise Region Start-End: 9,18-9,61
MaR2 - Spiked: 3,33 pg - Found: 2,5 pg (on column) - Accuracy: 76% RT: 9,80 min (1,05) - FWHM: 0,05 min - 28 points S/N: 6,4 - Area: 7613 Noise Region Start-End: 9,18-9,61
MaR2 - Spiked: 3,33 pg - Found: ... pg (on column) - Accuracy: 101% RT: 9,81 min (1,05) - FWHM: 0,05 min - 27 points S/N: 12,5 - Area: 12565 Noise Region Start-End: 9,18-9,61
MaR2 - Spiked: 3,33 pg - Found: 3,1 pg (on column) - Accuracy: 94% RT: 9,81 min (1,05) - FWHM: 0,05 min - 32 points S/N: 9,6 - Area: 10044 Noise Region Start-End: 9,18-9,61
MaR2 - Spiked: 3,33 pg - Found: 3,2 pg (on column) - Accuracy: 95% RT: 9,82 min (1,05) - FWHM: 0,05 min - 31 points S/N: 7,3 - Area: 9739 Noise Region Start-End: 9,18-9,61
MaR2 - Spiked: 3,33 pg - Found: ... pg (on column) - Accuracy: 108% RT: 9,81 min (1,05) - FWHM: 0,05 min - 28 points S/N: 10,0 - Area: 12993 Noise Region Start-End: 9,18-9,61
MaR2 - Spiked: 3,33 pg - Found: ... pg (on column) - Accuracy: 126% RT: 9,80 min (1,05) - FWHM: 0,06 min - 28 points S/N: 8,3 - Area: 8955 Noise Region Start-End: 9,18-9,61
MaR2 - Spiked: 3,33 pg - Found: 3,0 pg (on column) - Accuracy: 90% RT: 9,81 min (1,05) - FWHM: 0,05 min - 27 points S/N: 7,1 - Area: 10285 Noise Region Start-End: 9,18-9,61
MaR2 - Spiked: 10,00 pg - Found:...pg (on column) - Accuracy: 119% RT: 9,81 min (1,05) - FWHM: 0,05 min - 32 points S/N: 21,3 - Area: 34640 Noise Region Start-End: 9,18-9,61
MaR2 - Spiked: 10,00 pg - Found:...pg (on column) - Accuracy: 102% RT: 9,81 min (1,05) - FWHM: 0,06 min - 39 points S/N: 16,4 - Area: 33025 Noise Region Start-End: 9,18-9,61
MaR2 - Spiked: 10,00 pg - Found:... pg (on column) - Accuracy: 95% RT: 9,81 min (1,05) - FWHM: 0,05 min - 32 points S/N: 25,8 - Area: 32538 Noise Region Start-End: 9,18-9,61
MaR2 - Spiked: 10,00 pg - Found:...pg (on column) - Accuracy: 105% RT: 9,80 min (1,05) - FWHM: 0,06 min - 36 points S/N: 15,0 - Area: 37947 Noise Region Start-End: 9,18-9,61
MaR2 - Spiked: 10,00 pg - Found:... pg (on column) - Accuracy: 76% RT: 9,81 min (1,05) - FWHM: 0,06 min - 43 points S/N: 28,3 - Area: 32593 Noise Region Start-End: 9,18-9,61
MaR2 - Spiked: 10,00 pg - Found:... pg (on column) - Accuracy: 98% RT: 9,81 min (1,05) - FWHM: 0,05 min - 35 points S/N: 17,8 - Area: 31503 Noise Region Start-End: 9,18-9,61
MaR2 - Spiked: 10,00 pg - Found:...pg (on column) - Accuracy: 100% RT: 9,81 min (1,05) - FWHM: 0,06 min - 29 points S/N: 20,6 - Area: 41908 Noise Region Start-End: 9,18-9,61
MaR2 - Spiked: 33,33 pg - Found:... pg (on column) - Accuracy: 96% RT: 9,81 min (1,05) - FWHM: 0,06 min - 31 points S/N: 51,3 - Area: 121533 Noise Region Start-End: 9,18-9,61
MaR2 - Spiked: 33,33 pg - Found:... pg (on column) - Accuracy: 95% RT: 9,81 min (1,05) - FWHM: 0,06 min - 34 points S/N: 43,6 - Area: 86708 Noise Region Start-End: 9,18-9,61
MaR2 - Spiked: 100,00 pg - Found:... pg (on column) - Accuracy: 98% RT: 9,81 min (1,05) - FWHM: 0,05 min - 48 points S/N: 52,2 - Area: 346413 Noise Region Start-End: 9,18-9,61
MaR2 - Spiked: 100,00 pg - Found... pg (on column) - Accuracy: 98% RT: 9,81 min (1,05) - FWHM: 0,06 min - 41 points S/N: 58,5 - Area: 346511 Noise Region Start-End: 9,18-9,61
MaR2 - Spiked: 333,33 pg - Found... pg (on column) - Accuracy: 90% RT: 9,81 min (1,05) - FWHM: 0,06 min - 50 points S/N: 80,4 - Area: 1001222 Noise Region Start-End: 9,18-9,61
MaR2 - Spiked: 333,33 pg - Found... pg (on column) - Accuracy: 89% RT: 9,81 min (1,05) - FWHM: 0,05 min - 49 points S/N: 81,1 - Area: 1086098 Noise Region Start-End: 9,18-9,61
Intensity, cps
Time, min

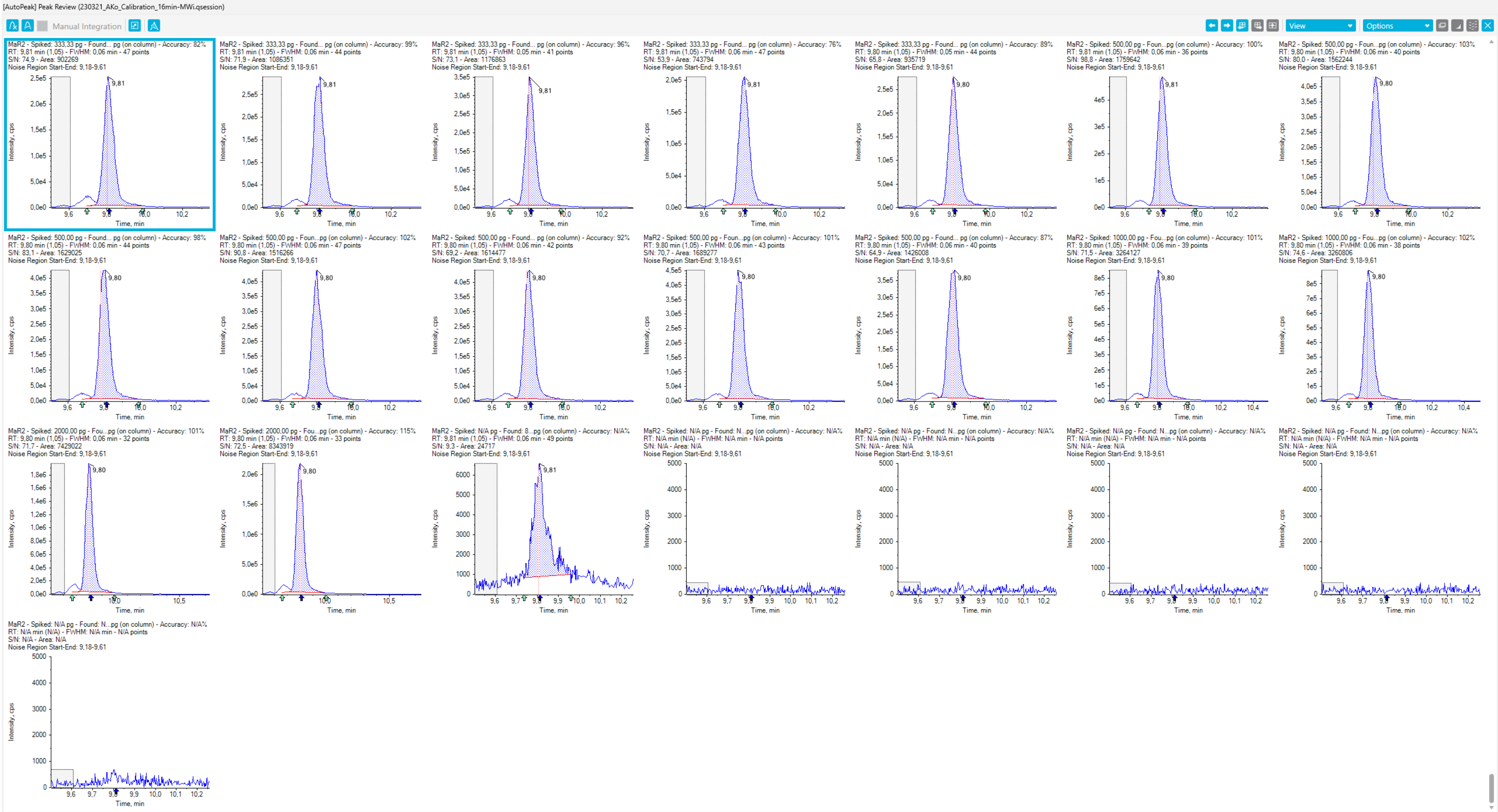

7-*epi*-MaR1

[AutoPeak] Results Table (230321_AKo_Calibration_16min-MWi.qsession)

50 rows Filters: 0 Qualify for Rules Filters

Sample Name	Sample T...	Component Name	Actual Concentr...	Calculated Concentrat...	Accuracy	Signal / Noise	Retent... Time	Relativ...	Width at 50%	Points Across...	Area	U...
230321_AKo_Calibration_16min-MWi_01	Standard	7-epi-MaR1	0,33	N/A	N/A	N/A	N/A	N/A	N/A	N/A	N/A	☑
230321_AKo_Calibration_16min-MWi_02	Standard	7-epi-MaR1	1,00	0,4	41	2,8	8,56	0,92	0,04	19	761	☑
230321_AKo_Calibration_16min-MWi_03	Standard	7-epi-MaR1	1,00	0,8	80	2,1	8,56	0,92	0,08	30	1317	☑
230321_AKo_Calibration_16min-MWi_04	Standard	7-epi-MaR1	1,00	0,9	94	7,8	8,55	0,92	0,05	30	1473	☑
230321_AKo_Calibration_16min-MWi_05	Standard	7-epi-MaR1	1,00	0,8	75	5,2	8,55	0,91	0,06	26	1227	☑
230321_AKo_Calibration_16min-MWi_06	Standard	7-epi-MaR1	1,00	0,9	87	5,6	8,56	0,92	0,03	20	1166	☑
230321_AKo_Calibration_16min-MWi_07	Standard	7-epi-MaR1	1,00	0,7	73	3,7	8,55	0,91	0,03	28	1181	☑
230321_AKo_Calibration_16min-MWi_08	Standard	7-epi-MaR1	1,00	1,0	100	7,8	8,56	0,91	0,04	31	1389	☑
230321_AKo_Calibration_16min-MWi_09	Standard	7-epi-MaR1	3,33	3,2	95	7,2	8,57	0,92	0,06	38	3535	☑
230321_AKo_Calibration_16min-MWi_10	Standard	7-epi-MaR1	3,33	3,8	113	21,7	8,56	0,92	0,04	45	5190	☑
230321_AKo_Calibration_16min-MWi_11	Standard	7-epi-MaR1	3,33	3,2	97	N/A	8,56	0,92	0,06	31	3851	☑
230321_AKo_Calibration_16min-MWi_12	Standard	7-epi-MaR1	3,33	4,3	129	8,3	8,57	0,92	0,05	41	4837	☑
230321_AKo_Calibration_16min-MWi_13	Standard	7-epi-MaR1	3,33	3,3	99	22,2	8,55	0,92	0,05	43	4444	☑
230321_AKo_Calibration_16min-MWi_14	Standard	7-epi-MaR1	3,33	4,0	122	4,4	8,57	0,92	0,06	38	3184	☑
230321_AKo_Calibration_16min-MWi_15	Standard	7-epi-MaR1	3,33	3,2	97	24,3	8,56	0,92	0,02	31	4146	☑
230321_AKo_Calibration_16min-MWi_16	Standard	7-epi-MaR1	10,00	10,6	106	87,0	8,56	0,92	0,05	44	10945	☑
230321_AKo_Calibration_16min-MWi_17	Standard	7-epi-MaR1	10,00	12,5	125	27,4	8,56	0,92	0,05	47	14300	☑
230321_AKo_Calibration_16min-MWi_18	Standard	7-epi-MaR1	10,00	12,4	124	35,8	8,56	0,92	0,06	53	15050	☑
230321_AKo_Calibration_16min-MWi_19	Standard	7-epi-MaR1	10,00	11,9	119	31,6	8,56	0,92	0,06	48	15091	☑
230321_AKo_Calibration_16min-MWi_20	Standard	7-epi-MaR1	10,00	10,2	102	19,5	8,56	0,92	0,05	50	15624	☑
230321_AKo_Calibration_16min-MWi_21	Standard	7-epi-MaR1	10,00	11,7	117	35,2	8,56	0,92	0,05	52	13303	☑
230321_AKo_Calibration_16min-MWi_22	Standard	7-epi-MaR1	10,00	11,5	115	63,6	8,56	0,92	0,05	43	16924	☑
230321_AKo_Calibration_16min-MWi_23	Standard	7-epi-MaR1	33,33	37,4	112	79,2	8,56	0,92	0,05	44	49218	☑
230321_AKo_Calibration_16min-MWi_24	Standard	7-epi-MaR1	33,33	39,5	118	58,1	8,57	0,92	0,05	51	37613	☑
230321_AKo_Calibration_16min-MWi_25	Standard	7-epi-MaR1	100,00	107,3	107	258,8	8,57	0,92	0,05	38	130711	☑
230321_AKo_Calibration_16min-MWi_26	Standard	7-epi-MaR1	100,00	98,8	99	267,5	8,56	0,92	0,05	37	121550	☑
230321_AKo_Calibration_16min-MWi_27	Standard	7-epi-MaR1	333,33	302,3	91	720,2	8,57	0,92	0,05	26	348645	☑
230321_AKo_Calibration_16min-MWi_28	Standard	7-epi-MaR1	333,33	312,6	94	2474,4	8,56	0,92	0,05	22	394353	☑
230321_AKo_Calibration_16min-MWi_29	Standard	7-epi-MaR1	333,33	281,2	84	605,8	8,57	0,92	0,06	29	322245	☑
230321_AKo_Calibration_16min-MWi_30	Standard	7-epi-MaR1	333,33	350,6	105	2428,4	8,57	0,92	0,05	26	397681	☑
230321_AKo_Calibration_16min-MWi_31	Standard	7-epi-MaR1	333,33	340,6	102	N/A	8,57	0,92	0,05	24	431689	☑
230321_AKo_Calibration_16min-MWi_32	Standard	7-epi-MaR1	333,33	318,4	96	679,5	8,56	0,92	0,05	28	321466	☑
230321_AKo_Calibration_16min-MWi_33	Standard	7-epi-MaR1	333,33	314,9	94	1923,9	8,56	0,92	0,06	28	344480	☑
230321_AKo_Calibration_16min-MWi_34	Standard	7-epi-MaR1	500,00	516,7	103	1234,7	8,56	0,92	0,06	26	630712	☑
230321_AKo_Calibration_16min-MWi_35	Standard	7-epi-MaR1	500,00	483,0	97	1070,1	8,57	0,92	0,05	26	508192	☑
230321_AKo_Calibration_16min-MWi_36	Standard	7-epi-MaR1	500,00	507,8	102	640,2	8,56	0,92	0,05	23	586522	☑
230321_AKo_Calibration_16min-MWi_37	Standard	7-epi-MaR1	500,00	488,6	98	2828,4	8,56	0,92	0,06	25	500402	☑
230321_AKo_Calibration_16min-MWi_38	Standard	7-epi-MaR1	500,00	466,0	93	N/A	8,55	0,91	0,05	25	562351	☑
230321_AKo_Calibration_16min-MWi_39	Standard	7-epi-MaR1	500,00	480,0	96	N/A	8,56	0,92	0,06	23	552567	☑
230321_AKo_Calibration_16min-MWi_40	Standard	7-epi-MaR1	500,00	469,4	94	3025,9	8,56	0,92	0,06	23	529168	☑
230321_AKo_Calibration_16min-MWi_41	Standard	7-epi-MaR1	1000,00	950,1	95	1916,6	8,56	0,92	0,06	23	1060315	☑
230321_AKo_Calibration_16min-MWi_42	Standard	7-epi-MaR1	1000,00	951,7	95	2036,4	8,55	0,92	0,06	24	1047285	☑
230321_AKo_Calibration_16min-MWi_43	Standard	7-epi-MaR1	2000,00	2102,2	105	7525,3	8,55	0,92	0,06	24	2674108	☑
230321_AKo_Calibration_16min-MWi_44	Standard	7-epi-MaR1	2000,00	2170,0	108	N/A	8,55	0,92	0,05	23	2723932	☑
230321_AKo_Calibration_16min-MWi_45	Unknown	7-epi-MaR1	N/A	1,0	N/A	3,5	8,55	0,91	0,05	33	1310	☑
Blank	Double Blank	7-epi-MaR1	N/A	N/A	N/A	N/A	N/A	N/A	N/A	N/A	N/A	☑
Blank	Double Blank	7-epi-MaR1	N/A	N/A	N/A	N/A	N/A	N/A	N/A	N/A	N/A	☑
Blank	Double Blank	7-epi-MaR1	N/A	N/A	N/A	N/A	N/A	N/A	N/A	N/A	N/A	☑
Blank	Double Blank	7-epi-MaR1	N/A	N/A	N/A	N/A	N/A	N/A	N/A	N/A	N/A	☑

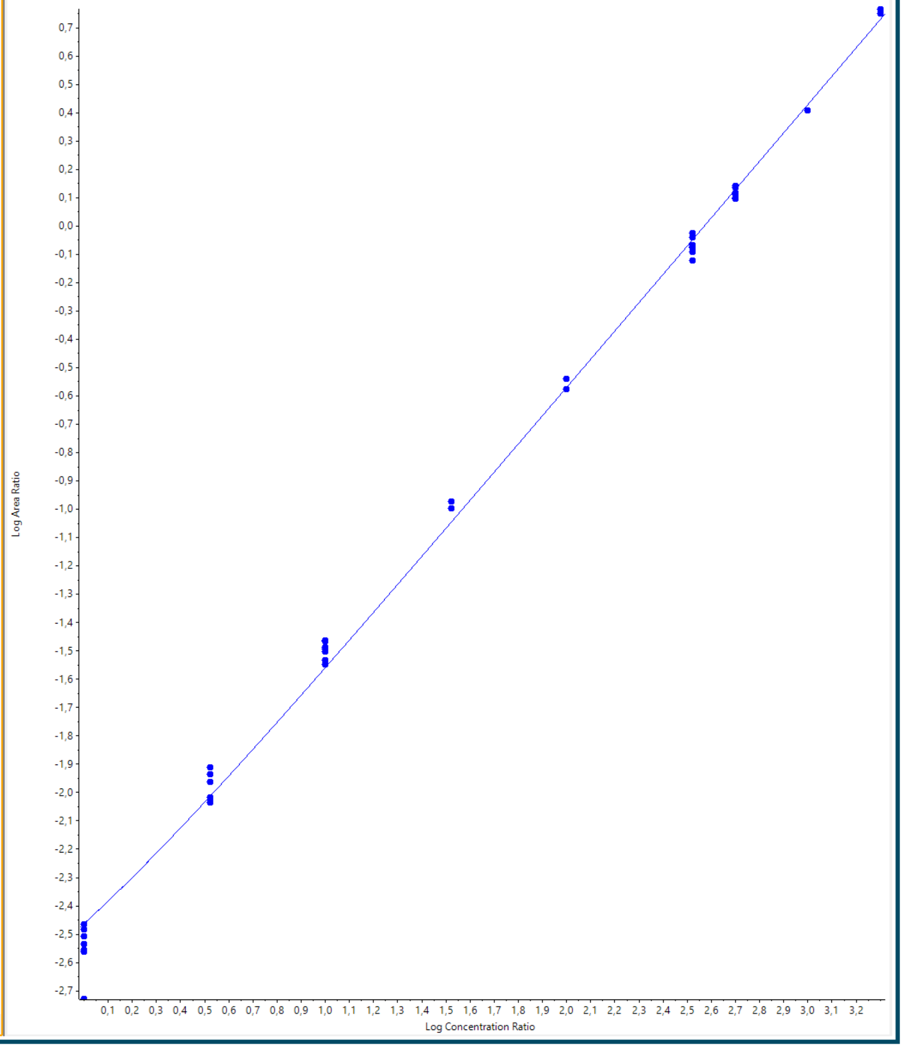

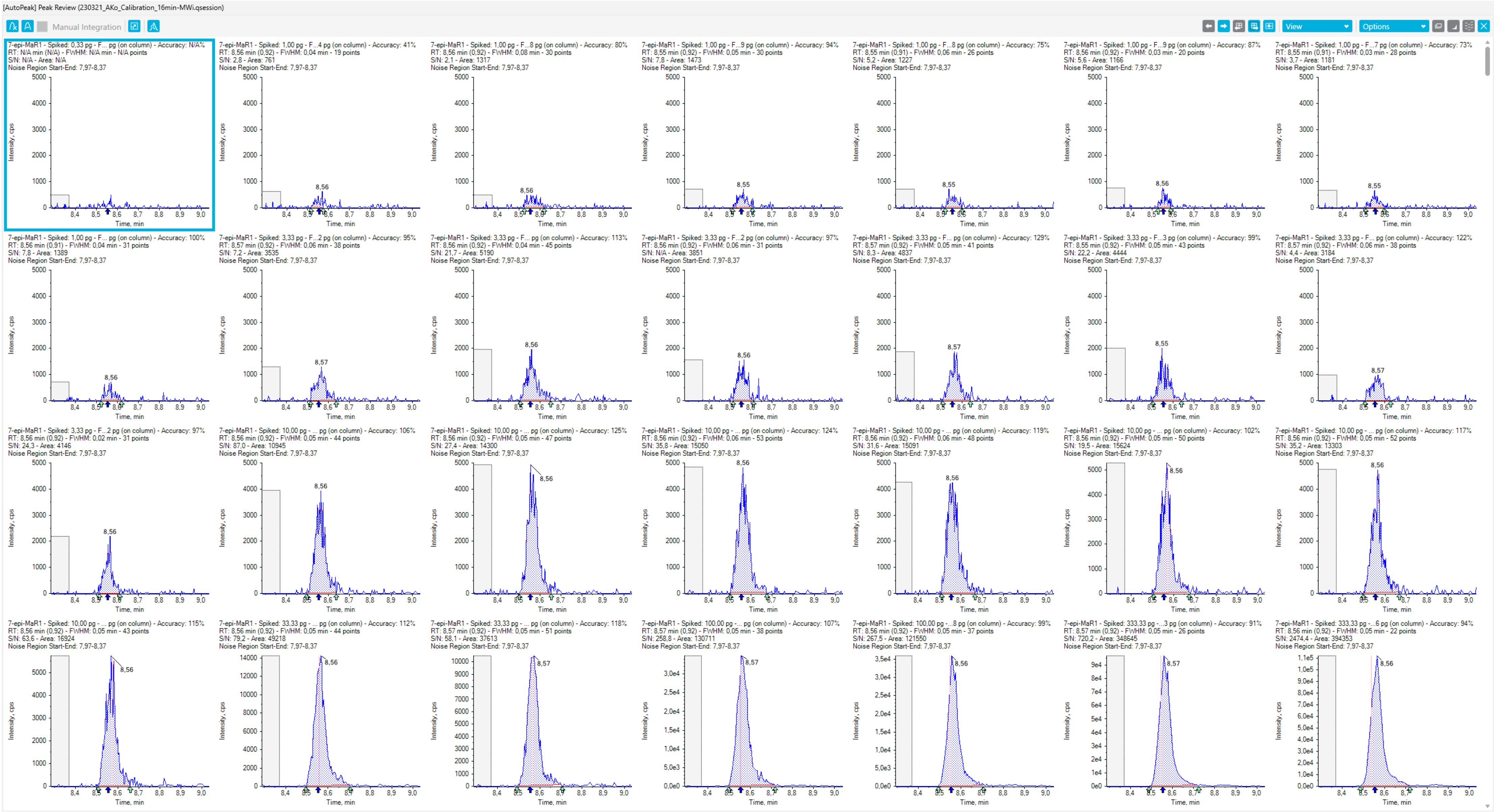

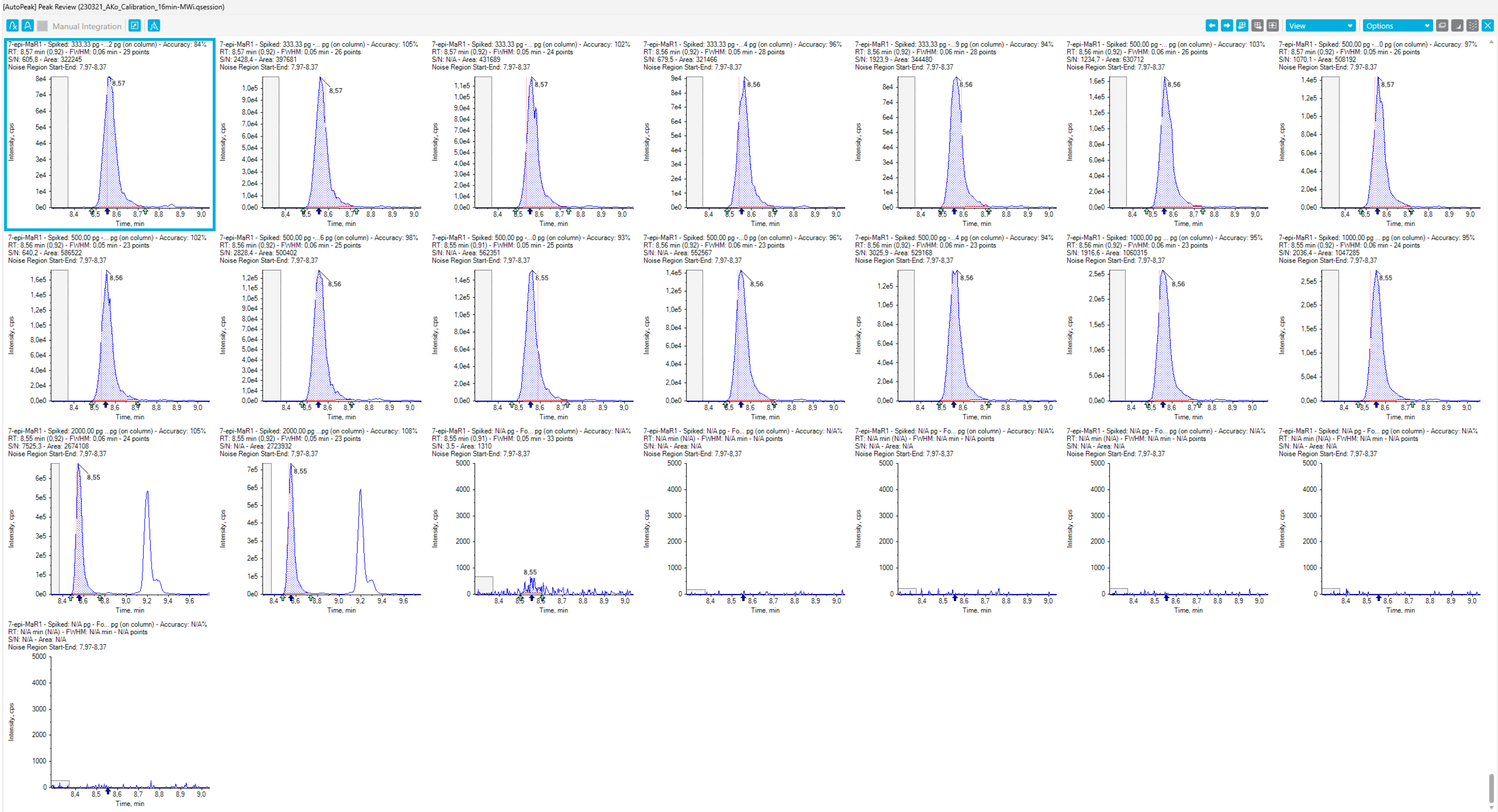
[AutoPeak] Peak Review (230321_AKo_Calibration_16min-MWi.qsession)
Manual Integration
View
Options
7-epi-MaR1 - Spiked: 333,33 pg -...2 pg (on column) - Accuracy: 84%
7-epi-MaR1 - Spiked: 333,33 pg -... pg (on column) - Accuracy: 105%
7-epi-MaR1 - Spiked: 333,33 pg -... pg (on column) - Accuracy: 102%
7-epi-MaR1 - Spiked: 333,33 pg -...4 pg (on column) - Accuracy: 96%
7-epi-MaR1 - Spiked: 333,33 pg -...9 pg (on column) - Accuracy: 94%
7-epi-MaR1 - Spiked: 500,00 pg -... pg (on column) - Accuracy: 103%
7-epi-MaR1 - Spiked: 500,00 pg -...0 pg (on column) - Accuracy: 97%
7-epi-MaR1 - Spiked: 500,00 pg -... pg (on column) - Accuracy: 102%
7-epi-MaR1 - Spiked: 500,00 pg -...6 pg (on column) - Accuracy: 98%
7-epi-MaR1 - Spiked: 500,00 pg -...0 pg (on column) - Accuracy: 93%
7-epi-MaR1 - Spiked: 500,00 pg -...0 pg (on column) - Accuracy: 96%
7-epi-MaR1 - Spiked: 500,00 pg -...4 pg (on column) - Accuracy: 94%
7-epi-MaR1 - Spiked: 1000,00 pg ... pg (on column) - Accuracy: 95%
7-epi-MaR1 - Spiked: 1000,00 pg ... pg (on column) - Accuracy: 95%
7-epi-MaR1 - Spiked: 2000,00 pg ...pg (on column) - Accuracy: 105%
7-epi-MaR1 - Spiked: 2000,00 pg ...pg (on column) - Accuracy: 108%
7-epi-MaR1 - Spiked: N/A pg - Fo... pg (on column) - Accuracy: N/A%
Noise Region Start-End: 7,97-8,37
Intensity, cps
Time, min

PDX

[AutoPeak] Results Table (230321_AKo_Calibration_16min-MWi.qsession)

50 rows Filters: 0 Qualify for Rules Filters

Sample Name	Sample T...	Component Name	Actual Concentr...	Calculated Concentrat...	Accuracy	Signal / Noise	Retent... Time	Relativ...	Width at 50%	Points Across...	Area	U...
230321_AKo_Calibration_16min-MWi_01	Standard	PDX	0,33	< 0	N/A	4,0	8,96	0,96	0,02	26	4066	☑
230321_AKo_Calibration_16min-MWi_02	Standard	PDX	1,00	0,7	73	10,8	8,95	0,96	0,06	37	13755	☑
230321_AKo_Calibration_16min-MWi_03	Standard	PDX	1,00	0,8	78	9,9	8,96	0,96	0,05	35	15871	☑
230321_AKo_Calibration_16min-MWi_04	Standard	PDX	1,00	0,9	94	17,3	8,96	0,96	0,06	44	17784	☑
230321_AKo_Calibration_16min-MWi_05	Standard	PDX	1,00	0,8	79	10,3	8,96	0,96	0,06	38	15768	☑
230321_AKo_Calibration_16min-MWi_06	Standard	PDX	1,00	0,8	82	8,1	8,96	0,96	0,05	33	13696	☑
230321_AKo_Calibration_16min-MWi_07	Standard	PDX	1,00	0,8	82	11,2	8,96	0,96	0,05	36	15697	☑
230321_AKo_Calibration_16min-MWi_08	Standard	PDX	1,00	0,9	87	8,5	8,96	0,96	0,05	33	15265	☑
230321_AKo_Calibration_16min-MWi_09	Standard	PDX	3,33	3,2	96	29,6	8,97	0,96	0,05	34	39592	☑
230321_AKo_Calibration_16min-MWi_10	Standard	PDX	3,33	3,5	106	37,0	8,96	0,96	0,04	28	53685	☑
230321_AKo_Calibration_16min-MWi_11	Standard	PDX	3,33	3,5	106	36,5	8,96	0,96	0,05	32	46231	☑
230321_AKo_Calibration_16min-MWi_12	Standard	PDX	3,33	3,7	111	24,2	8,96	0,96	0,05	33	46284	☑
230321_AKo_Calibration_16min-MWi_13	Standard	PDX	3,33	3,2	97	22,8	8,96	0,96	0,05	30	48280	☑
230321_AKo_Calibration_16min-MWi_14	Standard	PDX	3,33	3,8	114	19,2	8,96	0,96	0,06	32	33003	☑
230321_AKo_Calibration_16min-MWi_15	Standard	PDX	3,33	3,6	108	41,1	8,96	0,96	0,05	27	50482	☑
230321_AKo_Calibration_16min-MWi_16	Standard	PDX	10,00	11,8	118	75,9	8,95	0,96	0,05	27	129413	☑
230321_AKo_Calibration_16min-MWi_17	Standard	PDX	10,00	12,8	128	73,2	8,96	0,96	0,05	29	155559	☑
230321_AKo_Calibration_16min-MWi_18	Standard	PDX	10,00	11,8	118	60,2	8,96	0,96	0,06	25	152549	☑
230321_AKo_Calibration_16min-MWi_19	Standard	PDX	10,00	11,4	114	82,4	8,95	0,96	0,05	28	155519	☑
230321_AKo_Calibration_16min-MWi_20	Standard	PDX	10,00	11,1	111	81,2	8,96	0,96	0,06	24	180488	☑
230321_AKo_Calibration_16min-MWi_21	Standard	PDX	10,00	11,4	114	58,3	8,96	0,96	0,05	31	138362	☑
230321_AKo_Calibration_16min-MWi_22	Standard	PDX	10,00	11,2	112	56,1	8,96	0,96	0,05	29	177326	☑
230321_AKo_Calibration_16min-MWi_23	Standard	PDX	33,33	40,0	120	122,7	8,96	0,96	0,05	28	555595	☑
230321_AKo_Calibration_16min-MWi_24	Standard	PDX	33,33	39,5	118	71,5	8,96	0,96	0,05	31	397077	☑
230321_AKo_Calibration_16min-MWi_25	Standard	PDX	100,00	113,4	113	126,8	8,96	0,96	0,05	24	1454351	☑
230321_AKo_Calibration_16min-MWi_26	Standard	PDX	100,00	109,3	109	180,5	8,96	0,96	0,06	27	1414323	☑
230321_AKo_Calibration_16min-MWi_27	Standard	PDX	333,33	342,5	103	263,8	8,96	0,96	0,05	23	4152774	☑
230321_AKo_Calibration_16min-MWi_28	Standard	PDX	333,33	327,0	98	146,9	8,96	0,96	0,05	22	4337927	☑
230321_AKo_Calibration_16min-MWi_29	Standard	PDX	333,33	310,7	93	122,7	8,96	0,96	0,05	23	3743486	☑
230321_AKo_Calibration_16min-MWi_30	Standard	PDX	333,33	377,5	113	148,3	8,96	0,96	0,06	23	4501882	☑
230321_AKo_Calibration_16min-MWi_31	Standard	PDX	333,33	373,9	112	165,5	8,96	0,96	0,05	23	4982185	☑
230321_AKo_Calibration_16min-MWi_32	Standard	PDX	333,33	332,8	100	189,4	8,96	0,96	0,05	24	3532259	☑
230321_AKo_Calibration_16min-MWi_33	Standard	PDX	333,33	337,2	101	148,4	8,96	0,96	0,06	26	3879188	☑
230321_AKo_Calibration_16min-MWi_34	Standard	PDX	500,00	536,8	107	179,9	8,96	0,96	0,06	24	6887875	☑
230321_AKo_Calibration_16min-MWi_35	Standard	PDX	500,00	500,7	100	189,5	8,96	0,96	0,05	25	5538020	☑
230321_AKo_Calibration_16min-MWi_36	Standard	PDX	500,00	521,3	104	251,7	8,95	0,96	0,06	24	6329849	☑
230321_AKo_Calibration_16min-MWi_37	Standard	PDX	500,00	553,4	111	155,2	8,95	0,96	0,06	24	5958478	☑
230321_AKo_Calibration_16min-MWi_38	Standard	PDX	500,00	505,7	101	168,4	8,96	0,96	0,05	23	6414877	☑
230321_AKo_Calibration_16min-MWi_39	Standard	PDX	500,00	516,9	103	193,2	8,95	0,96	0,06	25	6255379	☑
230321_AKo_Calibration_16min-MWi_40	Standard	PDX	500,00	512,0	102	173,5	8,95	0,96	0,05	24	6067432	☑
230321_AKo_Calibration_16min-MWi_41	Standard	PDX	1000,00	1009,5	101	214,5	8,96	0,96	0,05	24	11841921	☑
230321_AKo_Calibration_16min-MWi_42	Standard	PDX	1000,00	992,1	99	125,2	8,95	0,96	0,06	24	11475540	☑
230321_AKo_Calibration_16min-MWi_43	Standard	PDX	2000,00	1867,3	93	120,7	8,95	0,96	0,06	25	24965658	☑
230321_AKo_Calibration_16min-MWi_44	Standard	PDX	2000,00	1869,3	93	130,7	8,95	0,96	0,06	24	24662443	☑
230321_AKo_Calibration_16min-MWi_45	Unknown	PDX	N/A	1,6	N/A	9,9	8,95	0,96	0,06	44	21527	☑
Blank	Double Blank	PDX	N/A	N/A	N/A	N/A	N/A	N/A	N/A	N/A	N/A	☑
Blank	Double Blank	PDX	N/A	N/A	N/A	N/A	N/A	N/A	N/A	N/A	N/A	☑
Blank	Double Blank	PDX	N/A	N/A	N/A	N/A	N/A	N/A	N/A	N/A	N/A	☑
Blank	Double Blank	PDX	N/A	N/A	N/A	N/A	N/A	N/A	N/A	N/A	N/A	☑
Blank	Double Blank	PDX	N/A	N/A	N/A	N/A	N/A	N/A	N/A	N/A	N/A	☑

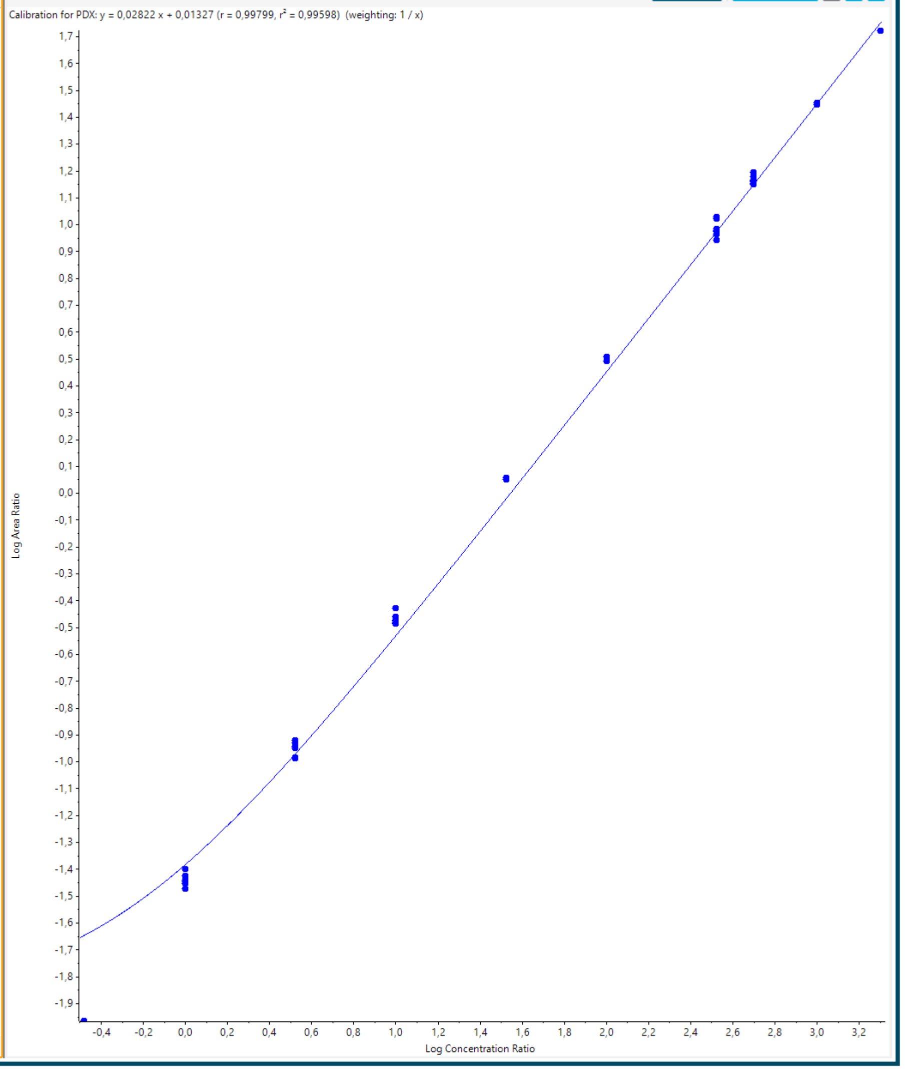

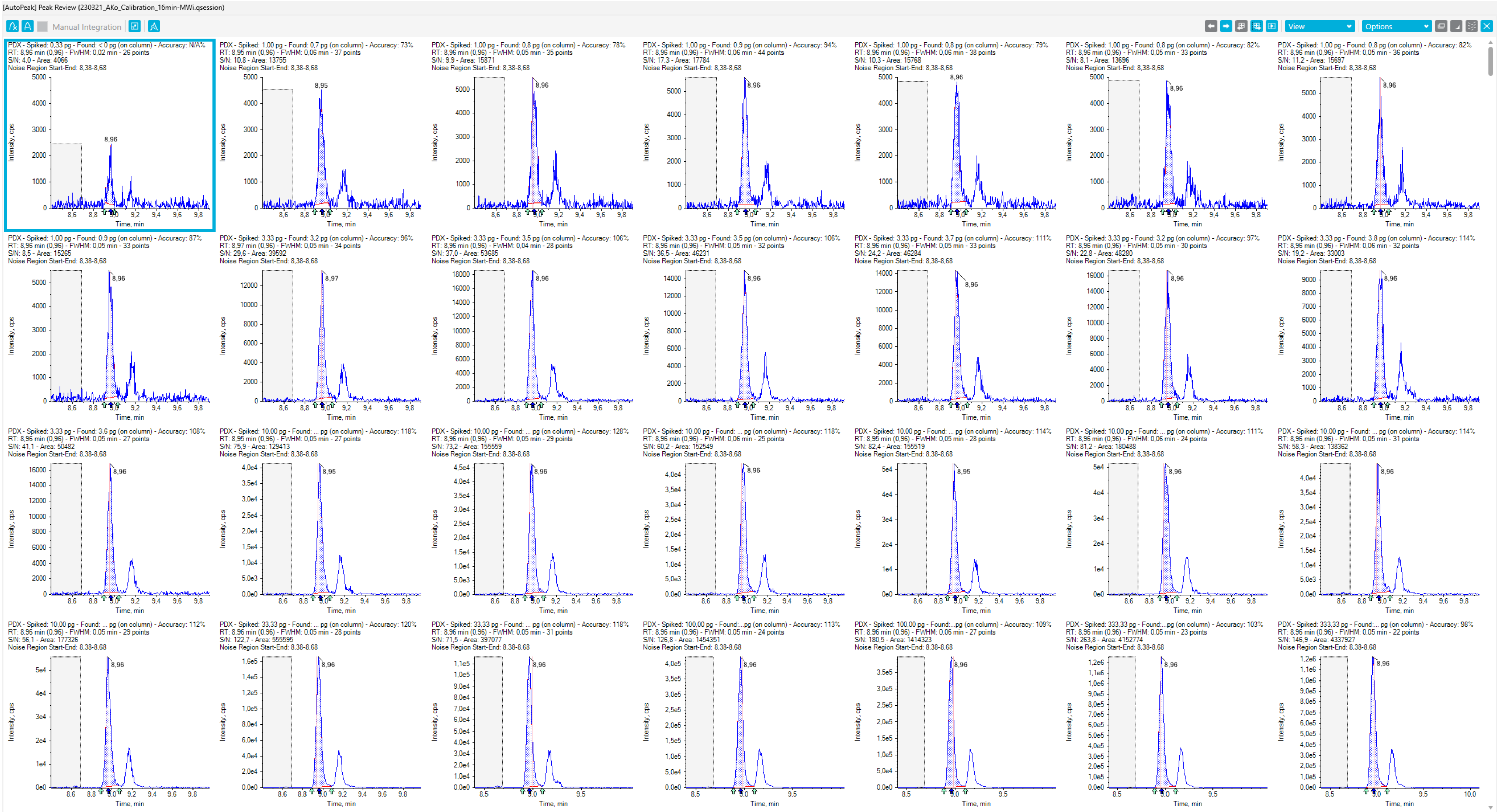
[AutoPeak] Peak Review (230321_AKo_Calibration_16min-MWi.qsession)
Manual Integration
View
Options
PDX - Spiked: 0,33 pg - Found: < 0 pg (on column) - Accuracy: N/A%
RT: 8,96 min (0,96) - FWHM: 0,02 min - 26 points
S/N: 4,0 - Area: 4066
Noise Region Start-End: 8,38-8,68
PDX - Spiked: 1,00 pg - Found: 0,7 pg (on column) - Accuracy: 73%
RT: 8,95 min (0,96) - FWHM: 0,06 min - 37 points
S/N: 10,8 - Area: 13755
Noise Region Start-End: 8,38-8,68
PDX - Spiked: 1,00 pg - Found: 0,8 pg (on column) - Accuracy: 78%
RT: 8,96 min (0,96) - FWHM: 0,05 min - 35 points
S/N: 9,9 - Area: 15871
Noise Region Start-End: 8,38-8,68
PDX - Spiked: 1,00 pg - Found: 0,9 pg (on column) - Accuracy: 94%
RT: 8,96 min (0,96) - FWHM: 0,06 min - 44 points
S/N: 17,3 - Area: 17784
Noise Region Start-End: 8,38-8,68
PDX - Spiked: 1,00 pg - Found: 0,8 pg (on column) - Accuracy: 79%
RT: 8,96 min (0,96) - FWHM: 0,06 min - 38 points
S/N: 10,3 - Area: 15768
Noise Region Start-End: 8,38-8,68
PDX - Spiked: 1,00 pg - Found: 0,8 pg (on column) - Accuracy: 82%
RT: 8,96 min (0,96) - FWHM: 0,05 min - 33 points
S/N: 8,1 - Area: 13696
Noise Region Start-End: 8,38-8,68
PDX - Spiked: 1,00 pg - Found: 0,8 pg (on column) - Accuracy: 82%
RT: 8,96 min (0,96) - FWHM: 0,05 min - 36 points
S/N: 11,2 - Area: 15697
Noise Region Start-End: 8,38-8,68
PDX - Spiked: 1,00 pg - Found: 0,9 pg (on column) - Accuracy: 87%
RT: 8,96 min (0,96) - FWHM: 0,05 min - 33 points
S/N: 8,5 - Area: 15265
Noise Region Start-End: 8,38-8,68
PDX - Spiked: 3,33 pg - Found: 3,2 pg (on column) - Accuracy: 96%
RT: 8,97 min (0,96) - FWHM: 0,05 min - 34 points
S/N: 29,6 - Area: 39592
Noise Region Start-End: 8,38-8,68
PDX - Spiked: 3,33 pg - Found: 3,5 pg (on column) - Accuracy: 106%
RT: 8,96 min (0,96) - FWHM: 0,04 min - 28 points
S/N: 37,0 - Area: 53685
Noise Region Start-End: 8,38-8,68
PDX - Spiked: 3,33 pg - Found: 3,5 pg (on column) - Accuracy: 106%
RT: 8,96 min (0,96) - FWHM: 0,05 min - 32 points
S/N: 36,5 - Area: 46231
Noise Region Start-End: 8,38-8,68
PDX - Spiked: 3,33 pg - Found: 3,7 pg (on column) - Accuracy: 111%
RT: 8,96 min (0,96) - FWHM: 0,05 min - 33 points
S/N: 24,2 - Area: 46284
Noise Region Start-End: 8,38-8,68
PDX - Spiked: 3,33 pg - Found: 3,2 pg (on column) - Accuracy: 97%
RT: 8,96 min (0,96) - FWHM: 0,05 min - 30 points
S/N: 22,8 - Area: 48280
Noise Region Start-End: 8,38-8,68
PDX - Spiked: 3,33 pg - Found: 3,8 pg (on column) - Accuracy: 114%
RT: 8,96 min (0,96) - FWHM: 0,06 min - 32 points
S/N: 19,2 - Area: 33003
Noise Region Start-End: 8,38-8,68
PDX - Spiked: 3,33 pg - Found: 3,6 pg (on column) - Accuracy: 108%
RT: 8,96 min (0,96) - FWHM: 0,05 min - 27 points
S/N: 41,1 - Area: 50482
Noise Region Start-End: 8,38-8,68
PDX - Spiked: 10,00 pg - Found: ... pg (on column) - Accuracy: 118%
RT: 8,95 min (0,96) - FWHM: 0,05 min - 27 points
S/N: 75,9 - Area: 129413
Noise Region Start-End: 8,38-8,68
PDX - Spiked: 10,00 pg - Found: ... pg (on column) - Accuracy: 128%
RT: 8,96 min (0,96) - FWHM: 0,05 min - 29 points
S/N: 73,2 - Area: 155559
Noise Region Start-End: 8,38-8,68
PDX - Spiked: 10,00 pg - Found: ... pg (on column) - Accuracy: 118%
RT: 8,96 min (0,96) - FWHM: 0,06 min - 25 points
S/N: 60,2 - Area: 152549
Noise Region Start-End: 8,38-8,68
PDX - Spiked: 10,00 pg - Found: ... pg (on column) - Accuracy: 114%
RT: 8,95 min (0,96) - FWHM: 0,05 min - 28 points
S/N: 82,4 - Area: 155519
Noise Region Start-End: 8,38-8,68
PDX - Spiked: 10,00 pg - Found: ... pg (on column) - Accuracy: 111%
RT: 8,96 min (0,96) - FWHM: 0,06 min - 24 points
S/N: 81,2 - Area: 180488
Noise Region Start-End: 8,38-8,68
PDX - Spiked: 10,00 pg - Found: ... pg (on column) - Accuracy: 114%
RT: 8,96 min (0,96) - FWHM: 0,05 min - 31 points
S/N: 58,3 - Area: 138362
Noise Region Start-End: 8,38-8,68
PDX - Spiked: 10,00 pg - Found: ... pg (on column) - Accuracy: 112%
RT: 8,96 min (0,96) - FWHM: 0,05 min - 29 points
S/N: 56,1 - Area: 177326
Noise Region Start-End: 8,38-8,68
PDX - Spiked: 33,33 pg - Found: ... pg (on column) - Accuracy: 120%
RT: 8,96 min (0,96) - FWHM: 0,05 min - 28 points
S/N: 122,7 - Area: 555595
Noise Region Start-End: 8,38-8,68
PDX - Spiked: 33,33 pg - Found: ... pg (on column) - Accuracy: 118%
RT: 8,96 min (0,96) - FWHM: 0,05 min - 31 points
S/N: 71,5 - Area: 397077
Noise Region Start-End: 8,38-8,68
PDX - Spiked: 100,00 pg - Found: ... pg (on column) - Accuracy: 113%
RT: 8,96 min (0,96) - FWHM: 0,05 min - 24 points
S/N: 126,8 - Area: 1454351
Noise Region Start-End: 8,38-8,68
PDX - Spiked: 100,00 pg - Found:... pg (on column) - Accuracy: 109%
RT: 8,96 min (0,96) - FWHM: 0,06 min - 27 points
S/N: 180,5 - Area: 1414323
Noise Region Start-End: 8,38-8,68
PDX - Spiked: 333,33 pg - Found:...pg (on column) - Accuracy: 103%
RT: 8,96 min (0,96) - FWHM: 0,05 min - 23 points
S/N: 263,8 - Area: 4152774
Noise Region Start-End: 8,38-8,68
PDX - Spiked: 333,33 pg - Found:... pg (on column) - Accuracy: 98%
RT: 8,96 min (0,96) - FWHM: 0,05 min - 22 points
S/N: 146,9 - Area: 4337927
Noise Region Start-End: 8,38-8,68
Intensity, cps
Time, min

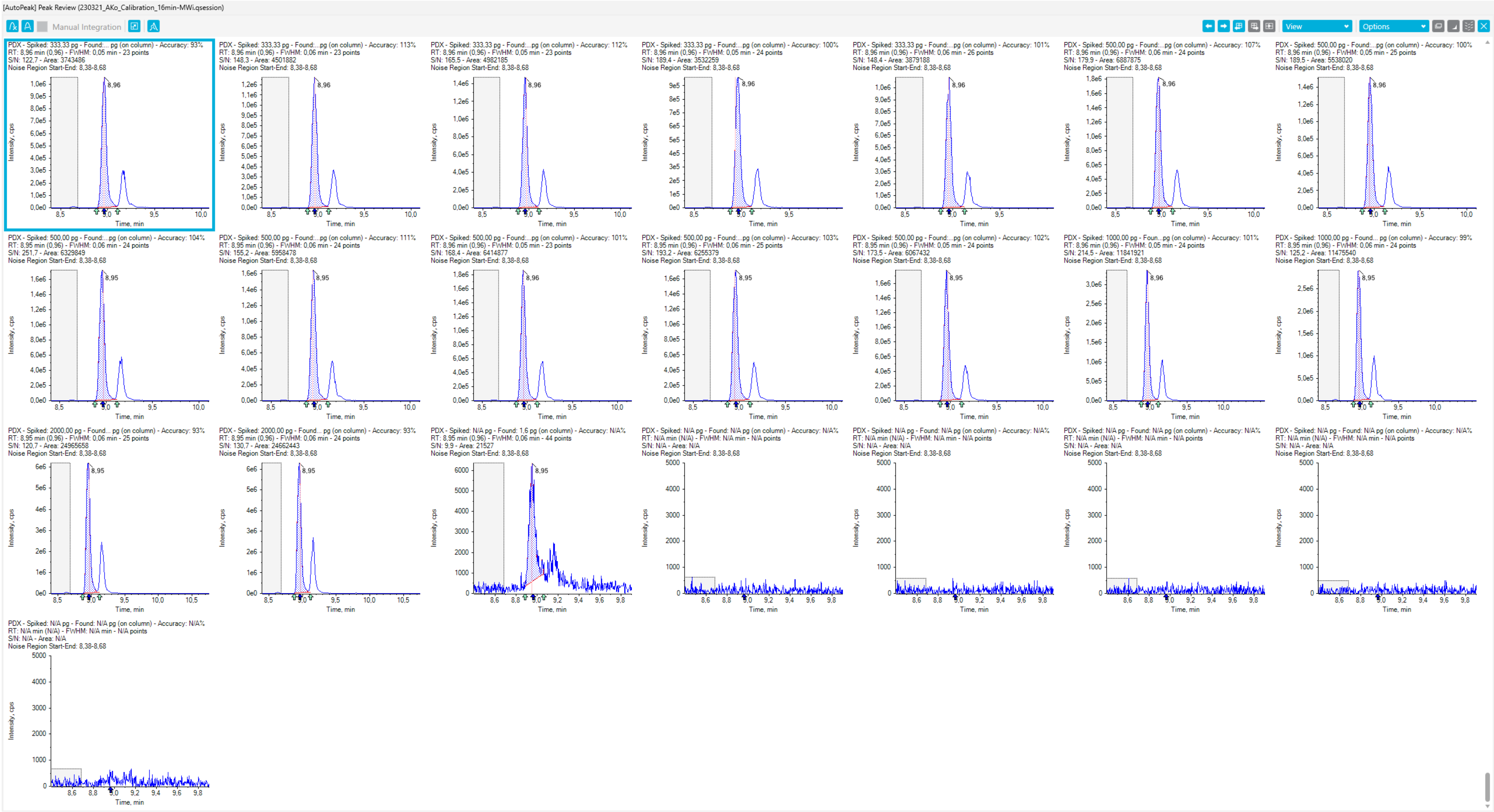
[AutoPeak] Peak Review (230321_AKo_Calibration_16min-MWi.qsession)
Manual Integration
View
Options

PD1

[AutoPeak] Results Table (230321_AKo_Calibration_16min-MWi.qsession)

50 rows Filters: 0

Sample Name	Sample T...	Component Name	Actual Concentr...	Calculated Concentrat...	Accuracy	Signal / Noise	Retent... Time	Relativ...	Width at 50%	Points Across...	Area	U...
230321_AKo_Calibration_16min-MWi_01	Standard	PD1	0,33	0,2	64	2,4	9,15	0,98	0,02	14	1152	☑
230321_AKo_Calibration_16min-MWi_02	Standard	PD1	1,00	0,8	82	3,1	9,17	0,98	0,06	29	3611	☑
230321_AKo_Calibration_16min-MWi_03	Standard	PD1	1,00	0,8	83	4,1	9,16	0,98	0,03	23	4027	☑
230321_AKo_Calibration_16min-MWi_04	Standard	PD1	1,00	1,1	113	6,7	9,16	0,98	0,06	29	5267	☑
230321_AKo_Calibration_16min-MWi_05	Standard	PD1	1,00	0,9	94	5,8	9,16	0,98	0,04	36	4418	☑
230321_AKo_Calibration_16min-MWi_06	Standard	PD1	1,00	1,0	99	3,6	9,16	0,98	0,05	25	3939	☑
230321_AKo_Calibration_16min-MWi_07	Standard	PD1	1,00	1,0	103	6,1	9,17	0,98	0,02	37	4672	☑
230321_AKo_Calibration_16min-MWi_08	Standard	PD1	1,00	1,1	106	3,0	9,17	0,98	0,04	31	4496	☑
230321_AKo_Calibration_16min-MWi_09	Standard	PD1	3,33	3,2	97	10,0	9,17	0,98	0,06	39	12122	☑
230321_AKo_Calibration_16min-MWi_10	Standard	PD1	3,33	3,5	106	10,6	9,16	0,98	0,05	38	16471	☑
230321_AKo_Calibration_16min-MWi_11	Standard	PD1	3,33	3,2	96	12,8	9,16	0,98	0,04	25	12851	☑
230321_AKo_Calibration_16min-MWi_12	Standard	PD1	3,33	3,4	103	12,4	9,16	0,98	0,06	32	13252	☑
230321_AKo_Calibration_16min-MWi_13	Standard	PD1	3,33	3,4	101	15,9	9,16	0,98	0,05	36	15254	☑
230321_AKo_Calibration_16min-MWi_14	Standard	PD1	3,33	3,7	110	8,1	9,16	0,98	0,03	39	9784	☑
230321_AKo_Calibration_16min-MWi_15	Standard	PD1	3,33	3,4	103	10,7	9,16	0,98	0,05	37	14764	☑
230321_AKo_Calibration_16min-MWi_16	Standard	PD1	10,00	12,0	120	22,0	9,17	0,98	0,06	39	42721	☑
230321_AKo_Calibration_16min-MWi_17	Standard	PD1	10,00	11,9	119	25,6	9,17	0,98	0,05	44	47264	☑
230321_AKo_Calibration_16min-MWi_18	Standard	PD1	10,00	10,5	105	23,5	9,17	0,98	0,05	42	44062	☑
230321_AKo_Calibration_16min-MWi_19	Standard	PD1	10,00	10,0	100	21,2	9,16	0,98	0,06	29	44178	☑
230321_AKo_Calibration_16min-MWi_20	Standard	PD1	10,00	9,6	96	34,4	9,17	0,98	0,06	29	50803	☑
230321_AKo_Calibration_16min-MWi_21	Standard	PD1	10,00	10,2	102	30,1	9,17	0,98	0,06	34	40410	☑
230321_AKo_Calibration_16min-MWi_22	Standard	PD1	10,00	10,7	107	36,1	9,17	0,98	0,05	41	54789	☑
230321_AKo_Calibration_16min-MWi_23	Standard	PD1	33,33	35,8	107	83,8	9,17	0,98	0,05	34	165340	☑
230321_AKo_Calibration_16min-MWi_24	Standard	PD1	33,33	34,6	104	79,5	9,17	0,98	0,06	36	115618	☑
230321_AKo_Calibration_16min-MWi_25	Standard	PD1	100,00	110,1	110	103,5	9,17	0,98	0,06	44	471737	☑
230321_AKo_Calibration_16min-MWi_26	Standard	PD1	100,00	100,9	101	158,0	9,16	0,98	0,06	42	436386	☑
230321_AKo_Calibration_16min-MWi_27	Standard	PD1	333,33	341,5	102	270,2	9,17	0,98	0,05	35	1386389	☑
230321_AKo_Calibration_16min-MWi_28	Standard	PD1	333,33	303,5	91	161,9	9,17	0,98	0,06	30	1348029	☑
230321_AKo_Calibration_16min-MWi_29	Standard	PD1	333,33	284,6	85	169,7	9,17	0,98	0,05	34	1148211	☑
230321_AKo_Calibration_16min-MWi_30	Standard	PD1	333,33	353,3	106	187,0	9,17	0,98	0,06	33	1411129	☑
230321_AKo_Calibration_16min-MWi_31	Standard	PD1	333,33	348,7	105	305,6	9,17	0,98	0,05	31	1555668	☑
230321_AKo_Calibration_16min-MWi_32	Standard	PD1	333,33	312,2	94	288,3	9,16	0,98	0,06	35	1109586	☑
230321_AKo_Calibration_16min-MWi_33	Standard	PD1	333,33	311,4	93	315,6	9,17	0,98	0,06	33	1199395	☑
230321_AKo_Calibration_16min-MWi_34	Standard	PD1	500,00	486,9	97	265,5	9,16	0,98	0,06	31	2092875	☑
230321_AKo_Calibration_16min-MWi_35	Standard	PD1	500,00	487,5	98	300,2	9,16	0,98	0,06	33	1806112	☑
230321_AKo_Calibration_16min-MWi_36	Standard	PD1	500,00	504,7	101	269,6	9,16	0,98	0,06	31	2052647	☑
230321_AKo_Calibration_16min-MWi_37	Standard	PD1	500,00	500,0	100	304,4	9,16	0,98	0,05	32	1803351	☑
230321_AKo_Calibration_16min-MWi_38	Standard	PD1	500,00	513,5	103	459,8	9,16	0,98	0,06	32	2182076	☑
230321_AKo_Calibration_16min-MWi_39	Standard	PD1	500,00	477,1	95	327,0	9,16	0,98	0,06	31	1933895	☑
230321_AKo_Calibration_16min-MWi_40	Standard	PD1	500,00	467,7	94	347,0	9,16	0,98	0,06	30	1856591	☑
230321_AKo_Calibration_16min-MWi_41	Standard	PD1	1000,00	994,2	99	377,6	9,16	0,98	0,06	30	3907502	☑
230321_AKo_Calibration_16min-MWi_42	Standard	PD1	1000,00	941,8	94	382,3	9,16	0,98	0,06	31	3650175	☑
230321_AKo_Calibration_16min-MWi_43	Standard	PD1	2000,00	2079,2	104	322,2	9,16	0,98	0,06	30	9315878	☑
230321_AKo_Calibration_16min-MWi_44	Standard	PD1	2000,00	2105,6	105	445,1	9,16	0,98	0,06	31	9309584	☑
230321_AKo_Calibration_16min-MWi_45	Unknown	PD1	N/A	1,3	N/A	3,0	9,16	0,98	0,09	27	5034	☑
Blank	Double Blank	PD1	N/A	N/A	N/A	N/A	N/A	N/A	N/A	N/A	N/A	☑
Blank	Double Blank	PD1	N/A	N/A	N/A	N/A	N/A	N/A	N/A	N/A	N/A	☑
Blank	Double Blank	PD1	N/A	N/A	N/A	N/A	N/A	N/A	N/A	N/A	N/A	☑
Blank	Double Blank	PD1	N/A	N/A	N/A	N/A	N/A	N/A	N/A	N/A	N/A	☑
Blank	Double Blank	PD1	N/A	N/A	N/A	N/A	N/A	N/A	N/A	N/A	N/A	☑

Calibration for PD1: y = 0,00946 x + 0,00108 (r = 0,99862, r^2 = 0,99724) (weighting: 1 / x)

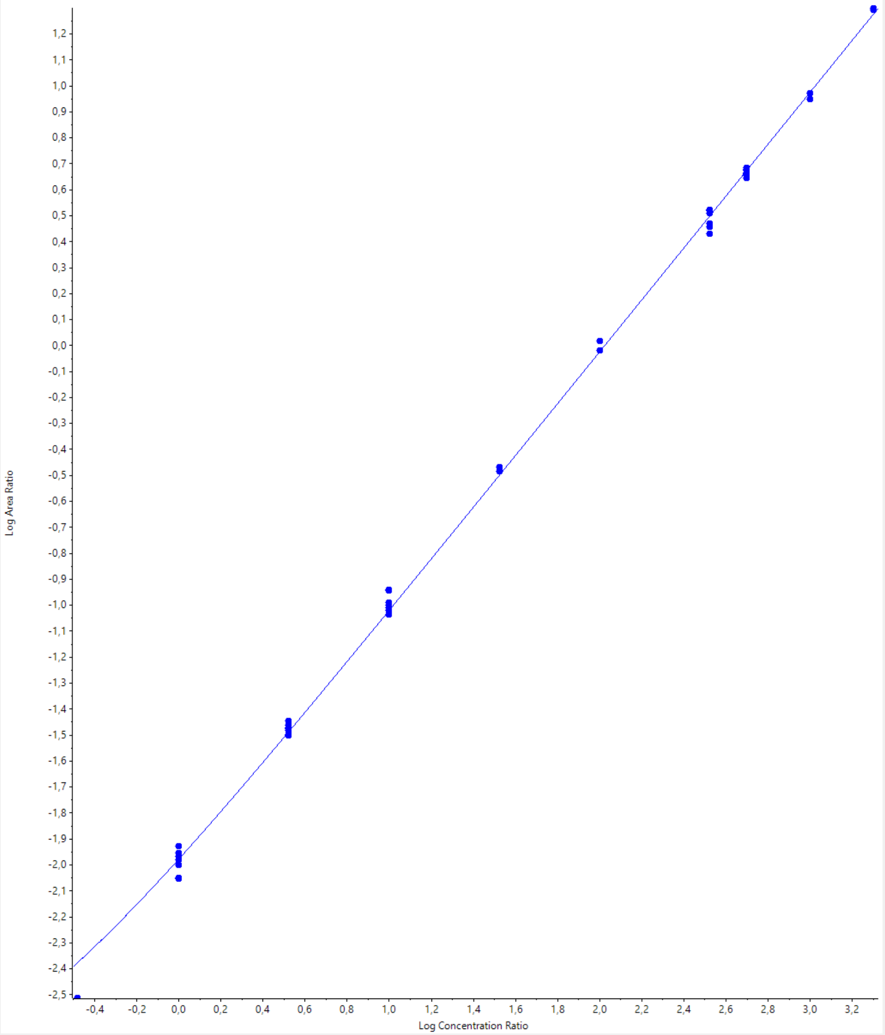

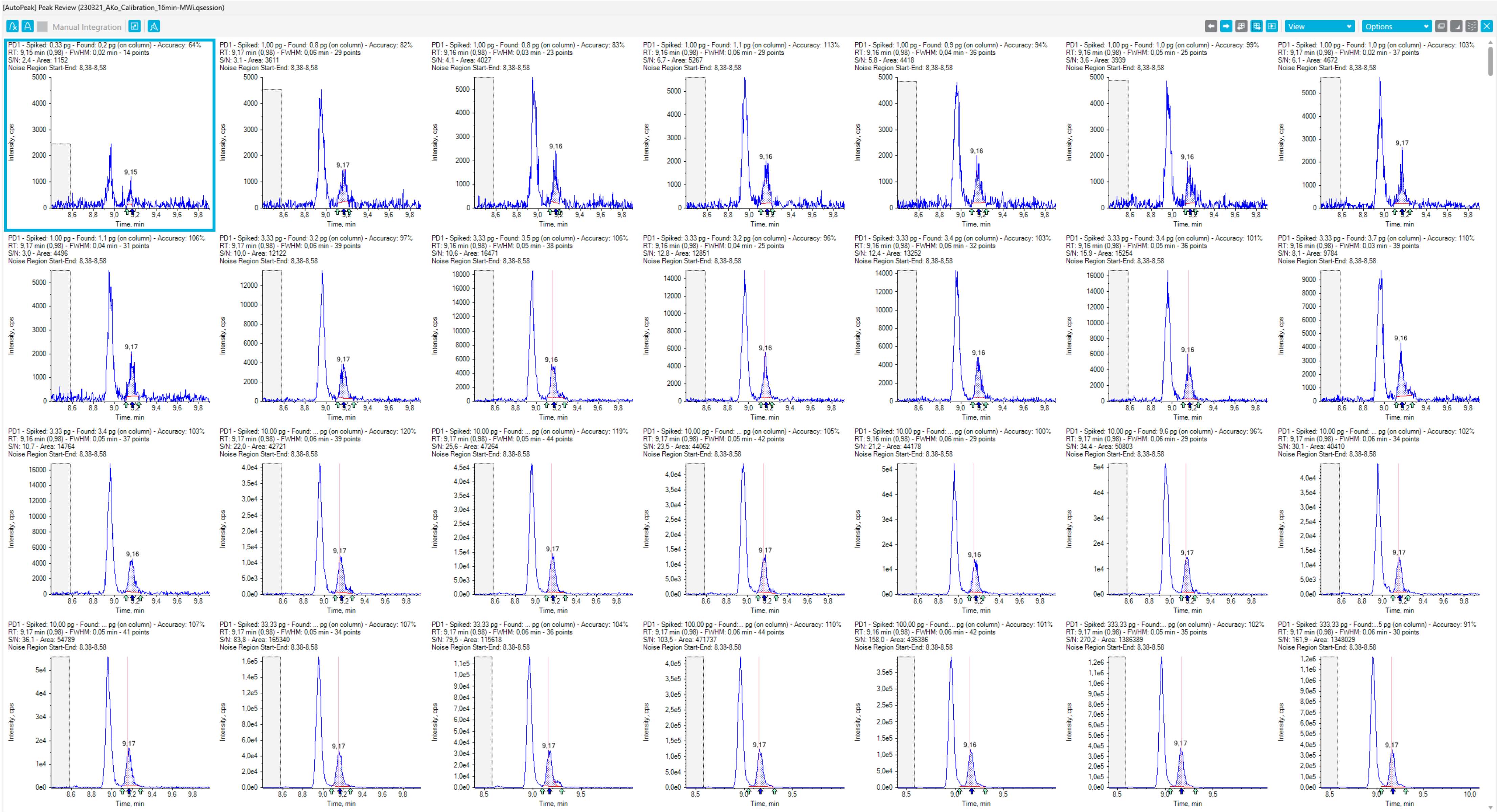
[AutoPeak] Peak Review (230321_AKo_Calibration_16min-MWi.qsession)
Manual Integration
View
Options

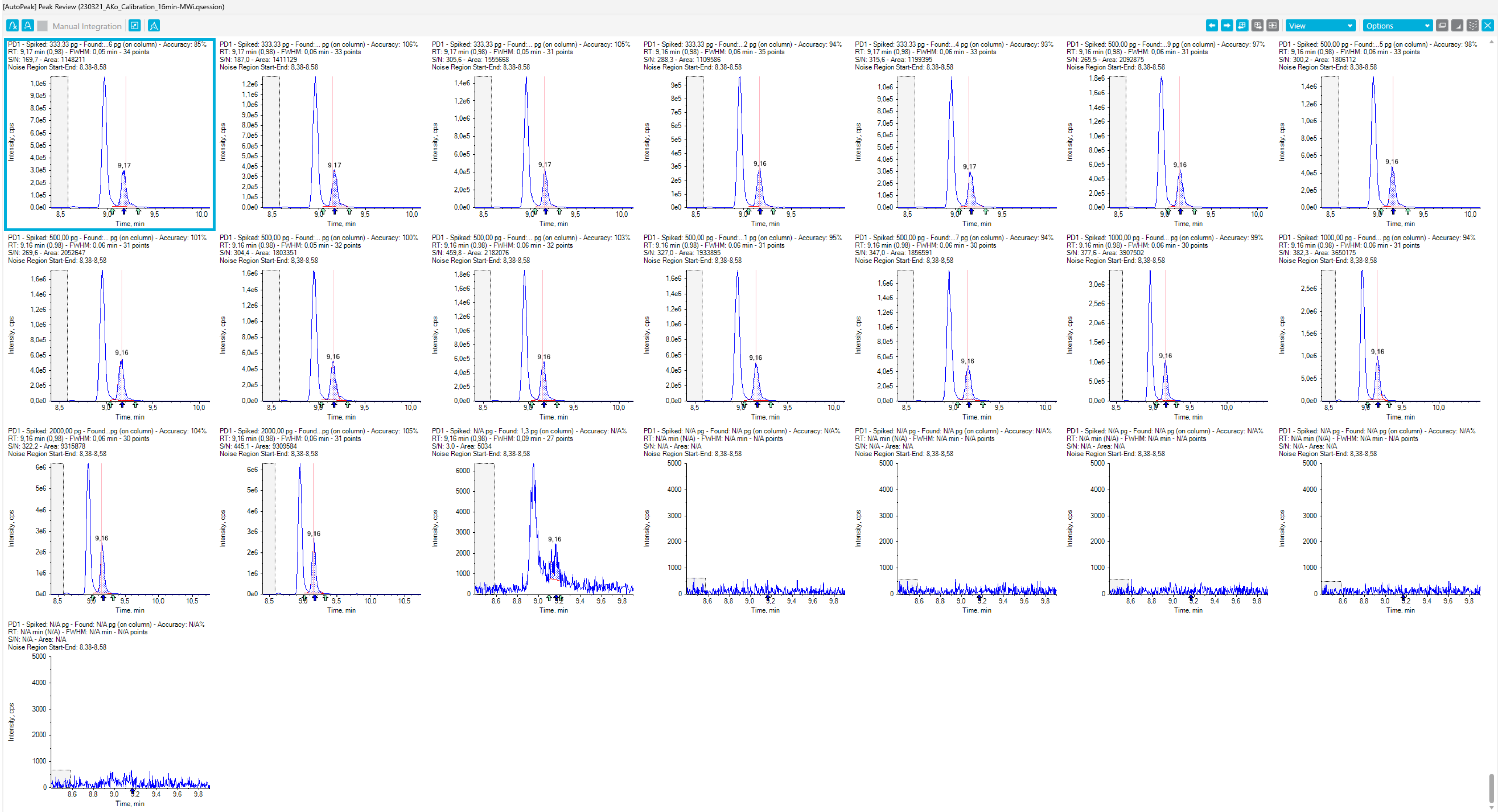
[AutoPeak] Peak Review (230321_AKo_Calibration_16min-MWi.qsession)
Manual Integration
View
Options
PD1 - Spiked: 333,33 pg - Found:...6 pg (on column) - Accuracy: 85%
RT: 9,17 min (0,98) - FWHM: 0,05 min - 34 points
S/N: 169,7 - Area: 1148211
Noise Region Start-End: 8,38-8,58
PD1 - Spiked: 333,33 pg - Found:... pg (on column) - Accuracy: 106%
PD1 - Spiked: 333,33 pg - Found:... pg (on column) - Accuracy: 105%
PD1 - Spiked: 333,33 pg - Found:...2 pg (on column) - Accuracy: 94%
PD1 - Spiked: 333,33 pg - Found:...4 pg (on column) - Accuracy: 93%
PD1 - Spiked: 500,00 pg - Found:...9 pg (on column) - Accuracy: 97%
PD1 - Spiked: 500,00 pg - Found:...5 pg (on column) - Accuracy: 98%
PD1 - Spiked: 500,00 pg - Found:... pg (on column) - Accuracy: 101%
PD1 - Spiked: 500,00 pg - Found:... pg (on column) - Accuracy: 100%
PD1 - Spiked: 500,00 pg - Found:... pg (on column) - Accuracy: 103%
PD1 - Spiked: 500,00 pg - Found:...1 pg (on column) - Accuracy: 95%
PD1 - Spiked: 500,00 pg - Found:...7 pg (on column) - Accuracy: 94%
PD1 - Spiked: 1000,00 pg - Found... pg (on column) - Accuracy: 99%
PD1 - Spiked: 1000,00 pg - Found... pg (on column) - Accuracy: 94%
PD1 - Spiked: 2000,00 pg - Found...pg (on column) - Accuracy: 104%
PD1 - Spiked: 2000,00 pg - Found...pg (on column) - Accuracy: 105%
PD1 - Spiked: N/A pg - Found: 1,3 pg (on column) - Accuracy: N/A%
PD1 - Spiked: N/A pg - Found: N/A pg (on column) - Accuracy: N/A%
Intensity, cps
Time, min

RvD5

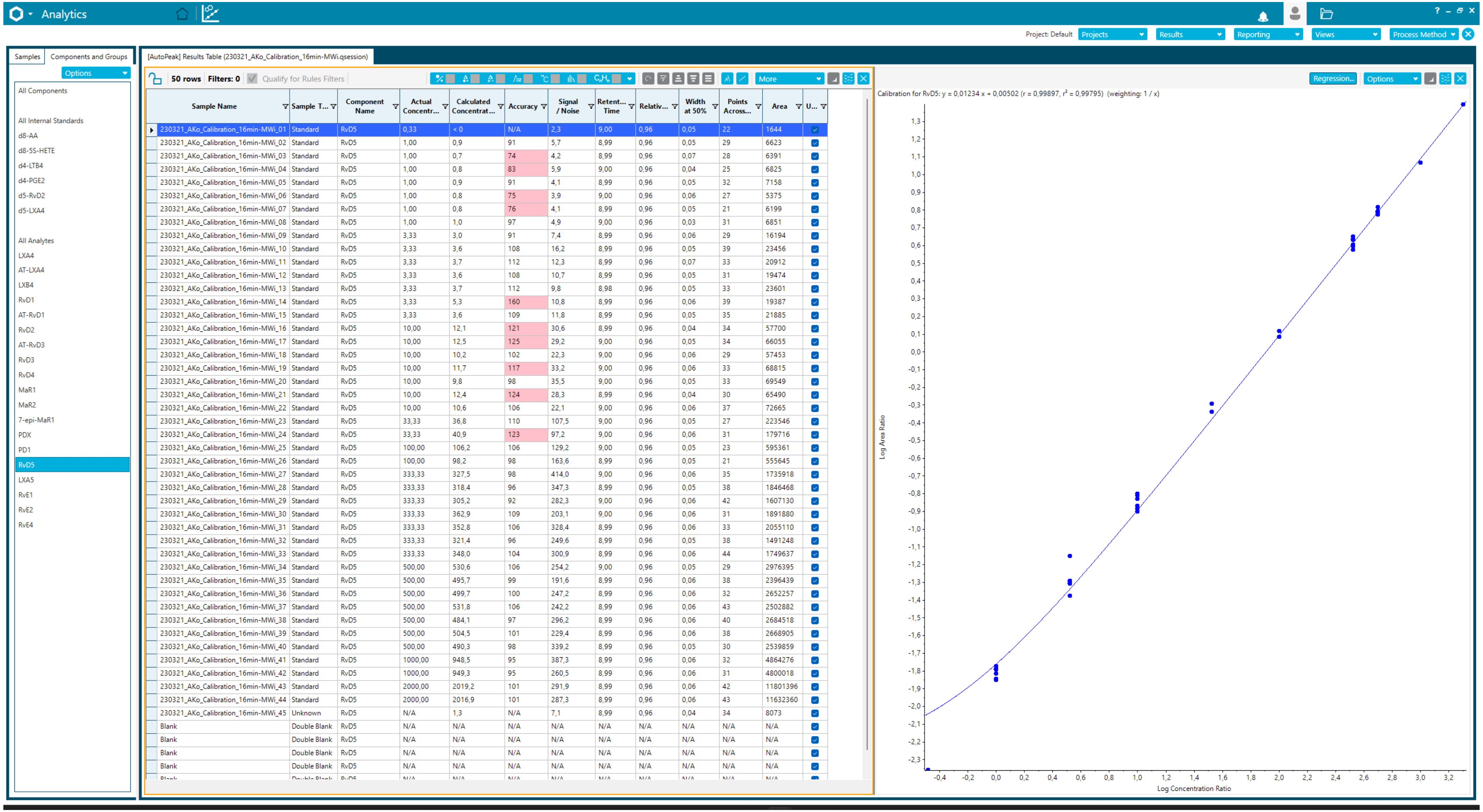

Sample Name	Sample T...	Component Name	Actual Concentr...	Calculated Concentrat...	Accuracy	Signal / Noise	Retent... Time	Relativ...	Width at 50%	Points Across...	Area	U...
230321_AKo_Calibration_16min-MWi_01	Standard	RvD5	0,33	< 0	N/A	2,3	9,00	0,96	0,05	22	1644	☑
230321_AKo_Calibration_16min-MWi_02	Standard	RvD5	1,00	0,9	91	5,7	8,99	0,96	0,05	29	6623	☑
230321_AKo_Calibration_16min-MWi_03	Standard	RvD5	1,00	0,7	74	4,2	8,99	0,96	0,07	28	6391	☑
230321_AKo_Calibration_16min-MWi_04	Standard	RvD5	1,00	0,8	83	5,9	9,00	0,96	0,04	25	6825	☑
230321_AKo_Calibration_16min-MWi_05	Standard	RvD5	1,00	0,9	91	4,1	8,99	0,96	0,05	32	7158	☑
230321_AKo_Calibration_16min-MWi_06	Standard	RvD5	1,00	0,8	75	3,9	9,00	0,96	0,06	27	5375	☑
230321_AKo_Calibration_16min-MWi_07	Standard	RvD5	1,00	0,8	76	4,1	8,99	0,96	0,05	21	6199	☑
230321_AKo_Calibration_16min-MWi_08	Standard	RvD5	1,00	1,0	97	4,9	9,00	0,96	0,03	31	6851	☑
230321_AKo_Calibration_16min-MWi_09	Standard	RvD5	3,33	3,0	91	7,4	8,99	0,96	0,06	29	16194	☑
230321_AKo_Calibration_16min-MWi_10	Standard	RvD5	3,33	3,6	108	16,2	8,99	0,96	0,05	39	23456	☑
230321_AKo_Calibration_16min-MWi_11	Standard	RvD5	3,33	3,7	112	12,3	8,99	0,96	0,07	33	20912	☑
230321_AKo_Calibration_16min-MWi_12	Standard	RvD5	3,33	3,6	108	10,7	8,99	0,96	0,05	31	19474	☑
230321_AKo_Calibration_16min-MWi_13	Standard	RvD5	3,33	3,7	112	9,8	8,98	0,96	0,05	33	23601	☑
230321_AKo_Calibration_16min-MWi_14	Standard	RvD5	3,33	5,3	160	10,8	8,99	0,96	0,06	39	19387	☑
230321_AKo_Calibration_16min-MWi_15	Standard	RvD5	3,33	3,6	109	11,8	8,99	0,96	0,05	35	21885	☑
230321_AKo_Calibration_16min-MWi_16	Standard	RvD5	10,00	12,1	121	30,6	8,99	0,96	0,04	34	57700	☑
230321_AKo_Calibration_16min-MWi_17	Standard	RvD5	10,00	12,5	125	29,2	9,00	0,96	0,05	34	66055	☑
230321_AKo_Calibration_16min-MWi_18	Standard	RvD5	10,00	10,2	102	22,3	9,00	0,96	0,06	29	57453	☑
230321_AKo_Calibration_16min-MWi_19	Standard	RvD5	10,00	11,7	117	33,2	9,00	0,96	0,06	33	68815	☑
230321_AKo_Calibration_16min-MWi_20	Standard	RvD5	10,00	9,8	98	35,5	9,00	0,96	0,05	33	69549	☑
230321_AKo_Calibration_16min-MWi_21	Standard	RvD5	10,00	12,4	124	28,3	8,99	0,96	0,04	30	65490	☑
230321_AKo_Calibration_16min-MWi_22	Standard	RvD5	10,00	10,6	106	22,1	9,00	0,96	0,06	37	72665	☑
230321_AKo_Calibration_16min-MWi_23	Standard	RvD5	33,33	36,8	110	107,5	9,00	0,96	0,05	27	223546	☑
230321_AKo_Calibration_16min-MWi_24	Standard	RvD5	33,33	40,9	123	97,2	9,00	0,96	0,06	31	179716	☑
230321_AKo_Calibration_16min-MWi_25	Standard	RvD5	100,00	106,2	106	129,2	9,00	0,96	0,05	23	595361	☑
230321_AKo_Calibration_16min-MWi_26	Standard	RvD5	100,00	98,2	98	163,6	8,99	0,96	0,05	21	555645	☑
230321_AKo_Calibration_16min-MWi_27	Standard	RvD5	333,33	327,5	98	414,0	9,00	0,96	0,06	35	1735918	☑
230321_AKo_Calibration_16min-MWi_28	Standard	RvD5	333,33	318,4	96	347,3	8,99	0,96	0,05	38	1846468	☑
230321_AKo_Calibration_16min-MWi_29	Standard	RvD5	333,33	305,2	92	282,3	9,00	0,96	0,06	42	1607130	☑
230321_AKo_Calibration_16min-MWi_30	Standard	RvD5	333,33	362,9	109	203,1	9,00	0,96	0,06	31	1891880	☑
230321_AKo_Calibration_16min-MWi_31	Standard	RvD5	333,33	352,8	106	328,4	8,99	0,96	0,06	33	2055110	☑
230321_AKo_Calibration_16min-MWi_32	Standard	RvD5	333,33	321,4	96	249,6	8,99	0,96	0,05	38	1491248	☑
230321_AKo_Calibration_16min-MWi_33	Standard	RvD5	333,33	348,0	104	300,9	8,99	0,96	0,06	44	1749637	☑
230321_AKo_Calibration_16min-MWi_34	Standard	RvD5	500,00	530,6	106	254,2	9,00	0,96	0,05	29	2976395	☑
230321_AKo_Calibration_16min-MWi_35	Standard	RvD5	500,00	495,7	99	191,6	8,99	0,96	0,06	38	2396439	☑
230321_AKo_Calibration_16min-MWi_36	Standard	RvD5	500,00	499,7	100	247,2	8,99	0,96	0,06	32	2652257	☑
230321_AKo_Calibration_16min-MWi_37	Standard	RvD5	500,00	531,8	106	242,2	8,99	0,96	0,06	43	2502882	☑
230321_AKo_Calibration_16min-MWi_38	Standard	RvD5	500,00	484,1	97	296,2	8,99	0,96	0,06	40	2684518	☑
230321_AKo_Calibration_16min-MWi_39	Standard	RvD5	500,00	504,5	101	229,4	8,99	0,96	0,06	38	2668905	☑
230321_AKo_Calibration_16min-MWi_40	Standard	RvD5	500,00	490,3	98	339,2	8,99	0,96	0,05	30	2539859	☑
230321_AKo_Calibration_16min-MWi_41	Standard	RvD5	1000,00	948,5	95	387,3	8,99	0,96	0,06	32	4864276	☑
230321_AKo_Calibration_16min-MWi_42	Standard	RvD5	1000,00	949,3	95	260,5	8,99	0,96	0,06	31	4800018	☑
230321_AKo_Calibration_16min-MWi_43	Standard	RvD5	2000,00	2019,2	101	291,9	8,99	0,96	0,06	42	11801396	☑
230321_AKo_Calibration_16min-MWi_44	Standard	RvD5	2000,00	2016,9	101	287,3	8,99	0,96	0,06	43	11632360	☑
230321_AKo_Calibration_16min-MWi_45	Unknown	RvD5	N/A	1,3	N/A	7,1	8,99	0,96	0,04	34	8073	☑
Blank	Double Blank	RvD5	N/A	N/A	N/A	N/A	N/A	N/A	N/A	N/A	N/A	☑
Blank	Double Blank	RvD5	N/A	N/A	N/A	N/A	N/A	N/A	N/A	N/A	N/A	☑
Blank	Double Blank	RvD5	N/A	N/A	N/A	N/A	N/A	N/A	N/A	N/A	N/A	☑
Blank	Double Blank	RvD5	N/A	N/A	N/A	N/A	N/A	N/A	N/A	N/A	N/A	☑
Blank	Double Blank	RvD5	N/A	N/A	N/A	N/A	N/A	N/A	N/A	N/A	N/A	☑

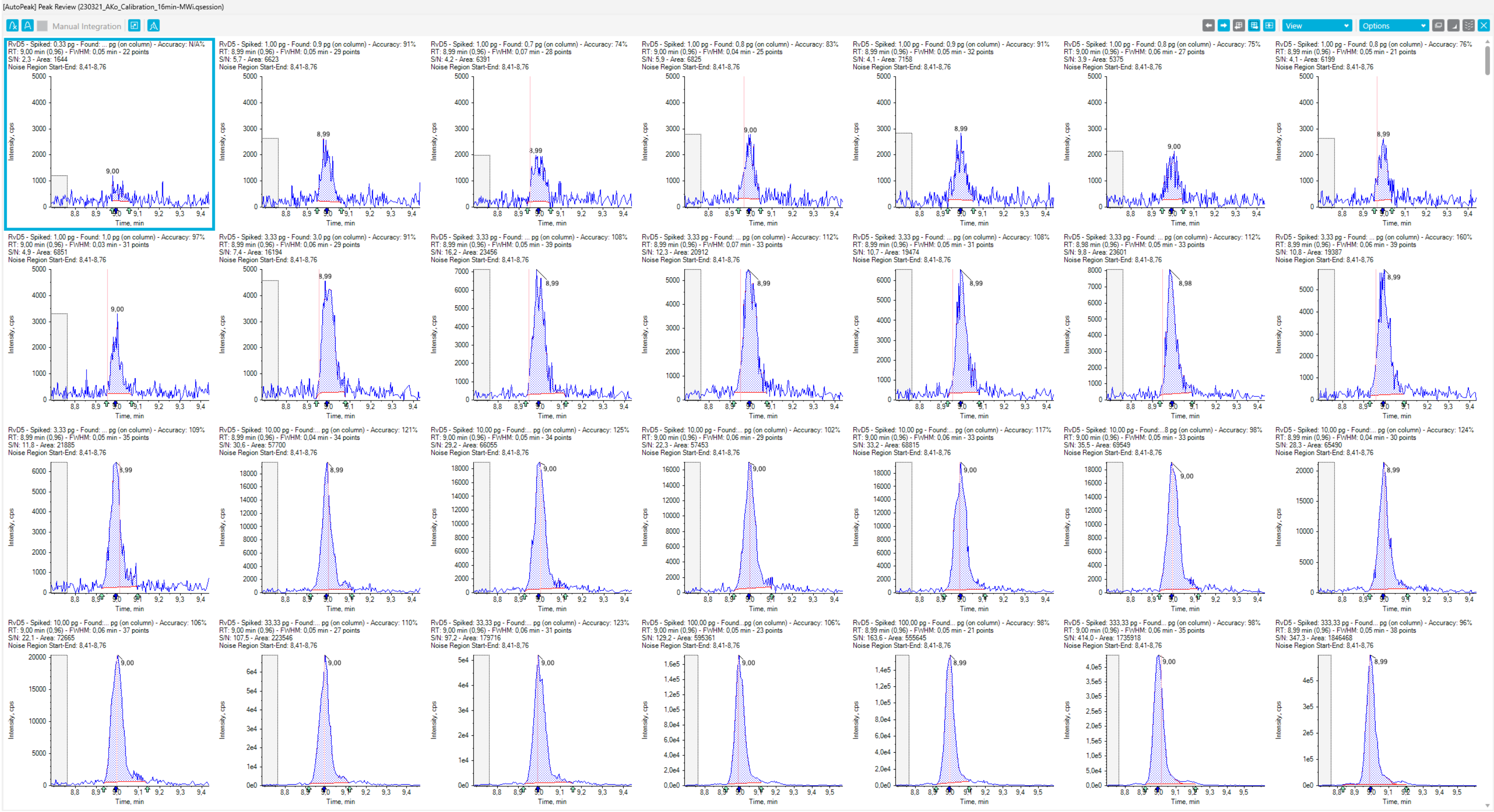
[AutoPeak] Peak Review (230321_AKo_Calibration_16min-MWi.qsession)
Manual Integration
View
Options

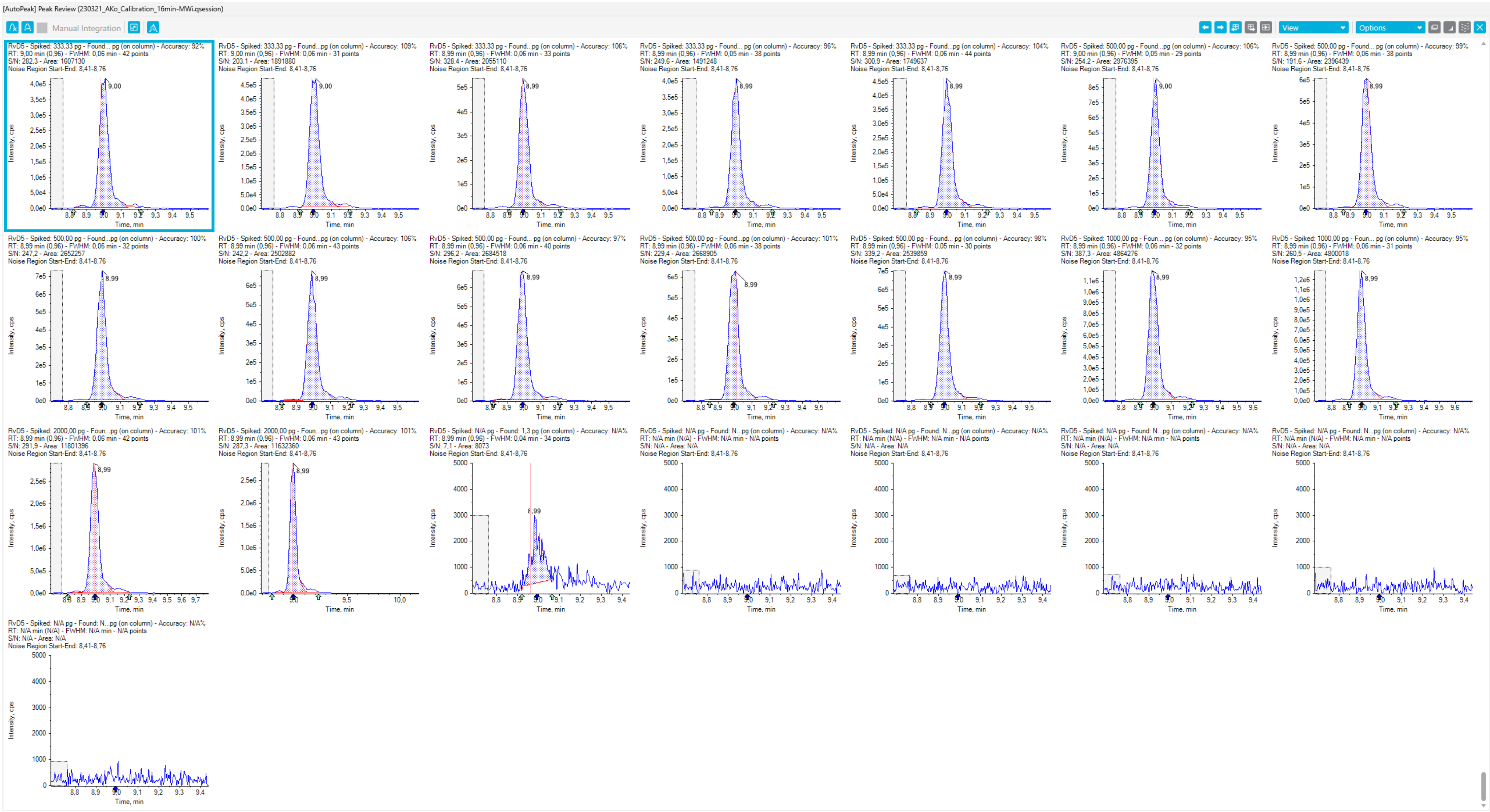

LXA$_5$

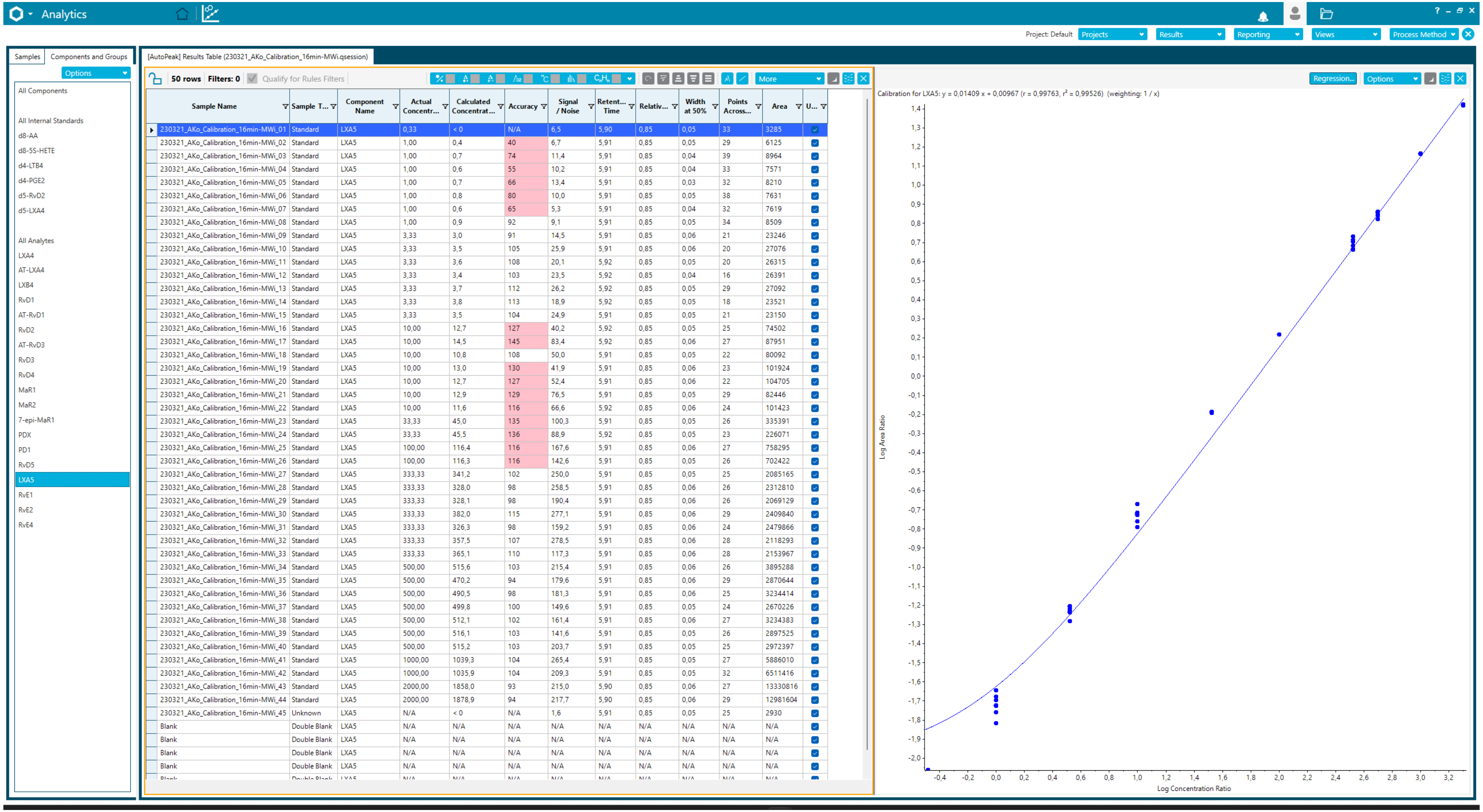

Sample Name	Sample T...	Component Name	Actual Concentr...	Calculated Concentrat...	Accuracy	Signal / Noise	Retent... Time	Relativ...	Width at 50%	Points Across...	Area	U...
230321_AKo_Calibration_16min-MWi_01	Standard	LXA5	0,33	< 0	N/A	6,5	5,90	0,85	0,05	33	3285	☑
230321_AKo_Calibration_16min-MWi_02	Standard	LXA5	1,00	0,4	40	6,7	5,91	0,85	0,05	29	6125	☑
230321_AKo_Calibration_16min-MWi_03	Standard	LXA5	1,00	0,7	74	11,4	5,91	0,85	0,04	39	8964	☑
230321_AKo_Calibration_16min-MWi_04	Standard	LXA5	1,00	0,6	55	10,2	5,91	0,85	0,04	33	7571	☑
230321_AKo_Calibration_16min-MWi_05	Standard	LXA5	1,00	0,7	66	13,4	5,91	0,85	0,03	32	8210	☑
230321_AKo_Calibration_16min-MWi_06	Standard	LXA5	1,00	0,8	80	10,0	5,91	0,85	0,05	38	7631	☑
230321_AKo_Calibration_16min-MWi_07	Standard	LXA5	1,00	0,6	65	5,3	5,91	0,85	0,04	32	7619	☑
230321_AKo_Calibration_16min-MWi_08	Standard	LXA5	1,00	0,9	92	9,1	5,91	0,85	0,05	34	8509	☑
230321_AKo_Calibration_16min-MWi_09	Standard	LXA5	3,33	3,0	91	14,5	5,91	0,85	0,06	21	23246	☑
230321_AKo_Calibration_16min-MWi_10	Standard	LXA5	3,33	3,5	105	25,9	5,91	0,85	0,06	20	27076	☑
230321_AKo_Calibration_16min-MWi_11	Standard	LXA5	3,33	3,6	108	20,1	5,92	0,85	0,05	20	26315	☑
230321_AKo_Calibration_16min-MWi_12	Standard	LXA5	3,33	3,4	103	23,5	5,92	0,85	0,04	16	26391	☑
230321_AKo_Calibration_16min-MWi_13	Standard	LXA5	3,33	3,7	112	26,2	5,92	0,85	0,05	29	27092	☑
230321_AKo_Calibration_16min-MWi_14	Standard	LXA5	3,33	3,8	113	18,9	5,92	0,85	0,05	18	23521	☑
230321_AKo_Calibration_16min-MWi_15	Standard	LXA5	3,33	3,5	104	24,9	5,91	0,85	0,05	21	23150	☑
230321_AKo_Calibration_16min-MWi_16	Standard	LXA5	10,00	12,7	127	40,2	5,92	0,85	0,05	25	74502	☑
230321_AKo_Calibration_16min-MWi_17	Standard	LXA5	10,00	14,5	145	83,4	5,92	0,85	0,06	27	87951	☑
230321_AKo_Calibration_16min-MWi_18	Standard	LXA5	10,00	10,8	108	50,0	5,91	0,85	0,05	22	80092	☑
230321_AKo_Calibration_16min-MWi_19	Standard	LXA5	10,00	13,0	130	41,9	5,91	0,85	0,06	23	101924	☑
230321_AKo_Calibration_16min-MWi_20	Standard	LXA5	10,00	12,7	127	52,4	5,91	0,85	0,06	22	104705	☑
230321_AKo_Calibration_16min-MWi_21	Standard	LXA5	10,00	12,9	129	76,5	5,91	0,85	0,05	29	82446	☑
230321_AKo_Calibration_16min-MWi_22	Standard	LXA5	10,00	11,6	116	66,6	5,92	0,85	0,06	24	101423	☑
230321_AKo_Calibration_16min-MWi_23	Standard	LXA5	33,33	45,0	135	100,3	5,91	0,85	0,05	26	335391	☑
230321_AKo_Calibration_16min-MWi_24	Standard	LXA5	33,33	45,5	136	88,9	5,92	0,85	0,05	23	226071	☑
230321_AKo_Calibration_16min-MWi_25	Standard	LXA5	100,00	116,4	116	167,6	5,91	0,85	0,06	27	758295	☑
230321_AKo_Calibration_16min-MWi_26	Standard	LXA5	100,00	116,3	116	142,6	5,91	0,85	0,05	26	702422	☑
230321_AKo_Calibration_16min-MWi_27	Standard	LXA5	333,33	341,2	102	250,0	5,91	0,85	0,05	25	2085165	☑
230321_AKo_Calibration_16min-MWi_28	Standard	LXA5	333,33	328,0	98	258,5	5,91	0,85	0,06	26	2312810	☑
230321_AKo_Calibration_16min-MWi_29	Standard	LXA5	333,33	328,1	98	190,4	5,91	0,85	0,06	26	2069129	☑
230321_AKo_Calibration_16min-MWi_30	Standard	LXA5	333,33	382,0	115	277,1	5,91	0,85	0,06	29	2409840	☑
230321_AKo_Calibration_16min-MWi_31	Standard	LXA5	333,33	326,3	98	159,2	5,91	0,85	0,06	24	2479866	☑
230321_AKo_Calibration_16min-MWi_32	Standard	LXA5	333,33	357,5	107	278,5	5,91	0,85	0,06	28	2118293	☑
230321_AKo_Calibration_16min-MWi_33	Standard	LXA5	333,33	365,1	110	117,3	5,91	0,85	0,06	28	2153967	☑
230321_AKo_Calibration_16min-MWi_34	Standard	LXA5	500,00	515,6	103	215,4	5,91	0,85	0,06	26	3895288	☑
230321_AKo_Calibration_16min-MWi_35	Standard	LXA5	500,00	470,2	94	179,6	5,91	0,85	0,06	29	2870644	☑
230321_AKo_Calibration_16min-MWi_36	Standard	LXA5	500,00	490,5	98	181,3	5,91	0,85	0,06	25	3234414	☑
230321_AKo_Calibration_16min-MWi_37	Standard	LXA5	500,00	499,8	100	149,6	5,91	0,85	0,05	24	2670226	☑
230321_AKo_Calibration_16min-MWi_38	Standard	LXA5	500,00	512,1	102	161,4	5,91	0,85	0,06	27	3234383	☑
230321_AKo_Calibration_16min-MWi_39	Standard	LXA5	500,00	516,1	103	141,6	5,91	0,85	0,05	26	2897525	☑
230321_AKo_Calibration_16min-MWi_40	Standard	LXA5	500,00	515,2	103	203,7	5,91	0,85	0,05	25	2972397	☑
230321_AKo_Calibration_16min-MWi_41	Standard	LXA5	1000,00	1039,3	104	265,4	5,91	0,85	0,05	27	5886010	☑
230321_AKo_Calibration_16min-MWi_42	Standard	LXA5	1000,00	1035,9	104	209,3	5,91	0,85	0,05	32	6511416	☑
230321_AKo_Calibration_16min-MWi_43	Standard	LXA5	2000,00	1858,0	93	215,0	5,90	0,85	0,06	27	13330816	☑
230321_AKo_Calibration_16min-MWi_44	Standard	LXA5	2000,00	1878,9	94	217,7	5,90	0,85	0,06	29	12981604	☑
230321_AKo_Calibration_16min-MWi_45	Unknown	LXA5	N/A	< 0	N/A	1,6	5,91	0,85	0,05	25	2930	☑
Blank	Double Blank	LXA5	N/A	N/A	N/A	N/A	N/A	N/A	N/A	N/A	N/A	☑
Blank	Double Blank	LXA5	N/A	N/A	N/A	N/A	N/A	N/A	N/A	N/A	N/A	☑
Blank	Double Blank	LXA5	N/A	N/A	N/A	N/A	N/A	N/A	N/A	N/A	N/A	☑
Blank	Double Blank	LXA5	N/A	N/A	N/A	N/A	N/A	N/A	N/A	N/A	N/A	☑
Blank	Double Blank	LXA5	N/A	N/A	N/A	N/A	N/A	N/A	N/A	N/A	N/A	[illegible]

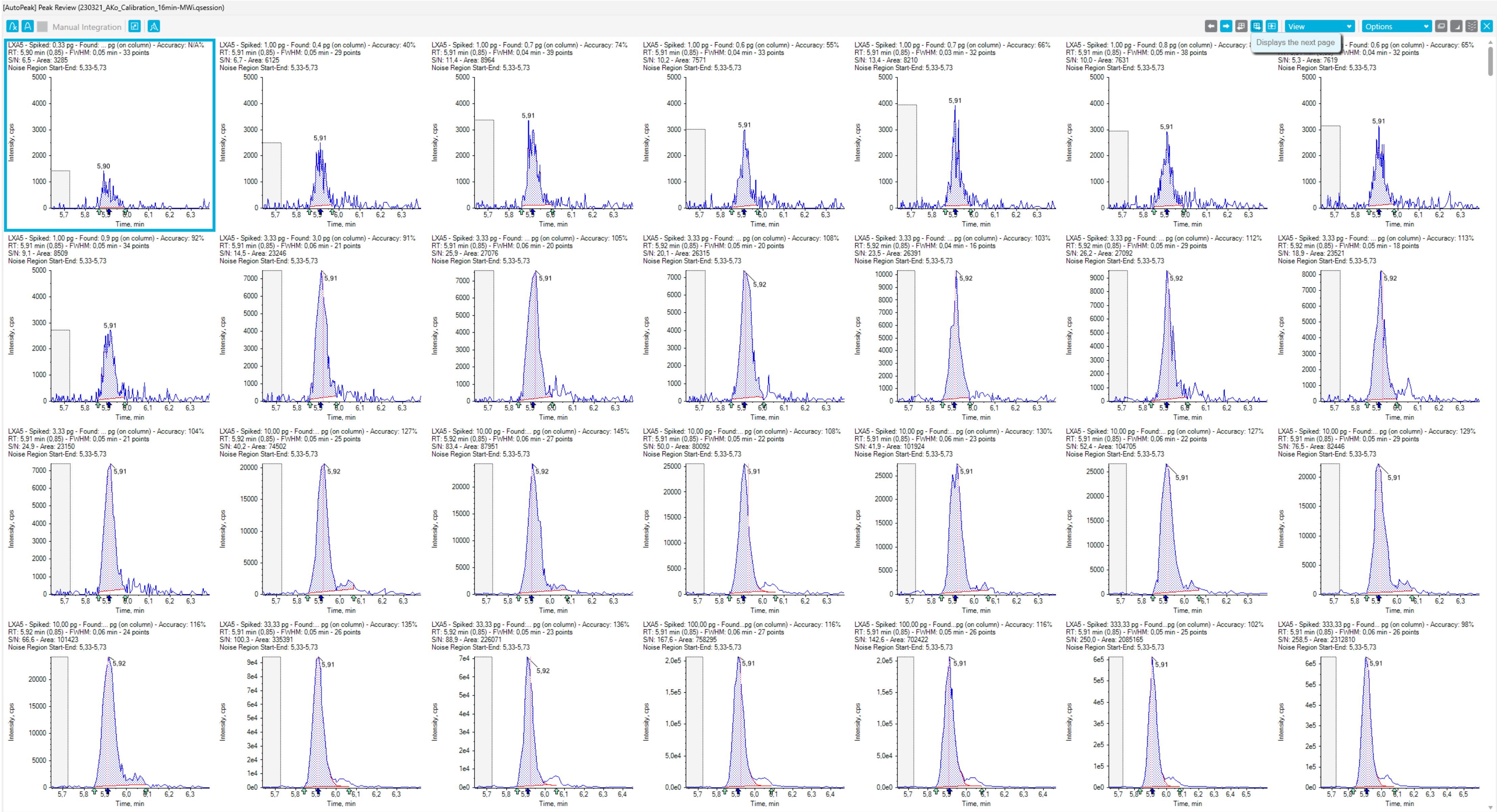
[AutoPeak] Peak Review (230321_AKo_Calibration_16min-MWi.qsession)

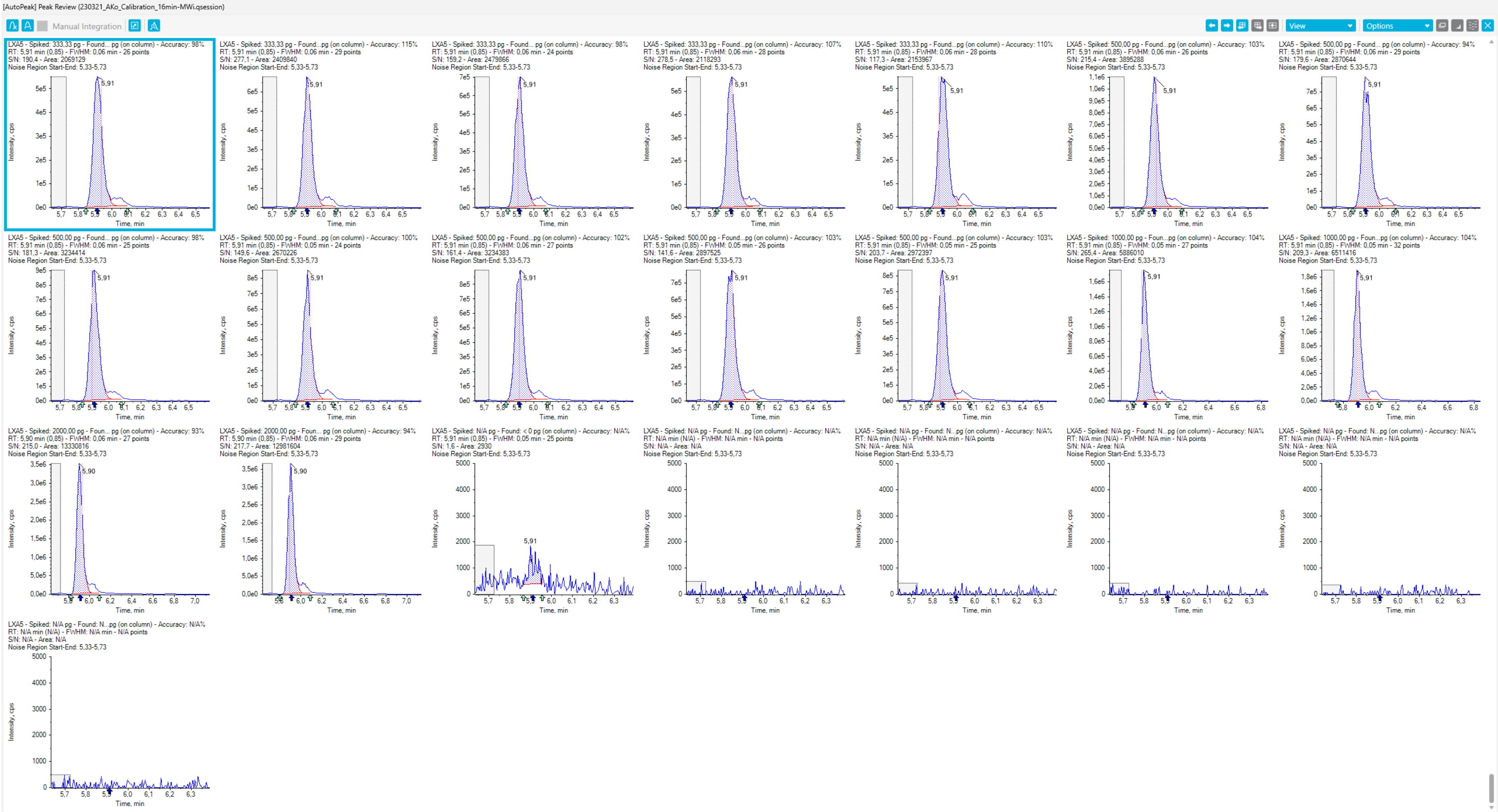
[AutoPeak] Peak Review (230321_AKo_Calibration_16min-MWi.qsession)
Manual Integration
View
Options
LXA5 - Spiked: 333,33 pg - Found... pg (on column) - Accuracy: 98%
RT: 5,91 min (0,85) - FWHM: 0,06 min - 26 points
S/N: 190,4 - Area: 2069129
Noise Region Start-End: 5,33-5,73
LXA5 - Spiked: 333,33 pg - Found...pg (on column) - Accuracy: 115%
RT: 5,91 min (0,85) - FWHM: 0,06 min - 29 points
S/N: 277,1 - Area: 2409840
Noise Region Start-End: 5,33-5,73
LXA5 - Spiked: 333,33 pg - Found... pg (on column) - Accuracy: 98%
RT: 5,91 min (0,85) - FWHM: 0,06 min - 24 points
S/N: 159,2 - Area: 2479866
Noise Region Start-End: 5,33-5,73
LXA5 - Spiked: 333,33 pg - Found...pg (on column) - Accuracy: 107%
RT: 5,91 min (0,85) - FWHM: 0,06 min - 28 points
S/N: 278,5 - Area: 2118293
Noise Region Start-End: 5,33-5,73
LXA5 - Spiked: 333,33 pg - Found...pg (on column) - Accuracy: 110%
RT: 5,91 min (0,85) - FWHM: 0,06 min - 28 points
S/N: 117,3 - Area: 2153967
Noise Region Start-End: 5,33-5,73
LXA5 - Spiked: 500,00 pg - Found...pg (on column) - Accuracy: 103%
RT: 5,91 min (0,85) - FWHM: 0,06 min - 26 points
S/N: 215,4 - Area: 3895288
Noise Region Start-End: 5,33-5,73
LXA5 - Spiked: 500,00 pg - Found... pg (on column) - Accuracy: 94%
RT: 5,91 min (0,85) - FWHM: 0,06 min - 29 points
S/N: 179,6 - Area: 2870644
Noise Region Start-End: 5,33-5,73
LXA5 - Spiked: 500,00 pg - Found... pg (on column) - Accuracy: 98%
RT: 5,91 min (0,85) - FWHM: 0,06 min - 25 points
S/N: 181,3 - Area: 3234414
Noise Region Start-End: 5,33-5,73
LXA5 - Spiked: 500,00 pg - Found...pg (on column) - Accuracy: 100%
RT: 5,91 min (0,85) - FWHM: 0,05 min - 24 points
S/N: 149,6 - Area: 2670226
Noise Region Start-End: 5,33-5,73
LXA5 - Spiked: 500,00 pg - Found...pg (on column) - Accuracy: 102%
RT: 5,91 min (0,85) - FWHM: 0,06 min - 27 points
S/N: 161,4 - Area: 3234383
Noise Region Start-End: 5,33-5,73
LXA5 - Spiked: 500,00 pg - Found...pg (on column) - Accuracy: 103%
RT: 5,91 min (0,85) - FWHM: 0,05 min - 26 points
S/N: 141,6 - Area: 2897525
Noise Region Start-End: 5,33-5,73
LXA5 - Spiked: 500,00 pg - Found...pg (on column) - Accuracy: 103%
RT: 5,91 min (0,85) - FWHM: 0,05 min - 25 points
S/N: 203,7 - Area: 2972397
Noise Region Start-End: 5,33-5,73
LXA5 - Spiked: 1000,00 pg - Foun...pg (on column) - Accuracy: 104%
RT: 5,91 min (0,85) - FWHM: 0,05 min - 27 points
S/N: 265,4 - Area: 5886010
Noise Region Start-End: 5,33-5,73
LXA5 - Spiked: 1000,00 pg - Foun...pg (on column) - Accuracy: 104%
RT: 5,91 min (0,85) - FWHM: 0,05 min - 32 points
S/N: 209,3 - Area: 6511416
Noise Region Start-End: 5,33-5,73
LXA5 - Spiked: 2000,00 pg - Foun... pg (on column) - Accuracy: 93%
RT: 5,90 min (0,85) - FWHM: 0,06 min - 27 points
S/N: 215,0 - Area: 13330816
Noise Region Start-End: 5,33-5,73
LXA5 - Spiked: 2000,00 pg - Foun... pg (on column) - Accuracy: 94%
RT: 5,90 min (0,85) - FWHM: 0,06 min - 29 points
S/N: 217,7 - Area: 12981604
Noise Region Start-End: 5,33-5,73
LXA5 - Spiked: N/A pg - Found: < 0 pg (on column) - Accuracy: N/A%
RT: 5,91 min (0,85) - FWHM: 0,05 min - 25 points
S/N: 1,6 - Area: 2930
Noise Region Start-End: 5,33-5,73
LXA5 - Spiked: N/A pg - Found: N...pg (on column) - Accuracy: N/A%
RT: N/A min (N/A) - FWHM: N/A min - N/A points
S/N: N/A - Area: N/A
Noise Region Start-End: 5,33-5,73
LXA5 - Spiked: N/A pg - Found: N...pg (on column) - Accuracy: N/A%
RT: N/A min (N/A) - FWHM: N/A min - N/A points
S/N: N/A - Area: N/A
Noise Region Start-End: 5,33-5,73
LXA5 - Spiked: N/A pg - Found: N...pg (on column) - Accuracy: N/A%
RT: N/A min (N/A) - FWHM: N/A min - N/A points
S/N: N/A - Area: N/A
Noise Region Start-End: 5,33-5,73
LXA5 - Spiked: N/A pg - Found: N...pg (on column) - Accuracy: N/A%
RT: N/A min (N/A) - FWHM: N/A min - N/A points
S/N: N/A - Area: N/A
Noise Region Start-End: 5,33-5,73
LXA5 - Spiked: N/A pg - Found: N...pg (on column) - Accuracy: N/A%
RT: N/A min (N/A) - FWHM: N/A min - N/A points
S/N: N/A - Area: N/A
Noise Region Start-End: 5,33-5,73
Intensity, cps
Time, min

RvE1

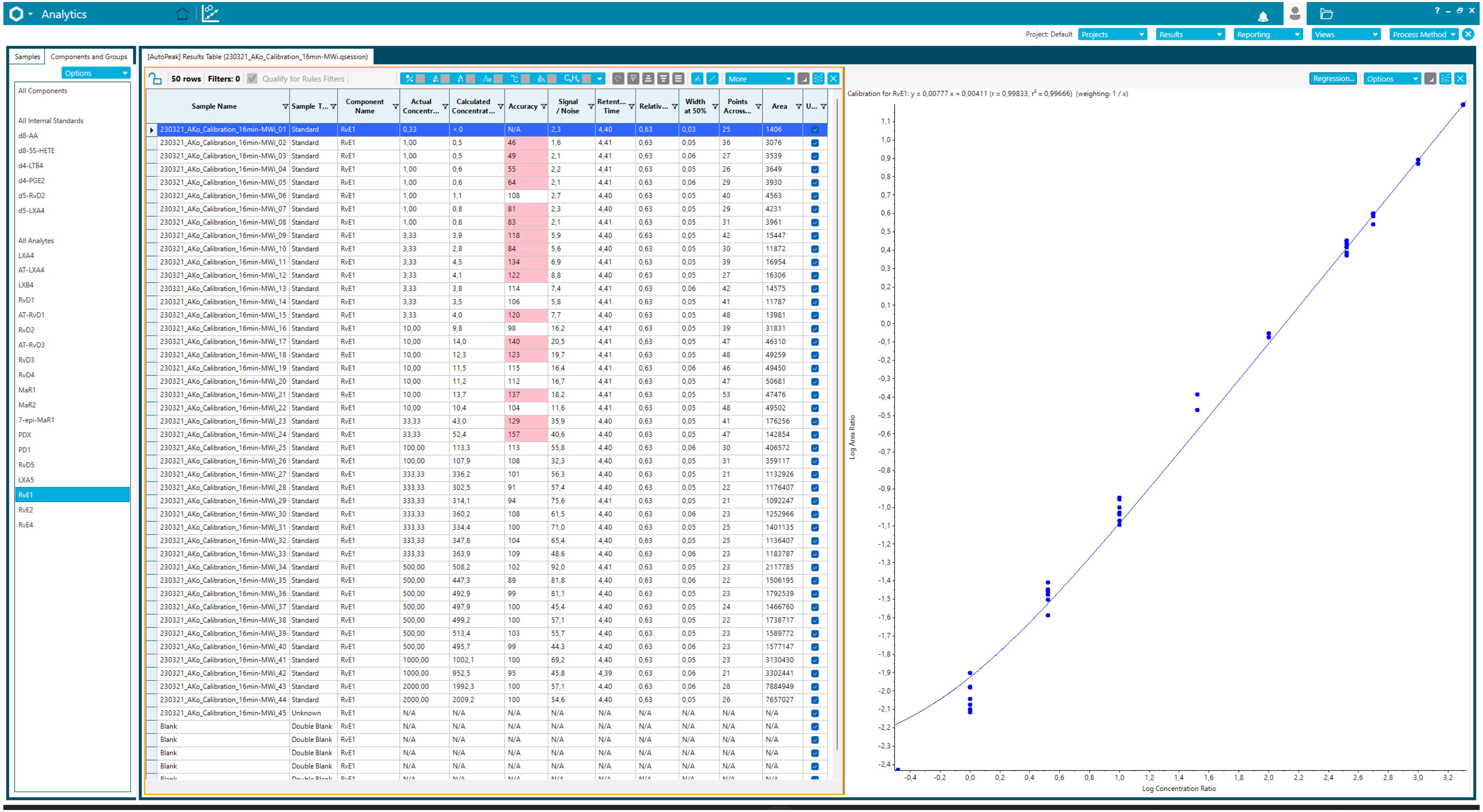

Sample Name	Sample T...	Component Name	Actual Concentr...	Calculated Concentrat...	Accuracy	Signal / Noise	Retent... Time	Relativ...	Width at 50%	Points Across...	Area	U...
230321_AKo_Calibration_16min-MWi_01	Standard	RvE1	0,33	< 0	N/A	2,3	4,40	0,63	0,03	25	1406	✓
230321_AKo_Calibration_16min-MWi_02	Standard	RvE1	1,00	0,5	46	1,6	4,41	0,63	0,05	36	3076	✓
230321_AKo_Calibration_16min-MWi_03	Standard	RvE1	1,00	0,5	49	2,1	4,41	0,63	0,06	27	3539	✓
230321_AKo_Calibration_16min-MWi_04	Standard	RvE1	1,00	0,6	55	2,2	4,41	0,63	0,05	26	3649	✓
230321_AKo_Calibration_16min-MWi_05	Standard	RvE1	1,00	0,6	64	2,1	4,41	0,63	0,06	29	3930	✓
230321_AKo_Calibration_16min-MWi_06	Standard	RvE1	1,00	1,1	108	2,7	4,40	0,63	0,05	40	4563	✓
230321_AKo_Calibration_16min-MWi_07	Standard	RvE1	1,00	0,8	81	2,3	4,40	0,63	0,05	29	4231	✓
230321_AKo_Calibration_16min-MWi_08	Standard	RvE1	1,00	0,8	83	2,1	4,41	0,63	0,05	31	3961	✓
230321_AKo_Calibration_16min-MWi_09	Standard	RvE1	3,33	3,9	118	5,9	4,40	0,63	0,05	42	15447	✓
230321_AKo_Calibration_16min-MWi_10	Standard	RvE1	3,33	2,8	84	5,6	4,40	0,63	0,05	30	11872	✓
230321_AKo_Calibration_16min-MWi_11	Standard	RvE1	3,33	4,5	134	6,9	4,41	0,63	0,05	39	16954	✓
230321_AKo_Calibration_16min-MWi_12	Standard	RvE1	3,33	4,1	122	8,8	4,40	0,63	0,05	27	16306	✓
230321_AKo_Calibration_16min-MWi_13	Standard	RvE1	3,33	3,8	114	7,4	4,41	0,63	0,06	42	14575	✓
230321_AKo_Calibration_16min-MWi_14	Standard	RvE1	3,33	3,5	106	5,8	4,41	0,63	0,05	41	11787	✓
230321_AKo_Calibration_16min-MWi_15	Standard	RvE1	3,33	4,0	120	7,7	4,40	0,63	0,05	48	13981	✓
230321_AKo_Calibration_16min-MWi_16	Standard	RvE1	10,00	9,8	98	16,2	4,41	0,63	0,05	39	31831	✓
230321_AKo_Calibration_16min-MWi_17	Standard	RvE1	10,00	14,0	140	20,5	4,41	0,63	0,05	47	46310	✓
230321_AKo_Calibration_16min-MWi_18	Standard	RvE1	10,00	12,3	123	19,7	4,41	0,63	0,05	48	49259	✓
230321_AKo_Calibration_16min-MWi_19	Standard	RvE1	10,00	11,5	115	16,4	4,41	0,63	0,06	46	49450	✓
230321_AKo_Calibration_16min-MWi_20	Standard	RvE1	10,00	11,2	112	16,7	4,41	0,63	0,05	47	50681	✓
230321_AKo_Calibration_16min-MWi_21	Standard	RvE1	10,00	13,7	137	18,2	4,41	0,63	0,05	53	47476	✓
230321_AKo_Calibration_16min-MWi_22	Standard	RvE1	10,00	10,4	104	11,6	4,41	0,63	0,05	48	49502	✓
230321_AKo_Calibration_16min-MWi_23	Standard	RvE1	33,33	43,0	129	35,9	4,40	0,63	0,05	41	176256	✓
230321_AKo_Calibration_16min-MWi_24	Standard	RvE1	33,33	52,4	157	40,6	4,40	0,63	0,05	47	142854	✓
230321_AKo_Calibration_16min-MWi_25	Standard	RvE1	100,00	113,3	113	55,8	4,40	0,63	0,06	30	406572	✓
230321_AKo_Calibration_16min-MWi_26	Standard	RvE1	100,00	107,9	108	32,3	4,40	0,63	0,05	31	359117	✓
230321_AKo_Calibration_16min-MWi_27	Standard	RvE1	333,33	336,2	101	56,3	4,40	0,63	0,05	21	1132926	✓
230321_AKo_Calibration_16min-MWi_28	Standard	RvE1	333,33	302,5	91	57,4	4,40	0,63	0,05	22	1176407	✓
230321_AKo_Calibration_16min-MWi_29	Standard	RvE1	333,33	314,1	94	75,6	4,41	0,63	0,05	21	1092247	✓
230321_AKo_Calibration_16min-MWi_30	Standard	RvE1	333,33	360,2	108	61,5	4,40	0,63	0,06	23	1252966	✓
230321_AKo_Calibration_16min-MWi_31	Standard	RvE1	333,33	334,4	100	71,0	4,40	0,63	0,05	25	1401135	✓
230321_AKo_Calibration_16min-MWi_32	Standard	RvE1	333,33	347,8	104	65,4	4,40	0,63	0,05	25	1136407	✓
230321_AKo_Calibration_16min-MWi_33	Standard	RvE1	333,33	363,9	109	48,6	4,40	0,63	0,06	23	1183787	✓
230321_AKo_Calibration_16min-MWi_34	Standard	RvE1	500,00	508,2	102	92,0	4,41	0,63	0,05	23	2117785	✓
230321_AKo_Calibration_16min-MWi_35	Standard	RvE1	500,00	447,3	89	81,8	4,40	0,63	0,06	22	1506195	✓
230321_AKo_Calibration_16min-MWi_36	Standard	RvE1	500,00	492,9	99	81,1	4,40	0,63	0,05	23	1792539	✓
230321_AKo_Calibration_16min-MWi_37	Standard	RvE1	500,00	497,9	100	45,4	4,40	0,63	0,05	24	1466760	✓
230321_AKo_Calibration_16min-MWi_38	Standard	RvE1	500,00	499,2	100	57,1	4,40	0,63	0,05	22	1738717	✓
230321_AKo_Calibration_16min-MWi_39	Standard	RvE1	500,00	513,4	103	55,7	4,40	0,63	0,05	23	1589772	✓
230321_AKo_Calibration_16min-MWi_40	Standard	RvE1	500,00	495,7	99	44,3	4,40	0,63	0,06	23	1577147	✓
230321_AKo_Calibration_16min-MWi_41	Standard	RvE1	1000,00	1002,1	100	69,2	4,40	0,63	0,05	23	3130430	✓
230321_AKo_Calibration_16min-MWi_42	Standard	RvE1	1000,00	952,5	95	45,8	4,39	0,63	0,06	21	3302441	✓
230321_AKo_Calibration_16min-MWi_43	Standard	RvE1	2000,00	1992,3	100	57,1	4,40	0,63	0,06	28	7884949	✓
230321_AKo_Calibration_16min-MWi_44	Standard	RvE1	2000,00	2009,2	100	54,6	4,40	0,63	0,05	26	7657027	✓
230321_AKo_Calibration_16min-MWi_45	Unknown	RvE1	N/A	N/A	N/A	N/A	N/A	N/A	N/A	N/A	N/A	✓
Blank	Double Blank	RvE1	N/A	N/A	N/A	N/A	N/A	N/A	N/A	N/A	N/A	✓
Blank	Double Blank	RvE1	N/A	N/A	N/A	N/A	N/A	N/A	N/A	N/A	N/A	✓
Blank	Double Blank	RvE1	N/A	N/A	N/A	N/A	N/A	N/A	N/A	N/A	N/A	✓
Blank	Double Blank	RvE1	N/A	N/A	N/A	N/A	N/A	N/A	N/A	N/A	N/A	✓
Blank	Double Blank	RvE1	N/A	N/A	N/A	N/A	N/A	N/A	N/A	N/A	N/A	✓

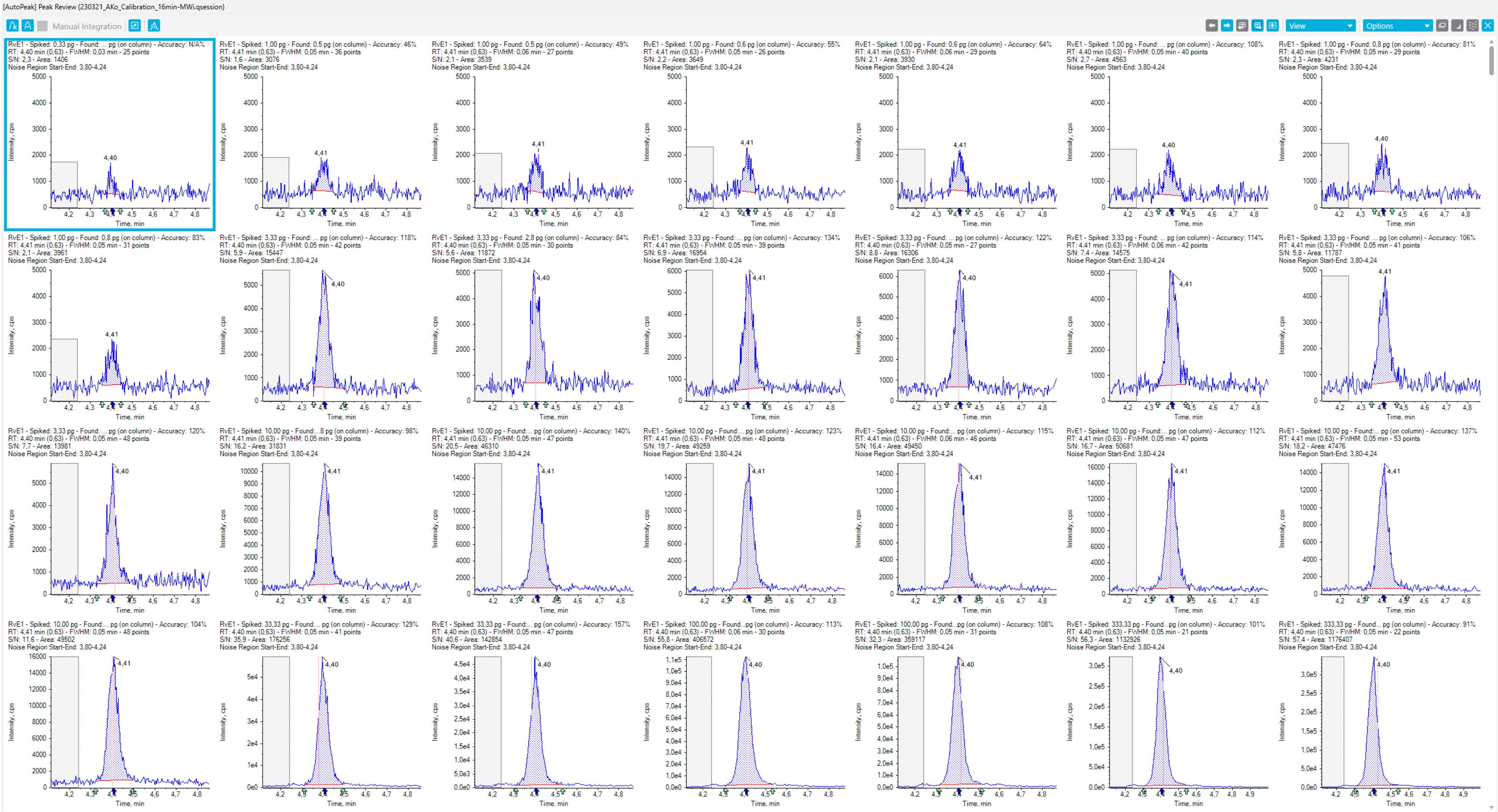
[AutoPeak] Peak Review (230321_AKo_Calibration_16min-MWi.qsession)
Manual Integration
View
Options
RvE1 - Spiked: 0.33 pg - Found: ... pg (on column) - Accuracy: N/A%
RT: 4.40 min (0.63) - FWHM: 0.03 min - 25 points
S/N: 2.3 - Area: 1406
Noise Region Start-End: 3.80-4.24
RvE1 - Spiked: 1.00 pg - Found: 0.5 pg (on column) - Accuracy: 46%
RT: 4.41 min (0.63) - FWHM: 0.05 min - 36 points
S/N: 1.6 - Area: 3076
RvE1 - Spiked: 1.00 pg - Found: 0.5 pg (on column) - Accuracy: 49%
RT: 4.41 min (0.63) - FWHM: 0.06 min - 27 points
S/N: 2.1 - Area: 3539
RvE1 - Spiked: 1.00 pg - Found: 0.6 pg (on column) - Accuracy: 55%
RT: 4.41 min (0.63) - FWHM: 0.05 min - 26 points
S/N: 2.2 - Area: 3649
RvE1 - Spiked: 1.00 pg - Found: 0.6 pg (on column) - Accuracy: 64%
RT: 4.41 min (0.63) - FWHM: 0.06 min - 29 points
S/N: 2.1 - Area: 3930
RvE1 - Spiked: 1.00 pg - Found: ... pg (on column) - Accuracy: 108%
RT: 4.40 min (0.63) - FWHM: 0.05 min - 40 points
S/N: 2.7 - Area: 4563
RvE1 - Spiked: 1.00 pg - Found: 0.8 pg (on column) - Accuracy: 81%
RT: 4.40 min (0.63) - FWHM: 0.05 min - 29 points
S/N: 2.3 - Area: 4231
RvE1 - Spiked: 1.00 pg - Found: 0.8 pg (on column) - Accuracy: 83%
RT: 4.41 min (0.63) - FWHM: 0.05 min - 31 points
S/N: 2.1 - Area: 3961
RvE1 - Spiked: 3.33 pg - Found: ... pg (on column) - Accuracy: 118%
RT: 4.40 min (0.63) - FWHM: 0.05 min - 42 points
S/N: 5.9 - Area: 15447
RvE1 - Spiked: 3.33 pg - Found: 2.8 pg (on column) - Accuracy: 84%
RT: 4.40 min (0.63) - FWHM: 0.05 min - 30 points
S/N: 5.6 - Area: 11872
RvE1 - Spiked: 3.33 pg - Found: ... pg (on column) - Accuracy: 134%
RT: 4.41 min (0.63) - FWHM: 0.05 min - 39 points
S/N: 6.9 - Area: 16954
RvE1 - Spiked: 3.33 pg - Found: ... pg (on column) - Accuracy: 122%
RT: 4.40 min (0.63) - FWHM: 0.05 min - 27 points
S/N: 8.8 - Area: 16306
RvE1 - Spiked: 3.33 pg - Found: ... pg (on column) - Accuracy: 114%
RT: 4.41 min (0.63) - FWHM: 0.06 min - 42 points
S/N: 7.4 - Area: 14575
RvE1 - Spiked: 3.33 pg - Found: ... pg (on column) - Accuracy: 106%
RT: 4.41 min (0.63) - FWHM: 0.05 min - 41 points
S/N: 5.8 - Area: 11787
RvE1 - Spiked: 3.33 pg - Found: ... pg (on column) - Accuracy: 120%
RT: 4.40 min (0.63) - FWHM: 0.05 min - 48 points
S/N: 7.7 - Area: 13981
RvE1 - Spiked: 10.00 pg - Found: ...8 pg (on column) - Accuracy: 98%
RT: 4.41 min (0.63) - FWHM: 0.05 min - 39 points
S/N: 16.2 - Area: 31831
RvE1 - Spiked: 10.00 pg - Found: ... pg (on column) - Accuracy: 140%
RT: 4.41 min (0.63) - FWHM: 0.05 min - 47 points
S/N: 20.5 - Area: 46310
RvE1 - Spiked: 10.00 pg - Found: ... pg (on column) - Accuracy: 123%
RT: 4.41 min (0.63) - FWHM: 0.05 min - 48 points
S/N: 19.7 - Area: 49259
RvE1 - Spiked: 10.00 pg - Found: ... pg (on column) - Accuracy: 115%
RT: 4.41 min (0.63) - FWHM: 0.06 min - 46 points
S/N: 16.4 - Area: 49450
RvE1 - Spiked: 10.00 pg - Found: ... pg (on column) - Accuracy: 112%
RT: 4.41 min (0.63) - FWHM: 0.05 min - 47 points
S/N: 16.7 - Area: 50681
RvE1 - Spiked: 10.00 pg - Found: ... pg (on column) - Accuracy: 137%
RT: 4.41 min (0.63) - FWHM: 0.05 min - 53 points
S/N: 18.2 - Area: 47476
RvE1 - Spiked: 10.00 pg - Found: ... pg (on column) - Accuracy: 104%
RT: 4.41 min (0.63) - FWHM: 0.05 min - 48 points
S/N: 11.6 - Area: 49502
RvE1 - Spiked: 33.33 pg - Found: ... pg (on column) - Accuracy: 129%
RT: 4.40 min (0.63) - FWHM: 0.05 min - 41 points
S/N: 35.9 - Area: 176256
RvE1 - Spiked: 33.33 pg - Found: ... pg (on column) - Accuracy: 157%
RT: 4.40 min (0.63) - FWHM: 0.05 min - 47 points
S/N: 40.6 - Area: 142854
RvE1 - Spiked: 100.00 pg - Found: ... pg (on column) - Accuracy: 113%
RT: 4.40 min (0.63) - FWHM: 0.06 min - 30 points
S/N: 55.8 - Area: 406572
RvE1 - Spiked: 100.00 pg - Found: ...pg (on column) - Accuracy: 108%
RT: 4.40 min (0.63) - FWHM: 0.05 min - 31 points
S/N: 32.3 - Area: 359117
RvE1 - Spiked: 333.33 pg - Found: ...pg (on column) - Accuracy: 101%
RT: 4.40 min (0.63) - FWHM: 0.05 min - 21 points
S/N: 56.3 - Area: 1132926
RvE1 - Spiked: 333.33 pg - Found: ... pg (on column) - Accuracy: 91%
RT: 4.40 min (0.63) - FWHM: 0.05 min - 22 points
S/N: 57.4 - Area: 1176407
Intensity, cps
Time, min

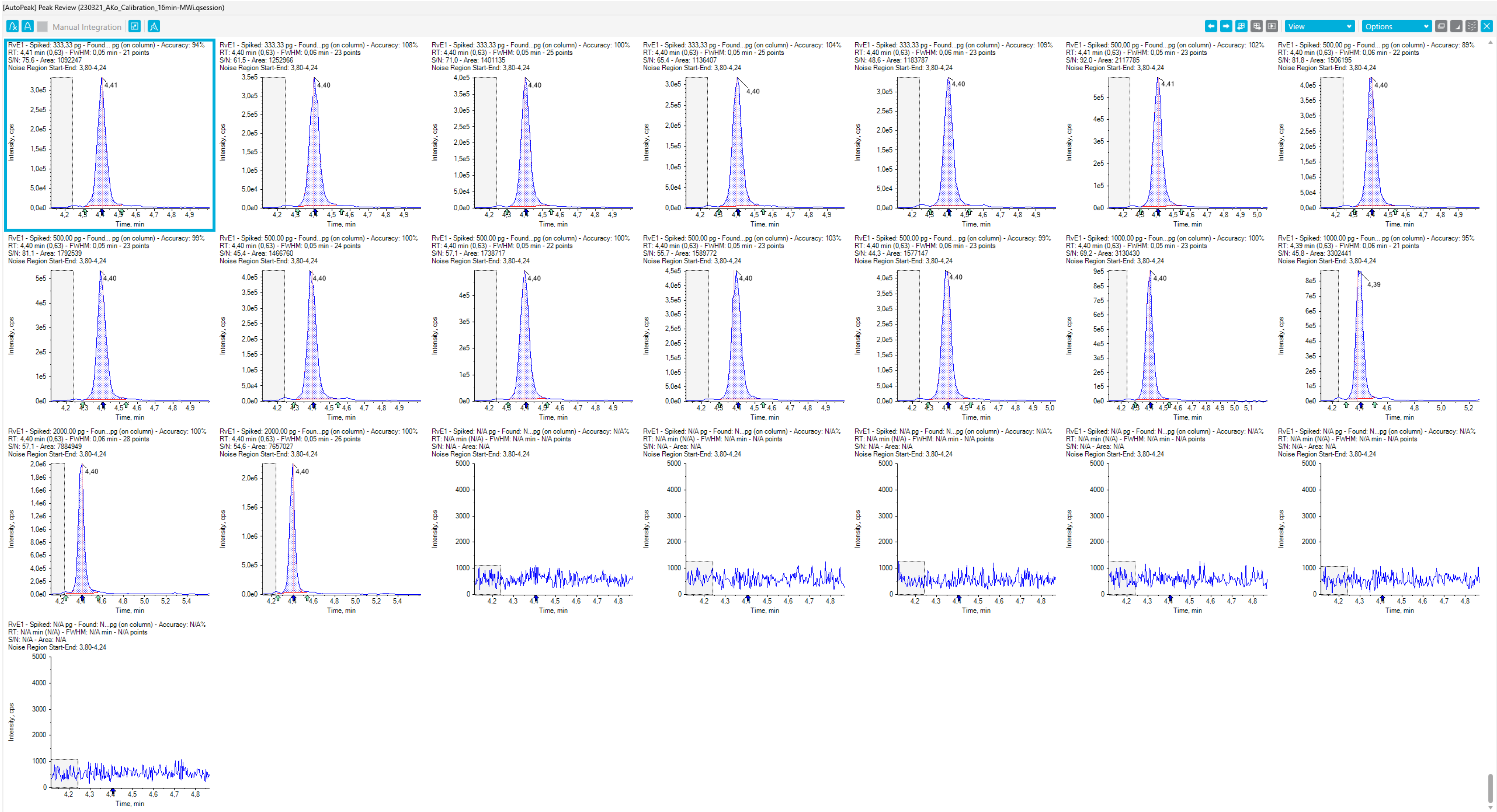
[AutoPeak] Peak Review (230321_AKo_Calibration_16min-MWi.qsession)
Manual Integration
View
Options
RvE1 - Spiked: 333,33 pg - Found... pg (on column) - Accuracy: 94%
RT: 4,41 min (0,63) - FWHM: 0,05 min - 21 points
S/N: 75,6 - Area: 1092247
Noise Region Start-End: 3,80-4,24
RvE1 - Spiked: 333,33 pg - Found...pg (on column) - Accuracy: 108%
RT: 4,40 min (0,63) - FWHM: 0,06 min - 23 points
S/N: 61,5 - Area: 1252966
Noise Region Start-End: 3,80-4,24
RvE1 - Spiked: 333,33 pg - Found...pg (on column) - Accuracy: 100%
RT: 4,40 min (0,63) - FWHM: 0,05 min - 25 points
S/N: 71,0 - Area: 1401135
Noise Region Start-End: 3,80-4,24
RvE1 - Spiked: 333,33 pg - Found...pg (on column) - Accuracy: 104%
RT: 4,40 min (0,63) - FWHM: 0,05 min - 25 points
S/N: 65,4 - Area: 1136407
Noise Region Start-End: 3,80-4,24
RvE1 - Spiked: 333,33 pg - Found...pg (on column) - Accuracy: 109%
RT: 4,40 min (0,63) - FWHM: 0,06 min - 23 points
S/N: 48,6 - Area: 1183787
Noise Region Start-End: 3,80-4,24
RvE1 - Spiked: 500,00 pg - Found...pg (on column) - Accuracy: 102%
RT: 4,41 min (0,63) - FWHM: 0,05 min - 23 points
S/N: 92,0 - Area: 2117785
Noise Region Start-End: 3,80-4,24
RvE1 - Spiked: 500,00 pg - Found... pg (on column) - Accuracy: 89%
RT: 4,40 min (0,63) - FWHM: 0,06 min - 22 points
S/N: 81,8 - Area: 1506195
Noise Region Start-End: 3,80-4,24
RvE1 - Spiked: 500,00 pg - Found... pg (on column) - Accuracy: 99%
RT: 4,40 min (0,63) - FWHM: 0,05 min - 23 points
S/N: 81,1 - Area: 1792539
Noise Region Start-End: 3,80-4,24
RvE1 - Spiked: 500,00 pg - Found...pg (on column) - Accuracy: 100%
RT: 4,40 min (0,63) - FWHM: 0,05 min - 24 points
S/N: 45,4 - Area: 1466760
Noise Region Start-End: 3,80-4,24
RvE1 - Spiked: 500,00 pg - Found...pg (on column) - Accuracy: 100%
RT: 4,40 min (0,63) - FWHM: 0,05 min - 22 points
S/N: 57,1 - Area: 1738717
Noise Region Start-End: 3,80-4,24
RvE1 - Spiked: 500,00 pg - Found...pg (on column) - Accuracy: 103%
RT: 4,40 min (0,63) - FWHM: 0,05 min - 23 points
S/N: 55,7 - Area: 1589772
Noise Region Start-End: 3,80-4,24
RvE1 - Spiked: 500,00 pg - Found... pg (on column) - Accuracy: 99%
RT: 4,40 min (0,63) - FWHM: 0,06 min - 23 points
S/N: 44,3 - Area: 1577147
Noise Region Start-End: 3,80-4,24
RvE1 - Spiked: 1000,00 pg - Foun...pg (on column) - Accuracy: 100%
RT: 4,40 min (0,63) - FWHM: 0,05 min - 23 points
S/N: 69,2 - Area: 3130430
Noise Region Start-End: 3,80-4,24
RvE1 - Spiked: 1000,00 pg - Foun... pg (on column) - Accuracy: 95%
RT: 4,39 min (0,63) - FWHM: 0,06 min - 21 points
S/N: 45,8 - Area: 3302441
Noise Region Start-End: 3,80-4,24
RvE1 - Spiked: 2000,00 pg - Foun...pg (on column) - Accuracy: 100%
RT: 4,40 min (0,63) - FWHM: 0,06 min - 28 points
S/N: 57,1 - Area: 7884949
Noise Region Start-End: 3,80-4,24
RvE1 - Spiked: 2000,00 pg - Foun...pg (on column) - Accuracy: 100%
RT: 4,40 min (0,63) - FWHM: 0,05 min - 26 points
S/N: 54,6 - Area: 7657027
Noise Region Start-End: 3,80-4,24
RvE1 - Spiked: N/A pg - Found: N...pg (on column) - Accuracy: N/A%
RT: N/A min (N/A) - FWHM: N/A min - N/A points
S/N: N/A - Area: N/A
Noise Region Start-End: 3,80-4,24
RvE1 - Spiked: N/A pg - Found: N...pg (on column) - Accuracy: N/A%
RT: N/A min (N/A) - FWHM: N/A min - N/A points
S/N: N/A - Area: N/A
Noise Region Start-End: 3,80-4,24
RvE1 - Spiked: N/A pg - Found: N...pg (on column) - Accuracy: N/A%
RT: N/A min (N/A) - FWHM: N/A min - N/A points
S/N: N/A - Area: N/A
Noise Region Start-End: 3,80-4,24
RvE1 - Spiked: N/A pg - Found: N...pg (on column) - Accuracy: N/A%
RT: N/A min (N/A) - FWHM: N/A min - N/A points
S/N: N/A - Area: N/A
Noise Region Start-End: 3,80-4,24
RvE1 - Spiked: N/A pg - Found: N...pg (on column) - Accuracy: N/A%
RT: N/A min (N/A) - FWHM: N/A min - N/A points
S/N: N/A - Area: N/A
Noise Region Start-End: 3,80-4,24
RvE1 - Spiked: N/A pg - Found: N...pg (on column) - Accuracy: N/A%
RT: N/A min (N/A) - FWHM: N/A min - N/A points
S/N: N/A - Area: N/A
Noise Region Start-End: 3,80-4,24
Intensity, cps
Time, min

RvE2

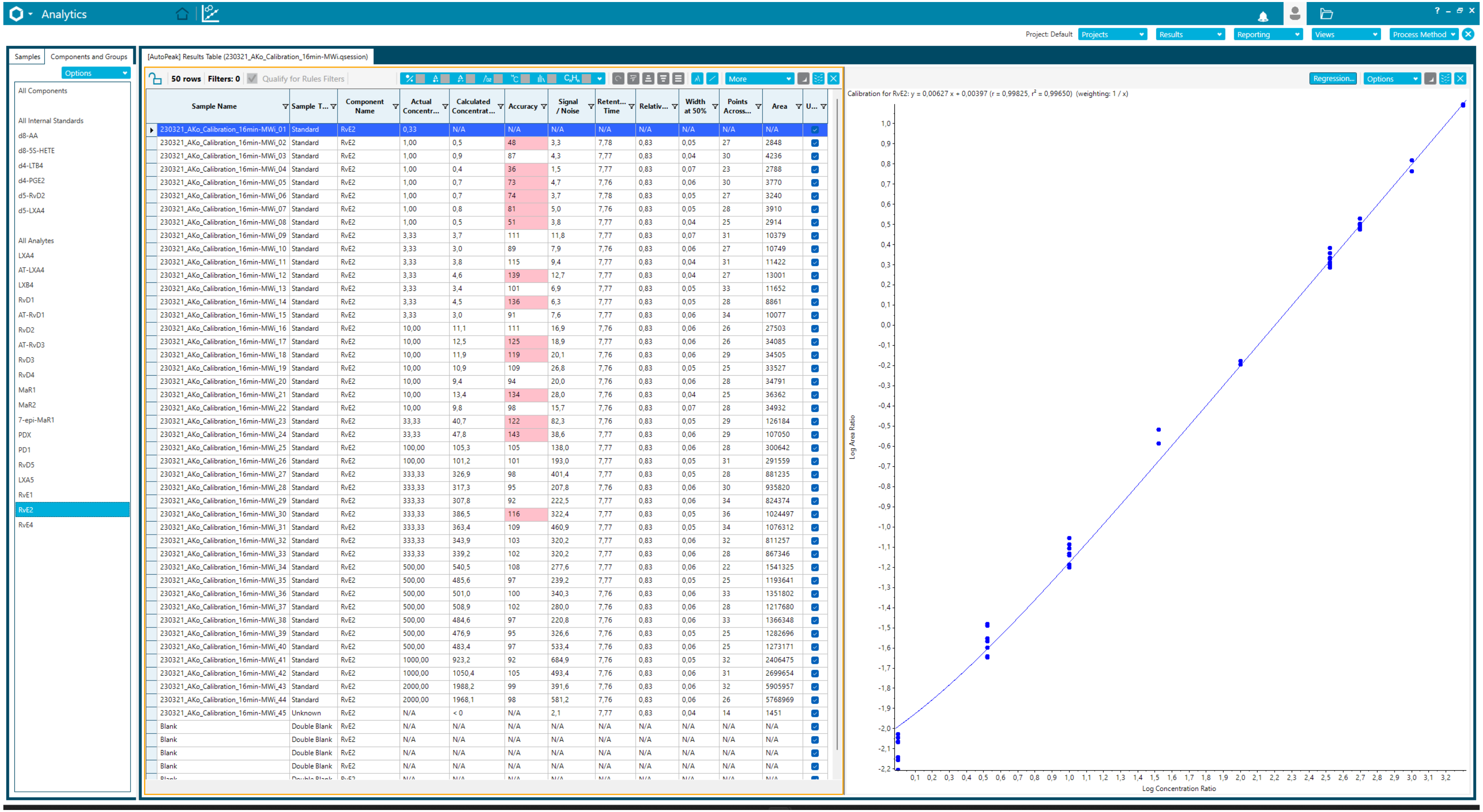

Sample Name	Sample T...	Component Name	Actual Concentr...	Calculated Concentrat...	Accuracy	Signal / Noise	Retent... Time	Relativ...	Width at 50%	Points Across...	Area	U...
230321_AKo_Calibration_16min-MWi_01	Standard	RvE2	0,33	N/A	N/A	N/A	N/A	N/A	N/A	N/A	N/A	☑
230321_AKo_Calibration_16min-MWi_02	Standard	RvE2	1,00	0,5	48	3,3	7,78	0,83	0,05	27	2848	☑
230321_AKo_Calibration_16min-MWi_03	Standard	RvE2	1,00	0,9	87	4,3	7,77	0,83	0,04	30	4236	☑
230321_AKo_Calibration_16min-MWi_04	Standard	RvE2	1,00	0,4	36	1,5	7,77	0,83	0,07	23	2788	☑
230321_AKo_Calibration_16min-MWi_05	Standard	RvE2	1,00	0,7	73	4,7	7,76	0,83	0,06	30	3770	☑
230321_AKo_Calibration_16min-MWi_06	Standard	RvE2	1,00	0,7	74	3,7	7,78	0,83	0,05	27	3240	☑
230321_AKo_Calibration_16min-MWi_07	Standard	RvE2	1,00	0,8	81	5,0	7,76	0,83	0,05	28	3910	☑
230321_AKo_Calibration_16min-MWi_08	Standard	RvE2	1,00	0,5	51	3,8	7,77	0,83	0,04	25	2914	☑
230321_AKo_Calibration_16min-MWi_09	Standard	RvE2	3,33	3,7	111	11,8	7,77	0,83	0,07	31	10379	☑
230321_AKo_Calibration_16min-MWi_10	Standard	RvE2	3,33	3,0	89	7,9	7,76	0,83	0,06	27	10749	☑
230321_AKo_Calibration_16min-MWi_11	Standard	RvE2	3,33	3,8	115	9,4	7,77	0,83	0,04	31	11422	☑
230321_AKo_Calibration_16min-MWi_12	Standard	RvE2	3,33	4,6	139	12,7	7,77	0,83	0,04	27	13001	☑
230321_AKo_Calibration_16min-MWi_13	Standard	RvE2	3,33	3,4	101	6,9	7,77	0,83	0,05	33	11652	☑
230321_AKo_Calibration_16min-MWi_14	Standard	RvE2	3,33	4,5	136	6,3	7,77	0,83	0,05	28	8861	☑
230321_AKo_Calibration_16min-MWi_15	Standard	RvE2	3,33	3,0	91	7,6	7,77	0,83	0,06	34	10077	☑
230321_AKo_Calibration_16min-MWi_16	Standard	RvE2	10,00	11,1	111	16,9	7,76	0,83	0,06	26	27503	☑
230321_AKo_Calibration_16min-MWi_17	Standard	RvE2	10,00	12,5	125	18,9	7,77	0,83	0,06	26	34085	☑
230321_AKo_Calibration_16min-MWi_18	Standard	RvE2	10,00	11,9	119	20,1	7,76	0,83	0,06	29	34505	☑
230321_AKo_Calibration_16min-MWi_19	Standard	RvE2	10,00	10,9	109	26,8	7,76	0,83	0,05	25	33527	☑
230321_AKo_Calibration_16min-MWi_20	Standard	RvE2	10,00	9,4	94	20,0	7,76	0,83	0,06	28	34791	☑
230321_AKo_Calibration_16min-MWi_21	Standard	RvE2	10,00	13,4	134	28,0	7,76	0,83	0,04	25	36362	☑
230321_AKo_Calibration_16min-MWi_22	Standard	RvE2	10,00	9,8	98	15,7	7,76	0,83	0,07	28	34932	☑
230321_AKo_Calibration_16min-MWi_23	Standard	RvE2	33,33	40,7	122	82,3	7,76	0,83	0,05	29	126184	☑
230321_AKo_Calibration_16min-MWi_24	Standard	RvE2	33,33	47,8	143	38,6	7,77	0,83	0,06	29	107050	☑
230321_AKo_Calibration_16min-MWi_25	Standard	RvE2	100,00	105,3	105	138,0	7,77	0,83	0,06	28	300642	☑
230321_AKo_Calibration_16min-MWi_26	Standard	RvE2	100,00	101,2	101	193,0	7,77	0,83	0,05	31	291559	☑
230321_AKo_Calibration_16min-MWi_27	Standard	RvE2	333,33	326,9	98	401,4	7,77	0,83	0,05	28	881235	☑
230321_AKo_Calibration_16min-MWi_28	Standard	RvE2	333,33	317,3	95	207,8	7,76	0,83	0,06	30	935820	☑
230321_AKo_Calibration_16min-MWi_29	Standard	RvE2	333,33	307,8	92	222,5	7,77	0,83	0,06	34	824374	☑
230321_AKo_Calibration_16min-MWi_30	Standard	RvE2	333,33	386,5	116	322,4	7,77	0,83	0,05	36	1024497	☑
230321_AKo_Calibration_16min-MWi_31	Standard	RvE2	333,33	363,4	109	460,9	7,77	0,83	0,05	34	1076312	☑
230321_AKo_Calibration_16min-MWi_32	Standard	RvE2	333,33	343,9	103	320,2	7,77	0,83	0,06	32	811257	☑
230321_AKo_Calibration_16min-MWi_33	Standard	RvE2	333,33	339,2	102	320,2	7,77	0,83	0,06	28	867346	☑
230321_AKo_Calibration_16min-MWi_34	Standard	RvE2	500,00	540,5	108	277,6	7,77	0,83	0,06	22	1541325	☑
230321_AKo_Calibration_16min-MWi_35	Standard	RvE2	500,00	485,6	97	239,2	7,77	0,83	0,05	25	1193641	☑
230321_AKo_Calibration_16min-MWi_36	Standard	RvE2	500,00	501,0	100	340,3	7,76	0,83	0,06	33	1351802	☑
230321_AKo_Calibration_16min-MWi_37	Standard	RvE2	500,00	508,9	102	280,0	7,76	0,83	0,06	28	1217680	☑
230321_AKo_Calibration_16min-MWi_38	Standard	RvE2	500,00	484,6	97	220,8	7,76	0,83	0,06	33	1366348	☑
230321_AKo_Calibration_16min-MWi_39	Standard	RvE2	500,00	476,9	95	326,6	7,76	0,83	0,05	25	1282696	☑
230321_AKo_Calibration_16min-MWi_40	Standard	RvE2	500,00	483,4	97	533,4	7,76	0,83	0,06	25	1273171	☑
230321_AKo_Calibration_16min-MWi_41	Standard	RvE2	1000,00	923,2	92	684,9	7,76	0,83	0,05	32	2406475	☑
230321_AKo_Calibration_16min-MWi_42	Standard	RvE2	1000,00	1050,4	105	493,4	7,76	0,83	0,06	31	2699654	☑
230321_AKo_Calibration_16min-MWi_43	Standard	RvE2	2000,00	1988,2	99	391,6	7,76	0,83	0,06	32	5905957	☑
230321_AKo_Calibration_16min-MWi_44	Standard	RvE2	2000,00	1968,1	98	581,2	7,76	0,83	0,06	26	5768969	☑
230321_AKo_Calibration_16min-MWi_45	Unknown	RvE2	N/A	< 0	N/A	2,1	7,77	0,83	0,04	14	1451	☑
Blank	Double Blank	RvE2	N/A	N/A	N/A	N/A	N/A	N/A	N/A	N/A	N/A	☑
Blank	Double Blank	RvE2	N/A	N/A	N/A	N/A	N/A	N/A	N/A	N/A	N/A	☑
Blank	Double Blank	RvE2	N/A	N/A	N/A	N/A	N/A	N/A	N/A	N/A	N/A	☑
Blank	Double Blank	RvE2	N/A	N/A	N/A	N/A	N/A	N/A	N/A	N/A	N/A	☑
Blank	Double Blank	RvE2	N/A	N/A	N/A	N/A	N/A	N/A	N/A	N/A	N/A	☑

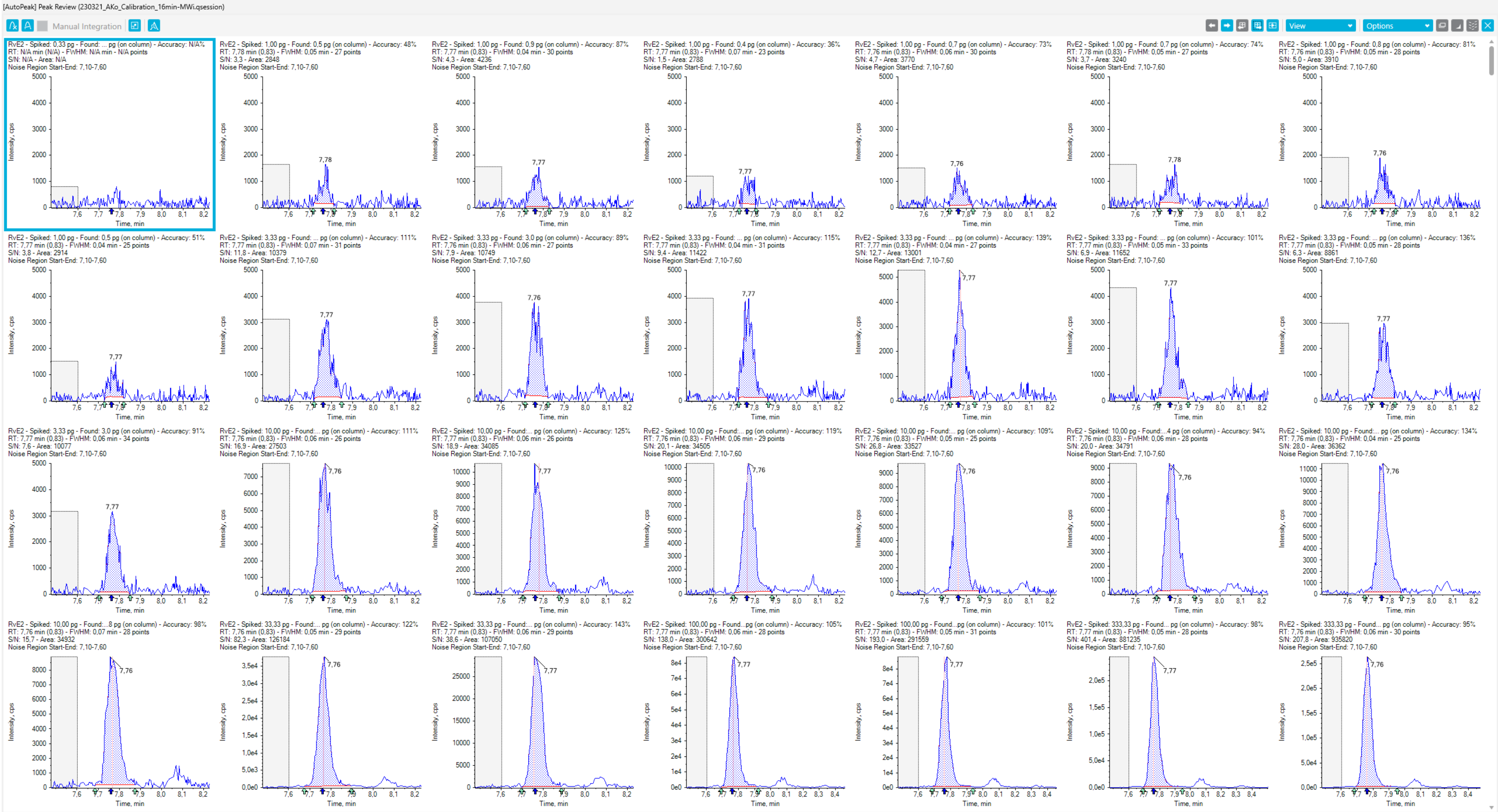
[AutoPeak] Peak Review (230321_AKo_Calibration_16min-MWi.qsession)
Manual Integration
View
Options

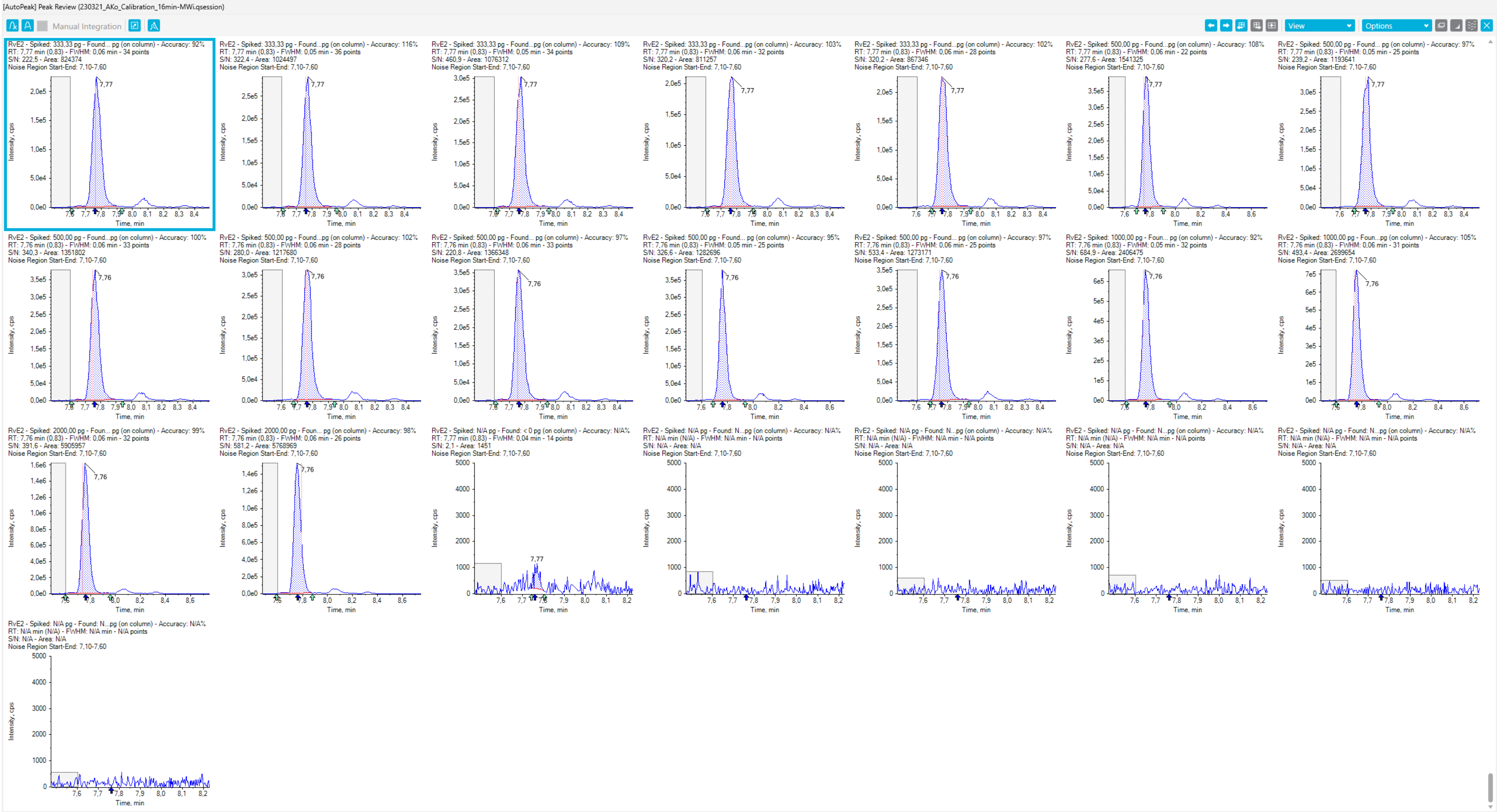
[AutoPeak] Peak Review (230321_AKo_Calibration_16min-MWi.qsession)
Manual Integration
View
Options
RvE2 - Spiked: 333,33 pg - Found... pg (on column) - Accuracy: 92%
RT: 7,77 min (0,83) - FWHM: 0,06 min - 34 points
S/N: 222,5 - Area: 824374
Noise Region Start-End: 7,10-7,60
RvE2 - Spiked: 333,33 pg - Found...pg (on column) - Accuracy: 116%
RT: 7,77 min (0,83) - FWHM: 0,05 min - 36 points
S/N: 322,4 - Area: 1024497
Noise Region Start-End: 7,10-7,60
RvE2 - Spiked: 333,33 pg - Found...pg (on column) - Accuracy: 109%
RT: 7,77 min (0,83) - FWHM: 0,05 min - 34 points
S/N: 460,9 - Area: 1076312
Noise Region Start-End: 7,10-7,60
RvE2 - Spiked: 333,33 pg - Found...pg (on column) - Accuracy: 103%
RT: 7,77 min (0,83) - FWHM: 0,06 min - 32 points
S/N: 320,2 - Area: 811257
Noise Region Start-End: 7,10-7,60
RvE2 - Spiked: 333,33 pg - Found...pg (on column) - Accuracy: 102%
RT: 7,77 min (0,83) - FWHM: 0,06 min - 28 points
S/N: 320,2 - Area: 867346
Noise Region Start-End: 7,10-7,60
RvE2 - Spiked: 500,00 pg - Found...pg (on column) - Accuracy: 108%
RT: 7,77 min (0,83) - FWHM: 0,06 min - 22 points
S/N: 277,6 - Area: 1541325
Noise Region Start-End: 7,10-7,60
RvE2 - Spiked: 500,00 pg - Found... pg (on column) - Accuracy: 97%
RT: 7,77 min (0,83) - FWHM: 0,05 min - 25 points
S/N: 239,2 - Area: 1193641
Noise Region Start-End: 7,10-7,60
RvE2 - Spiked: 500,00 pg - Found...pg (on column) - Accuracy: 100%
RT: 7,76 min (0,83) - FWHM: 0,06 min - 33 points
S/N: 340,3 - Area: 1351802
Noise Region Start-End: 7,10-7,60
RvE2 - Spiked: 500,00 pg - Found...pg (on column) - Accuracy: 102%
RT: 7,76 min (0,83) - FWHM: 0,06 min - 28 points
S/N: 280,0 - Area: 1217680
Noise Region Start-End: 7,10-7,60
RvE2 - Spiked: 500,00 pg - Found... pg (on column) - Accuracy: 97%
RT: 7,76 min (0,83) - FWHM: 0,06 min - 33 points
S/N: 220,8 - Area: 1366348
Noise Region Start-End: 7,10-7,60
RvE2 - Spiked: 500,00 pg - Found... pg (on column) - Accuracy: 95%
RT: 7,76 min (0,83) - FWHM: 0,05 min - 25 points
S/N: 326,6 - Area: 1282696
Noise Region Start-End: 7,10-7,60
RvE2 - Spiked: 500,00 pg - Found... pg (on column) - Accuracy: 97%
RT: 7,76 min (0,83) - FWHM: 0,06 min - 25 points
S/N: 533,4 - Area: 1273171
Noise Region Start-End: 7,10-7,60
RvE2 - Spiked: 1000,00 pg - Foun... pg (on column) - Accuracy: 92%
RT: 7,76 min (0,83) - FWHM: 0,05 min - 32 points
S/N: 684,9 - Area: 2406475
Noise Region Start-End: 7,10-7,60
RvE2 - Spiked: 1000,00 pg - Foun...pg (on column) - Accuracy: 105%
RT: 7,76 min (0,83) - FWHM: 0,06 min - 31 points
S/N: 493,4 - Area: 2699654
Noise Region Start-End: 7,10-7,60
RvE2 - Spiked: 2000,00 pg - Foun... pg (on column) - Accuracy: 99%
RT: 7,76 min (0,83) - FWHM: 0,06 min - 32 points
S/N: 391,6 - Area: 5905957
Noise Region Start-End: 7,10-7,60
RvE2 - Spiked: 2000,00 pg - Foun... pg (on column) - Accuracy: 98%
RT: 7,76 min (0,83) - FWHM: 0,06 min - 26 points
S/N: 581,2 - Area: 5768969
Noise Region Start-End: 7,10-7,60
RvE2 - Spiked: N/A pg - Found: < 0 pg (on column) - Accuracy: N/A%
RT: 7,77 min (0,83) - FWHM: 0,04 min - 14 points
S/N: 2,1 - Area: 1451
Noise Region Start-End: 7,10-7,60
RvE2 - Spiked: N/A pg - Found: N...pg (on column) - Accuracy: N/A%
RT: N/A min (N/A) - FWHM: N/A min - N/A points
S/N: N/A - Area: N/A
Noise Region Start-End: 7,10-7,60
RvE2 - Spiked: N/A pg - Found: N...pg (on column) - Accuracy: N/A%
RT: N/A min (N/A) - FWHM: N/A min - N/A points
S/N: N/A - Area: N/A
Noise Region Start-End: 7,10-7,60
RvE2 - Spiked: N/A pg - Found: N...pg (on column) - Accuracy: N/A%
RT: N/A min (N/A) - FWHM: N/A min - N/A points
S/N: N/A - Area: N/A
Noise Region Start-End: 7,10-7,60
RvE2 - Spiked: N/A pg - Found: N...pg (on column) - Accuracy: N/A%
RT: N/A min (N/A) - FWHM: N/A min - N/A points
S/N: N/A - Area: N/A
Noise Region Start-End: 7,10-7,60
RvE2 - Spiked: N/A pg - Found: N...pg (on column) - Accuracy: N/A%
RT: N/A min (N/A) - FWHM: N/A min - N/A points
S/N: N/A - Area: N/A
Noise Region Start-End: 7,10-7,60

RvE4

[AutoPeak] Results Table (230321_AKo_Calibration_16min-MWi.qsession)

50 rows Filters: 0

Sample Name	Sample T...	Component Name	Actual Concentr...	Calculated Concentrat...	Accuracy	Signal / Noise	Retent... Time	Relativ...	Width at 50%	Points Across...	Area	U...
230321_AKo_Calibration_16min-MWi_01	Standard	RvE4	0,33	< 0	N/A	3,2	8,06	0,86	0,06	41	7766	☑
230321_AKo_Calibration_16min-MWi_02	Standard	RvE4	1,00	0,1	13	8,1	8,06	0,86	0,06	48	24792	☑
230321_AKo_Calibration_16min-MWi_03	Standard	RvE4	1,00	0,2	21	11,6	8,08	0,86	0,06	48	29004	☑
230321_AKo_Calibration_16min-MWi_04	Standard	RvE4	1,00	0,3	33	11,5	8,07	0,86	0,05	50	30973	☑
230321_AKo_Calibration_16min-MWi_05	Standard	RvE4	1,00	0,3	32	9,7	8,07	0,86	0,06	58	30519	☑
230321_AKo_Calibration_16min-MWi_06	Standard	RvE4	1,00	0,6	62	11,1	8,07	0,86	0,05	62	30694	☑
230321_AKo_Calibration_16min-MWi_07	Standard	RvE4	1,00	0,2	21	11,9	8,07	0,86	0,06	42	27818	☑
230321_AKo_Calibration_16min-MWi_08	Standard	RvE4	1,00	0,4	39	12,5	8,07	0,86	0,05	50	29061	☑
230321_AKo_Calibration_16min-MWi_09	Standard	RvE4	3,33	4,1	123	35,9	8,07	0,86	0,05	55	87213	☑
230321_AKo_Calibration_16min-MWi_10	Standard	RvE4	3,33	3,5	105	37,7	8,06	0,86	0,04	44	96476	☑
230321_AKo_Calibration_16min-MWi_11	Standard	RvE4	3,33	4,3	128	46,2	8,07	0,86	0,05	49	96032	☑
230321_AKo_Calibration_16min-MWi_12	Standard	RvE4	3,33	4,6	138	37,1	8,07	0,86	0,05	51	97993	☑
230321_AKo_Calibration_16min-MWi_13	Standard	RvE4	3,33	3,9	116	25,1	8,07	0,86	0,06	61	100705	☑
230321_AKo_Calibration_16min-MWi_14	Standard	RvE4	3,33	5,1	152	28,8	8,07	0,86	0,05	54	73576	☑
230321_AKo_Calibration_16min-MWi_15	Standard	RvE4	3,33	3,6	107	24,3	8,06	0,86	0,06	55	90337	☑
230321_AKo_Calibration_16min-MWi_16	Standard	RvE4	10,00	14,6	146	86,6	8,07	0,86	0,06	45	249449	☑
230321_AKo_Calibration_16min-MWi_17	Standard	RvE4	10,00	15,8	158	99,3	8,07	0,86	0,06	51	298066	☑
230321_AKo_Calibration_16min-MWi_18	Standard	RvE4	10,00	14,4	144	99,3	8,07	0,86	0,06	47	291060	☑
230321_AKo_Calibration_16min-MWi_19	Standard	RvE4	10,00	14,9	149	100,3	8,07	0,86	0,06	44	314635	☑
230321_AKo_Calibration_16min-MWi_20	Standard	RvE4	10,00	13,4	134	139,9	8,07	0,86	0,06	49	342201	☑
230321_AKo_Calibration_16min-MWi_21	Standard	RvE4	10,00	14,4	144	93,7	8,07	0,86	0,06	45	272876	☑
230321_AKo_Calibration_16min-MWi_22	Standard	RvE4	10,00	12,4	124	121,2	8,07	0,86	0,06	52	309197	☑
230321_AKo_Calibration_16min-MWi_23	Standard	RvE4	33,33	52,3	157	155,2	8,07	0,86	0,06	34	1096552	☑
230321_AKo_Calibration_16min-MWi_24	Standard	RvE4	33,33	57,1	171	225,0	8,07	0,86	0,06	34	864489	☑
230321_AKo_Calibration_16min-MWi_25	Standard	RvE4	100,00	137,2	137	376,6	8,07	0,86	0,06	32	2634588	☑
230321_AKo_Calibration_16min-MWi_26	Standard	RvE4	100,00	135,0	135	366,8	8,07	0,86	0,06	38	2614975	☑
230321_AKo_Calibration_16min-MWi_27	Standard	RvE4	333,33	388,9	117	527,3	8,07	0,86	0,06	34	7034586	☑
230321_AKo_Calibration_16min-MWi_28	Standard	RvE4	333,33	368,7	111	235,0	8,07	0,86	0,06	31	7296794	☑
230321_AKo_Calibration_16min-MWi_29	Standard	RvE4	333,33	360,8	108	425,7	8,07	0,86	0,06	34	6484608	☑
230321_AKo_Calibration_16min-MWi_30	Standard	RvE4	333,33	437,0	131	529,5	8,07	0,86	0,05	34	7771382	☑
230321_AKo_Calibration_16min-MWi_31	Standard	RvE4	333,33	403,4	121	438,7	8,07	0,86	0,07	35	8017368	☑
230321_AKo_Calibration_16min-MWi_32	Standard	RvE4	333,33	413,1	124	285,0	8,07	0,86	0,06	33	6539444	☑
230321_AKo_Calibration_16min-MWi_33	Standard	RvE4	333,33	413,0	124	450,8	8,07	0,86	0,06	32	7084137	☑
230321_AKo_Calibration_16min-MWi_34	Standard	RvE4	500,00	603,2	121	491,2	8,07	0,86	0,06	34	11537487	☑
230321_AKo_Calibration_16min-MWi_35	Standard	RvE4	500,00	552,2	110	505,5	8,07	0,86	0,07	34	9105253	☑
230321_AKo_Calibration_16min-MWi_36	Standard	RvE4	500,00	544,9	109	222,0	8,06	0,86	0,07	34	9863557	☑
230321_AKo_Calibration_16min-MWi_37	Standard	RvE4	500,00	569,3	114	245,6	8,07	0,86	0,06	34	9136817	☑
230321_AKo_Calibration_16min-MWi_38	Standard	RvE4	500,00	552,6	111	485,7	8,06	0,86	0,06	35	10450371	☑
230321_AKo_Calibration_16min-MWi_39	Standard	RvE4	500,00	550,2	110	167,4	8,06	0,86	0,06	35	9926483	☑
230321_AKo_Calibration_16min-MWi_40	Standard	RvE4	500,00	538,8	108	213,0	8,06	0,86	0,06	34	9518943	☑
230321_AKo_Calibration_16min-MWi_41	Standard	RvE4	1000,00	920,3	92	318,0	8,06	0,86	0,08	31	16085070	☑
230321_AKo_Calibration_16min-MWi_42	Standard	RvE4	1000,00	988,6	99	291,0	8,06	0,86	0,07	33	17036894	☑
230321_AKo_Calibration_16min-MWi_43	Standard	RvE4	2000,00	1510,7	76	68,0	8,06	0,86	0,08	39	30084419	☑
230321_AKo_Calibration_16min-MWi_44	Standard	RvE4	2000,00	1573,3	79	57,8	8,06	0,86	0,08	29	30917672	☑
230321_AKo_Calibration_16min-MWi_45	Unknown	RvE4	N/A	0,4	N/A	6,2	8,06	0,86	0,09	62	27883	☑
Blank	Double Blank	RvE4	N/A	N/A	N/A	N/A	N/A	N/A	N/A	N/A	N/A	☑
Blank	Double Blank	RvE4	N/A	N/A	N/A	N/A	N/A	N/A	N/A	N/A	N/A	☑
Blank	Double Blank	RvE4	N/A	N/A	N/A	N/A	N/A	N/A	N/A	N/A	N/A	☑
Blank	Double Blank	RvE4	N/A	N/A	N/A	N/A	N/A	N/A	N/A	N/A	N/A	☑
Blank	Double Blank	RvE4	N/A	N/A	N/A	N/A	N/A	N/A	N/A	N/A	N/A	☑

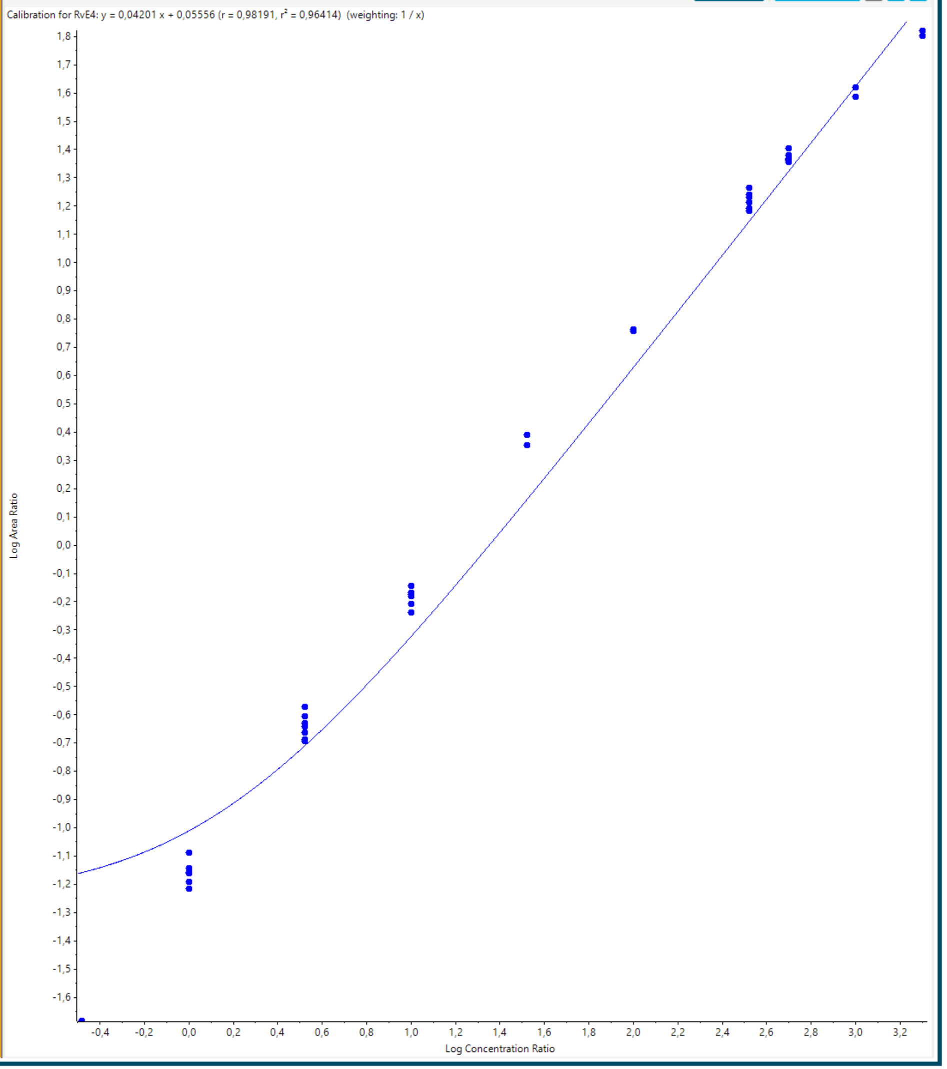

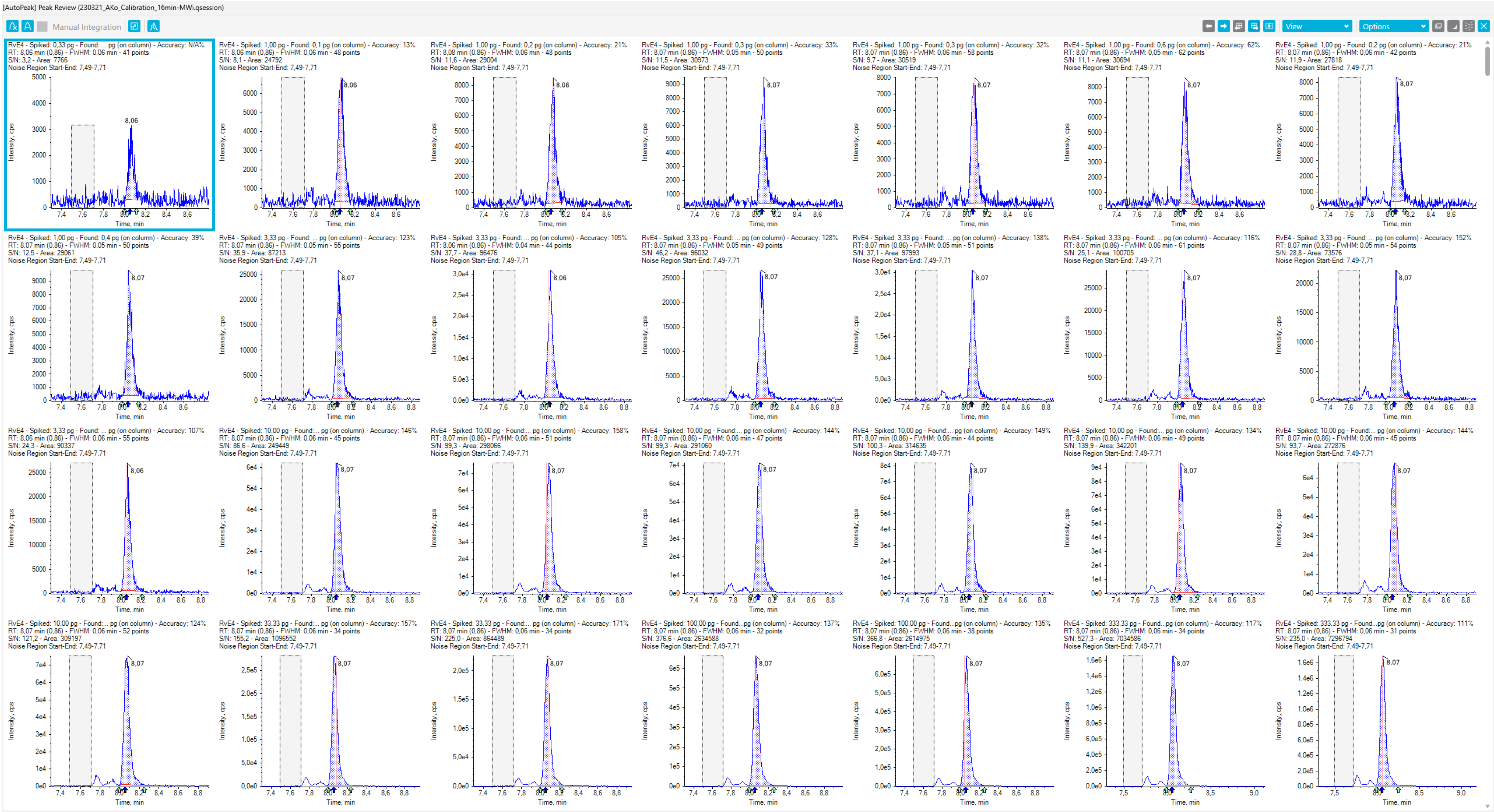
[AutoPeak] Peak Review (230321_AKo_Calibration_16min-MWi.qsession)
Manual Integration
View
Options

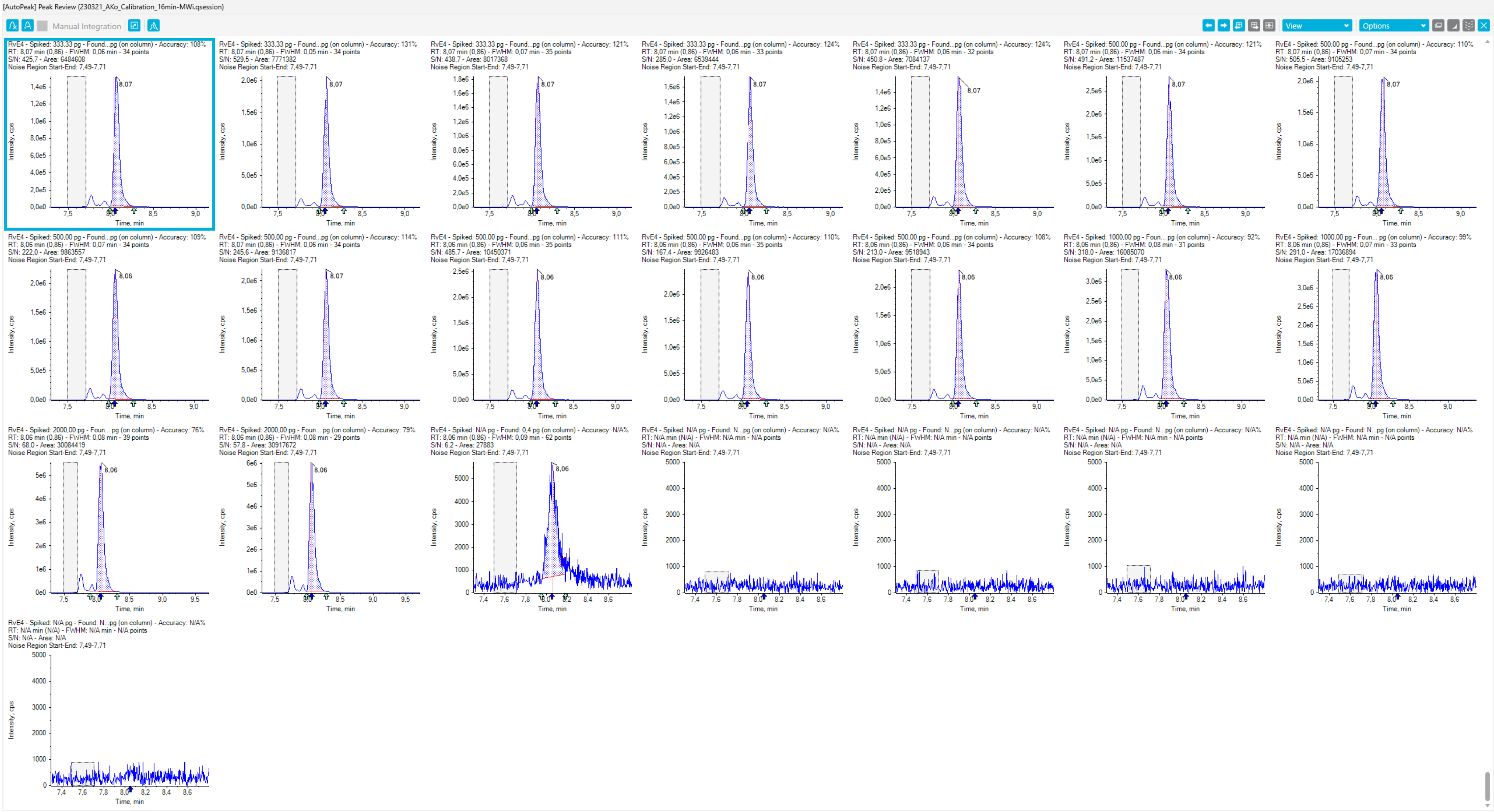
[AutoPeak] Peak Review (230321_AKo_Calibration_16min-MWi.qsession)
Manual Integration
View
Options

Fig. S5: Comparison of signal-to-noise calculation methods

Signal-to-noise (S/N) ratios obtained by different calculation algorithms using the example of LXA_4.
A) Summary of S/N comparison at different concentrations.

Representative chromatograms (1 pg LXA_4 on column) of the same sample using different S/N calculation algorithms.
B) Manual calculation.
C) Automatic calculation by the **peak-to-peak** algorithm.
D) Automatic calculation by the **SD** algorithm.
E) Automatic calculation by the **relative noise** algorithm.

Application of different calculation algorithms to biological samples.
M2-MDM (2×10^6 cells/mL) were stimulated with SACM (1%) for 90 min as described in the main text.
Chromatograms of the same sample are shown (calculated content of 133 pg LXA_4 on column).
F) Manual calculation based on the front noise region.
G) Automatic calculation by the **peak-to-peak** algorithm.
H) Automatic calculation by the **SD** algorithm.
I) Automatic calculation by the **relative noise** algorithm.

A)

Summary of S/N comparison at different concentrations. The reference standard LXA_4 was spiked at do surrogate matrix (PBS) at different concentrations, extracted by SPE, and analyzed by UHPLC-MS/MS (n = 3). The S/N ratios were calculated manually or using the indicated software-based algorithms (Sciex OS 4.0). Comparable results were obtained by manual calculation (independent of the noise region selection) and the peak-to-peak algorithm. SD yielded higher results that did not match visual estimation. Relative noise yielded intermediate results with the benefit of not requiring a user-specified noise region.

Calculation method	Noise region		1 pg on column			3.33 pg on column			10 pg on column			333.33 pg on column			500 pg on column		
	Selection	Location[a]	Mean		SD	Mean		SD	Mean		SD	Mean		SD	Mean		SD
Manually	By user	Tail	8	±	4	17	±	3	56	±	12	624	±	201	850	±	255
	By user	Front	7	±	2	21	±	6	68	±	31	527	±	192	796	±	351
Peak-to-peak	By user	Front	7	±	2	23	±	11	59	±	31	499	±	205	569	±	192
SD	By user	Front	24	±	5	69	±	22	184	±	74	1,314	±	230	1,560	±	445
Relative noise	None	Center	14	±	5	36	±	17	77	±	33	798	±	290	984	±	349

[a]Relative to target peak (in the same chromatogram)

B) 1 pg LXA_4 standard on column

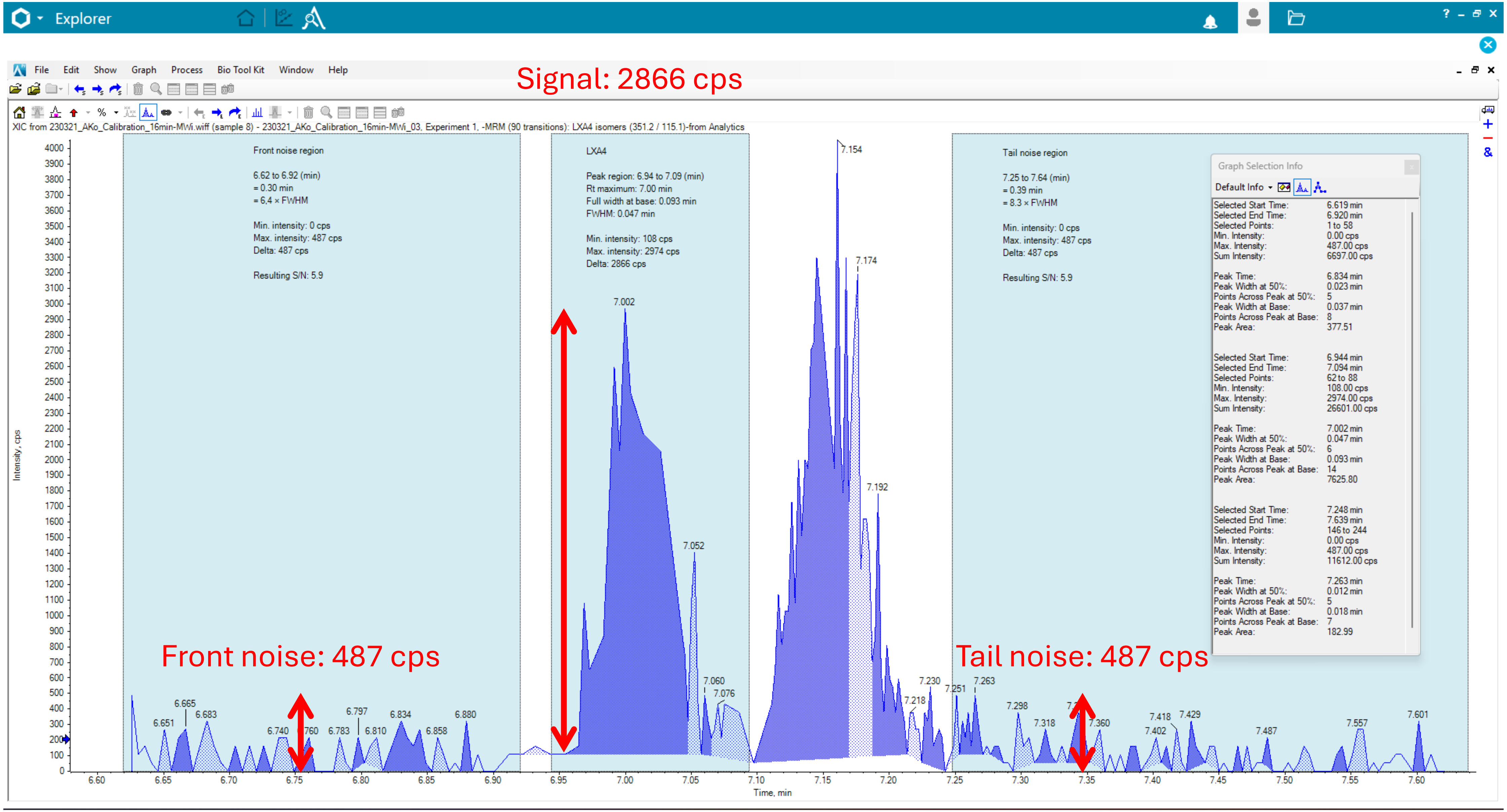

S/N_{front} = 5.9

S/N_{tail} = 5.9

C) 1 pg LXA_4 standard on column

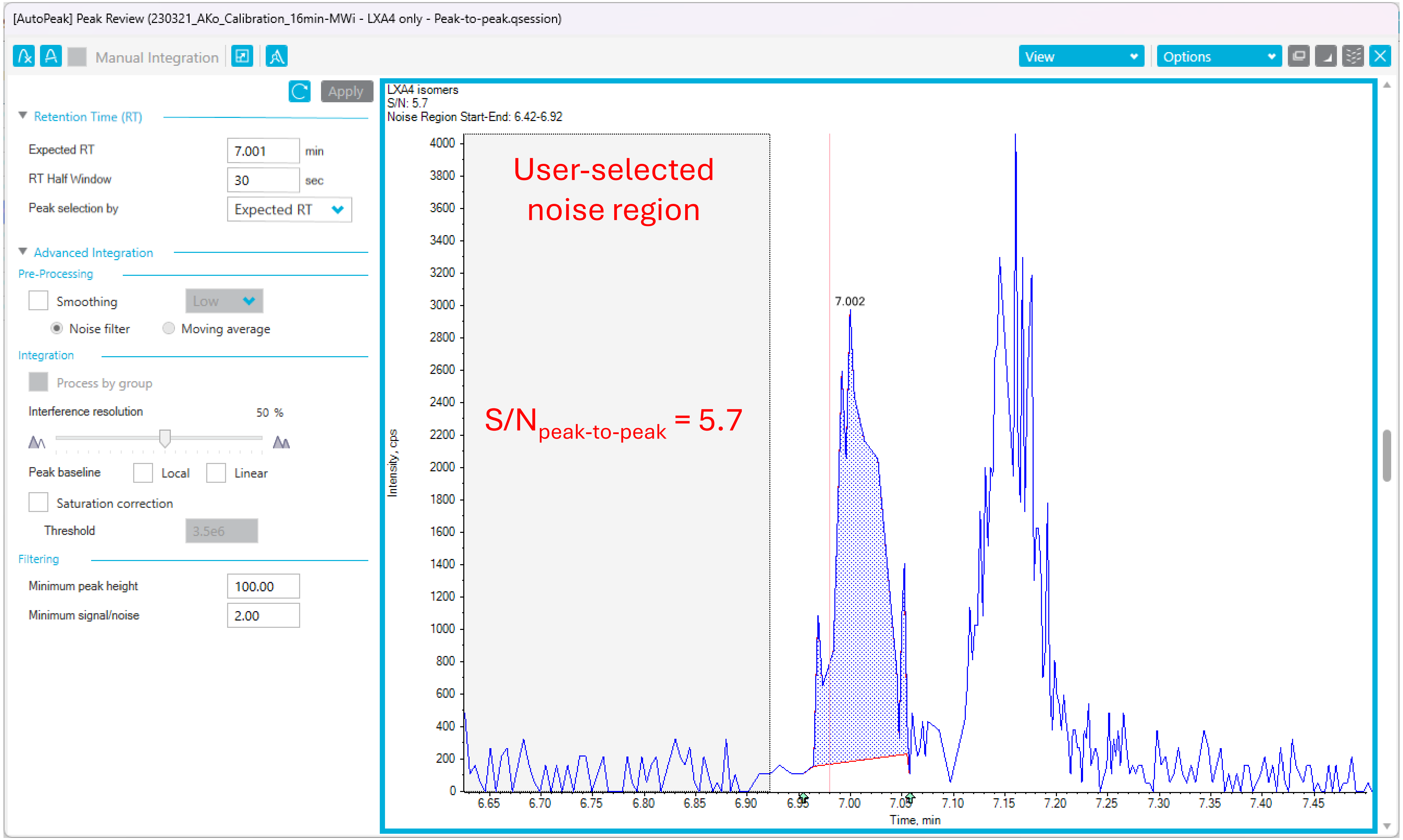

D)

1 pg LXA_4 standard on column

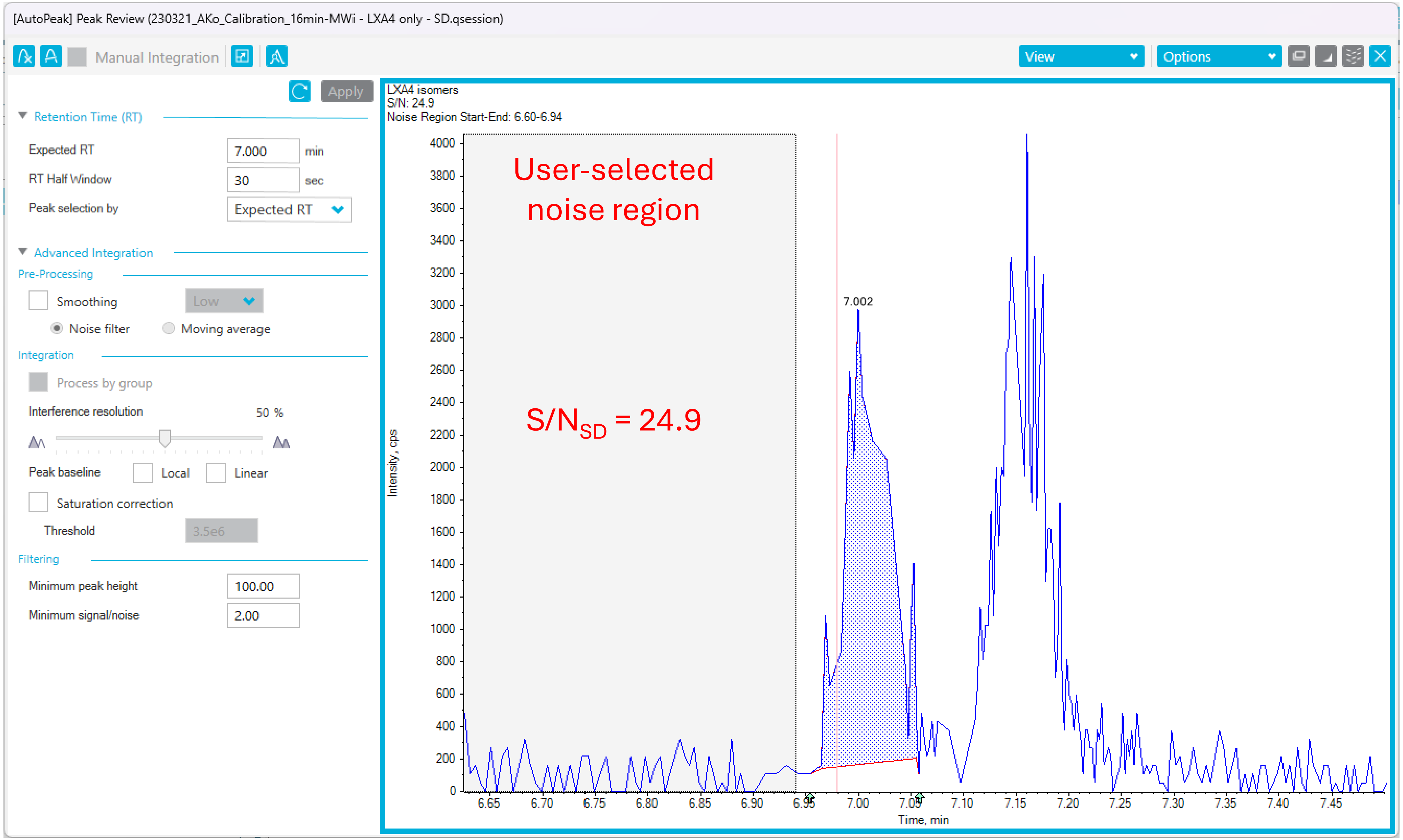

E)

1 pg LXA_4 standard on column

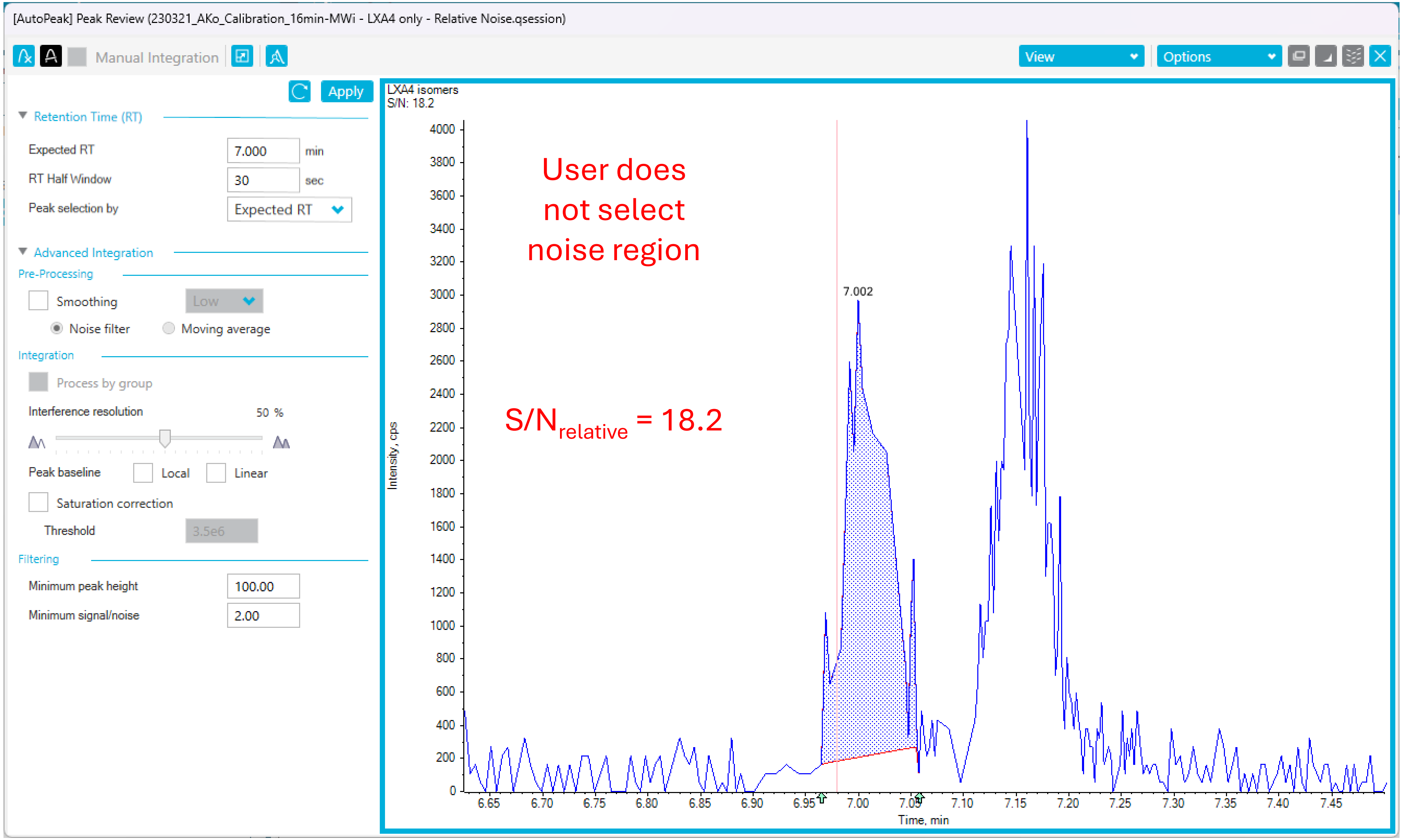

F) Biological sample (133 pg LXA_4 on column)

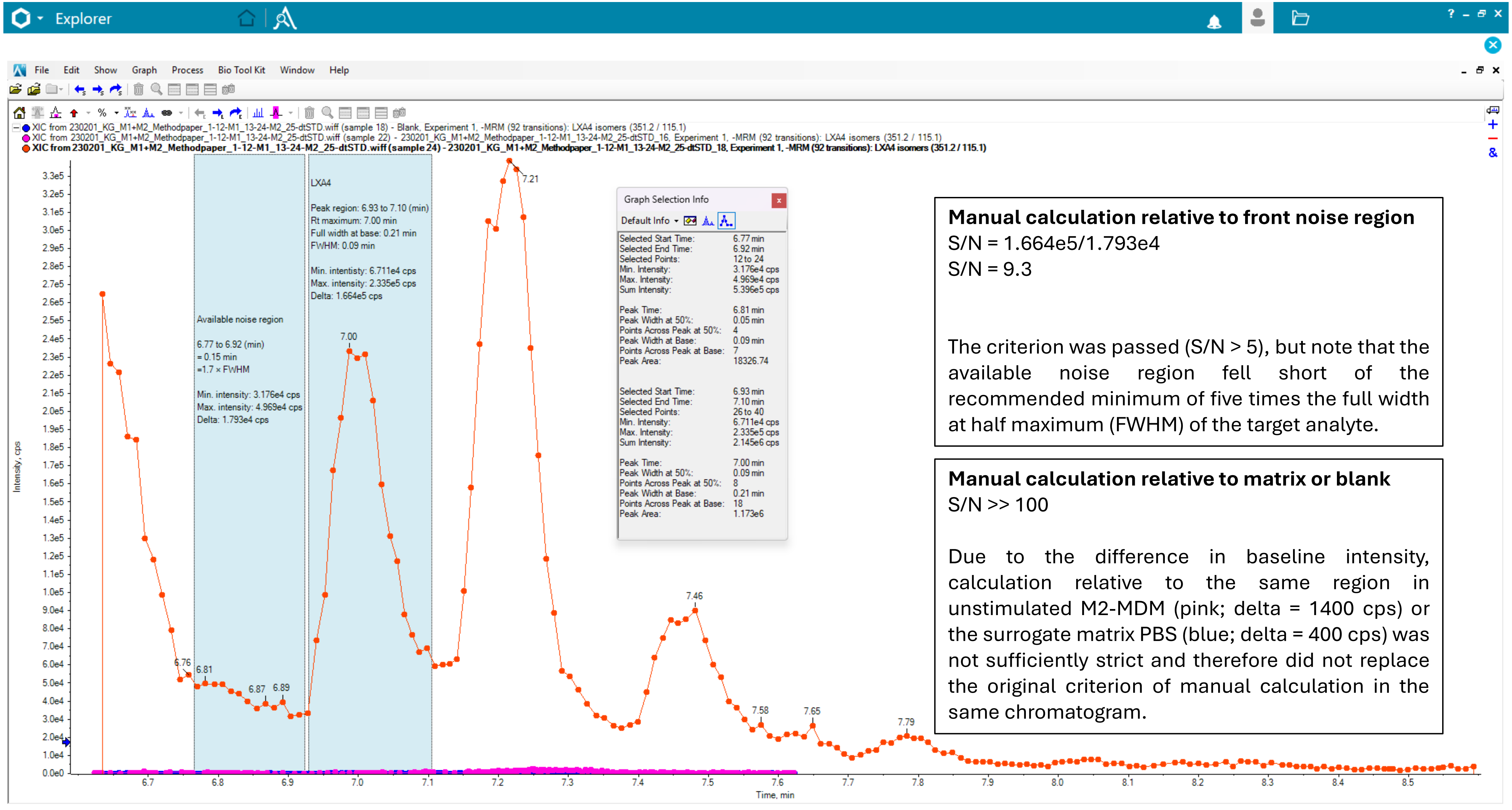

Manual calculation relative to front noise region

S/N = 1.664e5/1.793e4

S/N = 9.3

The criterion was passed (S/N > 5), but note that the available noise region fell short of the recommended minimum of five times the full width at half maximum (FWHM) of the target analyte.

Manual calculation relative to matrix or blank

S/N >> 100

Due to the difference in baseline intensity, calculation relative to the same region in unstimulated M2-MDM (pink; delta = 1400 cps) or the surrogate matrix PBS (blue; delta = 400 cps) was not sufficiently strict and therefore did not replace the original criterion of manual calculation in the same chromatogram.

G) Biological sample (133 pg LXA_4 on column)

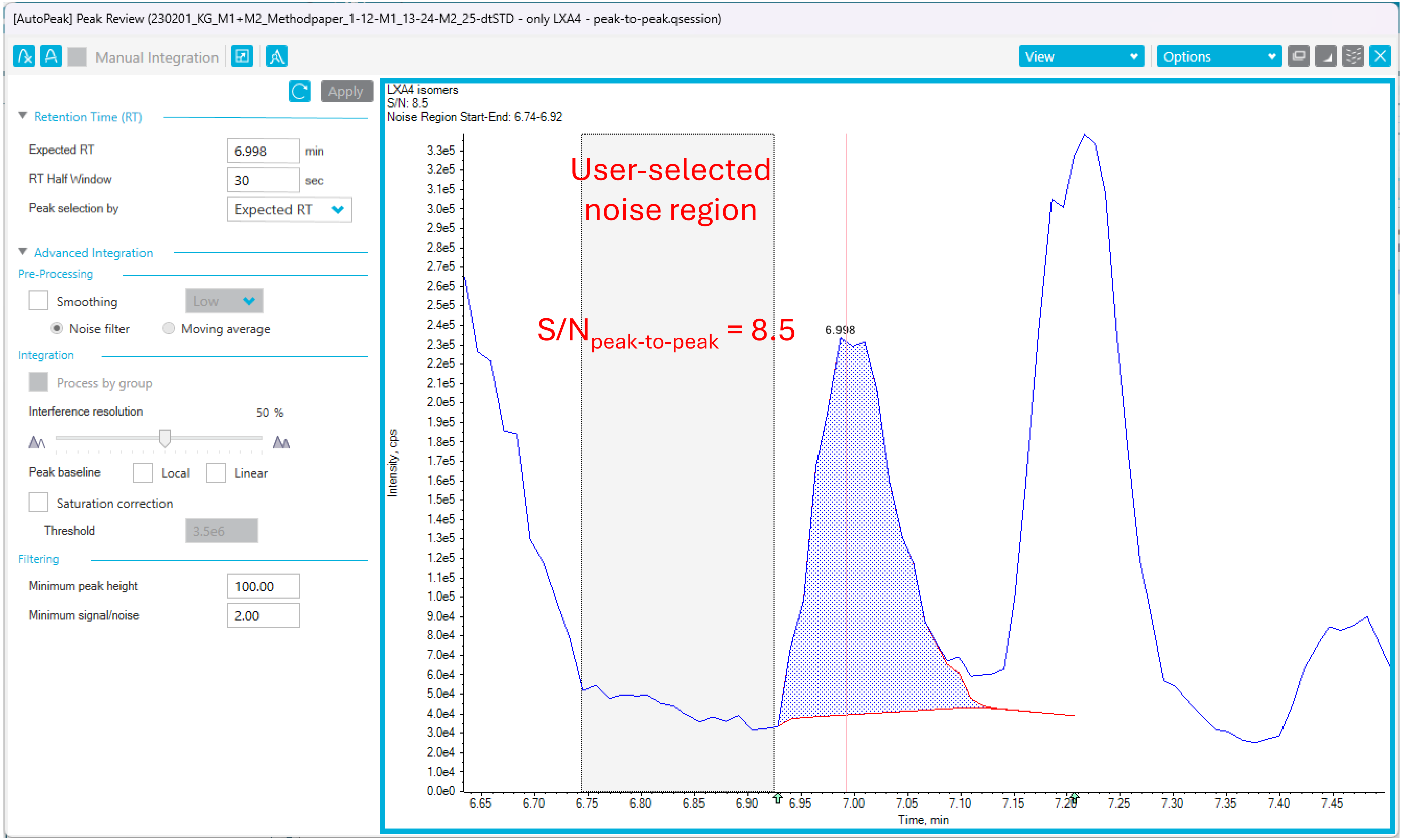

H) Biological sample (133 pg LXA_4 on column)

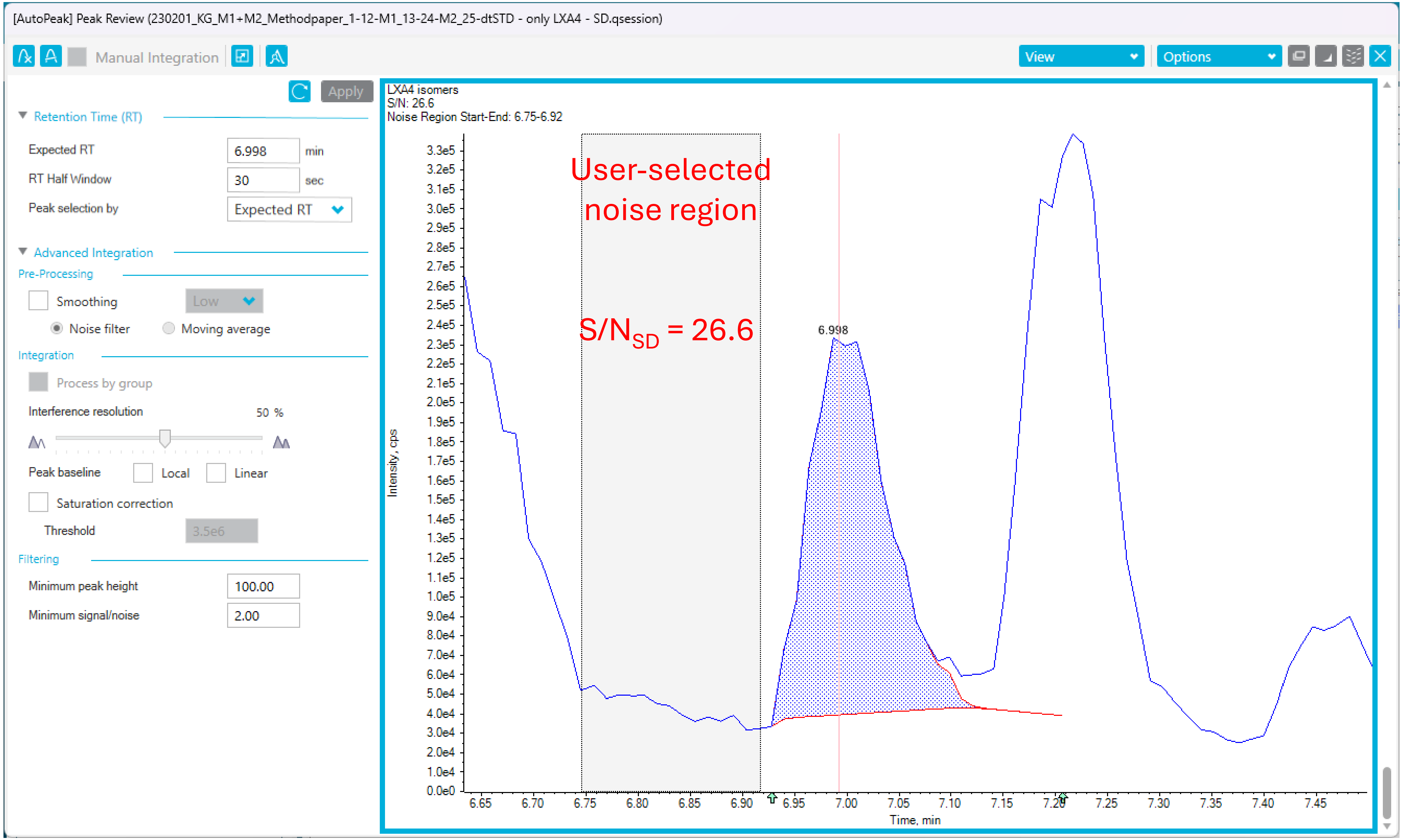

I)

Biological sample (133 pg LXA_4 on column)

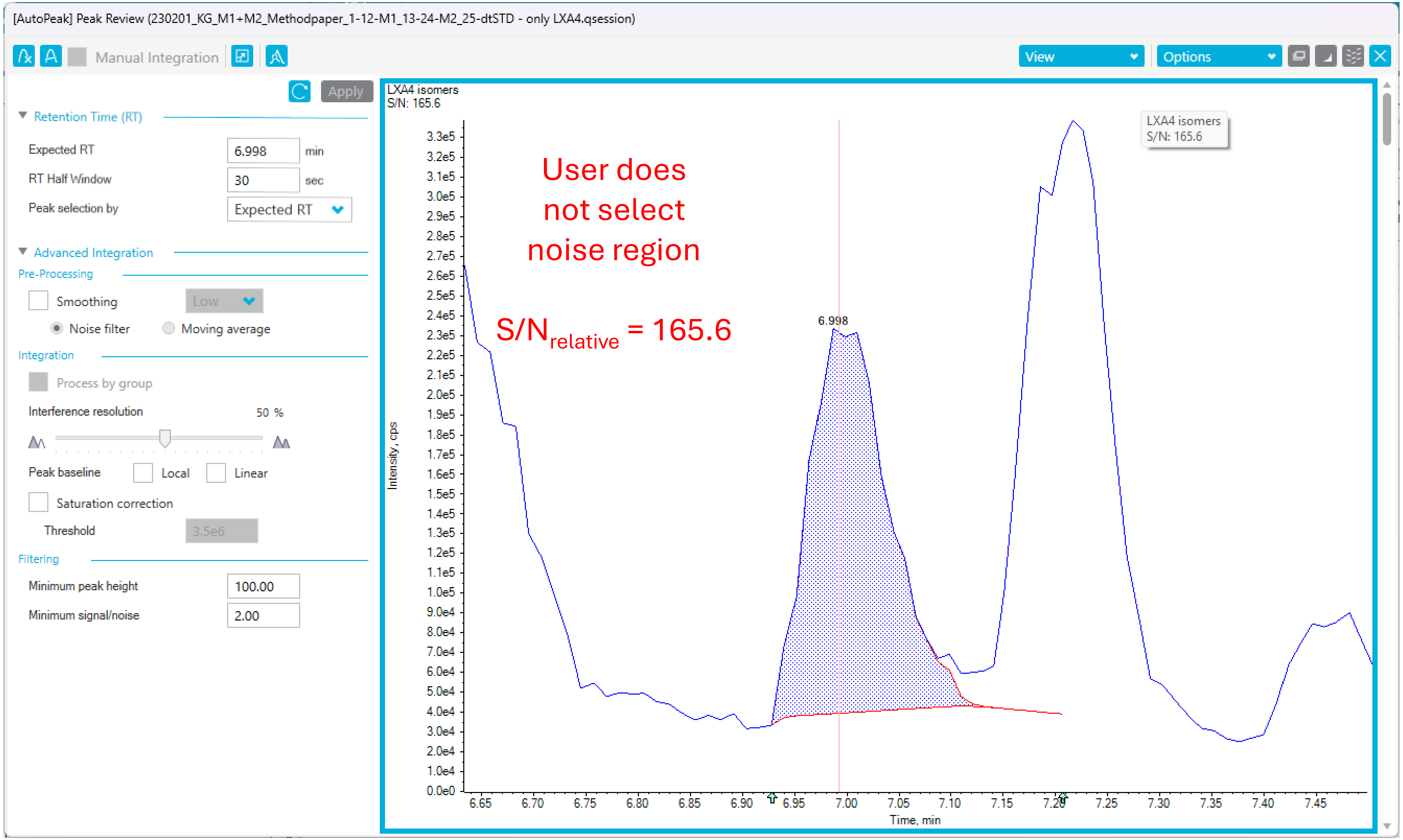

Fig. S6: Recovery

Relative recovery (%) of SPM by SPE. Standard solutions containing both external (ES) and internal standards (IS) were extracted by SPE (Sep-Pak Vac 6cc 500 mg/6 ml C18) and reconstituted in the same volume (n = 3). The resulting peak areas of ES were either compared directly (ES) or after IS-normalization (ES/IS) to areas obtained by direct injection without SPE. The experiment was then repeated with SPE-cartridges for which 20 cycles of re-use had been simulated (n = 4).
A) Individual recoveries as exemplified by RvD5. Results are shown as mean ± SD. Statistical analysis was performed by ordinary two-way ANOVA with Šidák's multiple comparisons test (single pooled variance). $*P < 0.05$.
B) Example chromatograms showing failure to recover RvD5 on aged columns.
C) Recovery of all SPM shown as average analyte loss (%) due to SPE.

A

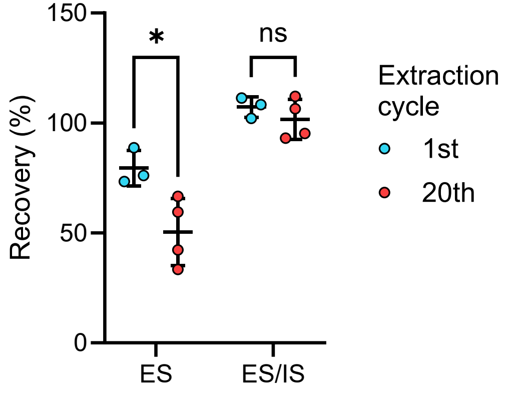

B

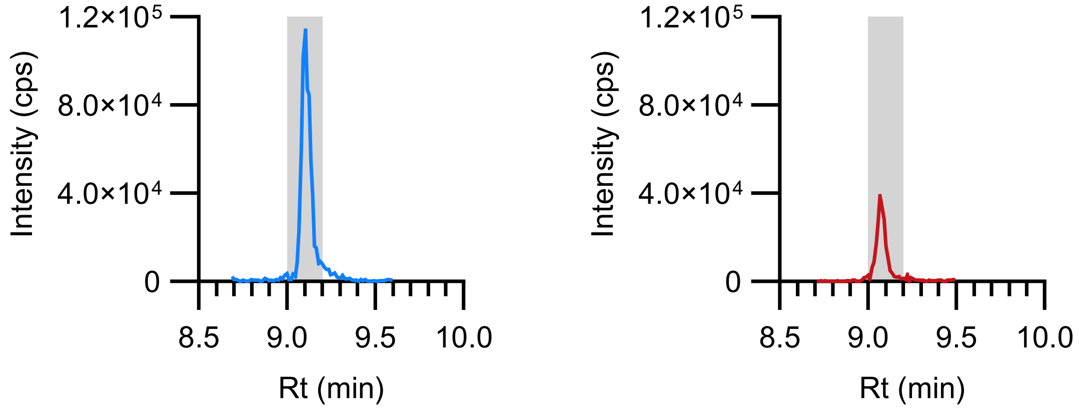

C

Analyte	First extraction cycle						20th extraction cycle					
	ES			ES/IS			ES			ES/IS		
	Mean	±	RSD	Mean	±	RSD	Mean	±	RSD	Mean	±	RSD
LXA_4	-25	±	8	13	±	5	-46	±	9	12	±	6
AT-LXA_4	-32	±	7	4	±	2	-45	±	10	14	±	7
LXA_5	-25	±	9	13	±	5	-45	±	9	15	±	6
LXB_4	-33	±	6	2	±	2	-39	±	13	26	±	11
MaR1	-23	±	5	5	±	9	-55	±	13	-9	±	10
MaR2	-31	±	7	-7	±	5	-61	±	15	-22	±	13
PD1	-28	±	7	-2	±	5	-51	±	18	-3	±	12
PDX	-17	±	9	11	±	4	-51	±	18	-3	±	13
RvD1	-23	±	10	4	±	3	-49	±	9	2	±	5
AT-RvD1	-28	±	9	-4	±	4	-49	±	10	1	±	6
RvD2	-29	±	9	-5	±	6	-43	±	11	12	±	11
RvD3	-22	±	10	4	±	3	-35	±	10	28	±	10
AT-RvD3	-16	±	11	12	±	3	-38	±	10	23	±	1
RvD4	-36	±	10	-14	±	2	-53	±	11	-7	±	9
RvD5	-21	±	8	7	±	5	-50	±	15	2	±	9
RvE1	-18	±	7	24	±	8	-29	±	7	50	±	10
RvE2	-27	±	5	-1	±	10	-45	±	13	14	±	18
RvE4	-15	±	11	14	±	4	-44	±	13	15	±	9
Mean	-25	±	8	5	±	5	-46	±	12	9	±	9

Fig. S7: Matrix effects of the homogenization buffer on the retention time

Representative chromatograms of PDX (filled) and PD1 (empty) in different media illustrating the retention time shift (0.1 min) caused by the homogenization buffer. Both PDX and PD1 share the same *m*/*z* used for quantification but differ in retention time by approximately 0.2 min. The retention time shift therefore must be taken into account to prevent mistaking PD1 for PDX in presence of homogenization buffer.

A) Standard mix in medium containing no homogenization buffer and injected without prior SPE.
Retention times: 8.9 min (PDX; filled) and 9.1 min (PD1; empty).

B) Blank homogenization buffer extracted by SPE.
No interfering oxylipins were observed.

C) Homogenization buffer spiked with standard mix and extracted by SPE.
Retention times: 8.8 min (PDX) and 9.0 min (PD1).

D) Homogenization buffer extracted by SPE and then spiked with standard mix.
Retention times: 8.8 min (PDX) and 9.0 min (PD1).

E) MilliQ spiked with standard mix and extracted by SPE.
Retention times: 8.9 min (PDX) and 9.1 min (PD1).

F) MilliQ extracted by SPE and then spiked with standard mix.
Retention times: 8.9 min (PDX) and 9.1 min (PD1)

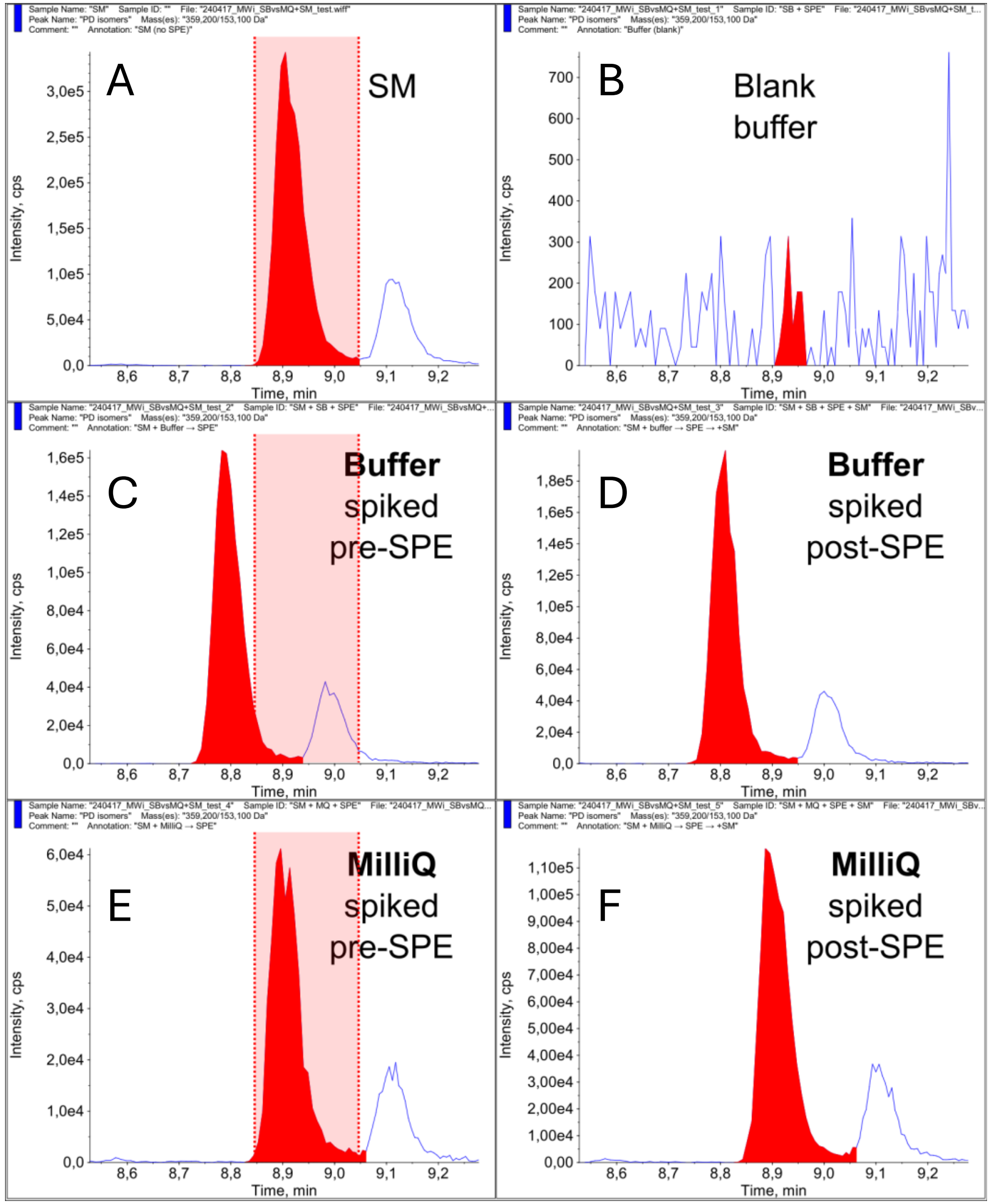

Fig. S8: Principal component analysis of *in vitro* and *ex vivo* oxylipin profiles

PBMC (2 × 10^6 cells), M1/M2a-MDM (2 × 10^6 cells), PMNL (10 × 10^6 cells), platelets (250 × 10^6 cells), or co-cultures of PMNL (10 × 10^6 cells) with platelets (250 × 10^6 cells) were left unstimulated, stimulated with A23187 (2.5 µmol/L), or stimulated with SACM (1%) for 90 min (n = 4). Whole blood (1 ml) was stimulated with A23187 (30 µmol/L) or stimulated with SACM (3%) for 90 min (n = 6). Oxylipins were extracted by SPE and determined by UHPLC-MS/MS. Results were analyzed by PCA using the R packages FactoMineR (https://cran.r-project.org/web/packages/FactoMineR/index.html) and factoextra (https://cran.r-project.org/web/packages/factoextra/index.html).

Left: Under unstimulated conditions, PCA analysis revealed clustering of *in vitro* models (PBMC, M1-MDM, M2a-MDM, PMNL, platelets, PMNL+platelet co-incubations) and clear separation from the *ex vivo* model (whole blood). **Middle**: Under A23187 stimulation, PCA analysis revealed four defined clusters: *i*) platelet-rich models (platelets, PMNL+platelets), *ii*) MDM models (M1, M2a), *iii*) primary leukocytes (PBMC and PMNL), and *iv*) whole blood. **Right**: Under SACM stimulation, PCA analysis revealed three defined clusters: *i*) platelets, PMNL, PBMC, blood; *ii*) MDM models (M1, M2a), *iii*) co-culture model (PMNL+platelets).

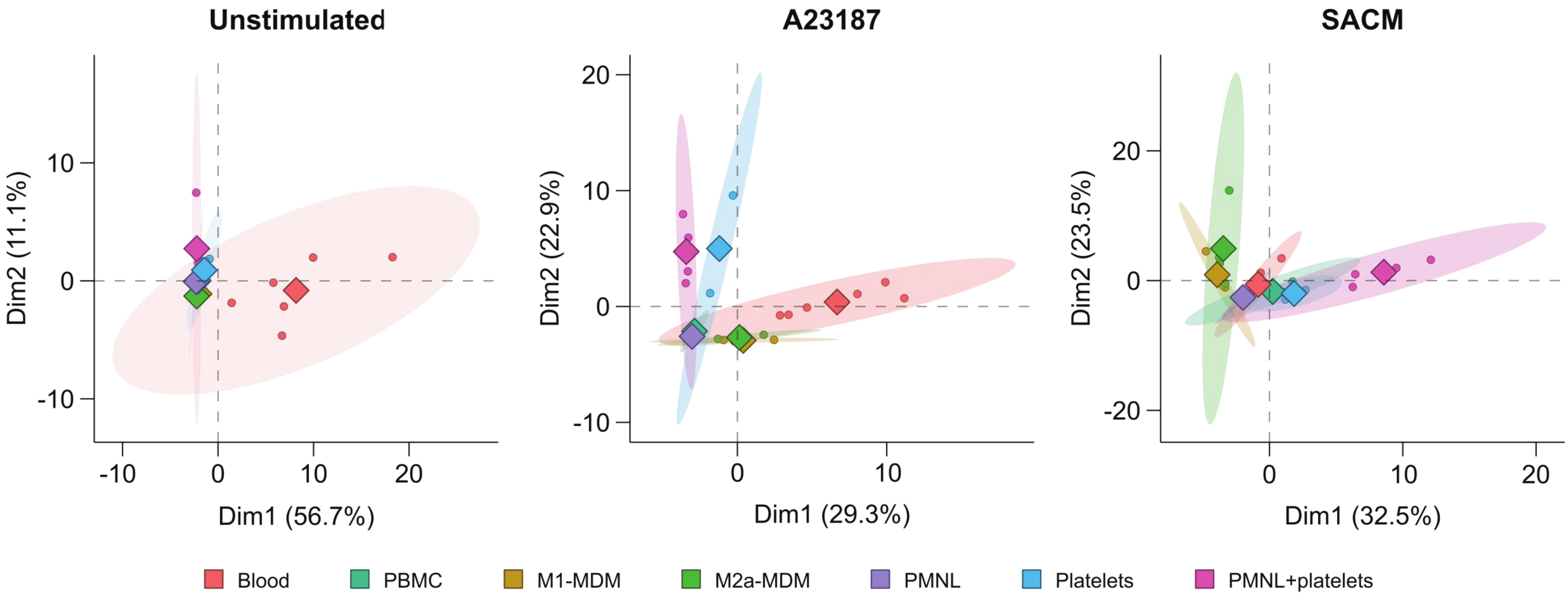

Supplement: Supplementary Material [file mmc4.pdf]
